# Characterizing the Metabolism of *Dehalococcoides* with a Constraint-Based Model

**DOI:** 10.1371/journal.pcbi.1000887

**Published:** 2010-08-19

**Authors:** M. Ahsanul Islam, Elizabeth A. Edwards, Radhakrishnan Mahadevan

**Affiliations:** Department of Chemical Engineering and Applied Chemistry, University of Toronto, Toronto, Ontario, Canada; ETH Zurich, Switzerland

## Abstract

*Dehalococcoides* strains respire a wide variety of chloro-organic compounds and are important for the bioremediation of toxic, persistent, carcinogenic, and ubiquitous ground water pollutants. In order to better understand metabolism and optimize their application, we have developed a pan-genome-scale metabolic network and constraint-based metabolic model of *Dehalococcoides*. The pan-genome was constructed from publicly available complete genome sequences of *Dehalococcoides* sp. strain CBDB1, strain 195, strain BAV1, and strain VS. We found that *Dehalococcoides* pan-genome consisted of 1118 core genes (shared by all), 457 dispensable genes (shared by some), and 486 unique genes (found in only one genome). The model included 549 metabolic genes that encoded 356 proteins catalyzing 497 gene-associated model reactions. Of these 497 reactions, 477 were associated with core metabolic genes, 18 with dispensable genes, and 2 with unique genes. This study, in addition to analyzing the metabolism of an environmentally important phylogenetic group on a pan-genome scale, provides valuable insights into *Dehalococcoides* metabolic limitations, low growth yields, and energy conservation. The model also provides a framework to anchor and compare disparate experimental data, as well as to give insights on the physiological impact of “incomplete” pathways, such as the TCA-cycle, CO_2_ fixation, and cobalamin biosynthesis pathways. The model, referred to as *i*AI549, highlights the specialized and highly conserved nature of *Dehalococcoides* metabolism, and suggests that evolution of *Dehalococcoides* species is driven by the electron acceptor availability.

## Author Summary

*Dehalococcoides* are strictly anaerobic bacteria capable of detoxifying widespread and harmful ground water pollutants — chlorinated ethenes, chlorinated benzenes, polychlorinated biphenyls and dioxins — largely originated from industry and agriculture sectors. However, how this unique niche has been acquired by these microbes is not well understood, specifically at the level of metabolism. In addition, these bacteria harness energy from the pollutant detoxification process — reductive dechlorination — for their growth; but they grow very slowly. Their slow growth rate, as well as their low growth yield, can hamper bioremediation processes. Thus, in order to obtain an improved understanding of *Dehalococcoides*' metabolism and the factors limiting their growth, we developed a constraint-based metabolic model of *Dehalococcoides* from publicly accessible genome sequences of 4 strains. This model, in addition to creating a valuable knowledgebase on *Dehalococcoides*, offers researchers in the bioremediation community a valuable tool for generating experimentally testable hypothesis for improving the efficacy of bioremediation by *Dehalococcoides*.

## Introduction

Genome sequencing has enabled the characterization of biological systems in a more comprehensive manner. Recent research in bioinformatics and systems biology has resulted in the development of numerous systematic approaches for the analysis of cellular physiology that have been reviewed elsewhere [1]–[4]. However, constraint-based reconstruction and analysis (COBRA), a mathematical framework for integrating sequence data with a plethora of experimental ‘omics’ data has been shown to be successful in the genome-wide analysis of cellular physiology [5]–. In addition, this approach has also been utilized to explore the metabolic potential, as well as the gene essentiality analysis of several organisms across different kingdoms of life [8]–[13]; however, the COBRA approach has not yet been implemented for *Dehalococcoides*, or any other known dechlorinating bacterium.

Using acetate as a carbon source and hydrogen as an electron donor, small, disc-shaped anaerobic bacteria *Dehalococcoides* are capable of dehalogenating a variety of halogenated organic compounds as electron acceptors, of which many are problematic ground water pollutants [14]–[17]. *Dehalococcoides* ethenogenes strain 195 (strain 195) is the first member of this phylogenetic branch that was grown as an isolate [18]. Subsequently, a number of *Dehalococcoides* strains were isolated: strain CBDB1 [19], strain BAV1 [20], strain FL2 [21], [22], strain GT [23], and strain VS [24]. The strains respire through a membrane-bound electron transport chain (ETC) [25]–[27], which is incompletely defined. Reductive dehalogenases (RDases), encoded by reductive dehalogenase homologous (*rdh*) genes, are pivotal membrane-associated enzymes of the ETC [26], [27]. Genome sequencing has revealed the presence of multiple non-identical putative *rdh* genes in each strain [28]–[31]. Since these microbes respire chlorinated pollutants by RDase-catalyzed reductive dechlorination reaction, *rdh* genes determine a significant part of *Dehalococcoides*' phenotypes. Functional characterization of only 5 of the over 190 *rdh* genes reveals that cobalamin — a corrinoid compound — is an essential cofactor for the corresponding RDases [32]–[36]. Hydrogenase (H_2_ase) is another key enzyme of *Dehalococcoides* ETC [26], [27], [29], [30]. Interestingly, the genomes of *Dehalococcoides* strains encode 5 different types of H_2_ases: membrane-bound *hup*, *ech*, *hyc*, *hym*, and cytoplasmic *vhu* [29], [30], [37], [38]. The presence of multiple types of H_2_ases clearly emphasizes the importance of H_2_ in their energy metabolism [18]–[21]. This multiplicity of H_2_ases and RDases further highlights redundancy in the organisms' energy conservation process that may ensure a rapid and efficient response of their energy metabolism towards changing growth conditions [39], [40].

In addition to RDase and H_2_ase, the ETC likely requires an in vivo electron carrier to mediate electron transport between these enzymes. Previous studies have shown that the reductive dechlorination reaction requires an in vivo electron donor of redox potential (E_0_^′^) ≤−360 mV [25], [27], similar to other dechlorinating bacteria [17], [41], [42]. The cob(II)alamin of corrinoid cofactor in the RDase enzyme is reduced to cob(I)alamin during the reductive dechlorination reaction; hence, necessitating a low-potential donor because the redox potential (E_0_′) of Co(II)/Co(I) couple is between −500 and −600 mV [17], [41], [43]. While quinones, such as menaquinone or ubiquinone could act as electron carriers in anaerobes [44]–[46], experimental evidence suggests this is not the case in *Dehalococcoides* [27], [47]. Moreover, the redox potentials for quinones (Menaquinone ox/red E_0_^′^ = −70 mV, Ubiquinone ox/red E_0_^′^ = +113 mV; [48]) are not compatible with the RDases' requirement of a low potential donor. Furthermore, cytochrome b — a typical donor for the quinones to participate in the redox reactions of anaerobic ETCs [49], [50] — appears to be absent in the genomes of *Dehalococcoides* [29], [30]. However, the genomes have ferredoxin, an iron-sulphur protein, which can act as the low-potential donor for RDases because ferredoxin is the most electronegative electron carrier yet found in the bacterial ETCs [42], [48], [51]–[55].

Although, *Dehalococcoides* are capable of harnessing free energy from the RDase catalyzed exergonic reductive dechlorination reactions by coupling to ATP generation for growth [14], [17], their pure culture growth is much less robust than their growth in mixed cultures [24], [56], [57]; even in mixed cultures, their growth yield is not as high as that predicted from the free energy of reductive dechlorination [26], [58]. Thus, in order to better understand dechlorination-metabolism, and given that to-date sequenced *Dehalococcoides* genomes are more than 85% identical at the amino acid level [38], [59], we developed a pan-genome-scale constraint-based *in silico* metabolic model of *Dehalococcoides*. The model was constructed from the complete genome sequences of 4 geographically distinct strains: strain CBDB1 from the Saale river near Jena, Germany [60], [61], strain BAV1 from Oscoda, Michigan, USA [62], [63], strain 195 from a wastewater treatment plant in Ithaca, New York, USA [18], [64], [65], and strain VS from Victoria, Texas, USA [24], [66]. Although the model comprises multiple genomes, it analyzed the outcome of metabolic genes only. Also, it did not include information about cellular regulation due to the lack of adequate knowledge about *Dehalococcoides* regulatory networks. Nonetheless, the model was primarily used to investigate the intrinsic metabolic limitations, in addition to addressing open questions regarding *Dehalococcoides* physiology, such as the incomplete nature of various metabolic pathways, and attendant implications on metabolism and growth. We also identified the environmental conditions from the model simulations that resulted in faster *in silico* growth of *Dehalococcoides*. Furthermore, the constraint-based model, along with the comparative analysis of 4 genomes, clarifies both similarities and differences among the strains in terms of their core metabolism and other biosynthetic processes leading to an improved understanding of metabolism and evolution in *Dehalococcoides*.

## Results/Discussion

### *Dehalococcoides* Metabolic Network

#### Pan-metabolic-genes of *Dehalococcoides*

**Figure 1:**
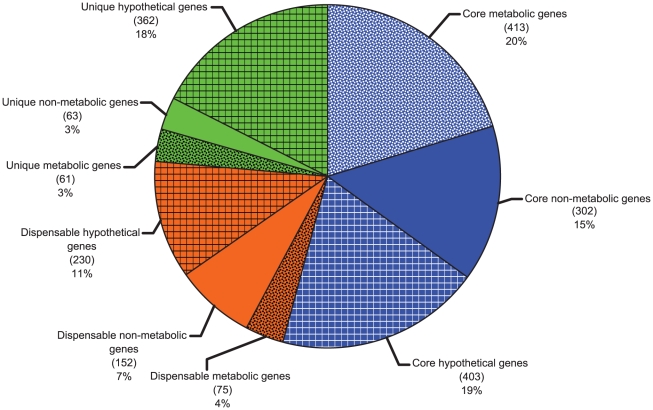
Composition of the *Dehalococcoides* pan-genome. Core, dispensable, and unique genomes are represented by blue, green, and orange, respectively. Genes in these genomes are also categorized as metabolic (spotted pattern), non-metabolic (plain), and hypothetical (grid pattern) on the basis of various bioinformatic analyses (see text for details).

**Figure 2:**
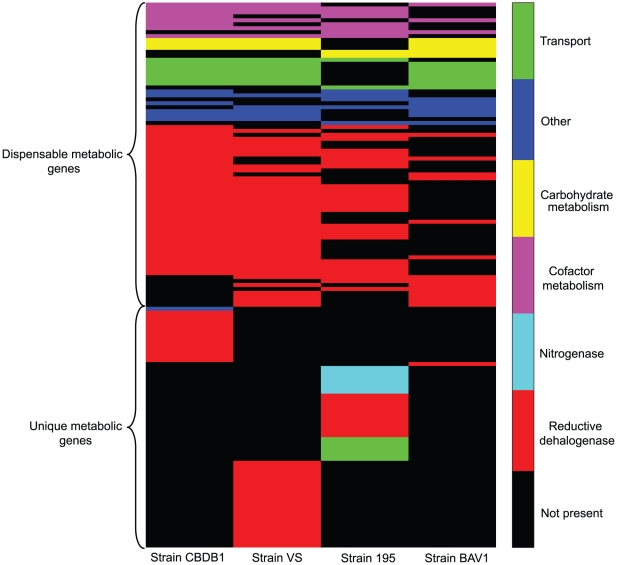
Distribution of dispensable and unique metabolic genes in different *Dehalococcoides* strains. Colors are assigned to further categorize the genes according to their function identified from annotation and verified by different bioinformatic analyses. Each color except black signifies the presence of a corresponding metabolic gene while black indicates the absence of the corresponding gene. Genes belonging to amino acid metabolism, lipid metabolism and nucleotide metabolism are small in number; hence, included in ‘other’ category. This heat map essentially describes the differences among *Dehalococcoides* strains from the context of metabolic genes.

**Figure 3:**
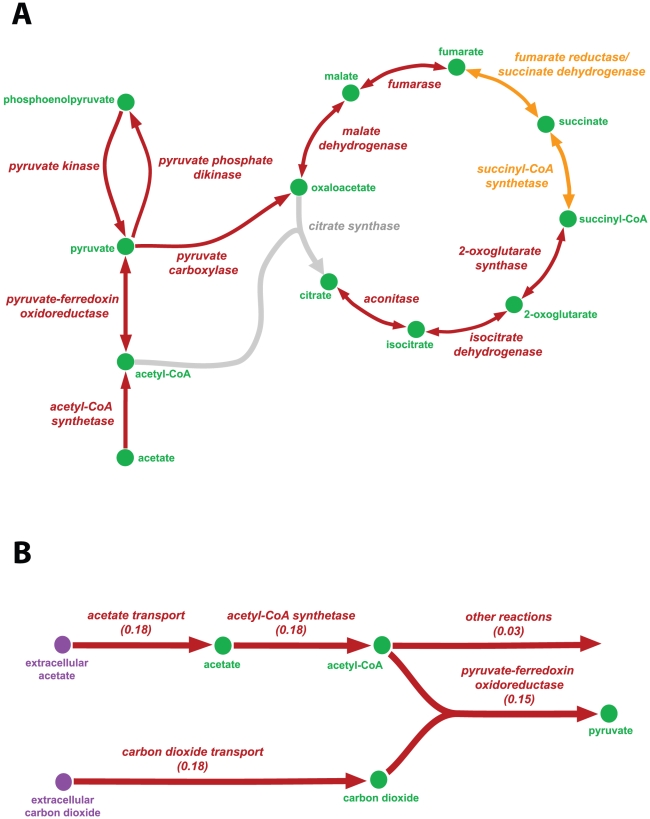
The reconstructed TCA-cycle and CO_2_ fixation pathway of *Dehalococcoides*. The arrows show the directionality of the reactions. (A) Grey: citrate synthase gene currently not identified in *i*AI549, but the pathway is suggested to be present by Tang et al. [84]; Orange: pathways for which homologous putative genes (∼30% amino acid sequence identity) were tentatively identified in *Dehalococcoides*, but are suggested to be absent by Tang et al. [84]; Red: pathways for which putative genes are confirmed to be present by both *i*AI549 and Tang et al. [84]. In all cases, the TCA-cycle of *Dehalococcoides* is not closed which explains their inability to use acetate as an energy source. (B) *Dehalococcoides'* requirement of CO_2_ in addition to acetate for their *in silico* growth. The numbers are flux values in mmol.gDCW^−1^.h^−1^. During pyruvate synthesis, *Dehalococcoides* require 67% carbon (molar basis) from acetate and 33% (molar basis) from CO_2_. Thus, *Dehalococcoides* fix carbon via the pyruvate-ferredoxin oxidoreductase or pyruvate synthase (POR) pathway.

**Figure 4:**
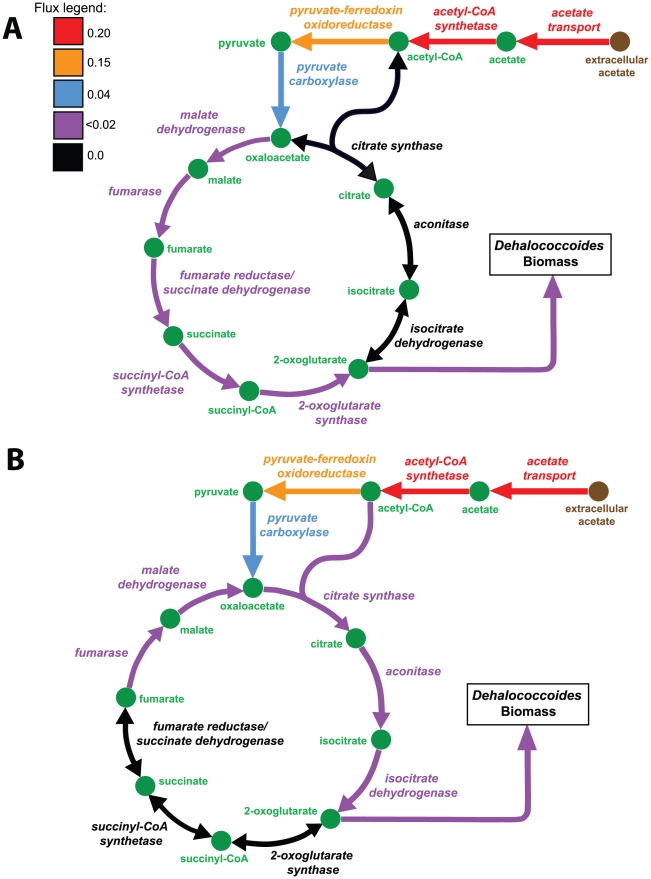
Analysis of the citrate synthase (CS) reaction on *Dehalococcoides* growth. (A) In the absence of the CS reaction, the TCA-cycle operates reductively via succinyl-CoA synthetase and 2-oxoglutarate synthase for producing biomass precursors for *Dehalococcoides* to grow. (B) The oxidative TCA-cycle operates when the CS reaction is present, but succinyl-CoA synthetase and 2-oxoglutarate synthase are absent, as suggested by the carbon labeling experiments [84]. However, *Dehalococcoides* growth remains almost unchanged with and without the CS reaction (0.0137 h^−1^ vs. 0.014 h^−1^) as represented by the flux values obtained from the growth simulations of *i*AI549.

The concept of a pan-genome was first investigated by Tettelin and colleagues for the 8 isolates of common human pathogen *Streptococcus agalactiae* [67]. While pan-genome analyses for other organisms have been reported [68], no such analysis has been performed to-date for any dechlorinating bacterium, or any other microbe of bioremediation importance. In addition, most of the reported pan-genome analyses were conducted on pathogenic isolates for designing vaccines by assessing their virulence evolution and diversity. Here, we developed the *Dehalococcoides* pan-genome from the complete sequenced genomes of four *Dehalococcoides* strains. Method details are provided in the Materials and Methods and in Figures 1–4 in Text S2. The pan-genome comprises 2061 genes (Figure 1). Of these 2061 genes, 1118 genes are in the core, 457 are dispensable, and 486 are unique (Figure 1). The genes are further classified as metabolic, non-metabolic, and hypothetical based on information obtained from the literature and various biochemical databases, such as SWISSPROT [69], UniProt [70], IMG [71], and PDB [72]. We defined metabolic genes as those that are exclusively related to metabolic processes such as carbon and energy metabolism of *Dehalococcoides*. Genes that are involved in DNA repair and metabolism, as well as encoding putative transposable elements and insertion elements [29], [30] are classified as non-metabolic. Putative genes with a non-specific metabolic function or genes without any function or annotation are categorized as hypothetical (Figure 1).

Most of the metabolic genes (413 out of 549) were found in the core genome while only a small number of those were identified in the dispensable (75) and unique (61) genomes. This abundance of core metabolic genes in the pan-genome indicate that the central metabolism of *Dehalococcoides* is very well conserved across strains since core genes are shared by all [2], [67], [73]–[75]. We further categorized the metabolic genes in the dispensable and unique genomes based on both function and strain (Figure 2). Clearly, the majority of differences among the strains (45 out of the 75 dispensable genes and 47 out of the 61 unique genes) are due to the *rdh* genes (Figure 2). In addition, only strain195 has nitrogen fixing genes and associated transporters related to the nitrogen fixation process. As a result of these genes, together with unique *rdh*s, strain 195 has the most unique genes of the 4 genomes compared. Due to the presence of a suite of multiple non-identical *rdh* genes, each strain metabolizes a unique set of specific chlorinated substrates [38], [76], [77]. Hence, the differences in *rdh* genes largely define the strain specific phenotypes of *Dehalococcoides*.

Though there were differences in *rdh* genes, most of these were found in the dispensable genome (Figure 2 and Table 14 in Text S1) while only 9 *rdh*s (5 *rdh*A and 4 *rdh*B genes with >35% amino acid sequence identity) were shared by all strains and found in the core genome (Table 13 in Text S1). The presence of majority of *rdh* genes in the dispensable genome further supports the hypothesis that they were acquired through lateral gene transfer events [29], [59], [78].

#### Features of the reconstructed metabolic network of *Dehalococcoides*

The reconstructed metabolic network of *Dehalococcoides*, denoted as *i*AI549 according to the established naming convention [79], accounted for 549 open reading frames (ORF) or protein coding genes (27% of the total 2061 genes). Metabolic genes were identified from the genome annotations which were verified with various bioinformatic analyses (see Materials and Methods). In addition, we annotated or revised the annotation for 70 ORFs based on information obtained from different biochemical databases (Table 2 in Text S1 provides a full list of reannotated genes). General features of *Dehalococcoides* metabolic network (*i*AI549) are provided in Table 1.

**Table 1:** General features of *Dehalococcoides* metabolic network (*i*AI549).

**Genes**	
Total number of genes	2,061
Number of included genes	549
Number of excluded genes	1,512
**Proteins**	
Total number of proteins	356
**Intra-system Reactions**	
Total number of model reactions	518
Gene associated model reactions	497
Non-gene associated model reactions	21
**Exchange Reactions**	
Total number of exchange reactions	36
Input-output reactions	35
Demand reactions	1
**Metabolites**	
Total number of metabolites	549
Number of extracellular metabolites	31
Number of intracellular biomass metabolites	110

*i*AI549 includes 518 model reactions and 549 metabolites where 497 reactions are gene associated and 21 (4%) are non-gene associated (Table 1). The non-gene associated reactions (Table 1 in Text S1) were added in order to fill gaps in the reconstructed network based on simulations. Although no gene associations were identified for these reactions, we provided a list of core hypothetical genes (Table 16 in Text S1) which potentially could contain genes associated with these reactions and are prime candidates for further biochemical testing. The network also comprises 36 exchange reactions, including one demand reaction called the biomass synthesis reaction (BIO_DHC_DM_61), to facilitate the transport of various metabolites into and out of the cell. The composition of the *in silico* minimal medium is shown in Table 2, while detailed composition of BIO_DHC_DM_61 is available in Text S2. We further categorized the genes and reactions of *i*AI549 into 7 different functional categories or subsystems based on the associated metabolic pathways (Figures 7 and 8 in Text S2). The differences among the strains are mainly observed in the energy metabolism category, which includes 51 dispensable and 54 unique metabolic genes, and most of these are *rdh*s (Figure 7 in Text S2). However, almost all the reactions of *i*AI549 (96% of the total 518) are core, which again indicates that the basic central metabolism of *Dehalococcoides* is strictly conserved (Figure 8 in Text S2). Although a number of dispensable metabolic genes are found in different subsystems, most of these genes are actually paralogs of the core metabolic genes. This relationship explains why, for example, there are 13 dispensable genes in the transport subsystem, 3 genes each in the lipid and nucleotide metabolism, but no corresponding dispensable reactions (Figure 8 in Text S2). Since *rdh*s were found in core, unique and dispensable genomes, we assigned the reductive dechlorination reaction as a core reaction. Therefore, the truly unique metabolic reactions of *i*AI549 are the nitrogen fixing reaction (EC-1.18.6.1) and the molybdate (required for synthesizing cofactor for the nitrogenase) transport reaction (TC-3.A.1.8) belonging to strain 195 only.

**Table 2:** Composition of the *in silico* minimal medium of *Dehalococcoides*.

Abbreviation	Exchange reaction	Equation
Acetate exchange	EX_ac(e)	ac < = = >
Vitamin B_12_ or cobalamin exchange	EX_cbl1(e)	cbl1 < = = >
Chloride exchange	EX_cl(e)	cl < = = >
Carbon dioxide exchange	EX_co2(e)	co2 < = = >
Proton exchange	EX_h(e)	h < = = >
Hydrogen exchange	EX_h2(e)	h2 < = = >
Water exchange	EX_h2o(e)	h2o < = = >
Dichlorobenzene exchange	EX_dcb(e)	dcb < = = >
Ethene exchange	EX_etl(e)	etl < = = >
Tetrachloroethene exchange	EX_pce(e)	pce < = = >
Hexachlorobenzene exchange	EX_hcb(e)	hcb < = = >
Ammonium exchange	EX_nh4(e)	nh4 < = = >
Inorganic phosphate exchange	EX_pi(e)	pi < = = >
Sulphate exchange	EX_so4(e)	so4 < = = >

Furthermore, we compared *i*AI549 to a number of *in silico* genome-scale models of other Bacteria and Archaea (Table 3): *i*AF1260 for *Escherichia coli* [80], *i*YO844 for *Bacillus subtilis* [81], *i*RM588 for *Geobacter sulfurreducens* [82], and *i*AF692 for *Methanosarcina barkeri* [83]. We found that *i*AI549 had the lowest number of total reactions because of the limited scope of *Dehalococcoides*' metabolism. In addition, these numbers also suggest that facultative anaerobes (*E. coli* and *B. subtilis*) are more versatile in their lifestyle and metabolism compared to obligate anaerobes (*Dehalococcoides*, *Geobacter* and *Methanosarcina*). These differences are further supported by the presence of a high number of transporters in *i*AF1260 and *i*YO844 compared to the presence of only 32 transporters in *i*AI549 (Table 3). A large number of reactions of *i*AI549 are found to be involved in the amino acid metabolism since the genes for *de novo* synthesis of all the amino acids except methionine are identified to be present in the genomes [29], [30]. Also, *i*AI549 comprises only 41 reactions for the central carbon metabolism — glycolysis, gluconeogenesis, TCA-cycle, pentose phosphate pathway, carbohydrate metabolism — compared to 262 reactions in *i*AF1260; an incomplete TCA-cycle and an inactive glycolysis pathway explain this low number for *i*AI549. Since *Dehalococcoides* lack a typical bacterial cell wall [18]–[20], *i*AI549 has only 81 reactions for the lipid metabolism category. Furthermore, the cofactor and prosthetic group biosynthesis comprises 101 reactions of *i*AI549 compared to 162 reactions of *i*AF1260 because the pathways for synthesizing vitamin B_12_ and quinones are predicted to be incomplete in *Dehalococcoides* [29], [30].

**Table 3:** Comparison of various *in silico* genome-scale models with *i*AI549.

*In silico* models (Organisms)	*i*AI549 (*Dehalococcoides*)	*i*RM588 (*G. sulfurreducens*)	*i*AF692 (*M. barkeri*)	*i*AF1260 (*E. coli*)	*i*YO844 (*B. subtilis*)
**Total reactions**	518	522	619	2077	1020
**Amino acid metabolism**	139	119	150	198	207
**Cofactor and prosthetic group biosynthesis**	102	100	153	162	83
**Nucleotide metabolism**	83	58	75	155	123
**Lipid metabolism**	81	93	46	522	126
**Central carbon metabolism**	41	64	72	252	196
**Energy metabolism**	40	37	41	90	41
**Transport**	32	51	82	698	244

### Model-Based Simulations of *Dehalococcoides* Physiology

#### Exploring the central metabolism of *Dehalococcoides*

The reconstructed network for glycolysis, gluconeogenesis, the TCA-cycle and the pentose phosphate pathway of *i*AI549 highlighted some of the key limitations of *Dehalococcoides* central metabolism. Although putative genes for glycolysis and gluconeogenesis were identified, no gene for a glucose or fumarate transporter was found in any of the genomes, explaining the inability of *Dehalococcoides* to use glucose or fumarate as a carbon source. The TCA-cycle of *Dehalococcoides* (Figure 3A) is incomplete, as previously reported [29], [30]. We could identify putative genes for 2-oxoglutarate synthase and succinyl Co-A synthetase (with 26% amino acid sequence identity to the *Methanococcus jannaschii* gene), and fumarate reductase/succinate dehydrogenase (with 31–33% amino acid sequence identity to the *E. coli* gene) (Table 3 in Text S1), but we could not find a gene encoding the citrate synthase (CS) in *Dehalococcoides*. In a scenario without CS, carbon assimilation could occur using a reductive TCA-cycle. However, the biosynthetic formation of citrate by *Dehalococcoides ethenogenes* strain 195 was recently demonstrated using ^13^C-labeled isotopomer experiments, although the gene encoding the putative *Re*-type CS enzyme was not identified [84]. The two *Dehalococcoides* genes that are most similar to the only biochemically characterized *Re*-type CS gene from *Clostridium kluyveri* DSM555 [85] are annotated as isopropyl malate and homocitrate synthase; however, these genes share only 27% amino acid sequence identity with CS gene from *C. kluyveri*. Hence, further experiments are required to establish the role of these genes, as well as the aforementioned putative TCA-cycle genes in *Dehalococcoides*. Nonetheless, these isotope labeling studies suggest the formation of 2-oxoglutarate from citrate through the oxidative branch of the TCA-cycle.

In order to analyze the effect of the presence of the CS reaction on *Dehalococcoides* growth, we conducted growth simulations with and without this reaction in *i*AI549. Only a subtle difference in the growth rate (0.0137 h^−1^ vs. 0.014 h^−1^) and yield (0.72 gDCW/eeq vs. 0.71 gDCW/eeq) was observed (Figures 4A, 4B and Table 32 in Text S2). Hence, regardless of whether the TCA- cycle is oxidative (Figure 4B) or reductive (4A), the fact that it is incomplete explains why *Dehalococcoides* are unable to use acetate as their energy source. Interestingly, *i*AI549 has one anaplerotic reaction — pyruvate carboxylase (PC) — which produces oxaloacetate from pyruvate (Figures 3A, 4A, and 4B). Generally, anaplerotic reactions generate intermediates of a TCA-cycle, but in the absence of a CS reaction, PC is essentially the sole pathway for producing oxaloacetate in the TCA-cycle of *i*AI549.

#### CO_2_-fixation by *Dehalococcoides*

Analysis of *i*AI549 also revealed the presence of a carbon fixation step via pyruvate-ferredoxin oxidoreductase or pyruvate synthase (POR) enzyme encoded by 4 putative *Dehalococcoides* genes (gene number 181, 182, 183, 184; Table 3 in Text S1). Anaerobes such as *Geobacter sulfurreducens* and *Methanosarcina barkeri* are also reported to utilize this step in their central metabolism [82], [86]. POR is essential for the *in silico* growth of *Dehalococcoides* using *i*AI549 since it is the only pathway for producing pyruvate from acetate (Figures 3A, 4A, and 4B). Growth simulations of *i*AI549 further predict that 33% of the total moles of carbon fixed into the biomass is from extracellular CO_2_ via POR, and the balance (67%) is from extracellular acetate through acetyl-CoA synthetase (Figure 3B); thus, clearly highlighting the important requirement for extracellular CO_2_ in addition to acetate as a carbon source for *Dehalococcoides*.

**Figure 5:**
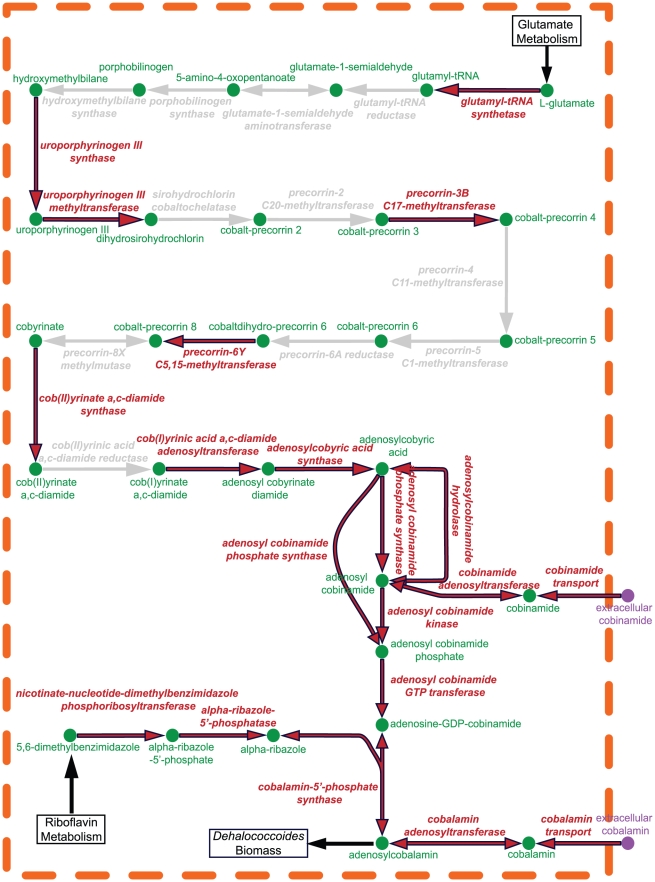
Reconstructed cobalamin biosynthesis pathway of *Dehalococcoides*. Dashed orange lines indicate cell membrane, grey lines indicate missing pathways, and red lines indicate existing pathways, putative genes of which are identified in *Dehalococcoides* during the reconstruction of *i*AI549. The arrows are denoting the directionality of the reactions. Since the genomes encode a putative cobalamin transporter, *Dehalococcoides* may salvage vitamin B_12_ either in the form of cobinamide or cobalamin from the environment as indicated by ‘cobinamide transport’ and ‘cobalamin transport’ reactions in the figure. The adenosylcobalamin, which is the end product of the entire pathway, is a biomass constituent and is assumed to take part in *Dehalococcoides* cell formation.

Moreover, the presence of both POR and carbon-monoxide dehydrogenase enzymes (CODHr) encoded by 4 putative genes of *i*AI549 (gene number 170, 171, 172, 174; Table 3 in Text S1) initially suggested that the Wood-Ljungdahl pathway [87] of CO_2_ fixation might be active in *Dehalococcoides*. However, the absence of several key enzyme encoding genes, such as the methylenetetrahydrofolate reductase and a methyltransferase in the folate-dependant branch of the Wood-Ljungdahl pathway [88]–[90] indicated that the pathway was incomplete in *Dehalococcoides* (Figure 5 in Text S2). All of these observations are consistent with the carbon labeling studies by Tang et al. [84].

#### Implications of the incomplete cobalamin synthesis pathway in *Dehalococcoides*

Cobalamin or vitamin B_12_ is essential for RDase activity; however, the pathway for producing cobalamin is incomplete in *Dehalococcoides* [29], [30], [91] (Figure 5). The complete *de novo* biosynthesis (aerobic or anaerobic) of vitamin B_12_ requires around 30 genes [92], of which only 18 (Tables 3–7 in Text S1) are identified in *Dehalococcoides*. Seven (7) of these genes belong to the “anaerobic” pathway while 2 are found to be involved in the “aerobic” pathway of cobalamin biosynthesis. Several key enzyme encoding genes required for the precorrin ring formation, cobalt insertion, and methylation were not found in *Dehalococcoides* genomes (Figure 5). However, 7 genes of *i*AI549 (3 core, 1 dispensable, and 3 unique genes: 161, 162, 163, 433, 524, 525, 526; Tables 3, 5, and 7 in Text S1) that encode a putative cobalamin transporter were identified; thus, indicating that *Dehalococcoides* could uptake vitamin B_12_ from the medium in the form of either cobinamide or cobalamin [93]. In fact, vitamin B_12_ has been shown to be required for the growth of pure cultures, and its addition to the medium has been reported to enhance the growth rate of *Dehalococcoides* [91].

**Figure 6:**
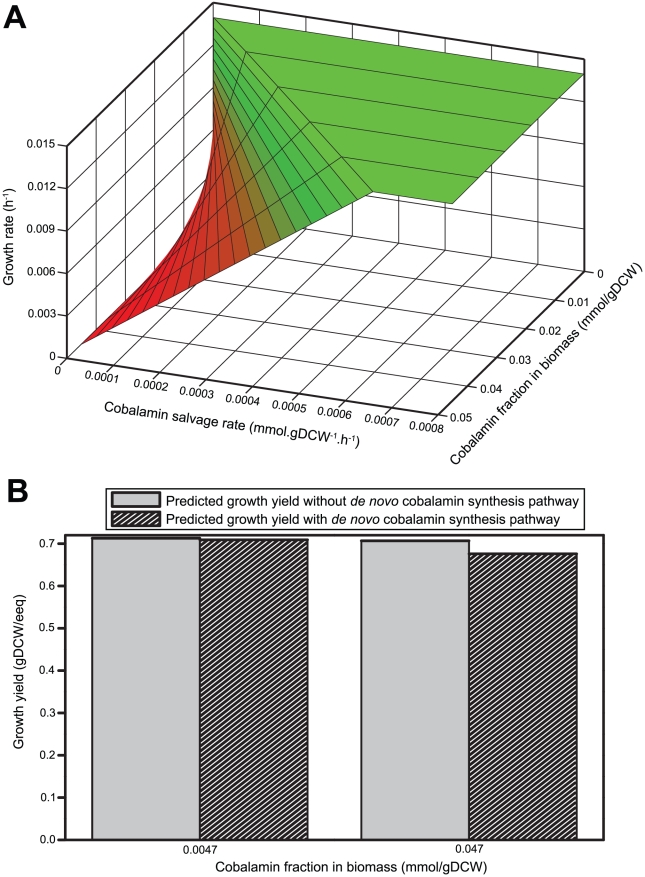
Influence of cobalamin on the growth rate and yield of *Dehalococcoides*. (A) Growth rate of *Dehalococcoides* is simulated as a function of both cobalamin salvage rate and cobalamin fraction in the biomass equation. It shows the role of cobalamin in limiting the growth rate of *Dehalococcoides*. Clearly, the cobalamin uptake or salvage rate at which *Dehalococcoides* growth is limiting increases with the increase of cobalamin fraction in the biomass. (B) The cost of *de novo* cobalamin synthesis in terms of *Dehalococcoides* growth yield is compared (see text for details). The predicted yield of *Dehalococcoides* with and without the *de novo* cobalamin synthesis pathway remains almost identical for the reported maximum cobalamin fraction in the biomass. However, the predicted yield decreased only by 4% (from 0.72 gDCW/eeq to 0.69 gDCW/eeq) with 10 fold increase of cobalamin fraction in the biomass indicating the low cost of *de novo* cobalamin synthesis.

Therefore, in order to examine the influence of cobalamin on the growth of *Dehalococcoides*, we conducted growth simulations for two scenarios using *i*AI549: 1) *Dehalococcoides* growth rate as a function of weight fraction of cobalamin in the biomass and cobalamin salvage rate from the medium (Figure 6A), and 2) *Dehalococcoides* growth yield assuming it could synthesize its own cobalamin (i.e., adding all the reactions to *i*AI549 required for *de novo* cobalamin synthesis) compared to the yield when B_12_ is salvaged from the medium (Figure 6B). Predictably, the growth rate decreases to zero at low cobalamin salvage rates (Figure 6A). Also, the cobalamin salvage rate at which metabolism becomes limited by B_12_ is a strong function of the cobalamin fraction in the biomass, which has never been experimentally measured for *Dehalococcoides* (Figure 6A). From the second simulation, it is clear that the energetic cost for synthesizing cobalamin *de novo* is not very significant since the predicted yield with and without a cobalamin synthesis pathway is almost identical (Figure 6B). Only if one assumes a biomass cobalamin fraction 10 times higher than the maximum reported, a small (4%) reduction in the growth yield (from 0.72 gDCW/eeq to 0.69 gDCW/eeq) is predicted as a penalty for synthesizing cobalamin *de novo* (Figure 6B and Table 31 in Text S2). This low synthesis cost, along with the fact that cobalamin is essential, yet its synthesis pathway is incomplete in *Dehalococcoides* suggests perhaps that *Dehalococcoides* might have evolved syntrophically with cobalamin secreters and never faced significant evolutionary pressure to acquire a complete cobalamin synthesis pathway in their genomes.

#### Does carbon or energy limit the *in silico* growth of *Dehalococcoides*?

**Figure 7:**
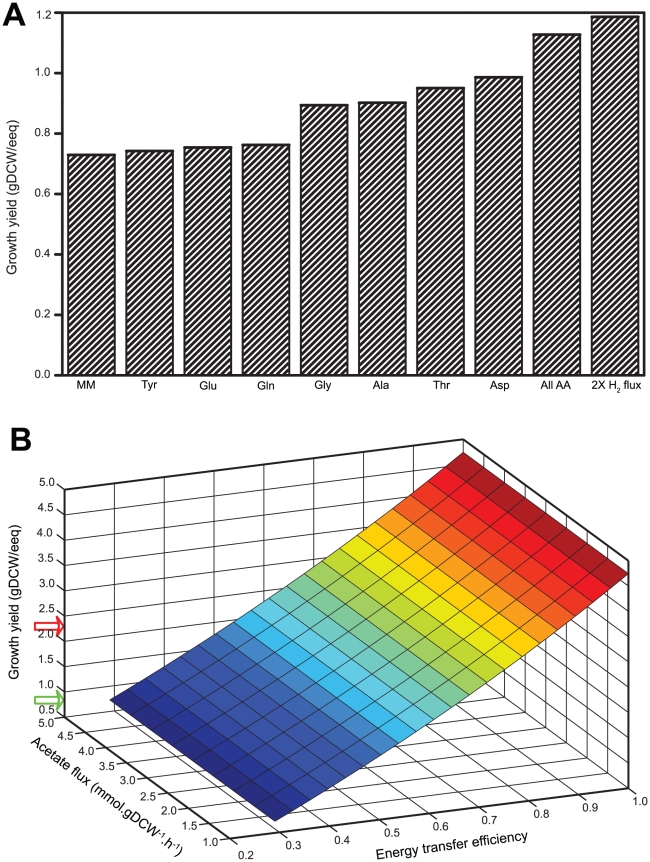
Effect of carbon and energy sources on the growth yield of *Dehalococcoides*. (A) The experimental growth yield of *Dehalococcoides* in the minimal medium (0.69 gDCW/eeq) is compared with increased growth yields achieved by allowing unlimited fluxes of all amino acids at a H_2_ flux of 10 mmol.gDCW^−1^.h^−1^ (corresponding to the experimental dechlorination rate), as well as doubling the H_2_ flux (20 mmol.gDCW^−1^.h^−1^). It shows that unlimited flux of amino acids (carbon source) increased the *in silico* growth yield of *Dehalococcoides* by 55%, whereas doubling the H_2_ flux (electron donor or energy source) alone enhanced the yield by 65%. (B) Analysis of the energy limited growth of *Dehalococcoides*. Since the growth yield of *Dehalococcoides* varies linearly with the energy transfer efficiency, their yield can be improved by increasing the flux of their energy source or electron donor to generate more ATP per electron. However, the variation in acetate fluxes has no effect on growth yields. Red and green arrows show growth yields and corresponding efficiencies for *Dehalococcoides* growth in mixed and pure cultures, respectively. ‘MM’ = minimal medium; ‘Tyr’ = tyrosine; ‘Glu’ = glutamate; ‘Gln’ = glutamine; ‘Gly’ = glycine; ‘Ala’ = alanine; ‘Thr’ = threonine; ‘Asp’ = aspartate; ‘All AA’ = all amino acids; ‘2X H_2_ flux’ = 20 mmol H_2_.gDCW^−1^.h^−1^.

Growth of *Dehalococcoides* is more rapid in mixed microbial communities than in pure cultures [24], [57], [60], [94] although the reasons for this discrepancy are not entirely clear. The difference in reported growth yields between pure and mixed cultures is more significant (p = 0.0005 at 95% confidence level) than the difference in reported growth rates (p = 0.05 at 95% confidence level) (Tables 25, 26 in Text S2). Thus, in order to examine the growth-limiting conditions, we simulated *Dehalococcoides* growth yields under two different conditions: 1) allowing unlimited flux of amino acids in the medium at a hydrogen flux of 10 mmol.gDCW^−1^.h^1^ (equivalent to the dechlorination rate obtained from average pure-culture growth yields and rates; Tables 24, 25 in Text S2), and 2) doubling the hydrogen flux (20 mmol. gDCW^−1^.h^−1^) without allowing any amino acid flux in the medium. The first condition mimics a carbon-rich environment while the second one represents an energy-rich situation. The model predicts that adding unlimited amount of any or all of the amino acids in the growth medium (obviating the need for the cell to synthesize these amino acids) increased the growth yield by a maximum of 55% (1.13 gDCW/eeq) compared to the case with no amino acids in the medium (0.72 gDCW/eeq) (Figure 7A). However, doubling only the hydrogen flux enhanced the growth yield by 65% (from 0.72 gDCW/eeq to 1.19 gDCW/eeq) (Figure 7A).

To further analyze this aspect of energy limitation, we simulated *in silico* growth yields of *Dehalococcoides* as a function of both acetate flux (carbon availability) and energy flux, represented by the energy transfer efficiency. This analysis (Figure 7B) shows that the growth of *Dehalococcoides* is energy-limited but not carbon-limited since growth yield increases proportionally to increase in energy transfer efficiency regardless of acetate flux. Moreover, simulations also reveal that growth yield of *Dehalococcoides* in a pure culture is only 30% efficient (corresponding to the green arrow) compared to 65% efficient (corresponding to the red arrow) in a mixed culture (Figure 7B). These simulations point towards electron flux from hydrogen to the RDase as the rate-limiting step, which is somehow more efficient in mixed cultures. It is possible that interspecies hydrogen transfer, such as in a mixed culture is more direct than hydrogen provided in the medium (as for pure cultures). Electrons supplied from an electrode that was polarized to a very low potential were shown to stimulate *Dehalococcoides* metabolism [95], possibly illustrating such an effect; if true, these results suggest a mechanism for the enhanced growth of *Dehalococcoides* and to a faster dechlorination of pollutants.

As described earlier, experimental studies clearly illustrate the favorable growth of *Dehalococcoides* in syntrophic microbial consortia compared to their isolated pure cultures. This obviously points towards the existence of some undefined beneficial metabolic interactions among the consortia members. Although *i*AI549 simulations suggested more efficient electron transfer and energy utilization in a mixed culture, this result requires further experimental validation. Because these microbes harness energy for their growth from reductive dechlorination reactions, their increased growth will certainly accelerate the bioremediation process. Hence, the current challenge is to understand the reason behind their favorable growth in a mixed microbial community prevailing in their natural habitat. Therefore, a genome-scale metabolic model of a syntrophic community of dechlorinating bacteria, where *Dehalococcoides* are the dominant members, can be useful to understand the factors influencing their growth. This information may also help to develop a defined bacterial community with enhanced bioremediation capability, in addition to developing effective strategies for exploiting these microbes for effective bioremediation of contaminated sites around the world.

### Conclusions

Although genome-scale constraint-based models are available for several microbes from all three forms of life, *i*AI549 is the first such endeavor for dechlorinating bacteria. This constraint-based flux balance model is consistent with the specialized nature of *Dehalococcoides* metabolism. The model supports the idea that evolution of the chlorinated compound specific *rdh* genes conferred the strain-specific metabolic phenotype to *Dehalococcoides*. In addition to cataloguing significant metabolic similarities among *Dehalococcoides* strains, the model also provides valuable insights regarding physiological and metabolic bottlenecks of these microbes. Reconstructed central metabolic pathways, for example, identified underlying reasons for *Dehalococcoides*' requirement of a separate energy source in addition to a carbon source for growth, as well as a carbon fixation step. Also, growth simulations revealed the energy-limited rather than carbon or cobalamin-limited growth of these organisms. In the process of developing the model, detailed tables of metabolic gene correspondences among 4 genomes, reannotations based on pathway analysis, and intrinsic kinetic and stoichiometric parameters were developed for the user community. We also created lists of core hypothetical genes and non-gene associated model reactions; these lists will be useful for designing enzyme assays for functional annotation of the hypothetical genes. Finally and most importantly, this pan-genome-scale metabolic model now provides a common and scalable framework as well as a knowledgebase, which can be used for visualization and interpretation of various omics-scale data from transcriptomics, proteomics and metabolomics for any *Dehalococcoides* strain; such analysis will further our understanding of these environmentally important organisms so that the outcome of bioremediation can be improved.

## Materials and Methods

### *Dehalococcoides* Pan-Genome

In order to develop the pan-genome of *Dehalococcoides*, we obtained strain CBDB1 genome sequence from JCVI (http://cmr.jcvi.org/tigr-scripts/CMR/CmrHomePage.cgi) while strain 195 and strain BAV1 genome sequences were downloaded from the IMG database (http://img.jgi.doe.gov/cgi-bin/pub/main.cgi). Strain VS genome sequence was obtained from Alfred Spormann at Stanford University, CA. The genome sequences were compared using OrthoMCL [96], a widely accepted method for finding orthologs across different genomes [97]. OrthoMCL is based on reciprocal best BLAST hit (RBH), but recognizes co-ortholog groups using a Markov graph clustering (MCL) algorithm [98]. The *Dehalococcoides* pan-genome was developed following a previously described approach [67], [68] outlined in Figures 1–4 in Text S2 and in the following section.

First, we identified putative orthologs between a reference genome and a subject genome which were selected arbitrarily from the 4 genomes compared. The analysis was conducted by OrthoMCL keeping the parameters of the algorithm in default settings. Subsequently, those genes that were present only in subject genome 1 were identified and combined with the reference genome to create the augmented genome 1 (Figure 1 in Text S2). Then, the augmented genome 1 was compared and analyzed with subject genome 2 as described above to construct the augmented genome 2. The pan-genome was obtained by comparing the augmented genome 2 and subject genome 3. The number of genes in a pan-genome was reported to depend on both the order of genomes analyzed and the reference genome [67]; hence, we constructed 6 pan-genomes for 6 different genome-order combinations. Of these 6 pan-genomes, we selected the one with the highest number of genes (2061) as *Dehalococcoides* pan-genome in order to capture the entire gene repertoire of *Dehalococcoides* species [74]. We also identified the core, dispensable and unique genomes for *Dehalococcoides* pan-genome using methods described previously [2], [67], (Figures 2–4 in Text S2).

### Reconstructing the Metabolic Network of *Dehalococcoides*

The pan-genome was used to reconstruct the pan-genome-scale metabolic network, and the constraint-based model of *Dehalococcoides* metabolism was developed from this reconstruction. Since the strains of *Dehalococcoides* share a high degree of sequence identity, we arbitrarily chose strain CBDB1 genome as a reference and constructed the metabolic network from its annotated genome sequence [29], publications regarding its physiology, and various genomic and biochemical databases [7]. Then, we included other metabolic genes from the pan-genome into the reconstructed network that were missing from strain CBDB1 genome. Five gene correspondence tables for the four genomes were prepared (Tables 3–7 in Text S1) for facilitating gene identification and cross-reference regardless of the genome of interest. We developed and manually curated the reconstructed network using the procedures described previously [3], [7], [99], [100] with the SimPheny platform (Genomatica Inc., San Diego, CA). Since genome annotations are error prone [101], annotated genes of strain CBDB1, as well as the pan-genome genes with defined metabolic functions were verified by identifying their homologs in other well characterized organisms, including *Escherichia coli*, *Bacillus subtilis*, *Geobacter sulfurreducens* and *Saccharomyces cerevisiae* with BLAST [102]. Subsequently, confidence levels were assigned based on the degree of sequence identities or reciprocal best BLAST hits. *Dehalococcoides* genes, for instance, having >40% amino acid sequence identity with homologs in the protein databases (SWISSPROT [103], IMG [71], PDB [72], GO [104]) were given a confidence level of 3, and genes with >30% and <30% identity were assigned a confidence level of 2 and 1, respectively. In addition, these genes were also evaluated on the basis of both gene order or conserved synteny [105], along with phylogenetic analysis with the updated versions of biological databases, such as UniProt [70], IMG [71], GO [104], and PDB [72]. Afterwards, both elementally and charge balanced biochemical reactions were assigned to the genes to create the gene-protein-reaction (GPR) associations [3]. These reactions were further verified by biochemical literature as well as enzyme databases, such as KEGG [106], BRENDA [107], MetaCyc [108], and ENZYME [109]. In some instances, genes required for some biosynthetic reactions essential for producing all the precursor metabolites for cell biomass were not identified. Such reactions (21 in number detailed in Table 1 in Text S1) were added to the reconstructed network as non-gene associated reactions.

### Estimation of Biomass Composition and Maintenance Energy Requirements

The biomass composition (dry basis) of 1 gram of *Dehalococcoides* cells was calculated from various published and experimental data, and expressed in mmol (millimoles)/g DCW (dry cell weight) (Tables 19–24 in Text S2). Due to the lack of detailed experimental data on the cellular composition of *Dehalococcoides*, the weight fractions of protein, lipid, carbohydrate, soluble pools and ions of the cell were estimated from the published genome-scale model of *Methanosarcina barkeri* [83]. We choose to use data from *M. barkeri* model — an archaeon — because *Dehalococcoides* cells are enclosed by the archaeal S-layer like protein instead of a typical bacterial cell wall [18]–[20]. The weight percent of DNA was estimated from the cell morphology, length of the genome sequence [110] and molar mass of the DNA, and the weight percent of RNA was calculated from the experimental data on a *Dehalococcoides* containing mixed microbial culture (see Text S2 for details). In addition, the detailed composition of each macromolecule as well as the composition of cofactors, and other soluble pools and ions are presented in Tables 19–24 in Text S2. The distribution of amino acids, nucleotides and cofactors in the biomass was calculated from the data reported previously [111], [112] while the weight fractions of different fatty acids were estimated from White et al. [113]. These compositions were then integrated into the model as a biomass synthesis reaction, BIO_DHC_DM_61 (see Text S2 for additional details).

Maintenance energy accounts for the ATP requirements of cellular processes, such as turnover of the amino acid pools, polymerization of cellular macromolecules, and ion transport, which are not included in the biomass synthesis reaction [114]–[116]. These ATP requirements can be either growth associated (GAM), i.e., related to assembly and polymerization of macromolecules (eg. proteins, DNA, etc.), or non-growth associated (NGAM) that corresponds to maintaining membrane potential for keeping cellular integrity [114], [115], [117]. Due to the lack of experimental chemostat data required for calculating both maintenance parameters [118], the NGAM for a *Dehalococcoides* cell (1.8 mmol ATP.gDCW^−1^.h^−1^) was calculated from the experimental decay rate (0.09 day^−1^) [24] and the average of pure-culture experimental growth yields (0.69 g DCW/eeq; Table 25 in Text S2) following the procedures described previously [114], [116]. The GAM was estimated by regression, using an initial estimate of 26 mmol ATP/g DCW for a typical bacterial cell (Table 28 in Text S2) [111]. The initial estimate of GAM and the calculated NGAM were then used to simulate (using flux balance analysis, described below) the average of reported pure-culture experimental growth rates (0.014 h^−1^; Table 26 in Text S2). A GAM of 61 mmol ATP/g DCW gave the best prediction of the experimental growth rate.

### *In Silico* Analysis of *Dehalococcoides* Metabolism

Flux Balance Analysis (FBA) relies on the imposition of a series of constraints including stoichiometric mass balance constraints derived from the metabolic network, thermodynamic reversibility constraints and any available enzyme capacity constraints [3], [119], [120]. The imposition of these constraints leads to a linear optimization (Linear Programming, LP) problem formulated to maximize a cellular objective function such as the growth rate. Hence, the biomass synthesis reaction is assumed to be the objective function to be maximized to solve the LP problem in SimPheny. In addition, a number of reversible reactions were added in the network for exchanging external metabolites, such as acetate (ac), chloride (Cl^−^), carbondioxide (CO_2_), sulphate (SO_4_^−2^) etc., to represent the *in silico* minimal medium (Table 2) for *Dehalococcoides*. As discussed earlier, cobalamin is essential for *Dehalococcoides* growth, but they are unable to synthesize it *de novo*; hence, they salvage cobalamin from the medium. In order to analyze whether cobalamin flux can limit *Dehalococcoides* growth, we performed a robustness analysis on the cobalamin exchange reaction for different weight fractions of cobalamin in the biomass. We also simulated growth rates by incorporating all the pathways required for *de novo* cobalamin synthesis in *i*AI549 for analyzing cobalamin synthesis cost, and its effect on *Dehalococcoides* growth. Finally, to identify whether the growth of *Dehalococcoides* was carbon or energy limited, the growth simulations were conducted by varying acetate fluxes and energy transfer efficiencies since acetate and H_2_ are the carbon and energy sources of these microbes, respectively [18]–[21], . Energy transfer efficiencies were calculated by normalizing the ATP fluxes to the maximum ATP that could be generated from H_2_ based on Gibb's free energy of H_2_ oxidation and the energetic cost of ATP synthesis (mol ATP/mol H_2_) (see Table 30 and Text S2 for additional details). The constraints set used to simulate *Dehalococcoides* growth is listed in Table 18 in Text S1, and the SBML file for the reconstructed network (*i*AI549) is presented as Dataset S1.

## Supporting Information

Dataset S1 SBML File for *Dehalococcoides* Metabolic Model, *i*AI549.
(0.48 MB XML)

Text S1 This file contains the detailed list of genes, proteins, reactions and metabolites included in the *Dehalococcoides* pan-metabolic-model, *i*AI549. Tables 3–7 are gene correspondences where a unique gene number is provided for each gene in the model (Model gene number) so that a gene or corresponding reaction can be located conveniently irrespective of the 4 genomes of interest. Strain VS gene locus names are obtained from Alfred Spormann at Stanford University, CA. A reaction can be associated with more than one gene where some genes are core, some are dispensable and others can be unique. For such instances, 2 additional gene correspondence tables (Table 5 and Table 7) are created for dispensable and unique genes respectively.
(5.41 MB PDF)

Text S2 This file contains the tables of detailed macromolecular composition of a gram of *Dehalococcoides* cell, experimental values of various pan-model parameters and the detailed procedure to calculate those parameters, as well as supplemental text regarding energy conservation process of *Dehalococcoides*. In addition, all the supplemental figures are included at the end of this document.
(0.31 MB PDF)
