# Supplementary material for: Characterizing the Metabolism of Dehalococcoides with a Constraint-Based Model
Source: PLoS Comput Biol. 2010 Aug 19;6(8):e1000887. doi: 10.1371/journal.pcbi.1000887 (PMC2930330; doi:10.1371/journal.pcbi.1000887)
Supplement: Text S1 — This file contains the detailed list of genes, proteins, reactions and metabolites included in the Dehalococcoides pan-metabolic-model, iAI549. Tables 3–7 are gene correspondences where a unique gene number is provided for each gene in the model (Model gene number) so that a gene or corresponding reaction can be located conveniently irrespective of the 4 genomes of interest. Strain VS gene locus names are obtained from Alfred Spormann at Stanford University, CA. A reaction can be associated with more than one gene where some genes are core, some are dispensable and others can be unique. For such instances, 2 additional gene correspondence tables (Table 5 and Table 7) are created for dispensable and unique genes respectively. (5.41 MB PDF) [file pcbi.1000887.s002.pdf]

Supplementary Information - 1

Characterizing the Metabolism of *Dehalococcoides* with a Constraint-based Model

M. Ahsanul Islam, Elizabeth A. Edwards, Radhakrishnan Mahadevan*

Department of Chemical Engineering and Applied Chemistry, University of Toronto

Toronto, ON M5S 3E5, Canada

*Corresponding author

Email addresses:
ahsan.islam@utoronto.ca
elizabeth.edwards@utoronto.ca
krishna.mahadevan@utoronto.ca

Supplementary information – 1: This file contains the detailed list of genes, proteins, reactions and metabolites included in the *Dehalococcoides* pan-metabolic-model, *i*AI549. Tables 3-7 are gene correspondences where a unique gene number is provided for each gene in the model (**Model gene number**) so that a gene or corresponding reaction can be located conveniently irrespective of the 4 genomes of interest. Strain VS gene locus names are obtained from Alfred Spormann at Stanford University, CA. A reaction can be associated with more than one gene where some genes are core, some are dispensable and others can be unique. For such instances, 2 additional gene correspondence tables (Table 5 and Table 7) are created for dispensable and unique genes respectively.

Table of Contents

Table 1. List of Non-gene Associated Reactions Included in *i*AI549 ········ 2
Table 2. List of Reannotated Genes of Different *Dehalococcoides* Strains ········ 4
Table 3. Gene Correspondence for Core Genes Mapped to Reactions of *i*AI549 ········ 16
Table 4. Gene Correspondence for Dispensable Genes Mapped to New Reactions of *i*AI549 ········ 63
Table 5. Gene Correspondence for Dispensable Genes Mapped to Reactions of *i*AI549 Already Present in Core ········ 65
Table 6. Gene Correspondence for Unique Genes Mapped to New Reactions of *i*AI549 ········ 73
Table 7. Gene Correspondence for Unique Genes Mapped to Reactions of *i*AI549 Already Present in Core ········ 75
Table 8. Detailed List of Proteins of *i*AI549 ········ 81
Table 9. List of Reactions of *i*AI549 Associated with Core Genes (Core Reactions) ········ 94
Table 10. List of Reactions of *i*AI549 Associated with Dispensable Genes (Dispensable Reactions) ········ 125
Table 11. List of Reactions of *i*AI549 Associated with Unique Genes (Unique Reactions) ········ 127
Table 12. Detailed List of Metabolites of *i*AI549 ········ 128
Table 13. Core Reductive Dehalogenase Homologous (*rdh*) Genes of *i*AI549 ········ 155
Table 14. Dispensable Reductive Dehalogenase Homologous (*rdh*) Genes of *i*AI549 ········ 156
Table 15. Unique Reductive Dehalogenase Homologous (*rdh*) Genes of *i*AI549 ········ 160
Table 16. List of Core Hypothetical Genes of *Dehalococcoides* Pan-genome ········ 164
Table 17. List of Exchange Reactions of *i*AI549 ········ 189
Table 18. Set of Constraints for Simulating *Dehalococcoides* Growth using *i*AI549 ········ 191

Table 1. List of Non-gene Associated Reactions Included in *i* AI549

No	Abbreviation	Name	Equation	Subsystem	Reason for adding
1	AGMT	agmatinase	[c] : agm + h2o --> ptrc + urea	Amino Acid Metabolism	Required for putrecine synthesis.
2	ALAR	alanine racemase	[c] : ala-L <==> ala-D	Amino Acid Metabolism	Required for D-alanine biosynthesis.
3	APSPT	adenosine 5'-phosphosulfate reductase;	[c] : aps + trdrd <==> amp + (2) h + so3 + trdox	Amino Acid Metabolism	Required for cysteine biosynthesis.
4	G5SADs	L-glutamate 5-semialdehyde dehydratase (spontaneous)	[c] : glu5sa <==> 1pyr5c + h + h2o	Amino Acid Metabolism	Spontaneous reaction. Required for proline synthesis.
5	HCO3E	carbonate dehydratase (HCO3 equilibration reaction)	[c] : co2 + h2o <==> h + hco3	Amino Acid Metabolism	Required for arginine biosynthesis.
6	HSPMS	Homospermidine synthase	[c] : (2) ptrc --> hspmd + nh4	Amino Acid Metabolism	Required for homospermidine synthesis.
7	METS	methionine synthase	[c] : 5mthf + hcys-L --> h + met-L + thf	Amino Acid Metabolism	Required for methionine synthesis.
8	METACH	O-Acetyl-L-homoserine acetate-lyase (adding methanethiol)	[c] : achms + h2s <==> ac + hcys-L	Amino Acid Metabolism	Required for methionine synthesis.
9	MTHFR1	5,10-methylenetetrahydrofolate reductase (NADP)	[c] : (2) h + mlthf + nadph --> 5mthf + nadp	Amino Acid Metabolism	Required for methionine biosynthesis.
10	OIVD1	2-oxoisovalerate dehydrogenase (acylating; 4-methyl-2-oxopentaoate)	[c] : 4mop + coa + nad <==> co2 + ivcoa + nadh	Amino Acid Metabolism	Required for biomass synthesis
11	OIVD2	2-oxoisovalerate dehydrogenase (acylating; 3-methyl-2-oxobutanoate)	[c] : 3mob + coa + nad --> co2 + ibcoa + nadh	Amino Acid Metabolism	Required for biomass synthesis
12	OIVD3	2-oxoisovalerate dehydrogenase (acylating; 3-methyl-2-oxopentanoate)	[c] : 3mop + coa + nad --> 2mbcoa + co2 + nadh	Amino Acid Metabolism	Required for biomass synthesis
13	OMCDC	2-Oxo-4-methyl-3-carboxypentanoate decarboxylation	[c] : 3c4mop + h --> 4mop + co2	Amino Acid Metabolism	Spontaneous reaction. Required for leucine biosynthesis.
14	SULR	sulfite reductase (NADPH2)	[c] : (3) h2o + h2s + (3) nadp <==> (4) h + (3) nadph + so3	Amino Acid Metabolism	Required for cystein biosynthesis.

No	Abbreviation	Name	Equation	Subsystem	Reason for adding
15	X00005	Lumped reaction for degradation of glycoaldehyde	[c] : coa + gcald --> accoa + h2o	Cofactor and Prosthetic Group Biosynthesis	Required for glycolaldehyde degradation so that 5,6,7,8-tetrahydrofolate can be synthesiszed.
16	ATPM	ATP maintenance requirment	[c] : atp + h2o --> adp + h + pi	Energy Metabolism	Modeling requirement
17	ACt6	acetate transport in/out via proton symport	ac[e] + h[e] <==> ac[c] + h[c]	Transport	Acetate is the carbon source for *Dehalococcoides*
18	CO2t	CO2 transport out via diffusion	co2[e] <==> co2[c]	Transport	Diffusion process
19	H2Ot5	H2O transport via diffusion	h2o[e] <==> h2o[c]	Transport	Diffusion process
20	H2td	Hydrogen transport	h2[c] <==> h2[e]	Transport	Diffusion process
21	N2t	Nitrogen transport	n2[c] <==> n2[e]	Transport	Diffusion process, required for N2 fixation

Table 2. List of Reannotated Genes of Different *Dehalococcoides* Strains

No	CBDB1 gene locus	195 gene locus	BAV1 gene locus	VS gene locus	Primary annotation	Putative reannotation	Model protein	Model reaction	Reason for reannotation	Confidence level*
1	cbdbA143	DET0123	DehaBAV1_0247	VS131	pyridine nucleotide-disulphide oxidoreductase family protein	Glutamate synthase small chain	GltB	GLUSy	Assigned based on 28% sequence identity with G. sulfurreducens in IMG, JGI, 25% in PDB, 30% in SWISSPROT, GO annotation and synteny in IMG, JGI. Required for glutamate synthesis	2
2	cbdbA182	DET0153	DehaBAV1_0217	VS162	hypothetical protein	4-carboxymuconolactone decarboxylase	PcaC2	CMLDC	Assigned based on 30% sequence identity with Desulfatibacillum alkenivorans AK-01 in IMG, JGI and in SWISSPROT, 31% with Ruegeria pomeroyi DSS-3 in GO database.	2
3	cbdbA196	DET0189	DehaBAV1_0164	VS177	putative methyltransferase	Asparagine synthetase	AsnA	ASNS2	Assigned based on 29% sequence identity with Streptococcus pneumoniae SP19-BS75 in IMG, JGI, and EC equivalogs (TIGR protein family) in IMG, JGI. Also required for asparagine synthesis.	1
4	cbdbA247	DET0240	No gene	VS78	precorrin methylase	precorrin-6Y C5,15-methyltransferase	CbiE	CPC6MT	Assigned based on 23% sequence identity with Clostridium kluyveri DSM 555 in IMG, JGI, 26% with Methanococcus jannaschii in SWISSPROT and on GO annotation.	1
5	cbdbA254	DET0246	No gene	VS72	cobalamin biosynthesis protein	Adenosylcobinamide-phosphate synthase	CbiD	ADCOBAS, ADCPS2	Assigned based on 36% sequence identity with Pseudomonas putida KT2440 in IMG, JGI, and SWISSPROT.	2
6	cbdbA259	DET0250	No gene	VS68	putative precorrin methylase	Vitamin B12 transport protein	BtuF	CBL1abc	Assigned based on 28% sequence identity with Burkholderia oklahomensis EO147 in IMG-JGI and SWISSPROT, 28% in PDB with E.coli and GO annotation.	1
7	cbdbA260	DET0297	No gene	No gene	conserved hypothetical protein	Pyridoxamine 5'-phosphate oxdase	PdxHec	PYAM5PO, PDX5PO	Assigned based on 29% sequence identity with Methanococcus aeolicus Nankai-3 in IMG-JGI, SWISSPROT and in PDB.	1

No	CBDB1 gene locus	195 gene locus	BAV1 gene locus	VS gene locus	Primary annotation	Putative reannotation	Model protein	Model reaction	Reason for reannotation	Confidence level*
8	cbdbA327	DET0380	DehaBAV1_0362	VS324	pyridoxine biosynthesis protein	Pyridoxamine 5'-phosphate synthase	PdxAJ	PDX5PS	Assigned based on 64% sequence identity with Clostridium kluyveri DSM 555 in IMG-JGI, 66% with Geobacillus sp. H6a in SWISSPROT, 67% in PDB and GO annotation.	3
9	cbdbA331	DET0386	DehaBAV1_0366	VS329	conserved hypothetical protein TIGR00096	Uroporphyrinogen III cosynthase	HemD	UPP3MT, UPP3S	Assigned based on 24% sequence identity in Swissprot curated database with Clostridium josui, 24% with Thermus thermophilus in PDB, 51% with Nostoc punctiforme PCC 73102 in IMG, JGI.	1
10	cbdbA359	DET0407	DehaBAV1_0386	VS350	conserved domain protein	Mannose-1-phosphate guanylyltransferase	ManC	MAN1PT2	Assigned based on 62% sequence identity with Salmonella enterica in SWISSPROT.	3
11	cbdbA367	DET0414	DehaBAV1_0393	VS357	conserved hypothetical protein	Diacylglycerol kinase	DgkA	DAGK	Assigned based on EC equivalogs (TIGR protein family) and high sequence identity with Dehalococcoides BAV1, VS; 33% identity with Anaeromyxobacter sp. (strain Fw109-5) in IMG and SWISSPROT and 31% identity in PDB.	2
12	cbdbA381	DET0428	DehaBAV1_0405	VS370	transcriptional regulator	Pantothenate kinase	CoaA	PNTK	Assigned based on 47% sequence identity in PDB, 46% with B. subtilis and 49% with G. sulfurreducens in SWISSPROT, and GO annotation.	3
13	cbdbA392	DET0438	DehaBAV1_0415	VS380	metallo-beta-lactamase family protein	Hydroxyacylglutathione hydrolase	GloB	GLYOX	Assigned based on EC equivalogs (TIGR protein family), 29% sequence identity with E. coli, 33% with Bacillus cereus ATCC 14579, and 26% in PDB.	2
14	cbdbA400	DET0444	DehaBAV1_0421	VS386	conserved hypothetical protein	Glucose 1-phosphate adenylyltransferase	GlgC	GLGC	Assigned based on 45% sequence identity with Erythrobacter sp. NAP1 and EC equivalogs in KEGG.	3
15	cbdbA416	DET0456	DehaBAV1_0433	VS398	MutT	ADP-ribose disphosphatase/pyrophosphatase	NudF	ADPRDP	Assigned based on 39% sequence identity with Bacillus sp. SG-1 in SWISSPROT and IMG, 34% in PDB, EC equivalogs and GO annotation.	3

No	CBDB1 gene locus	195 gene locus	BAV1 gene locus	VS gene locus	Primary annotation	Putative reannotation	Model protein	Model reaction	Reason for reannotation	Confidence level*
16	cbdbA417	DET0457	DehaBAV1_0434	VS399	acetyltransferase, GNAT family	N-acetylglutamate synthase	ArgA	ACGS	Assigned based on 49% sequence identity with Pelotomaculum thermopropionicum SI in IMG-JGI, 56% with Pelobacter carbinolicus in SWISSPROT, 24% in PDB and GO annotation.	3
17	cbdbA419	DET0458	DehaBAV1_0435	VS400	inorganic polyphosphate	NAD kinase	NadK	NADK	Assigned based on 38% sequence identity with G. sulfurreducens in IMG-JGI, SWISSPROT, 33% in PDB and GO annotation.	3
18	cbdbA472	DET0509	DehaBAV1_0485	VS450	SIS domain protein	Mannose-6-phosphate isomerase and glucose-6-phosphate isomerase	Pgi, ManA	MAN6PI, PGI	Assigned based on EC equivalogs (TIGR protein family), annotation provided by JGI, IMG, 28% sequence identity with Metallosphaera sedula DSM 5348 and 25% sequence identity in PDB.	1
19	cbdbA484	DET0518	DehaBAV1_0494	VS459	translation initiation factor, putative,aIF-2BIfamily	5-methylthioribose-1-phosphate isomerase	YkrS	MTRI	Assigned based on 58% sequence identity with Geobacter metallireducens, 50% with Desulfuromonas acetoxidans DSM 684 in IMG-JGI and SWISSPROT, GO annotation and 56% in PDB.	3
20	cbdbA490	DET0523	DehaBAV1_0499	VS464	acetyltransferase, GNAT family	N-acetylglutamate synthase	ArgA	ACGS	Assigned based on 49% sequence identity with Pelotomaculum thermopropionicum SI in IMG-JGI, 56% with Pelobacter carbinolicus in SWISSPROT, 24% in PDB and GO annotation.	3
21	cbdbA514	DET0540	DehaBAV1_0516	VS481	replicative DNA helicase	Dihydroneopterin triphosphate pyrophophorylase	NtpA	DNMPPA, DNTPPA	Assigned based on EC equivalogs and 33% sequence identity with Campylobacter concisus 13826.	2
22	cbdbA520	DET0545	DehaBAV1_0521	VS486	amidohydrolase family protein	Pantetheine hydrolase/succinyl-diaminopimelate desuccinylase	DapE, Phyd	SDPDS, PNTEH	Assigned based on 36% sequence identity with Geobacter sulfurreducens in IMG-JGI, SWISSPROT and EC equivalogs.	2
23	cbdbA553	DET0576	DehaBAV1_0549	VS515	aminotransferase, classes I and II	Aspartate aminotransferase	AspC	ASPTA1	Assigned based on 34% sequence identity with Korarchaeum cryptofilum (strain OPF8) in SWISSPROT and 24% in PDB.	2

No	CBDB1 gene locus	195 gene locus	BAV1 gene locus	VS gene locus	Primary annotation	Putative reannotation	Model protein	Model reaction	Reason for reannotation	Confidence level*
24	cbdbA610	DET0624	DehaBAV1_0597	VS565	response regulator	Adenylate cyclase	CyaA	ADNCYC	Assigned based on EC equivalogs and 32% sequence identity with Beggiatoa sp. PS, 29% with Ralstonia eutropha JMP134 in IMG-JGI and SWISSPROT.	1
25	cbdbA637	DET0687	DehaBAV1_0623	VS591	conserved hypothetical protein	Adenosylcobinamide hydrolase	CbiZ	ADCOBHS	Added based on 29% sequence identity with Rhodobacter sphaeroides 2.4.1 and 36% with Methanocaldococcus jannaschii DSM 2661.	2
26	cbdbA667	DET0711	DehaBAV1_0646	VS618	carbohydrate kinase, PfkB family	Ribokinase	RbsK	RBK	Assigned based on 28% sequence identity in PDB, 32% with Methanosphaera stadtmanae in SWISSPROT and GO annotation	2
27	cbdbB16	DET0724	DehaBAV1_0656	VS630	pyruvic-ferredoxin oxidoreductase, gammasubunit	Pyruvate synthase/2-oxoglutarate synthase	Ofo, Pfor	OOR2r, POR2	Assigned based on 50% sequence identity with Moorella thermoacetica, 50% with M. barkeri in swissprot, 46% in PDB with Thermotoga maritima and GO annotation. For 2-oxoglutarate synthase, function assigned based on Based on 41% sequence identity with Thermoproteus tenax in swissprot, 39% with W. succinogens and GO annotation.	3
28	cbdbA680	DET0725	DehaBAV1_0657	VS631	pyruvic-ferredoxin oxidoreductase, delta subunit	Pyruvate synthase/2-oxoglutarate synthase	Ofo, Pfor	OOR2r, POR2	Assigned based on 50% sequence identity with Moorella thermoacetica, 50% with M. barkeri in swissprot, 46% in PDB with Thermotoga maritima and GO annotation. For 2-oxoglutarate synthase, function assigned based on Based on 41% sequence identity with Thermoproteus tenax in swissprot, 39% with W. succinogens and GO annotation.	3

No	CBDB1 gene locus	195 gene locus	BAV1 gene locus	VS gene locus	Primary annotation	Putative reannotation	Model protein	Model reaction	Reason for reannotation	Confidence level*
29	cbdbA681	DET0726	DehaBAV1_0658	VS632	pyruvic-ferredoxin oxidoreductase, alpha subunit	Pyruvate synthase/2-oxoglutarate synthase/pyruvate dehydrogenase	Ofo, Pfor, PdhAB	OOR2r, PDH, POR2	Assigned based on 50% sequence identity with Moorella thermoacetica, 50% with M. barkeri in swissprot, 46% in PDB with Thermotoga maritima and GO annotation. For 2-oxoglutarate synthase, function assigned based on Based on 41% sequence identity with Thermoproteus tenax in swissprot, 39% with W. succinogens and GO annotation. For pyruvate dehydrogenase, function assigned based on EC equivalogs and 28% sequence identity with Syntrophus aciditrophicus SB, 27% sequence identity in PDB with archeon Pyrobaculum aerophilum.	3
30	cbdbA682	DET0727	DehaBAV1_0659	VS633	pyruvic-ferredoxin oxidoreductase, beta subunit	Pyruvate synthase/2-oxoglutarate synthase	Ofo, Pfor	OOR2r, POR2	Assigned based on 50% sequence identity with Moorella thermoacetica, 50% with M. barkeri in swissprot, 46% in PDB with Thermotoga maritima and GO annotation. For 2-oxoglutarate synthase, function assigned based on Based on 41% sequence identity with Thermoproteus tenax in swissprot, 39% with W. succinogens and GO annotation.	3
31	cbdbA689	DET0736	DehaBAV1_0666	VS642	oxidoreductase, short chaindehydrogenase	3-oxoacyl [acyl-carrier-protein] reductase	FabG1	HDER4, HBUR1, HTDR6, HDDR5, HICR9, HOCR3, HHYR2, HHDR7, HODR8_#1	Assigned based on 43% sequence identity with Desulfitobacterium hafniense in IMG-JGI, SWISSPROT, 38% in PDB and GO annotation.	3
32	cbdbA730	DET0758	DehaBAV1_0685	VS664	conserved hypothetical protein	succinyl-diaminopimelate desuccinylase	DapE	SDPDS	Assigned based on 37% sequence identity with Haemophilus influenzae PittHH in IMG-JGI and EC equivalogs.	2

No	CBDB1 gene locus	195 gene locus	BAV1 gene locus	VS gene locus	Primary annotation	Putative reannotation	Model protein	Model reaction	Reason for reannotation	Confidence level*
33	cbdbA762	DET0785	DehaBAV1_0711	VS691	metallo-beta-lactamase family protein	Hydroxyacylglutathione hydrolase	GloB	GLYOX	Assigned based on EC equivalogs (TIGR protein family), 29% sequence identity with E. coli, 33% with Bacillus cereus ATCC 14579, and 26% in PDB.	2
34	cbdbA802	DET0824	DehaBAV1_0743	VS728	probable nitroreductase family protein	Glucose 1-phosphate adenylyltransferase	GlgC	GLGC	Assigned based on 25% sequence identity with Thermus thermophilus HB27 in IMG-JGI and EC equivalogs (TIGR protein family) in KEGG pathways provided by IMG-JGI.	1
35	cbdbA841	DET0859	DehaBAV1_0777	VS762	GTP-binding protein LepA	Sulfate adenylyltransferase	CysH	SADT2	Assigned based on EC equivalogs (TIGR protein family) in KEGG pathways provided by IMG-JGI.	1
36	cbdbA849	DET0866	DehaBAV1_0784	VS769	putative hydrogenase subunit	Glutamate synthase	GltB	GLUSy	Assigned based on 28% sequence identity with G. sulfurreducens in IMG, JGI, 25% in PDB, 30% in SWISSPROT, GO annotation and synteny in IMG, JGI.	1
37	cbdbA894	DET0940	DehaBAV1_0824	VS811	acyl-CoA synthetase (AMP-forming) / AMP-acid ligase	Phenylacetate-CoA ligase	PaaK	PACCOAL	Assigned based on 30% sequence identity with G. sulfurreducens, 30% with E. coli in IMG-JGI, 37% with Desulfovibrio desulfuricans in SWISSPROT and GO annotation.	2

No	CBDB1 gene locus	195 gene locus	BAV1 gene locus	VS gene locus	Primary annotation	Putative reannotation	Model protein	Model reaction	Reason for reannotation	Confidence level*
38	cbdbA901	DET0946	DehaBAV1_0830	VS817	acyl-CoA synthetase (AMP-forming) / AMP-acid ligase	Long chain fatty acid-CoA ligase	FadD	FACOAL181, FACOAL140, FACOAL160, FACOAL120, FACOAL170(ISO), FACOAL180, FACOAL200	Assigned based on 25% sequence identity with Thermus thermophilus HB8 in PDB, with G. sulfurreducens and B. subtulis in IMG-JGI.	1
39	cbdbA942	DET0978	DehaBAV1_0869	VS851	glycosyl transferase, group 1 family protein	Glycogen synthase	GlgA1	GLCS1	Assigned based on 27% sequence identity in PDB, 28% with Thermoanaerobacter pseudethanolicus ATCC 33223 in IMG-JGI and GO annotation.	1
40	cbdbA963	DET1002	DehaBAV1_0890	VS874	glycosyl transferase, group 1 family protein	Glycogen synthase	GlgA1	GLCS1	Assigned based on 27% sequence identity in PDB, 28% with Thermoanaerobacter pseudethanolicus ATCC 33223 in IMG-JGI and GO annotation.	1
41	cbdbA988	DET1015	DehaBAV1_0902	VS886	acyl-CoA synthetase (AMP-forming) / AMP-acid ligase	Phenylacetate-CoA ligase	PaaK	PACCOAL	Assigned based on 30% sequence identity with G. sulfurreducens, 30% with E. coli in IMG-JGI, 37% with Desulfovibrio desulfuricans in SWISSPROT and GO annotation.	2

No	CBDB1 gene locus	195 gene locus	BAV1 gene locus	VS gene locus	Primary annotation	Putative reannotation	Model protein	Model reaction	Reason for reannotation	Confidence level*
42	cbdbA1006	DET1033	DehaBAV1_0916	VS904	acyl-CoA synthetase (AMP-forming) / AMP-acid ligase	Long chain fatty acid-CoA ligase	FadD	FACOAL181, FACOAL140, FACOAL160, FACOAL120, FACOAL170(ISO), FACOAL180, FACOAL200	Assigned based on 25% sequence identity with Thermus thermophilus HB8 in PDB, with G. sulfurreducens and B. subtulis in IMG-JGI.	1
43	cbdbA1008	DET1035	DehaBAV1_0917	VS905	pyridoxal-phosphate dependent TrpB-like enzyme	Tryptophan synthaes beta subunit	TrpB	TRPS1	Assigned based on 63% sequence identity with Methanospirillum hungatei JF-1 in IMG-JGI, 61% with Methanoculleus marisnigri strain ATCC 35101 in SWISSPROT, 33% in PDB and on GO annotation.	3
44	cbdbA1054	DET1127	DehaBAV1_0956	VS945	conserved hypothetical protein	Glutamate synthase small chain	GltB	GLUSy	Assigned based on 28% sequence identity with G. sulfurreducens in IMG, JGI, 25% in PDB, 30% in SWISSPROT, GO annotation and synteny in IMG, JGI.	1
45	cbdbA1058	DET1130	DehaBAV1_0959	VS948	glutamine amidotransferase, class II	Glutamate synthase small chain	GltB	GLUSy	Assigned based on 28% sequence identity with G. sulfurreducens in IMG, JGI, 25% in PDB, 30% in SWISSPROT, GO annotation and synteny in IMG, JGI.	1
46	cbdbA1059	DET1131	DehaBAV1_0960	VS949	pyridine nucleotide-disulfide oxidoreductase family protein	Nitrite reductase	NasDE	NTRIRy, NTRIRx	Assigned based on 30% sequence identity with Bacillus halodurans C-125 in IMG-JGI and SWISSPROT, 33% in PDB, and GO annotation.	2

No	CBDB1 gene locus	195 gene locus	BAV1 gene locus	VS gene locus	Primary annotation	Putative reannotation	Model protein	Model reaction	Reason for reannotation	Confidence level*
47	cbdbA1061	DET1133	DehaBAV1_0962	VS951	iron-sulfur cluster-binding protein	Glutamate synthase small chain	GltB	GLUSy	Assigned based on 33% sequence identity with E.coli ATC 8739 in IMG-JGI and SWISSPROT.	2
48	cbdbA1070	DET1140	DehaBAV1_0969	VS958	carbon-nitrogen hydrolase family protein	NAD synthase	NadE	NADS1	Assigned based on 30% sequence identity with Methylobacillus flagellatus strain KT in SWISSPROT, 23% with Psychrobacter arcticus 273-4 in IMG-JGI and GO annotation.	1
49	cbdbA1077	0	DehaBAV1_0975	No gene	GCN5-related N-acetyltransferase	Succinyl-CoA synthetase/long chain fatty acid-CoA ligase	FadD, SucCD1	SUCOAS, FACOAL160, FACOAL200, FACOAL181, FACOAL170(ISO), FACOAL140, FACOAL180, FACOAL120	Assigned based on 35% sequence identity with Thermoplasma volcanium GSS1, evidence in Pfam and InterPro databases as well as EC equivologs found in KEGG pathways provided by IMG, JGI.	2
50	cbdbA1098	DET1184	DehaBAV1_0995	VS967	BioY family protein	Biotin synthase	BioBec	BTS	Assigned based on 35% sequence identity with Methanosarcina barkeri fusaro in IMG-JGI and SWISSPROT and EC equivalogs (TIGR protein family) in KEGG pathways provided by IMG-JGI.	2
51	cbdbA1111	DET1196	DehaBAV1_1007	VS979	ATP-dependent DNA helicase PcrA	Dihydroneopterin triphosphate pyrophophorylase/UDP-N-acetylglucosamine 1-carboxyvinyltransferase	NtpA, MurAA	DNMPPA, UAGCVT, DNTPPA	Assigned based on EC equivalogs and 33% sequence identity with Campylobacter concisus 13826.	2

No	CBDB1 gene locus	195 gene locus	BAV1 gene locus	VS gene locus	Primary annotation	Putative reannotation	Model protein	Model reaction	Reason for reannotation	Confidence level*
52	cbdbA1112	DET1198	DehaBAV1_1008	VS981	pyrimidine operon regulatory protein	Uracil phosphoribosyltransferase	Upp2	UPPRT	Assigned based on 61% sequence identity with Moorella thermoacetica in IMG-JGI, SWISSPROT and 56% in PDB.	3
53	cbdbA1125	DET1208	DehaBAV1_1018	VS991	nucleotidyl transferase family protein	Mannose-1-phosphate guanylyltransferase/Glucose-1-phosphate adenylyltransferase	Mpg, GlgC	MAN1PT1, GLGC	Assigned based on 34% sequence identity with Geobacter sulfurreducens in IMG-JGI, SWISSPROT and GO annotation.	2
54	cbdbA1136	DET1218	DehaBAV1_1028	VS1001	HAD-superfamily hydrolase, subfamily IIB	Phosphoserine phosphatase	SerB	PSP_L	Assigned based on 50% sequence identity with Pelobacter carbinolicus DSM 2380 in IMG-JGI and 46% sequence identity in PDB.	3
55	cbdbA1140	DET1223	DehaBAV1_1032	VS1005	fumarylacetoacetate hydrolase family protein	Acylpyruvate hydrolase	P_3.7.1.5	APYRH	Assigned based on 37% sequence identity with Geobacter sulfurreducens, 33% with E.coli in IMG-JGI and 34% sequence identity in PDB.	2
56	cbdbA1142	DET1225	DehaBAV1_1034	VS1007	conserved hypothetical protein	Fructose-1,6-bisphosphatase	Fbp	FBP	Assigned based on 68% sequence identity with Moorella thermoacetica in IMG-JGI, SWISSPROT, 60% in PDB and GO annotation.	3
57	cbdbA1143	DET1226	DehaBAV1_1035	VS1008	non-canonical purine NTP pyrophosphatase, RdgB	dITP/XTP pyrophosphatase	IxpP	XTPASE, ITPASE	Assigned based on 47% sequence identity with Escherichia coli K12 in IMG-JGI, SWISSPROT and 44% in PDB.	3
58	cbdbA1194	DET1268	DehaBAV1_1079	VS1051	ATP-dependent DNA helicase RecG	Dihydroneopterin triphosphate pyrophophorylase	NtpA	DNMPPA, DNTPPA	Assigned based on EC equivalogs and 33% sequence identity with Campylobacter concisus 13826.	2
59	cbdbA1237	DET1298	DehaBAV1_1109	VS1081	GTP-binding protein TypA	Sulfate adenylyltransferase	CysH	SADT2	Assigned based on EC equivalogs (TIGR protein family) in KEGG pathways provided by IMG-JGI.	1

No	CBDB1 gene locus	195 gene locus	BAV1 gene locus	VS gene locus	Primary annotation	Putative reannotation	Model protein	Model reaction	Reason for reannotation	Confidence level*
60	cbdbA1238	DET1299	DehaBAV1_1110	VS1082	major facilitator family transporter	Proline/betaine transporter	ProPec	GLYBt6, PROt6	Assigned based on 32% sequence identity with E. coli in Transport Classification database (TCDB).	2
61	cbdbA1269	DET1324	DehaBAV1_1135	VS1106	conserved hypothetical protein	UDP-glucose-4-epimerase	GalE	UDPG4E	Assigned based on EC equivalogs (TIGR protein family) in IMG-JGI, 27% sequence identity with E. coli in IMG-JGI and 25% sequence identity in PDB.	1
62	cbdbA1338	No gene	DehaBAV1_1193	VS1164	L-aspartate oxidase(EC:1.4.3.16)	L-aspartate oxidase(EC:1.4.3.16)/succinate dehydrogenase	Frd, NadB	FRD5, ASPO1, ASPO3, ASPO4, ASPO5, ASPO6	Assigned based on 35% sequence identity with Geobacter metallireducens, 25% identity with Geobacter sulfurreducens and 40% identity in PDB.	2
63	cbdbA1346	DET1390	DehaBAV1_1199	VS1171	PAP2 family protein	Phosphotidylglycerophosphate phosphohydrolase	PgpA, Pmdpht	PMDPHT, PGPPH	Assigned based on 29% sequence identity with Bacillus cereus E33L in IMG-JGI SWISSPROT, 32% sequence identity in PDB and strong interaction in string database.	1
64	cbdbA1359	DET1400	DehaBAV1_1209	VS1182	co-chaperone protein GrpE	Ribonucleoside-triphosphate reductase	NrdD	RNTR2, RNTR3, RNTR1, RNTR4	Assigned based on EC equivalogs, 23% sequence identity with Lactobacillus acidophilus NCFM in IMG-JGI, SWISSPROT and 26% sequence identity in PDB.	1
65	cbdbA1527	DET1510	0	No gene	conserved hypothetical protein	Phosphomethylpyrimidine kinase	ThiD	PMPK	Assigned based on EC equivalogs and 33% sequence identity with Campylobacter concisus 13826 in IMG-JGI and SWISSPROT.	2
66	cbdbA1557	DET0089	No gene	No gene	radical SAM domain protein	Pyruvate formate lyase	PflC	PFLi	Assigned based on 29% sequence identity with Methanopyrus kandleri AV19, 35% sequence identity with Vibrio harveyi HY01 in IMG-JGI, SWISSPROT and 25% in PDB.	2

No	CBDB1 gene locus	195 gene locus	BAV1 gene locus	VS gene locus	Primary annotation	Putative reannotation	Model protein	Model reaction	Reason for reannotation	Confidence level*
67	cbdbA1667	No gene	DehaBAV1_1328	VS1458	putative dihydroflavonol 4-reductase	UDP-glucose-4-epimerase	GalE	UDPG4E	Assigned based on EC equivalogs (TIGR protein family) in IMG-JGI, 27% sequence identity with E. coli in IMG-JGI and 25% sequence identity in PDB.	1
68	cbdbA1668	No gene	DehaBAV1_1329	VS1459	hypothetical protein	UDP-glucose-4-epimerase	GalE	UDPG4E	Assigned based on EC equivalogs (TIGR protein family) in IMG-JGI, 27% sequence identity with E. coli in IMG-JGI and 25% sequence identity in PDB.	1
69	cbdbA1735	DET1637	DehaBAV1_1381	VS1519	mazG family protein	Nucleotide triphosphate pyrophosphohydrolase	MazG	NTPP5, NTPP2, NTPP4, NTPP6, NTPP8, NTPP1, NTPP7, NTPP3	Assigned based on 42% sequence identity in PDB, 47% with Moorella thermoacetica in IMG-JGI and SWISSPROT.	3
70	cbdbA1741	DET1641	DehaBAV1_1385	VS1523	aldolase, class II	L-fuculose phosphate aldolase	FucA	FCLPA	Assigned based on 38% sequence identity with Thermus thermophilus HB8 in PDB, 44% with Methanosarcina mazei Go1 in IMG-JGI and SWISSPROT.	3

* >40% amino acid sequence identity =confidence level 3, >30% identity = confidence level 2, <30% identity = confidence level 1

Table 3. Gene Correspondence for Core Genes Mapped to Reactions of *i* AI549

Model gene number	CBDB1 core metabolic genes	195 core metabolic genes	BAV1 core metabolic genes	VS core metabolic genes	CBDB1 annotation	195 annotation	BAV1 annotation	VS annotation	Model protein	Model reaction
1	cbdbA3	DET0003	DehaBAV1_0003	VS3	nicotinate (nicotinamide) nucleotideadenylyltransferase	nicotinate (nicotinamide) nucleotide adenylyltransferase	nicotinate (nicotinamide) nucleotide adenylyltransferase(EC:3.5.4.4)	nicotinate (nicotinamide) nucleotide adenylyltransferase (EC:2.7.7.18)	NadD	NNATr, NMNATr
2	cbdbA5	DET0005	DehaBAV1_0005	VS5	GTP pyrophosphokinase	GTP pyrophosphokinase(EC:2.7.6.5)	(p)ppGpp synthetase I, SpoT/RelA(EC:2.7.6.5)	(p)ppGpp synthetase I, SpoT/RelA(EC:2.7.6.5)	RelA	GTPDK
3	cbdbA7	DET0006	DehaBAV1_0006	VS6	histidyl-tRNA synthetase	histidyl-tRNA synthetase (EC:6.1.1.21)	histidyl-tRNA synthetase(EC:6.1.1.21)	histidyl-tRNA synthetase(EC:6.1.1.21)	HisS	HISTRS
4	cbdbA9	DET0007	DehaBAV1_0007	VS7	hypothetical protein	hypothetical protein	Saccharopine dehydrogenase	Saccharopine dehydrogenase	Lys1	SACCD2
5	cbdbA11	DET0009	DehaBAV1_0009	VS9	branched-chain amino acid aminotransferase	branched-chain amino acid aminotransferase(EC:2.6.1.42)	branched-chain amino acid aminotransferase	branched-chain amino acid aminotransferase	IlvE	VALTA, LEUTA, ILETA
6	cbdbA14	DET0013	DehaBAV1_0012	VS12	orotidine 5-phosphate decarboxylase	orotidine 5'-phosphate decarboxylase(EC:4.1.1.23)	orotidine 5'-phosphate decarboxylase	orotidine 5'-phosphate decarboxylase	PyrF	OMPDC
7	cbdbA18	DET0016	DehaBAV1_0015	VS15	putative folylpolyglutamate synthetase	folylpolyglutamate synthetase(EC:6.3.2.17)	FolC bifunctional protein	FolC bifunctional protein	MetA1, FolC	HSAT, DHFS
8	cbdbA41	DET0034	DehaBAV1_0031	VS32	putative phosphate transporter	phosphate transporter	phosphate transporter	phosphate transporter	PitA	PIt6
9	cbdbA42	DET0035	DehaBAV1_0032	VS33	putative guanylate kinase	guanylate kinase(EC:2.7.4.8)	Guanylate kinase(EC:2.7.4.8)	Guanylate kinase(EC:2.7.4.8)	Gmk	GK1, DGK1
10	cbdbA45	DET0037	DehaBAV1_0034	VS35	PyrK family protein	PyrK family protein	oxidoreductase FAD/NAD(P)-binding domain protein	oxidoreductase FAD/NAD(P)-binding domain protein	PyrK	DHORDf

Model gene number	CBDB1 core metabolic genes	195 core metabolic genes	BAV1 core metabolic genes	VS core metabolic genes	CBDB1 annotation	195 annotation	BAV1 annotation	VS annotation	Model protein	Model reaction
11	cbdbA46	DET0038	DehaBAV1_0035	VS36	glutamate synthase (NADPH), homotetrameric	glutamate synthase (NADPH), homotetrameric(EC:1.4.1.13)	glutamate synthase (NADPH), homotetrameric	glutamate synthase (NADPH), homotetrameric	GltBD, P_1.18.1.2	GLUSy, FNOR2
12	cbdbA52	DET0041	DehaBAV1_0038	VS41	SAM-dependent methyltransferase UbiE/COQ5 family	quinone methlytransferase, UbiE family	Methyltransferase type 11	Methyltransferase type 11	UbiE	OMBZLM
13	cbdbA63	DET0049	DehaBAV1_0046	VS49	transcriptional repressor, ROK family	ROK family protein	ROK family protein	ROK family protein	GlcK	HEX1
14	cbdbA65	DET0050	DehaBAV1_0047	VS50	alanyl-tRNA synthetase	alanyl-tRNA synthetase(EC:6.1.1.7)	alanyl-tRNA synthetase(EC:6.1.1.7)	alanyl-tRNA synthetase(EC:6.1.1.7)	AlaS	ALATRS
15	cbdbA69	DET0053	DehaBAV1_0050	VS53	alcohol dehydrogenase, iron-containing	alcohol dehydrogenase, iron-containing	iron-containing alcohol dehydrogenase	iron-containing alcohol dehydrogenase	AdhC	ALCD19, ALCD2x, ALCD3
16	cbdbA74	DET0059	DehaBAV1_0053	VS59	2-C-methyl-D-erythritol 4-phosphatecytidylyltransferase	2-C-methyl-D-erythritol 4-phosphate cytidylyltransferase(EC:2.7.7.60)	2-C-methyl-D-erythritol 4-phosphate cytidylyltransferase	2-C-methyl-D-erythritol 4-phosphate cytidylyltransferase	IspD	MEPCT
17	cbdbA75	DET0060	DehaBAV1_0054	VS60	2-C-methyl-D-erythritol 2,4-cyclodiphosphate synthase	2C-methyl-D-erythritol 2,4-cyclodiphosphate synthase(EC:4.6.1.12)	2C-methyl-D-erythritol 2,4-cyclodiphosphate synthase(EC:4.6.1.12)	2C-methyl-D-erythritol 2,4-cyclodiphosphate synthase(EC:4.6.1.12)	IspF	MECDPS
18	cbdbA76	DET0061	DehaBAV1_0055	VS61	cysteinyl-tRNA synthetase	cysteinyl-tRNA synthetase(EC:6.1.1.16)	cysteinyl-tRNA synthetase(EC:6.1.1.16)	cysteinyl-tRNA synthetase(EC:6.1.1.16)	CysS	CYSTRS
19	cbdbA96	DET0318	DehaBAV1_0112	VS1387	putative reductive dehalogenase	reductive dehalogenase, putative	reductive dehalogenase	reductive dehalogenase	RdhA	RDR1, RDR2
20	cbdbA128	DET0109	DehaBAV1_0259	VS119	putative Ni/Fe hydrogenase maturation protease	hydrogenase maturation protease	hydrogenase maturation protease	hydrogenase maturation protease	FrdH	FRHD

Model gene number	CBDB1 core metabolic genes	195 core metabolic genes	BAV1 core metabolic genes	VS core metabolic genes	CBDB1 annotation	195 annotation	BAV1 annotation	VS annotation	Model protein	Model reaction
21	cbdbA129	DET0110	DehaBAV1_0258	VS120	Ni/Fe hydrogenase large subunit	[Ni/Fe] hydrogenase, group 1, large subunit, putative	nickel-dependent hydrogenase, large subunit	nickel-dependent hydrogenase, large subunit	FrdH	FRHD
22	cbdbA130	DET0111	DehaBAV1_0257	VS121	Ni/Fe hydrogenase small subunit	[Ni/Fe] hydrogenase, group 1, small subunit, putative	hydrogenase (NiFe) small subunit HydA(EC:1.12.99.6)	hydrogenase (NiFe) small subunit HydA(EC:1.12.99.6)	FrdH	FRHD
23	cbdbA131	DET0112	DehaBAV1_0256	VS122	putative Ni/Fe hydrogenase, iron-sulfur cluster-binding subunit	[Ni/Fe] hydrogenase, iron-sulfur cluster-binding subunit, putative	4Fe-4S ferredoxin, iron sulfur binding domain protein	4Fe-4S ferredoxin, iron-sulfur binding domain protein	FrdH	FRHD
24	cbdbA139	DET0119	DehaBAV1_0250	VS128	oxaloacetate decarboxylase, alpha subunit	oxaloacetate decarboxylase(EC:4.1.1.3)	oxaloacetate decarboxylase alpha subunit	oxaloacetate decarboxylase alpha subunit	AccC, Pyc, OadD	OXAD2, ACCOAC, PC
25	cbdbA141	DET0120	DehaBAV1_0249	VS129	acetyl-CoA carboxylase, biotin carboxylase	acetyl-CoA carboxylase(EC:6.4.1.1,EC:6.4.1.2)	acetyl-CoA carboxylase, biotin carboxylase(EC:6.4.1.1)	acetyl-CoA carboxylase, biotin carboxylase(EC:6.4.1.1)	AccC, Pyc	ACCOAC, PC
26	cbdbA143	DET0123	DehaBAV1_0247	VS131	pyridine nucleotide-disulphide oxidoreductase family protein	pyridine nucleotide-disulphide oxidoreductase family protein	4Fe-4S ferredoxin, iron sulfur binding domain protein	4Fe-4S ferredoxin, iron-sulfur binding domain protein	GltB	GLUSy
27	cbdbA145	DET0125	DehaBAV1_0246	VS132	alcohol dehydrogenase, zinc-containing	alcohol dehydrogenase, zinc-containing	Alcohol dehydrogenase GroES domain protein	Alcohol dehydrogenase GroES domain protein	AdhC	ALCD19, ALCD2x, ALCD3
28	cbdbA146	DET0126	DehaBAV1_0245	VS133	anthranilate phosphoribosyltransferase	anthranilate phosphoribosyltransferase(EC:2.4.2.18)	anthranilate phosphoribosyltransferase	anthranilate phosphoribosyltransferase(EC:2.4.2.18)	TrpD	ANPRT
29	cbdbA149	DET0128	DehaBAV1_0243	VS135	cobyrinic acid a,c-diamide synthase	cobyrinic acid a,c-diamide synthase	cobyrinic acid a,c-diamide synthase	cobyrinic acid a,c-diamide synthase	CobB	CYRDAS
30	cbdbA160	DET0137	DehaBAV1_0233	VS145	methylglyoxal synthase	methylglyoxal synthase(EC:4.2.3.3)	methylglyoxal synthase(EC:4.2.3.3)	methylglyoxal synthase(EC:4.2.3.3)	MgsA	MGSA

Model gene number	CBDB1 core metabolic genes	195 core metabolic genes	BAV1 core metabolic genes	VS core metabolic genes	CBDB1 annotation	195 annotation	BAV1 annotation	VS annotation	Model protein	Model reaction
31	cbdbA161	DET0138	DehaBAV1_0232	VS146	phosphate ABC transporter, phosphate-binding protein	phosphate ABC transporter, phosphate-binding protein	phosphate binding protein	phosphate binding protein	PstABCS	PIabc
32	cbdbA162	DET0139	DehaBAV1_0231	VS147	phosphate ABC transporter, permease protein	phosphate ABC transporter, permease protein	phosphate ABC transporter, inner membrane subunit PstC	phosphate ABC transporter, inner membrane subunit PstC	PstABCS	PIabc
33	cbdbA164	DET0140	DehaBAV1_0230	VS148	phosphate ABC transporter, permease protein	phosphate ABC transporter, permease protein	phosphate ABC transporter, inner membrane subunit PstA	phosphate ABC transporter, inner membrane subunit PstA	PstABCS	PIabc
34	cbdbA165	DET0141	DehaBAV1_0229	VS149	phosphate ABC transporter, ATP-binding protein	phosphate ABC transporter, ATP-binding protein(EC:3.6.3.27)	phosphate ABC transporter, ATPase subunit(EC:3.6.3.27)	phosphate ABC transporter, ATPase subunit(EC:3.6.3.27)	PstABCS	PIabc
35	cbdbA169	DET0145	DehaBAV1_0226	VS153	putative [Fe] hydrogenase, HymA subunit	[Fe] hydrogenase, HymA subunit, putative	NADH dehydrogenase (ubiquinone), 24 kDa subunit	NADH dehydrogenase (ubiquinone), 24 kDa subunit	Nadhd5	NADH6, NADH7, NADH4, NADH8
36	cbdbA170	DET0146	DehaBAV1_0225	VS154	putative [Fe] hydrogenase, HymB subunit	[Fe] hydrogenase, HymB subunit, putative(EC:1.6.99.5)	NADH dehydrogenase (quinone)(EC:1.6.99.5)	NADH dehydrogenase (quinone)(EC:1.6.99.5)	Nadhd5	NADH6, NADH7, NADH4, NADH8
37	cbdbA171	DET0147	DehaBAV1_0224	VS155	putative [Fe] hydrogenase, large subunit HymC	[Fe] hydrogenase, large subunit HymC, putative	hydrogenase, Fe-only	hydrogenase, Fe-only	Nadphd, Nadhd5, Fdx	NADH6, NADPH12, NADH7, NADH4, FRHD, NADH8
38	cbdbA173	DET0148	DehaBAV1_0223	VS156	putative [Fe] hydrogenase, HymD subunit	[Fe] hydrogenase, HymD subunit, putative	hypothetical protein	putative (Fe) hydrogenase, HymD subunit	Nadhd5	NADH6, NADH7, NADH4, NADH8
39	cbdbA182	DET0153	DehaBAV1_0217	VS162	hypothetical protein	hypothetical protein	hypothetical protein	alkylhydroperoxidase like protein, AhpD family	PcaC2	CMLDC

Model gene number	CBDB1 core metabolic genes	195 core metabolic genes	BAV1 core metabolic genes	VS core metabolic genes	CBDB1 annotation	195 annotation	BAV1 annotation	VS annotation	Model protein	Model reaction
40	cbdbA187	DET0180	DehaBAV1_0173	VS169	putative reductive dehalogenase	reductive dehalogenase, putative	reductive dehalogenase	reductive dehalogenase	RdhA	RDR1, RDR2
41	cbdbA188	DET0181	DehaBAV1_0172	VS170	putative reductive dehalogenase anchoring protein	reductive dehalogenase anchoring protein, putative	hypothetical protein	reductive dehalogenase anchoring protein, putative	RdhB	RDR1, RDR2
42	cbdbA196	DET0189	DehaBAV1_0164	VS177	putative methyltransferase	methyltransferase, putative	putative methyltransferase	putative methyltransferase	AsnA	ASNS2
43	cbdbA197	DET0190	DehaBAV1_0163	VS178	pantetheine-phosphate adenylyltransferase	pantetheine-phosphate adenylyltransferase(EC:2.7.7.3)	pantetheine-phosphate adenylyltransferase(EC:2.7.7.3)	pantetheine-phosphate adenylyltransferase (EC:2.7.7.3)	CoaD	PTPAT
44	cbdbA198	DET0191	DehaBAV1_0162	VS179	ferredoxin, iron-sulfur cluster-binding protein	iron-sulfur cluster-binding protein	4Fe-4S ferredoxin, iron sulfur binding domain protein	4Fe-4S ferredoxin, iron-sulfur binding domain protein	Fdx	FRHD
45	cbdbA200	DET0193	DehaBAV1_0160	VS181	pyrroline-5-carboxylate reductase	pyrroline-5-carboxylate reductase(EC:1.5.1.2)	pyrroline-5-carboxylate reductase(EC:1.5.1.2)	pyrroline-5-carboxylate reductase(EC:1.5.1.2)	ProC	P5CR
46	cbdbA202	DET0194	DehaBAV1_0159	VS182	leucyl-tRNA synthetase	leucyl-tRNA synthetase(EC:6.1.1.4)	leucyl-tRNA synthetase	leucyl-tRNA synthetase	LeuS	LEUTRS
47	cbdbA208	DET0199	DehaBAV1_0154	VS187	putative ferredoxin-thioredoxin reductase,catalyticsubunit	ferredoxin-thioredoxin reductase, catalytic subunit, putative/rubredoxin	Rubredoxin-type Fe(Cys)4 protein	putative ferredoxin-thioredoxin reductase,catalyticsubunit	Fdx	FRHD
48	cbdbA219	DET0221	DehaBAV1_0133	VS197	L-lysine 2,3-aminomutase homologe, probable frameshift	L-lysine 2,3-aminomutase, putative/acetyltransferase, GNAT family	lysine 2,3-aminomutase YodO family protein(EC:5.4.3.2)	lysine 2,3-aminomutase YodO family protein	KamA	LYSAM
49	cbdbA238	DET0302	DehaBAV1_0121	VS1353	putative reductive dehalogenase	reductive dehalogenase, putative	reductive dehalogenase	reductive dehalogenase	RdhA	RDR1, RDR2

Model gene number	CBDB1 core metabolic genes	195 core metabolic genes	BAV1 core metabolic genes	VS core metabolic genes	CBDB1 annotation	195 annotation	BAV1 annotation	VS annotation	Model protein	Model reaction
50	cbdbA239	DET0303	DehaBAV1_ 0120	VS1352	putative reductive dehalogenase anchoring protein	reductive dehalogenase anchoring protein, putative	hypothetical protein	putative reductive dehalogenase anchoring protein	RdhB	RDR1, RDR2
51	cbdbA243	DET0235	DehaBAV1_ 0119	VS1347	putative reductive dehalogenase	reductive dehalogenase, putative	reductive dehalogenase	reductive dehalogenase	RdhA	RDR1, RDR2
52	cbdbA287	DET0345	DehaBAV1_ 0324	VS286	ribonucleotide reductase	ribonucleotide-diphosphate reductase alpha subunit(EC:1.17.4.1)	ribonucleoside-diphosphate reductase, adenosylcobalamin-dependent(EC:1.17.4.1)	ribonucleoside-diphosphate reductase, adenosylcobalamin-dependent	NrdD, NrdEF	RNDR3, RNDR1, RNDR4, RNDR2, RNTR2, RNTR3, RNTR1, RNTR4
53	cbdbA307	DET0365	DehaBAV1_ 0347	VS309	phenylalanyl-tRNA synthetase, alpha subunit	phenylalanyl-tRNA synthetase, alpha subunit(EC:6.1.1.20)	phenylalanyl-tRNA synthetase, alpha subunit	phenylalanyl-tRNA synthetase, alpha subunit(EC:6.1.1.20)	PheRS	PHETRS
54	cbdbA308	DET0366	DehaBAV1_ 0348	VS310	phenylalanyl-tRNA synthetase, beta subunit	phenylalanyl-tRNA synthetase, beta subunit(EC:6.1.1.20)	phenylalanyl-tRNA synthetase, beta subunit(EC:6.1.1.20)	phenylalanyl-tRNA synthetase, beta subunit(EC:6.1.1.20)	PheRS	PHETRS
55	cbdbA309	DET0367	DehaBAV1_ 0349	VS311	inorganic pyrophosphatase	inorganic pyrophosphatase(EC:3.6.1.1)	Inorganic diphosphatase(EC:3.6.1.1)	Inorganic diphosphatase(EC:3.6.1.1)	Ppa	PPA
56	cbdbA310	DET0368	DehaBAV1_ 0350	VS312	prolyl-tRNA synthetase	prolyl-tRNA synthetase(EC:6.1.1.15)	prolyl-tRNA synthetase	prolyl-tRNA synthetase	ProS	PROTRS
57	cbdbA311	DET0369	DehaBAV1_ 0351	VS313	1-hydroxy-2-methyl-2-(E)-butenyl 4-diphosphatesynthase	1-hydroxy-2-methyl-2-(E)-butenyl 4-diphosphate synthase(EC:1.17.4.3)	1-hydroxy-2-methyl-2-(E)-butenyl 4-diphosphate synthase(EC:1.17.4.3)	1-hydroxy-2-methyl-2-(E)-butenyl 4-diphosphate synthase(EC:1.17.4.3)	IspG	MECDPDH

Model gene number	CBDB1 core metabolic genes	195 core metabolic genes	BAV1 core metabolic genes	VS core metabolic genes	CBDB1 annotation	195 annotation	BAV1 annotation	VS annotation	Model protein	Model reaction
58	cbdbA314	DET0371	DehaBAV1_0353	VS315	1-deoxy-D-xylulose 5-phosphate reductoisomerase	1-deoxy-D-xylulose 5-phosphate reductoisomerase(EC:1.1.1.267)	1-deoxy-D-xylulose 5-phosphate reductoisomerase(EC:1.1.1.267)	1-deoxy-D-xylulose 5-phosphate reductoisomerase(EC:1.1.1.267)	Dxr	DXPRI
59	cbdbA315	DET0372	DehaBAV1_0354	VS316	phosphatidate cytidylyltransferase	phosphatidate cytidylyltransferase(EC:2.7.7.41)	phosphatidate cytidylyltransferase	phosphatidate cytidylyltransferase	CdsA	PHCYT
60	cbdbA316	DET0373	DehaBAV1_0355	VS317	undecaprenyl diphosphate synthase	undecaprenyl diphosphate synthase(EC:2.5.1.31)	undecaprenyl diphosphate synthase(EC:2.5.1.31)	undecaprenyl diphosphate synthase(EC:2.5.1.31)	UppS	UDPDPS
61	cbdbA319	DET0375	DehaBAV1_0357	VS319	uridylate kinase	uridylate kinase(EC:2.7.4.-)	uridylate kinase	uridylate kinase	PyrH	URIDK2, URIDK1
62	cbdbA323	DET0378	DehaBAV1_0360	VS322	phosphoribosylformylglycinamidine synthase I	phosphoribosylformylglycinamidine synthase I(EC:6.3.5.3)	phosphoribosylformylglycinamidine synthase I(EC:6.3.5.3)	phosphoribosylformylglycinamidine synthase I	PurQ	PRFGS
63	cbdbA325	DET0379	DehaBAV1_0361	VS323	phosphoribosylformylglycinamidine synthase II	phosphoribosylformylglycinamidine synthase II(EC:6.3.5.3)	phosphoribosylformylglycinamidine synthase II(EC:6.3.5.3)	phosphoribosylformylglycinamidine synthase II(EC:6.3.5.3)	PurL	PRFGS
64	cbdbA327	DET0380	DehaBAV1_0362	VS324	pyridoxine biosynthesis protein	pyridoxine biosynthesis protein	pyridoxine biosynthesis protein	pyridoxine biosynthesis protein	PdxAJ	PDX5PS
65	cbdbA329	DET0384	DehaBAV1_0365	VS328	IMP dehydrogenase family protein	inositol-5-monophosphate dehydrogenase	IMP dehydrogenase family protein	IMP dehydrogenase family protein	GuaB, GuaC	GMPR, IMPD
66	cbdbA334	DET0388	DehaBAV1_0368	VS331	methionyl-tRNA synthetase	methionyl-tRNA synthetase(EC:6.1.1.10)	methionyl-tRNA synthetase	methionyl-tRNA synthetase	MetG	METTRS
67	cbdbA335	DET0389	DehaBAV1_0369	VS332	heptaprenyl diphosphate synthase component II	heptaprenyl diphosphate synthase component II(EC:2.5.1.30)	Polyprenyl synthetase	Trans-hexaprenyltranstransferase(EC:2.5.1.30)	IspA, HepT	PPTT, FRTT, GGTT, DMATT, HEPTT, OCTT, HEXTT, GRTT

Model gene number	CBDB1 core metabolic genes	195 core metabolic genes	BAV1 core metabolic genes	VS core metabolic genes	CBDB1 annotation	195 annotation	BAV1 annotation	VS annotation	Model protein	Model reaction
68	cbdbA343	DET0394	DehaBAV1_ 0373	VS336	nucleoside diphosphate kinase	nucleoside diphosphate kinase(EC:2.7.4.6)	Nucleoside-diphosphate kinase(EC:2.7.4.6)	Nucleoside-diphosphate kinase(EC:2.7.4.6)	Ndk	NDPK7, NDPK3, NDPK5, NDPK2, NDPK8, NDPK6, NDPK4, NDPK1
69	cbdbA347	DET0397	DehaBAV1_ 0376	VS340	thiamine-monophosphate kinase	thiamine-monophosphate kinase(EC:2.7.4.16)	thiamine-monophosphate kinase	thiamine-monophosphate kinase(EC:2.7.4.16)	ThiL	TMPKr
70	cbdbA349	DET0398	DehaBAV1_ 0377	VS341	putative ubiquinone/menaquinone biosynthesis methlytransferase UbiE	ubiquinone/menaquinone biosynthesis methlytransferase UbiE, putative	UbiE/COQ5 methyltransferase	UbiE/COQ5 methyltransferase	UbiE	OMBZLM
71	cbdbA350	DET0399	DehaBAV1_ 0378	VS342	putative ubiquinone/menaquinone biosynthesis methlytransferase UbiE	ubiquinone/menaquinone biosynthesis methlytransferase UbiE, putative	Methyltransferase type 11	Methyltransferase type 12	UbiE	OMBZLM
72	cbdbA351	DET0400	DehaBAV1_ 0379	VS343	3-dehydroquinate synthase	3-dehydroquinate synthase family protein(EC:4.2.3.-)	3-dehydroquinate synthase	3-dehydroquinate synthase	AroB	DHQS
73	cbdbA352	DET0401	DehaBAV1_ 0380	VS344	putative 1,4-dihydroxy-2-naphthoateoctaprenyltransferase	1,4-dihydroxy-2-naphthoate octaprenyltransferase, putative	UbiA prenyltransferase	UbiA prenyltransferase	MenA	DHNAOT
74	cbdbA356	DET0405	DehaBAV1_ 0384	VS348	4-diphosphocytidyl-2C methyl-D-erythritolkinase	4-diphosphocytidyl-2C-methyl-D-erythritol kinase(EC:2.7.1.148)	4-diphosphocytidyl-2C-methyl-D-erythritol kinase	4-diphosphocytidyl-2C-methyl-D-erythritol kinase	IspE, ThrB	CDPMEK, HSK
75	cbdbA359	DET0407	DehaBAV1_ 0386	VS350	conserved domain protein	hypothetical protein	hypothetical protein	conserved hypothetical protein	ManC	MAN1PT2

Model gene number	CBDB1 core metabolic genes	195 core metabolic genes	BAV1 core metabolic genes	VS core metabolic genes	CBDB1 annotation	195 annotation	BAV1 annotation	VS annotation	Model protein	Model reaction
76	cbdbA365	DET0413	DehaBAV1_0392	VS356	putative deoxycytidylate deaminase	cytidine/deoxycytidylate deaminase family protein	CMP/dCMP deaminase, zinc-binding	CMP/dCMP deaminase, zinc-binding	ComEB	DCMPDA3ir, DCMPDA2ir
77	cbdbA367	DET0414	DehaBAV1_0393	VS357	conserved hypothetical protein	conserved hypothetical protein TIGR00147	diacylglycerol kinase, catalytic region	diacylglycerol kinase, catalytic region	DgkA	DAGK
78	cbdbA368	DET0415	DehaBAV1_0394	VS358	deoxyuridine 5-triphosphatenucleotidohydrolase	putative deoxyuridine 5'-triphosphate nucleotidohydrolase(EC:3.6.1.23)	Deoxycytidine deaminase-like protein	putative deoxyuridine 5'-triphosphate nucleotidohydrolase	Dutec	DUTPDP
79	cbdbA369	DET0417	DehaBAV1_0396	VS360	amino acid ABC transporter, ATP-binding protein	amino acid ABC transporter, ATP-binding protein	ABC transporter related	ABC transporter related	GlnQ	GLNabc
80	cbdbA370	DET0418	DehaBAV1_0397	VS361	amino acid ABC transporter, permease protein	amino acid ABC transporter, permease protein, His/Glu/Gln/Arg/opine family	polar amino acid ABC transporter, inner membrane subunit	polar amino acid ABC transporter, inner membrane subunit	GlnP	GLNabc
81	cbdbA371	DET0419	DehaBAV1_0398	VS362	amino acid ABC transporter, periplasmic aminoacid-binding protein	amino acid ABC transporter, periplasmic amino acid-binding protein	extracellular solute-binding protein, family 3	extracellular solute-binding protein, family 3	GlnH	GLNabc
82	cbdbA374	DET0424	DehaBAV1_0400	VS365	putative magnesium and cobalt transport protein	magnesium and cobalt transport protein, putative	Mg2+ transporter protein, CorA family protein	Mg2+ transporter protein, CorA family protein	CorA1	MGt5, COBALTt5
83	cbdbA381	DET0428	DehaBAV1_0405	VS370	transcriptional regulator	transcriptional activator, putative	putative transcriptional acitvator, Baf family	putative transcriptional acitvator, Baf family	CoaA	PNTK
84	cbdbA383	DET0429	DehaBAV1_0406	VS371	phosphopantothenoyl cysteine synthetase/decarboxylase	phosphopantothenoyl cysteine decarboxylase/phosphopantothenate--cysteine ligase	phosphopantothenoylcysteine decarboxylase/phosphopantothenate--cysteine ligase(EC:4.1.1.36,EC:6.3.2.5)	phosphopantothenoylcysteine decarboxylase/phosphopantothenate--cysteine ligase	Dfp	PPCDC, PPNCL

Model gene number	CBDB1 core metabolic genes	195 core metabolic genes	BAV1 core metabolic genes	VS core metabolic genes	CBDB1 annotation	195 annotation	BAV1 annotation	VS annotation	Model protein	Model reaction
85	cbdbA384	DET0430	DehaBAV1_0407	VS372	valyl-tRNA synthetase	valyl-tRNA synthetase(EC:6.1.1.9)	valyl-tRNA synthetase(EC:6.1.1.9)	valyl-tRNA synthetase(EC:6.1.1.9)	ValS	VALTRS
86	cbdbA389	DET0435	DehaBAV1_0412	VS377	ribose-phosphate pyrophosphokinase	ribose-phosphate pyrophosphokinase(EC:2.7.6.1)	ribose-phosphate pyrophosphokinase(EC:2.7.6.1)	ribose-phosphate pyrophosphokinase (EC:2.7.6.1)	Prs	PRPPS
87	cbdbA390	DET0436	DehaBAV1_0413	VS378	serine hydroxymethyltransferase	Serine hydroxymethyltransferase(EC:2.1.2.1)	Glycine hydroxymethyltransferase(EC:2.1.2.1)	Glycine hydroxymethyltransferase(EC:2.1.2.1)	GlyA	GHMT, ALATA_L2, ALATA_D2
88	cbdbA392	DET0438	DehaBAV1_0415	VS380	metallo-beta-lactamase family protein	metallo-beta-lactamase family protein	beta-lactamase domain protein	beta-lactamase domain protein	GloB	GLYOX
89	cbdbA400	DET0444	DehaBAV1_0421	VS386	conserved hypothetical protein	conserved hypothetical protein TIGR01033	protein of unknown function DUF28	protein of unknown function DUF28	GlgC	GLGC
90	cbdbA401	DET0445	DehaBAV1_0422	VS387	holo-(acyl-carrier-protein) synthase	holo-(acyl-carrier-protein) synthase(EC:2.7.8.7)	holo-acyl-carrier-protein synthase(EC:2.7.8.7)	holo-acyl-carrier-protein synthase	AcpS	ACPS1
91	cbdbA403	DET0446	DehaBAV1_0423	VS388	putative [Fe] hydrogenase, HymA subunit	[Fe] hydrogenase, HymA subunit, putative	NADH dehydrogenase (ubiquinone), 24 kDa subunit	NADH dehydrogenase (ubiquinone), 24 kDa subunit	Nadhd5	NADH6, NADH7, NADH4, NADH8
92	cbdbA404	DET0447	DehaBAV1_0424	VS389	acyl CoA biotin-dependant carboxyltransferase	acyl CoA biotin-dependant carboxyltransferase(EC:6.4.1.3)	carboxyl transferase(EC:6.4.1.3)	carboxyl transferase(EC:6.4.1.3)	YngE	PPCOAC
93	cbdbA405	DET0448	DehaBAV1_0425	VS390	homoaconitate hydratase family protein	3-isopropylmalate dehydratase	homoaconitate hydratase family protein(EC:4.2.1.33)	homoaconitate hydratase family protein(EC:4.2.1.33)	LeuCD, Aco3	MICITH, ACONT, IPPMIb, IPPMIa, IPMI1, IPMI2
94	cbdbA407	DET0449	DehaBAV1_0426	VS391	aconitase C-terminal domain protein	isopropylmalate isomerase small subunit	3-isopropylmalate dehydratase, small subunit	3-isopropylmalate dehydratase, small subunit	LeuCD, Aco3	MICITH, ACONT, IPPMIb, IPPMIa, IPMI1, IPMI2

Model gene number	CBDB1 core metabolic genes	195 core metabolic genes	BAV1 core metabolic genes	VS core metabolic genes	CBDB1 annotation	195 annotation	BAV1 annotation	VS annotation	Model protein	Model reaction
95	cbdbA408	DET0450	DehaBAV1_0427	VS392	putative isocitrate dehydrogenase	isocitrate dehydrogenase, putative(EC:1.1.1.41)	Isocitrate dehydrogenase (NAD(+))(EC:1.1.1.41)	Isocitrate dehydrogenase (NAD(+))(EC:1.1.1.41)	Icd	ICDHy
96	cbdbA409	DET0451	DehaBAV1_0428	VS393	malate dehydrogenase, NAD-dependent	malate dehydrogenase, NAD-dependent(EC:1.1.1.37)	malate dehydrogenase, NAD-dependent	malate dehydrogenase, NAD-dependent	Ldh, Mdh	MDH, LDH_L
97	cbdbA411	DET0453	DehaBAV1_0430	VS395	putative fumarate hydratase, alpha subunit	fumarate hydratase	hydro-lyase, Fe-S type, tartrate/fumarate subfamily, alpha subunit(EC:4.2.1.2)	hydro-lyase, Fe-S type, tartrate/fumarate subfamily, alpha subunit	TtdA	FUM
98	cbdbA413	DET0454	DehaBAV1_0431	VS396	putative fumarate hydratase, beta subunit	fumarate hydratase, beta subunit, putative	hydro-lyase, Fe-S type, tartrate/fumarate subfamily, beta subunit	hydro-lyase, Fe-S type, tartrate/fumarate subfamily, beta subunit	FumB	FUM
99	cbdbA416	DET0456	DehaBAV1_0433	VS398	MutT	MutT/nudix family protein	NUDIX hydrolase	NUDIX hydrolase	NudF	ADPRDP
100	cbdbA417	DET0457	DehaBAV1_0434	VS399	acetyltransferase, GNAT family	acetyltransferase, GNAT family	GCN5-related N-acetyltransferase	GCN5-related N-acetyltransferase	ArgA	ACGS
101	cbdbA419	DET0458	DehaBAV1_0435	VS400	inorganic polyphosphate	inorganic polyphosphate/ATP-NAD kinase, putative	ATP-NAD/AcoX kinase	NAD(+) kinase(EC:2.7.1.23)	NadK	NADK
102	cbdbA423	DET0460	DehaBAV1_0437	VS403	prephenate dehydrogenase	prephenate dehydrogenase(EC:1.3.1.12)	Prephenate dehydrogenase	Prephenate dehydrogenase	YlbQ, TyrA	DPR, PPND
103	cbdbA424	DET0461	DehaBAV1_0438	VS404	chorismate mutase/prephenate dehydratase (P-protein)	chorismate mutase/prephenate dehydratase(EC:5.4.99.5)	prephenate dehydratase	prephenate dehydratase	PheA	PPNDH, CHORM
104	cbdbA425	DET0462	DehaBAV1_0439	VS405	chorismate synthase	chorismate synthase(EC:4.2.3.5,EC:4.6.1.4)	Chorismate synthase(EC:4.2.3.5)	Chorismate synthase(EC:4.2.3.5)	AroC1	CHORS

Model gene number	CBDB1 core metabolic genes	195 core metabolic genes	BAV1 core metabolic genes	VS core metabolic genes	CBDB1 annotation	195 annotation	BAV1 annotation	VS annotation	Model protein	Model reaction
105	cbdbA427	DET0463	DehaBAV1_0440	VS406	3-phosphoshikimate 1-carboxyvinyltransferase	3-phosphoshikimate 1-carboxyvinyltransferase(EC:2.5.1.19)	3-phosphoshikimate 1-carboxyvinyltransferase	3-phosphoshikimate 1 carboxyvinyltransferase	Cmk, AroA	PSCVT, CYTK2, UMPK, CYTK1
106	cbdbA428	DET0464	DehaBAV1_0441	VS407	shikimate kinase	shikimate kinase(EC:2.7.1.71)	Shikimate kinase(EC:2.7.1.71)	Shikimate kinase(EC:2.7.1.71)	AroK	SHKK
107	cbdbA429	DET0465	DehaBAV1_0442	VS408	shikimate 5-dehydrogenase	shikimate 5-dehydrogenase(EC:1.1.1.25)	shikimate 5-dehydrogenase	shikimate 5-dehydrogenase(EC:1.1.1.25)	AroE1	SHK3D
108	cbdbA430	DET0466	DehaBAV1_0443	VS409	3-dehydroquinate dehydratase, type I	3-dehydroquinate dehydratase, type I(EC:4.2.1.10)	3-dehydroquinate dehydratase, type I(EC:4.2.1.10)	3-dehydroquinate dehydratase, type I(EC:4.2.1.10)	AroD1	DHQD
109	cbdbA431	DET0467	DehaBAV1_0444	VS410	3-dehydroquinate synthase	3-dehydroquinate synthase(EC:4.2.3.4)	3-dehydroquinate synthase(EC:4.2.3.4)	3-dehydroquinate synthase(EC:4.2.3.4)	AroB	DHQS
110	cbdbA432	DET0468	DehaBAV1_0445	VS411	phospho-2-dehydro-3-deoxyheptonate aldolase	3-deoxy-7-phosphoheptulonate synthase(EC:2.5.1.54,EC:4.1.2.15)	phospho-2-dehydro-3-deoxyheptonate aldolase(EC:2.5.1.54)	phospho-2-dehydro-3-deoxyheptonate aldolase(EC:2.5.1.54)	AroG	DAHPS
111	cbdbA459	DET0495	DehaBAV1_0472	VS437	adenylate kinase	adenylate kinase(EC:2.7.4.3)	Nucleoside-triphosphate--adenylate kinase(EC:2.7.4.10,EC:2.7.4.3)	adenylate kinase(EC:2.7.4.3)	Adk	ADK1, ADK4, ADNK1, ADK3, ADK2, DADK
112	cbdbA468	DET0504	DehaBAV1_0481	VS446	tRNA pseudouridine synthase A	tRNA pseudouridine synthase A(EC:4.2.1.70)	tRNA pseudouridine synthase A(EC:5.4.99.12)	tRNA pseudouridine synthase A(EC:4.2.1.70)	RluB	PSUDS
113	cbdbA472	DET0509	DehaBAV1_0485	VS450	SIS domain protein	hypothetical protein	bifunctional phosphoglucose/phosphomannose isomerase(EC:5.3.1.8)	bifunctional phosphoglucose/phosphomannose isomerase	Pgi, ManA	MAN6PI, PGI

Model gene number	CBDB1 core metabolic genes	195 core metabolic genes	BAV1 core metabolic genes	VS core metabolic genes	CBDB1 annotation	195 annotation	BAV1 annotation	VS annotation	Model protein	Model reaction
114	cbdbA474	DET0510	DehaBAV1_0486	VS451	phosphoglucomutase	phosphoglucomutase/phosphomannomutase family protein	Phosphomannomutase(EC:5.4.2.8)	Phosphomannomutase(EC:5.4.2.8)	GlmM, Pmgm1	PGAMT, PGMT, PMANM
115	cbdbA476	DET0512	DehaBAV1_0488	VS453	S-adenosylmethionine synthetase	S-adenosylmethionine synthetase(EC:2.5.1.6)	Methionine adenosyltransferase(EC:2.5.1.6)	Methionine adenosyltransferase(EC:2.5.1.6)	MetK	METAT
116	cbdbA477	DET0513	DehaBAV1_0489	VS454	adenosylhomocysteinase	S-adenosyl-L-homocysteine hydrolase(EC:3.3.1.1)	adenosylhomocysteinase(EC:3.3.1.1)	adenosylhomocysteinase(EC:3.3.1.1)	AhcY	AHC
117	cbdbA482	DET0517	DehaBAV1_0493	VS458	methylthioadenosine phosphorylase	methylthioadenosine phosphorylase(EC:2.4.2.28)	methylthioadenosine phosphorylase(EC:2.4.2.28)	methylthioadenosine phosphorylase(EC:2.4.2.28)	MtaP	MTAP
118	cbdbA484	DET0518	DehaBAV1_0494	VS459	translation initiation factor, putative,aIF-2BIfamily	translation initiation factor, putative, aIF-2BI family(EC:5.3.1.23)	putative translation initiation factor, aIF-2BI family(EC:5.3.1.23)	putative translation initiation factor, aIF-2BI family(EC:5.3.1.23)	YkrS	MTRI
119	cbdbA490	DET0523	DehaBAV1_0499	VS464	acetyltransferase, GNAT family	acetyltransferase, GNAT family	GCN5-related N-acetyltransferase	GCN5-related N-acetyltransferase	ArgA	ACGS
120	cbdbA497	DET0528	DehaBAV1_0504	VS469	phosphomannomutase	phosphoglucomutase/phosphomannomutase family protein	Phosphoglucosamine mutase(EC:5.4.2.10)	phosphoglucomutase/phosphomannomutase alpha/beta/alpha domain I	GlmM, Pmgm1	PGAMT, PGMT, PMANM
121	cbdbA499	DET0529	DehaBAV1_0505	VS470	glucose-1-phosphate thymidylyltransferase	glucose-1-phosphate thymidylyltransferase (EC:2.7.7.24)	Nucleotidyl transferase	Nucleotidyl transferase	DapD, GlmU, RfbA, GalF	THDPS, G1PTMT, G1PACT, GALU, UAGDP
122	cbdbA500	DET0530	DehaBAV1_0506	VS471	glucose-1-phosphate thymidylyltransferase	glucose-1-phosphate thymidylyltransferase (EC:2.7.7.24)	Nucleotidyl transferase	Nucleotidyl transferase	GalF, RfbA, GlmU	G1PTMT, G1PACT, UAGDP, GALU

Model gene number	CBDB1 core metabolic genes	195 core metabolic genes	BAV1 core metabolic genes	VS core metabolic genes	CBDB1 annotation	195 annotation	BAV1 annotation	VS annotation	Model protein	Model reaction
123	cbdbA503	DET0531	DehaBAV1_ 0507	VS472	glucosamine-fructose-6-phosphateaminotransferase, isomerizing	glucosamine--fructose-6-phosphate aminotransferase, isomerizing(EC:2.6.1.16)	glucosamine--fructose-6-phosphate aminotransferase, isomerizing(EC:2.6.1.16)	glucosamine--fructose-6-phosphate aminotransferase, isomerizing(EC:2.6.1.16)	GlmS	GF6PTA
124	cbdbA508	DET0534	DehaBAV1_ 0510	VS475	diaminopimelate decarboxylase	diaminopimelate decarboxylase(EC:4.1.1.20)	diaminopimelate decarboxylase(EC:4.1.1.20)	diaminopimelate decarboxylase(EC:4.1.1.20)	SpeC, LysA, SpeA	ARGDC, DAPDC, ORNDC
125	cbdbA514	DET0540	DehaBAV1_ 0516	VS481	replicative DNA helicase	replicative DNA helicase(EC:3.6.1.-)	replicative DNA helicase	replicative DNA helicase	NtpA	DNMPPA, DNTPPA
126	cbdbA516	DET0542	DehaBAV1_ 0518	VS483	thioredoxin reductase	thioredoxin-disulfide reductase(EC:1.8.1.9)	thioredoxin reductase	thioredoxin reductase	TrxB	TRDR
127	cbdbA520	DET0545	DehaBAV1_ 0521	VS486	amidohydrolase family protein	amidohydrolase family protein(EC:3.5.1.-)	amidohydrolase	amidohydrolase	DapE, Phyd	SDPDS, PNTEH
128	cbdbA528	DET0553	DehaBAV1_ 0528	VS493	putative deoxyguanosinetriphosphate triphosphohydrolase	deoxyguanosinetriphosphate triphosphohydrolase-like protein	putative deoxyguanosinetriphosphate triphosphohydrolase	putative deoxyguanosinetriphosphate triphosphohydrolase	Dgt	NTPTP2, DGTPH
129	cbdbA529	DET0554	DehaBAV1_ 0529	VS494	phosphoenolpyruvate synthase	phosphoenolpyruvate synthase(EC:2.7.9.2)	phosphoenolpyruvate synthase(EC:2.7.9.2)	phosphoenolpyruvate synthase(EC:2.7.9.2)	PpdK, Ppsa, Pyk	PPS, PYK, PPDK
130	cbdbA532	DET0558	DehaBAV1_ 0532	VS497	ATP synthase F0, A subunit	ATP synthase F0, A subunit(EC:3.6.3.14)	ATP synthase F0, A subunit	ATP synthase F0, A subunit	AtpABCDEF GHI	ATPS3r
131	cbdbA533	DET0559	DehaBAV1_ 0533	VS498	ATP synthase F0, C subunit	ATP synthase F0, C subunit(EC:3.6.3.14)	ATP synthase F0, C subunit	ATP synthase F0, C subunit	AtpABCDEF GHI	ATPS3r
132	cbdbA534	DET0560	DehaBAV1_ 0534	VS499	ATP synthase F0, B subunit	ATP synthase F0, B subunit(EC:3.6.3.14)	ATP synthase F0, B subunit	ATP synthase F0, B subunit	AtpABCDEF GHI	ATPS3r

Model gene number	CBDB1 core metabolic genes	195 core metabolic genes	BAV1 core metabolic genes	VS core metabolic genes	CBDB1 annotation	195 annotation	BAV1 annotation	VS annotation	Model protein	Model reaction
133	cbdbA535	DET0561	DehaBAV1_ 0535	VS500	ATP synthase F1, delta subunit	ATP synthase F1, delta subunit(EC:3.6.3.14)	ATP synthase F1, delta subunit	ATP synthase F1, delta subunit	AtpABCDEF GHI	ATPS3r
134	cbdbA536	DET0562	DehaBAV1_ 0536	VS501	ATP synthase F1, alpha subunit	ATP synthase subunit A(EC:3.6.3.14)	ATP synthase F1, alpha subunit(EC:3.6.3.14)	ATP synthase F1, alpha subunit(EC:3.6.3.15)	AtpABCDEF GHI	ATPS3r
135	cbdbA537	DET0563	DehaBAV1_ 0537	VS502	ATP synthase F1, gamma subunit	ATP synthase F1, gamma subunit(EC:3.6.3.14)	ATP synthase F1, gamma subunit(EC:3.6.3.14)	ATP synthase F1, gamma subunit(EC:3.6.3.15)	AtpABCDEF GHI	ATPS3r
136	cbdbA538	DET0564	DehaBAV1_ 0538	VS503	ATP synthase F1, beta subunit	ATP synthase subunit B(EC:3.6.3.14)	ATP synthase F1, beta subunit	ATP synthase F1, beta subunit(EC:3.6.3.15)	AtpABCDEF GHI	ATPS3r
137	cbdbA539	DET0565	DehaBAV1_ 0539	VS504	ATP synthase F1, epsilon subunit	ATP synthase F1, epsilon subunit(EC:3.6.3.14)	ATP synthase F1, epsilon subunit	ATP synthase F1, epsilon subunit	AtpABCDEF GHI	ATPS3r
138	cbdbA547	DET0573	DehaBAV1_ 0547	VS512	ABC-type dipeptide/oligopeptide/ nickel transport system, periplasmic component	oligopeptide-binding protein, putative	extracellular solute-binding protein, family 5	extracellular solute-binding protein, family 5	NikA	NIabc
139	cbdbA553	DET0576	DehaBAV1_ 0549	VS515	aminotransferase, classes I and II	aminotransferase, classes I and II	aminotransferase, class I and II	aminotransferase, class I and II	AspC	ASPTA1
140	cbdbA554	DET0577	DehaBAV1_ 0550	VS516	seryl-tRNA synthetase	seryl-tRNA synthetase(EC:6.1.1.11)	seryl-tRNA synthetase(EC:6.1.1.11)	seryl-tRNA synthetase(EC:6.1.1.11)	SerS	SERTRS
141	cbdbA555	DET0578	DehaBAV1_ 0551	VS517	lysyl-tRNA synthetase	lysyl-tRNA synthetase(EC:6.1.1.6)	lysyl-tRNA synthetase(EC:6.1.1.6)	lysyl-tRNA synthetase(EC:6.1.1.6)	LysS	LYSTRS
142	cbdbA560	DET0582	DehaBAV1_ 0556	VS521	magnesium and cobalt transport protein CorA	magnesium and cobalt transport protein CorA	magnesium and cobalt transport protein CorA	magnesium and cobalt transport protein CorA	CorA1	MGt5, COBALTt5
143	cbdbA569	DET0590	DehaBAV1_ 0565	VS531	glyceraldehyde-3-phosphate dehydrogenase, typeI	glyceraldehyde-3-phosphate dehydrogenase, type I(EC:1.2.1.- ,EC:1.2.1.12)	glyceraldehyde-3-phosphate dehydrogenase, type I(EC:1.2.1.12)	glyceraldehyde-3-phosphate dehydrogenase, type I(EC:1.2.1.12)	Epd, Gap	GAPD, E4PD

Model gene number	CBDB1 core metabolic genes	195 core metabolic genes	BAV1 core metabolic genes	VS core metabolic genes	CBDB1 annotation	195 annotation	BAV1 annotation	VS annotation	Model protein	Model reaction
144	cbdbA573	DET0593	DehaBAV1_ 0568	VS533	enolase	enolase(EC:4.2.1.11)	Phosphopyruvate hydratase(EC:4.2.1.11)	Phosphopyruvate hydratase(EC:4.2.1.11)	Eno	ENO
145	cbdbA580	DET0599	DehaBAV1_ 0574	VS539	D-3-phosphoglycerate dehydrogenase	D-3-phosphoglycerate dehydrogenase(EC:1.1.1.95)	D-3-phosphoglycerate dehydrogenase	D-3-phosphoglycerate dehydrogenase	SerA2	PGCDr, PGCD
146	cbdbA581	DET0600	DehaBAV1_ 0575	VS540	soluble hydrogenase, tritium exchange subunit	soluble hydrogenase, tritium exchange subunit(EC:1.12.-)	aminotransferase, class V	Serine--glyoxylate transaminase(EC:2.6.1.45)	SerC	PSERTr
147	cbdbA583	DET0601	DehaBAV1_ 0576	VS541	tyrosyl-tRNA synthetase	tyrosyl-tRNA synthetase(EC:6.1.1.1)	tyrosyl-tRNA synthetase	tyrosyl-tRNA synthetase	TyrS	TYRTRS
148	cbdbA584	DET0602	DehaBAV1_ 0577	VS542	riboflavin biosynthesis protein RibF	riboflavin biosynthesis protein RibF	riboflavin biosynthesis protein RibF	riboflavin biosynthesis protein RibF	RibF	FMNAT, RBFK
149	cbdbA596	DET0614	DehaBAV1_ 0587	VS552	hydrogenase, group 3, VhuG subunit	hydrogenase, group 3, VhuG subunit, putative	NADH ubiquinone oxidoreductase, 20 kDa subunit	NADH ubiquinone oxidoreductase, 20 kDa subunit	Nadhd5	NADH6, NADH7, NADH4, NADH8
150	cbdbA597	DET0615	DehaBAV1_ 0588	VS553	hydrogenase, group 3, VhuA subunit	hydrogenase, group 3, VhuA subunit, putative	nickel-dependent hydrogenase, large subunit	nickel-dependent hydrogenase, large subunit	FrdH	FRHD
151	cbdbA608	DET0345	DehaBAV1_ 0324	VS564	ribonucleotide reductase	ribonucleotide-diphosphate reductase alpha subunit(EC:1.17.4.1)	ribonucleoside-diphosphate reductase, adenosylcobalamin-dependent(EC:1.17.4.1)	ribonucleoside-diphosphate reductase, adenosylcobalamin-dependent	NrdEF	RNDR3, RNDR1, RNDR4, RNDR2
152	cbdbA610	DET0624	DehaBAV1_ 0597	VS565	response regulator	response regulator	response regulator receiver modulated metal dependent phosphohydrolase	response regulator receiver modulated metal dependent phosphohydrolase	CyaA	ADNCYC

Model gene number	CBDB1 core metabolic genes	195 core metabolic genes	BAV1 core metabolic genes	VS core metabolic genes	CBDB1 annotation	195 annotation	BAV1 annotation	VS annotation	Model protein	Model reaction
153	cbdbA615	DET0628	DehaBAV1_0601	VS569	lysine 2,3-aminomutase	radical SAM domain protein	lysine 2,3-aminomutase YodO family protein(EC:5.4.3.2)	lysine 2,3-aminomutase YodO family protein	KamA	LYSAM
154	cbdbA616	DET0629	DehaBAV1_0602	VS570	D-ala D-alanine ligase	D-ala D-ala ligase(EC:6.3.2.4)	D-alanine--D-alanine ligase(EC:6.3.2.4)	D-alanine--D-alanine ligase(EC:6.3.2.4)	MurB, Ddl	UAPGR, ALAALA
155	cbdbA618	DET0631	DehaBAV1_0604	VS572	cation ABC transporter, periplasmc-binding protein	cation ABC transporter, periplasmc-binding protein	periplasmic solute binding protein	periplasmic solute binding protein	ZnuA	ZN2abc2, MNabc
156	cbdbA619	DET0632	DehaBAV1_0605	VS573	cation ABC transporter, ATP-binding protein	cation ABC transporter, ATP-binding protein	ABC transporter related	ABC transporter related	ZnuC	ZN2abc2, MNabc
157	cbdbA620	DET0633	DehaBAV1_0606	VS574	cation ABC transporter, permease protein	cation ABC transporter, permease protein	ABC-3 protein	ABC-3 protein	ZnuB	ZN2abc2, MNabc
158	cbdbA627	DET0642	DehaBAV1_0614	VS582	putative ribulose-phosphate 3-epimerase	ribulose-phosphate 3-epimerase, putative	Ribulose-phosphate 3-epimerase(EC:5.1.3.1)	Ribulose-phosphate 3-epimerase(EC:5.1.3.1)	SgcE	RPE
159	cbdbA628	DET0643	DehaBAV1_0615	VS583	putative ribose-5-phosphate isomerase	ribose-5-phosphate isomerase B	sugar-phosphate isomerase, RpiB/LacA/LacB family	sugar-phosphate isomerase, RpiB/LacA/LacB family	RpiB	RPI
160	cbdbA629	DET0644	DehaBAV1_0616	VS584	transketolase	transketolase(EC:2.2.1.1)	transketolase	transketolase	TktB	TKT1, TKT2
161	cbdbA633	DET0650	DehaBAV1_0620	VS588	ABC-type cobalamin/Fe3+-siderophores transport system, periplasmic component	ABC-type cobalamin/Fe3+-siderophores transport systems, periplasmic binding protein, putative	periplasmic binding protein	periplasmic binding protein	BtuF	CBL1abc

Model gene number	CBDB1 core metabolic genes	195 core metabolic genes	BAV1 core metabolic genes	VS core metabolic genes	CBDB1 annotation	195 annotation	BAV1 annotation	VS annotation	Model protein	Model reaction
162	cbdbA635	DET0651	DehaBAV1_0621	VS589	ABC-type cobalamin/Fe3+-siderophores transport system, permease component	ABC-type cobalamin/Fe3+-siderophores transport systems, permease component	transport system permease protein	transport system permease protein	BtuC	CBL1abc
163	cbdbA636	DET0652	DehaBAV1_0622	VS590	ABC-type cobalamin/Fe3+-siderophores transport system, ATPase component	ABC-type cobalamin/Fe3+-siderophores transport system, ATP-binding protein	ABC transporter related	ABC transporter related	BtuD	CBL1abc
164	cbdbA638	DET0654	DehaBAV1_0624	VS592	cobalamin biosynthesis protein	cobalamin biosynthesis protein CobD	cobalamin biosynthesis protein CobD	cobalamin biosynthesis protein CobD	CbiB	ADCOBAS, ADCPS2
165	cbdbA639	DET0655	DehaBAV1_0625	VS593	putative threonine-phosphate decarboxylase (L-threonine-O-3-phosphate decarboxylase)	histidinol-phosphate aminotransferase, putative	aminotransferase, class I and II	aminotransferase, class I and II	CobQ-CobD	THRPD
166	cbdbA641	DET0657	DehaBAV1_0626	VS595	nicotinate-nucleotide-dimethylbenzimidazole phosphoribosyltransferase	nicotinate-nucleotide-dimethylbenzimidazole phosphoribosyltransferase(EC:2.4.2.21)	Nicotinate-nucleotide--dimethylbenzimidazole phosphoribosyltransferase(EC:2.4.2.21)	Nicotinate-nucleotide--dimethylbenzimidazole phosphoribosyltransferase(EC:2.4.2.21)	CobT	NNDMBRT3, NNDMBRT
167	cbdbA642	DET0658	DehaBAV1_0627	VS596	cobalamin 5-phosphate synthase	cobalamin 5'-phosphate synthase	cobalamin 5'-phosphate synthase	cobalamin 5'-phosphate synthase	CobS	COBPS, COBPS2
168	cbdbA643	DET0659	DehaBAV1_0628	VS597	alpha-ribazole-5-phosphate phosphatase	alpha-ribazole-5-phosphate phosphatase, putative	Phosphoglycerate mutase	Phosphoglycerate mutase	CobC	RBZP

Model gene number	CBDB1 core metabolic genes	195 core metabolic genes	BAV1 core metabolic genes	VS core metabolic genes	CBDB1 annotation	195 annotation	BAV1 annotation	VS annotation	Model protein	Model reaction
169	cbdbA644	DET0660	DehaBAV1_0629	VS598	cobinamide kinase	cobinamide kinase/cobinamide phosphate guanylyltransferase	cobalbumin biosynthesis enzyme	cobalbumin biosynthesis enzyme	CobU	ACBIPGT, ADCOBAK
170	cbdbA651	DET0665	DehaBAV1_0634	VS603	carbon monoxide dehydrogenase/acetyl-CoA synthase large subunit	acetyl-CoA decarbonylase/synthase complex subunit gamma	CO dehydrogenase/acetyl-CoA synthase delta subunit	CO dehydrogenase/acetyl-CoA synthase delta subunit	CooS	CODHr
171	cbdbA652	DET0666	DehaBAV1_0635	VS604	carbon monoxide dehydrogenase, alpha subunit	acetyl-CoA decarbonylase/synthase complex subunit beta(EC:1.2.99.2)	CO dehydrogenase/acetyl-CoA synthase complex, beta subunit(EC:2.3.1.169)	CO dehydrogenase/acetyl-CoA synthase complex, beta subunit(EC:2.3.1.169)	CooS	CODHr
172	cbdbA654	DET0667	DehaBAV1_0636	VS605	carbon monoxide dehydrogenase/acetyl-CoA synthase small subunit	acetyl-CoA decarbonylase/synthase complex subunit delta	CO dehydrogenase/acetyl-CoA synthase delta subunit	CO dehydrogenase/acetyl-CoA synthase delta subunit	CooS	CODHr
173	cbdbA656	DET0668	DehaBAV1_0637	VS606	methylenetetrahydrofolate dehydrogenase	methylenetetrahydrofolate dehydrogenase/methenyltetrahydrofolate cyclohydrolase(EC:1.5.1.5)	Methenyltetrahydrofolate cyclohydrolase(EC:1.5.1.5,EC:3.5.4.9)	Methylenetetrahydrofolate dehydrogenase (NADP(+))(EC:1.5.1.5)	FolD	MTHFC, MTHFD
174	cbdbA658	DET0669	DehaBAV1_0638	VS607	carbon monoxide dehydrogenase nickel-insertion accessory protein	carbon monoxide dehydrogenase nickel-insertion accessory protein	CO dehydrogenase maturation factor-like protein	carbon monoxide dehydrogenase nickel-insertion accessory protein	CooS	CODHr
175	cbdbA660	DET0671	DehaBAV1_0640	VS609	formate-tetrahydrofolate ligase	formate--tetrahydrofolate ligase(EC:6.3.4.3)	Formate--tetrahydrofolate ligase(EC:6.3.4.3)	Formate--tetrahydrofolate ligase(EC:6.3.4.3)	Fhs	FTHFLr, MTHFC, MTHFD

Model gene number	CBDB1 core metabolic genes	195 core metabolic genes	BAV1 core metabolic genes	VS core metabolic genes	CBDB1 annotation	195 annotation	BAV1 annotation	VS annotation	Model protein	Model reaction
176	cbdbA662	DET0673	DehaBAV1_0642	VS611	hypothetical protein	hypothetical protein	hypothetical protein	hypothetical protein	LpxB	LPADSS
177	cbdbA663	DET0708	DehaBAV1_0643	VS612	aspartyl-tRNA synthetase	aspartyl-tRNA synthetase(EC:6.1.1.12)	aspartyl-tRNA synthetase	aspartyl-tRNA synthetase	AspS	ASPTRS
178	cbdbA667	DET0711	DehaBAV1_0646	VS618	carbohydrate kinase, PfkB family	carbohydrate kinase, PfkB family	PfkB domain protein	PfkB domain protein	RbsK	RBK
179	cbdbA668	DET0712	DehaBAV1_0647	VS619	probable phosphoribosylglycinamide transformylase	phosphoribosylglycinamide transformylase, putative	Folate-dependent phosphoribosylglycinamide formyltransferase PurN-like protein	formyl transferase domain protein	PurN	GARFT
180	cbdbA678	DET0722	DehaBAV1_0655	VS628	putative antiporter GerN	sodium/hydrogen exchanger family protein	sodium/hydrogen exchanger	sodium/hydrogen exchanger	KefC2	Kt6
181	cbdbB16	DET0724	DehaBAV1_0656	VS630	pyruvic-ferredoxin oxidoreductase, gammasubunit	pyruvic-ferredoxin oxidoreductase, gamma subunit(EC:1.2.7.1)	pyruvate/ketoisovalerate oxidoreductase, gamma subunit(EC:1.2.7.1)	pyruvate/ketoisovalerate oxidoreductase, gamma subunit(EC:1.2.7.1)	Pfor, Ofo	OOR2r, POR
182	cbdbA680	DET0725	DehaBAV1_0657	VS631	pyruvic-ferredoxin oxidoreductase, delta subunit	pyruvic-ferredoxin oxidoreductase, delta subunit(EC:1.2.7.1)	pyruvate ferredoxin/flavodoxin oxidoreductase, delta subunit	pyruvate ferredoxin/flavodoxin oxidoreductase, delta subunit	Pfor, Ofo	OOR2r, POR
183	cbdbA681	DET0726	DehaBAV1_0658	VS632	pyruvic-ferredoxin oxidoreductase, alpha subunit	pyruvic-ferredoxin oxidoreductase, alpha subunit(EC:1.2.7.1)	pyruvate flavodoxin/ferredoxin oxidoreductase domain protein	pyruvate flavodoxin/ferredoxin oxidoreductase domain protein	Pfor, Ofo, PdhAB	OOR2r, PDH, POR
184	cbdbA682	DET0727	DehaBAV1_0659	VS633	pyruvic-ferredoxin oxidoreductase, beta subunit	pyruvic-ferredoxin oxidoreductase, beta subunit(EC:1.2.7.1)	thiamine pyrophosphate enzyme domain protein TPP-binding	thiamine pyrophosphate enzyme domain protein TPP-binding	Pfor, Ofo	OOR2r, POR

Model gene number	CBDB1 core metabolic genes	195 core metabolic genes	BAV1 core metabolic genes	VS core metabolic genes	CBDB1 annotation	195 annotation	BAV1 annotation	VS annotation	Model protein	Model reaction
185	cbdbA683	DET0728	DehaBAV1_0660	VS634	putative [Fe] hydrogenase, HymA subunit	[Fe] hydrogenase, HymA subunit, putative	NADH dehydrogenase (ubiquinone), 24 kDa subunit	NADH dehydrogenase (ubiquinone), 24 kDa subunit	Nadhd5	NADH6, NADH7, NADH4, NADH8
186	cbdbA684	DET0729	DehaBAV1_0661	VS635	putative [Fe] hydrogenase, HymB subunit	[Fe] hydrogenase, HymB subunit, putative	NADH dehydrogenase (quinone)(EC:1.6.99.5)	NADH dehydrogenase (quinone)(EC:1.6.99.5)	Nadhd5	NADH6, NADH7, NADH4, NADH8
187	cbdbA685	DET0730	DehaBAV1_0662	VS636	putative [Fe] hydrogenase, HymC subunit	[Fe] hydrogenase, HymC subunit, putative	[Fe] hydrogenase, HymC subunit, putative	4Fe-4S ferredoxin, iron-sulfur binding domain protein	Nadhd5, Nadphd, Fdx	NADPH12, NADH6, NADH7, NADH4, FRHD, NADH8
188	cbdbA687	DET0732	DehaBAV1_0664	VS638	mercuric reductase	mercuric reductase, putative	FAD-dependent pyridine nucleotide-disulphide oxidoreductase	FAD-dependent pyridine nucleotide-disulphide oxidoreductase	LpdA, MerA	HGR, GCCc
189	cbdbA689	DET0736	DehaBAV1_0666	VS642	oxidoreductase, short chaindehydrogenase	oxidoreductase, short chain dehydrogenase/reductase family	short-chain dehydrogenase/reductase SDR	short-chain dehydrogenase/reductase SDR	FabG1	HDER4, HBUR1, HTDR6, HDDR5, HICR9, HOCR3, HHYR2, HHDR7, HODR8_#1
190	cbdbA714	DET0739	DehaBAV1_0669	VS646	putative aspartate aminotransferase	aspartate aminotransferase	aminotransferase, class I and II	aminotransferase, class I and II	DapAT, AspC	ASPTA1, DAPAT
191	cbdbA715	DET0740	DehaBAV1_0670	VS647	diaminopimelate epimerase	diaminopimelate epimerase(EC:5.1.1.7)	Diaminopimelate epimerase(EC:5.1.1.7)	Diaminopimelate epimerase(EC:5.1.1.7)	DapF	DAPE
192	cbdbA717	DET0742	DehaBAV1_0672	VS649	triosephosphate isomerase	triosephosphate isomerase(EC:5.3.1.1)	Triose-phosphate isomerase(EC:5.3.1.1)	Triose-phosphate isomerase(EC:5.3.1.1)	TpiA	TPI

Model gene number	CBDB1 core metabolic genes	195 core metabolic genes	BAV1 core metabolic genes	VS core metabolic genes	CBDB1 annotation	195 annotation	BAV1 annotation	VS annotation	Model protein	Model reaction
193	cbdbA718	DET0743	DehaBAV1_0673	VS650	putative phosphoglycerate mutase,2,3-bisphosphoglycerate-independent	cofactor-independent phosphoglycerate mutase	phosphonopyruvate decarboxylase-related protein(EC:5.4.2.1)	phosphonopyruvate decarboxylase-related protein(EC:5.4.2.1)	Pgml	PGM
194	cbdbA719	DET0744	DehaBAV1_0674	VS651	phosphoglycerate kinase	phosphoglycerate kinase(EC:2.7.2.3)	Phosphoglycerate kinase(EC:2.7.2.3)	Phosphoglycerate kinase(EC:2.7.2.3)	Pgk	PGK
195	cbdbA720	DET0745	DehaBAV1_0675	VS652	deoxyxylulose-5-phosphate synthase	1-deoxy-D-xylulose-5-phosphate synthase(EC:2.2.1.7)	deoxyxylulose-5-phosphate synthase(EC:2.2.1.7)	deoxyxylulose-5-phosphate synthase(EC:2.2.1.7)	Dxs	DXPS
196	cbdbA722	DET0749	DehaBAV1_0677	VS655	putative hypoxanthine phosphoribosyl transferase with additional GAF motif	hypoxanthine phosphoribosyltransferase(EC:2.4.2.8)	putative GAF sensor protein(EC:2.4.2.8)	putative GAF sensor protein(EC:2.4.2.8)	Hpt	GUAPRT, HXPRT
197	cbdbA726	DET0753	DehaBAV1_0681	VS659	threonyl-tRNA synthetase	threonyl-tRNA synthetase(EC:6.1.1.3)	threonyl-tRNA synthetase	Anticodon-binding domain protein	ThrS	THRTRS
198	cbdbA730	DET0758	DehaBAV1_0685	VS664	conserved hypothetical protein	conserved hypothetical protein TIGR00044	alanine racemase domain protein	alanine racemase domain protein	DapE	SDPDS
199	cbdbA733	DET0760	DehaBAV1_0687	VS666	polypeptide deformylase	peptide deformylase(EC:3.5.1.88)	peptide deformylase(EC:3.5.1.88)	peptide deformylase(EC:3.5.1.88)	Def	FMETDF
200	cbdbA738	DET0766	DehaBAV1_0692	VS672	V-type H(+)-translocating pyrophosphatase	V-type H(+)-translocating pyrophosphatase(EC:3.6.1.1)	V-type H(+)-translocating pyrophosphatase(EC:3.6.1.1)	V-type H(+)-translocating pyrophosphatase(EC:3.6.1.1)	Ppa1	PPA_1
201	cbdbA749	DET0775	DehaBAV1_0701	VS681	thiamine biosynthesis protein ThiC	thiamine biosynthesis protein ThiC	thiamine biosynthesis protein ThiC	thiamine biosynthesis protein ThiC	ThiC	AMPMS, AHMMPS
202	cbdbA752	DET0778	DehaBAV1_0704	VS684	thymidylate kinase	thymidylate kinase(EC:2.7.4.9)	dTMP kinase(EC:2.7.4.9)	dTMP kinase(EC:2.7.4.9)	Tmk	DTMPK, TMDK1

Model gene number	CBDB1 core metabolic genes	195 core metabolic genes	BAV1 core metabolic genes	VS core metabolic genes	CBDB1 annotation	195 annotation	BAV1 annotation	VS annotation	Model protein	Model reaction
203	cbdbA758	DET0782	DehaBAV1_0708	VS688	putative thiamine-phosphate pyrophosphorylase	ThiE-associated domain protein/thiamine-phosphate pyrophosphorylase	thiamine-phosphate pyrophosphorylase(EC:2.5.1.3)	thiamine-phosphate pyrophosphorylase(EC:2.5.1.3)	ThiE	HMPK4, TMPPP, HMPK1, HMPK3, HMPK2
204	cbdbA761	DET0784	DehaBAV1_0710	VS690	V-type H(+)-translocating pyrophosphatase	V-type H(+)-translocating pyrophosphatase(EC:3.6.1.1)	V-type H(+)-translocating pyrophosphatase(EC:3.6.1.1)	V-type H(+)-translocating pyrophosphatase(EC:3.6.1.1)	Ppa1	PPA_1
205	cbdbA762	DET0785	DehaBAV1_0711	VS691	metallo-beta-lactamase family protein	metallo-beta-lactamase family protein	beta-lactamase domain protein	beta-lactamase domain protein	GloB	GLYOX
206	cbdbA765	DET0787	DehaBAV1_0713	VS693	glycyl-tRNA synthetase	glycyl-tRNA synthetase(EC:6.1.1.14)	glycyl-tRNA synthetase	glycyl-tRNA synthetase	GlyQS	GLYTRS
207	cbdbA767	DET0789	DehaBAV1_0715	VS695	pseudouridine synthase, RsuA family	pseudouridine synthase, RsuA family	pseudouridine synthase	pseudouridine synthase	RluB	PSUDS
208	cbdbA769	DET0791	DehaBAV1_0717	VS697	adenine deaminase	adenine deaminase(EC:3.5.4.2)	adenine deaminase(EC:3.5.4.2)	adenine deaminase(EC:3.5.4.2)	AdeC	ADD
209	cbdbA770	DET0792	DehaBAV1_0718	VS698	hypoxanthine phosphoribosyltransferase	hypoxanthine phosphoribosyltransferase(EC:2.4.2.8)	hypoxanthine phosphoribosyltransferase(EC:2.4.2.8)	hypoxanthine phosphoribosyltransferase(EC:2.4.2.8)	Hpt	GUAPRT, HXPRT
210	cbdbA780	DET0802	DehaBAV1_0726	VS706	aspartate 1-decarboxylase	aspartate 1-decarboxylase precursor(EC:4.1.1.11)	aspartate 1-decarboxylase(EC:4.1.1.11)	aspartate 1-decarboxylase(EC:4.1.1.11)	PanD	ASP1DC
211	cbdbA781	DET0803	DehaBAV1_0727	VS707	3-methyl-2-oxobutanoate hydroxymethyltransferase	3-methyl-2-oxobutanoate hydroxymethyltransferase(EC:2.1.2.11)	3-methyl-2-oxobutanoate hydroxymethyltransferase(EC:2.1.2.11)	3-methyl-2-oxobutanoate hydroxymethyltransferase(EC:2.1.2.11)	PanB	MOHMT
212	cbdbA782	DET0804	DehaBAV1_0728	VS708	pantoate-beta-alanine ligase	pantoate--beta-alanine ligase(EC:6.3.2.1)	pantoate--beta-alanine ligase(EC:6.3.2.1)	pantoate--beta-alanine ligase(EC:6.3.2.1)	PanC	PANTS

Model gene number	CBDB1 core metabolic genes	195 core metabolic genes	BAV1 core metabolic genes	VS core metabolic genes	CBDB1 annotation	195 annotation	BAV1 annotation	VS annotation	Model protein	Model reaction
213	cbdbA795	DET0817	DehaBAV1_ 0737	VS721	ABC transporter, ATP-binding protein	ABC transporter, ATP binding protein	ABC transporter related	ABC transporter related	CysAP	TSULabc, SULabc
214	cbdbA796	DET0819	DehaBAV1_ 0738	VS723	thymidylate synthase	thymidylate synthase, putative	Thymidylate synthase-like protein	thymidylate synthase	ThyA, DfrA	TMDS, DHFOR3, DHFR, DHFOR2
215	cbdbA802	DET0824	DehaBAV1_ 0743	VS728	probable nitroreductase family protein	nitroreductase family protein	nitroreductase	nitroreductase family protein	GlgC	GLGC
216	cbdbA803	DET0825	DehaBAV1_ 0744	VS729	2-isopropylmalate synthase	2-isopropylmalate synthase	2-isopropylmalate synthase/homocitrate synthase family protein(EC:2.3.3.13)	2-isopropylmalate synthase/homocitra te synthase family protein(EC:2.3.3.13)	LeuA, CimA	IPPS, CITRMS
217	cbdbA804	DET0826	DehaBAV1_ 0745	VS730	3-isopropylmalate dehydrogenase	3-isopropylmalate dehydrogenase(EC:1.1.1.85)	3-isopropylmalate dehydrogenase(EC:1.1.1.85)	3-isopropylmalate dehydrogenase(EC:1.1.1.85)	LeuB	IPMD
218	cbdbA805	DET0827	DehaBAV1_ 0746	VS731	3-isopropylmalate dehydratase, small subunit	3-isopropylmalate dehydratase, small subunit(EC:4.2.1.33)	3-isopropylmalate dehydratase, small subunit	3-isopropylmalate dehydratase, small subunit	LeuCD, Aco3	MICITH, ACONT, IPPMIb, IPPMIa, IPMI1, IPMI2
219	cbdbA806	DET0828	DehaBAV1_ 0747	VS732	3-isopropylmalate dehydratase, large subunit	3-isopropylmalate dehydratase, large subunit(EC:4.2.1.33)	3-isopropylmalate dehydratase, large subunit(EC:4.2.1.33)	3-isopropylmalate dehydratase, large subunit(EC:4.2.1.33)	LeuCD, Aco3	MICITH, ACONT, IPPMIb, IPPMIa, IPMI1, IPMI2
220	cbdbA808	DET0830	DehaBAV1_ 0749	VS734	2-isopropylmalate synthase	2-isopropylmalate synthase(EC:2.3.3.13)	2-isopropylmalate synthase	2-isopropylmalate synthase	LeuA	IPPS
221	cbdbA811	DET0831	DehaBAV1_ 0750	VS735	ketol-acid reductoisomerase	ketol-acid reductoisomerase(EC:1.1.1.86)	ketol-acid reductoisomerase(EC:1.1.1.86)	ketol-acid reductoisomerase(EC:1.1.1.86)	IlvC, YlbQ	KARA1, DPR, KARA2
222	cbdbA812	DET0832	DehaBAV1_ 0751	VS736	acetolactate synthase, small subunit	acetolactate synthase, small subunit(EC:4.1.3.18)	acetolactate synthase, small subunit	acetolactate synthase, small subunit	IlvBH	ACLS, ACHBS

Model gene number	CBDB1 core metabolic genes	195 core metabolic genes	BAV1 core metabolic genes	VS core metabolic genes	CBDB1 annotation	195 annotation	BAV1 annotation	VS annotation	Model protein	Model reaction
223	cbdbA813	DET0833	DehaBAV1_0752	VS737	acetolactate synthase, large subunit	acetolactate synthase, large subunit, biosynthetic type(EC:2.2.1.6,EC:4.1.3.18)	acetolactate synthase, large subunit, biosynthetic type(EC:2.2.1.6)	acetolactate synthase, large subunit, biosynthetic type(EC:2.2.1.6)	IlvBH	ACLS, ACHBS
224	cbdbA815	DET0834	DehaBAV1_0753	VS738	dihydroxy-acid dehydratase	dihydroxy-acid dehydratase(EC:4.2.1.9)	dihydroxy-acid dehydratase(EC:4.2.1.9)	dihydroxy-acid dehydratase(EC:4.2.1.9)	IlvD	DHAD1, DHAD2, DHAD3
225	cbdbA817	DET0836	DehaBAV1_0755	VS740	GMP synthase	GMP synthase(EC:6.3.5.2)	GMP synthase, large subunit	GMP synthase, large subunit	GuaA	GMPS2
226	cbdbA819	DET0838	DehaBAV1_0757	VS742	phosphoribosylamine-glycine ligase	phosphoribosylamine--glycine ligase(EC:6.3.4.13)	(EC:6.3.4.13)	phosphoribosylamine--glycine ligase(EC:6.3.4.13)	PurD	PRAGS
227	cbdbA820	DET0839	DehaBAV1_0758	VS743	phosphoribosylaminoimidazole carboxylase,catalytic subunit	phosphoribosylaminoimidazole carboxylase, catalytic subunit(EC:4.1.1.21)	phosphoribosylaminoimidazole carboxylase, catalytic subunit(EC:4.1.1.21)	phosphoribosylaminoimidazole carboxylase, catalytic subunit(EC:4.1.1.21)	PurE	AIRC
228	cbdbA821	DET0840	DehaBAV1_0759	VS744	adenylosuccinate lyase	adenylosuccinate lyase(EC:4.3.2.2)	adenylosuccinate lyase(EC:4.3.2.2)	adenylosuccinate lyase(EC:4.3.2.2)	PurB	ADSL1, ADSL2
229	cbdbA823	DET0841	DehaBAV1_0760	VS745	phosphoribosylaminoimidazole-succinocarboxamidesynthase	phosphoribosylaminoimidazole-succinocarboxamide synthase(EC:6.3.2.6)	phosphoribosylaminoimidazole-succinocarboxamide synthase(EC:6.3.2.6)	phosphoribosylaminoimidazole-succinocarboxamide synthase(EC:6.3.2.6)	PurC	PRASCS
230	cbdbA824	DET0842	DehaBAV1_0761	VS746	imidazoleglycerol-phosphate dehydratase	imidazoleglycerol-phosphate dehydratase(EC:4.2.1.19)	Imidazoleglycerol-phosphate dehydratase(EC:4.2.1.19)	Imidazoleglycerol-phosphate dehydratase(EC:4.2.1.19)	HisB	IGPDH
231	cbdbA825	DET0843	DehaBAV1_0762	VS747	histidinol-phosphate aminotransferase	histidinol-phosphate aminotransferase(EC:2.6.1.9)	histidinol-phosphate aminotransferase	histidinol-phosphate aminotransferase	HisC	HSTPT, TYRTA, PHETA1

Model gene number	CBDB1 core metabolic genes	195 core metabolic genes	BAV1 core metabolic genes	VS core metabolic genes	CBDB1 annotation	195 annotation	BAV1 annotation	VS annotation	Model protein	Model reaction
232	cbdbA826	DET0844	DehaBAV1_0763	VS748	histidinol dehydrogenase	histidinol dehydrogenase(EC:1.1.1.23)	Histidinol dehydrogenase(EC:1.1.1.23)	Histidinol dehydrogenase(EC:1.1.1.23)	HisD	HISTD
233	cbdbA828	DET0845	DehaBAV1_0764	VS749	probable ATP phosphoribosyltransferase	ATP phosphoribosyltransferase, putaitve	ATP phosphoribosyltransferase, catalytic region	ATP phosphoribosyltransferase, catalytic region	HisG	ATPPRT
234	cbdbA829	DET0846	DehaBAV1_0765	VS750	histidyl-tRNA synthetase	histidyl-tRNA synthetase, putative	Histidine--tRNA ligase(EC:6.1.1.21)	Histidine--tRNA ligase(EC:6.1.1.21)	HisS2	HISTRS
235	cbdbA832	DET0849	DehaBAV1_0768	VS753	biotin-acetyl-CoA-carboxylase ligase	biotin--acetyl-CoA-carboxylase ligase	biotin--acetyl-CoA-carboxylase ligase	biotin--acetyl-CoA-carboxylase ligase	BirA	BACCL
236	cbdbA840	DET0858	DehaBAV1_0776	VS761	oxygen-independent coproporphyrinogen III oxidase	oxygen-independent coproporphyrinogen III oxidase, putative	putative oxygen-independent coproporphyrinogen III oxidase(EC:1.3.99.22)	putative oxygen-independent coproporphyrinogen III oxidase	HemN	CPPPGOAN2
237	cbdbA841	DET0859	DehaBAV1_0777	VS762	GTP-binding protein LepA	GTP-binding protein LepA	GTP-binding protein LepA	GTP-binding protein LepA	CysH	SADT2
238	cbdbA842	DET0860	DehaBAV1_0778	VS763	hydrogenase, EchA subunit	hydrogenase, EchA subunit, putative	NADH dehydrogenase (quinone)(EC:1.6.99.5)	NADH dehydrogenase (quinone)(EC:1.6.99.5)	FrdH	FRHD
239	cbdbA843	DET0861	DehaBAV1_0779	VS764	hydrogenase, EchB subunit	hydrogenase, EchB subunit, putative	respiratory-chain NADH dehydrogenase, subunit 1	respiratory-chain NADH dehydrogenase, subunit 1	FrdH	FRHD
240	cbdbA844	DET0862	DehaBAV1_0780	VS765	hydrogenase, group 4, EchC subunit	hydrogenase, group 4, EchC subunit, putative	NADH ubiquinone oxidoreductase, 20 kDa subunit	NADH ubiquinone oxidoreductase, 20 kDa subunit	FrdH	FRHD
241	cbdbA845	DET0863	DehaBAV1_0781	VS766	hydrogenase subunit HymA	hydrogenase subunit HymA, putative	NADH dehydrogenase (ubiquinone), 24 kDa subunit	NADH dehydrogenase (ubiquinone), 24 kDa subunit	Nadhd5	NADH6, NADH7, NADH4, NADH8

Model gene number	CBDB1 core metabolic genes	195 core metabolic genes	BAV1 core metabolic genes	VS core metabolic genes	CBDB1 annotation	195 annotation	BAV1 annotation	VS annotation	Model protein	Model reaction
242	cbdbA846	DET0729	DehaBAV1_0661	VS767	hydrogenase subunit HymB	[Fe] hydrogenase, HymB subunit, putative	NADH dehydrogenase (quinone)(EC:1.6.99.5)	NADH dehydrogenase (quinone)(EC:1.6.99.5)	Nadhd5	NADH6, NADH7, NADH4, NADH8
243	cbdbA848	DET0865	DehaBAV1_0783	VS768	hydrogenase subunit HymC	hydrogenase subunit HymC, putative	ferredoxin	ferredoxin	Nadphd, Nadhd5, Fdx	NADH6, NADPH12, NADH7, NADH4, FRHD, NADH8
244	cbdbA849	DET0866	DehaBAV1_0784	VS769	putative hydrogenase subunit	hydrogenase subunit, putative	FAD-dependent pyridine nucleotide-disulphide oxidoreductase	FAD-dependent pyridine nucleotide-disulphide oxidoreductase	GltB	GLUSy
245	cbdbA850	DET0867	DehaBAV1_0785	VS770	hydrogenase, group 4, EchE subunit	hydrogenase, group 4, EchE subunit, putative	NADH-ubiquinone oxidoreductase, chain 49kDa	NADH dehydrogenase (ubiquinone)(EC:1.6.5.3)	FrdH	FRHD
246	cbdbA851	DET0868	DehaBAV1_0786	VS771	hydrogenase, EchF subunit	hydrogenase, EchF subunit, putative	4Fe-4S ferredoxin, iron sulfur binding domain protein	4Fe-4S ferredoxin, iron-sulfur binding domain protein	FrdH	FRHD
247	cbdbA853	DET0869	DehaBAV1_0787	VS772	lysyl-tRNA synthetase-related protein	lysyl-tRNA synthetase-related protein	tRNA synthetase, class II (D, K and N)	tRNA synthetase, class II (D, K and N)	LysS	LYSTRS
248	cbdbA859	DET0908	DehaBAV1_0793	VS780	arsenical pump membrane protein	arsenical pump membrane protein, putative	Arsenical pump membrane protein	Arsenical pump membrane protein	ArsB	ARSt1
249	cbdbA874	DET0923	DehaBAV1_0807	VS794	proton-translocating NADH-quinone oxidoreductase, A subunit	proton-translocating NADH-quinone oxidoreductase, A subunit	NADH-ubiquinone/plastoquinone oxidoreductase, chain 3	NADH-ubiquinone/plastoquinone oxidoreductase, chain 3	Nadhd5	NADH6, NADH7, NADH4, NADH8

Model gene number	CBDB1 core metabolic genes	195 core metabolic genes	BAV1 core metabolic genes	VS core metabolic genes	CBDB1 annotation	195 annotation	BAV1 annotation	VS annotation	Model protein	Model reaction
250	cbdbA875	DET0924	DehaBAV1_0808	VS795	proton-translocating NADH-quinone oxidoreductase, B subunit	proton-translocating NADH-quinone oxidoreductase, B subunit(EC:1.6.99.3)	NADH-quinone oxidoreductase, B subunit(EC:1.6.99.3,EC:1.6.99.5)	NADH-quinone oxidoreductase, B subunit(EC:1.6.99.3)	Nadhd5	NADH6, NADH7, NADH4, NADH8
251	cbdbA876	DET0925	DehaBAV1_0809	VS796	proton-translocating NADH-quinone oxidoreductase, C subunit	proton-translocating NADH-quinone oxidoreductase, C subunit	NADH dehydrogenase (ubiquinone), 30 kDa subunit	NADH dehydrogenase (ubiquinone), 30 kDa subunit	Nadhd5	NADH6, NADH7, NADH4, NADH8
252	cbdbA877	DET0926	DehaBAV1_0810	VS797	proton-translocating NADH-quinone oxidoreductase, D subunit	proton-translocating NADH-quinone oxidoreductase, D subunit	NADH dehydrogenase I, D subunit(EC:1.6.99.5)	NADH dehydrogenase I, D subunit(EC:1.6.5.3)	Nadhd5	NADH6, NADH7, NADH4, NADH8
253	cbdbA879	DET0927	DehaBAV1_0811	VS798	proton-translocating NADH-quinone oxidoreductase, H subunit	proton-translocating NADH-quinone oxidoreductase, H subunit	NADH dehydrogenase (quinone)(EC:1.6.99.5)	respiratory-chain NADH dehydrogenase, subunit 1	Nadhd5	NADH6, NADH7, NADH4, NADH8
254	cbdbA880	DET0928	DehaBAV1_0812	VS799	proton-translocating NADH-quinone oxidoreductase, I subunit	proton-translocating NADH-quinone oxidoreductase, I subunit	NADH-quinone oxidoreductase, chain I	NADH-quinone oxidoreductase, chain I	Nadhd5	NADH6, NADH7, NADH4, NADH8
255	cbdbA881	DET0929	DehaBAV1_0813	VS800	proton-translocating NADH-quinone oxidoreductase, J subunit	proton-translocating NADH-quinone oxidoreductase, J subunit	NADH-ubiquinone/plastoquinone oxidoreductase, chain 6	NADH-ubiquinone/plastoquinone oxidoreductase, chain 6	Nadhd5	NADH6, NADH7, NADH4, NADH8
256	cbdbA882	DET0930	DehaBAV1_0814	VS801	proton-translocating NADH-quinone oxidoreductase, K subunit	proton-translocating NADH-quinone oxidoreductase, K subunit	NADH-ubiquinone oxidoreductase, chain 4L	NADH-ubiquinone oxidoreductase, chain 4L	Nadhd5	NADH6, NADH7, NADH4, NADH8

Model gene number	CBDB1 core metabolic genes	195 core metabolic genes	BAV1 core metabolic genes	VS core metabolic genes	CBDB1 annotation	195 annotation	BAV1 annotation	VS annotation	Model protein	Model reaction
257	cbdbA883	DET0931	DehaBAV1_ 0815	VS802	proton-translocating NADH-quinone oxidoreductase, L subunit	proton-translocating NADH-quinone oxidoreductase, L subunit	proton-translocating NADH-quinone oxidoreductase, chain L(EC:1.6.99.5)	proton-translocating NADH-quinone oxidoreductase, chain L(EC:1.6.99.5)	Nadhd5	NADH6, NADH7, NADH4, NADH8
258	cbdbA884	DET0932	DehaBAV1_ 0816	VS803	proton-translocating NADH-quinone oxidoreductase, M subunit	proton-translocating NADH-quinone oxidoreductase, M subunit	proton-translocating NADH-quinone oxidoreductase, chain M(EC:1.6.99.5)	proton-translocating NADH-quinone oxidoreductase, chain M(EC:1.6.99.5)	Nadhd5	NADH6, NADH7, NADH4, NADH8
259	cbdbA885	DET0933	DehaBAV1_ 0817	VS804	proton-translocating NADH-quinone oxidoreductase, N subunit	proton-translocating NADH-quinone oxidoreductase, N subunit	proton-translocating NADH-quinone oxidoreductase, chain N(EC:1.6.99.5)	proton-translocating NADH-quinone oxidoreductase, chain N(EC:1.6.99.5)	Nadhd5	NADH6, NADH7, NADH4, NADH8
260	cbdbA890	DET0936	DehaBAV1_ 0820	VS807	cobyric acid synthase CobQ	cobyric acid synthase CobQ	cobyric acid synthase CobQ	cobyric acid synthase CobQ	CobQ	CPC3MT, ADCOBHEXS
261	cbdbA892	DET0938	DehaBAV1_ 0822	VS809	branched-chain amino acid ABC transporter,ATP-binding protein	high-affinity branched-chain amino acid ABC transporter, ATP binding protein	ABC transporter related	ABC transporter related	BraB	VALabc, LEUabc, ILEabc
262	cbdbA894	DET0940	DehaBAV1_ 0824	VS811	acyl-CoA synthetase (AMP-forming) / AMP-acid ligase	AMP-binding enzyme	Phenylacetate--CoA ligase(EC:6.2.1.30)	Phenylacetate--CoA ligase(EC:6.2.1.30)	PaaK	PACCOAL
263	cbdbA895	DET0941	DehaBAV1_ 0825	VS812	branched chain amino acid ABC transporter,ATP-binding protein	high-affinity branched chain amino acid ABC transporter, ATP binding protein	ABC transporter related	ABC transporter related	AzlCD	VALabc, ILEabc, LEUabc

Model gene number	CBDB1 core metabolic genes	195 core metabolic genes	BAV1 core metabolic genes	VS core metabolic genes	CBDB1 annotation	195 annotation	BAV1 annotation	VS annotation	Model protein	Model reaction
264	cbdbA896	DET0942	DehaBAV1_0826	VS813	putative branched-chain amino acid ABC transporter,periplasmic amino acid-binding protein	lipoprotein, putative	ABC-type branched-chain amino acid transport systems periplasmic component-like protein	putative branched-chain amino acid ABC transporter,periplasmic amino acid-binding protein	AzlCD	VALabc, ILEabc, LEUabc
265	cbdbA897	DET0943	DehaBAV1_0827	VS814	putative branched-chain amino acid ABC transporter,periplasmic amino acid-binding protein	lipoprotein, putative	ABC-type branched-chain amino acid transport systems periplasmic component-like protein	lipoprotein, putative	AzlCD	VALabc, ILEabc, LEUabc
266	cbdbA898	DET0944	DehaBAV1_0828	VS815	branched-chain amino acid ABC transporter,permease protein	high-affinity branched-chain amino acid ABC transporter, permease protein, putative	inner-membrane translocator	inner-membrane translocator	AzlCD	VALabc, ILEabc, LEUabc
267	cbdbA899	DET0945	DehaBAV1_0829	VS816	branched-chain amino acid ABC transporter,permease protein	high-affinity branched-chain amino acid ABC transporter, permease protein, putative	inner-membrane translocator	inner-membrane translocator	AzlCD	VALabc, ILEabc, LEUabc
268	cbdbA901	DET0946	DehaBAV1_0830	VS817	acyl-CoA synthetase (AMP-forming) / AMP-acid ligase	AMP-binding protein	AMP-dependent synthetase and ligase	AMP-dependent synthetase and ligase	FadD	FACOAL181, FACOAL140, FACOAL160, FACOAL120, FACOAL170(ISO), FACOAL180, FACOAL200
269	cbdbA902	DET0947	DehaBAV1_0831	VS818	indolepyruvate ferredoxin oxidoreductase, alpha subunit	indolepyruvate ferredoxin oxidoreductase, alpha subunit	thiamine pyrophosphate enzyme domain protein TPP-binding	thiamine pyrophosphate enzyme domain protein TPP-binding	IorAB1	IOR2, IOR3, IOR

Model gene number	CBDB1 core metabolic genes	195 core metabolic genes	BAV1 core metabolic genes	VS core metabolic genes	CBDB1 annotation	195 annotation	BAV1 annotation	VS annotation	Model protein	Model reaction
270	cbdbA903	DET0948	DehaBAV1_0832	VS819	indolepyruvate ferredoxin oxidoreductase, beta subunit	indolepyruvate ferredoxin oxidoreductase, beta subunit	Indolepyruvate ferredoxin oxidoreductase(EC:1.2.7.8)	Indolepyruvate ferredoxin oxidoreductase(EC:1.2.7.8)	IorAB1	IOR2, IOR3, IOR
271	cbdbA908	DET0953	DehaBAV1_0837	VS824	copper-translocating P-type ATPase	copper-translocating P-type ATPase(EC:3.6.3.4)	heavy metal translocating P-type ATPase(EC:3.6.3.4)	copper-translocating P-type ATPase(EC:3.6.3.4)	Cudp	Cuabc
272	cbdbA912	DET0956	DehaBAV1_0840	VS827	superoxide dismutase	superoxide dismutase(EC:1.15.1.1)	manganese and iron superoxide dismutase	manganese and iron superoxide dismutase	SodA	SOD
273	cbdbA916	DET0960	DehaBAV1_0851	VS833	imidazoleglycerol phosphate synthase, cyclase subunit	imidazoleglycerol phosphate synthase, cyclase subunit, putative	histidine biosynthesis protein	histidine biosynthesis	HisF	IG3PS
274	cbdbA921	DET0963	DehaBAV1_0854	VS836	3-oxoacyl-[acyl-carrier-protein] synthase II	3-oxoacyl-[acyl-carrier-protein] synthase II(EC:2.3.1.41)	beta-ketoacyl synthase	beta-ketoacyl synthase	FabF	MCMAT7, AACPAT, MCMAT9, MCMAT4, MCMAT5, ACMAT1, MCMAT2, MCMAT8, MCMAT3, MCMAT6
275	cbdbA933	DET0971	DehaBAV1_0862	VS844	dihydrodipicolinate reductase	dihydrodipicolinate reductase(EC:1.3.1.26)	Dihydrodipicolinate reductase(EC:1.3.1.26)	Dihydrodipicolinate reductase(EC:1.3.1.26)	DapB	DHDPRy
276	cbdbA934	DET0972	DehaBAV1_0863	VS845	aspartate-semialdehyde dehydrogenase	aspartate-semialdehyde dehydrogenase(EC:1.2.1.11)	aspartate-semialdehyde dehydrogenase(EC:1.2.1.11)	aspartate-semialdehyde dehydrogenase(EC:1.2.1.11)	Asd	ASAD
277	cbdbA935	DET0973	DehaBAV1_0864	VS846	dihydrodipicolinate synthase	dihydrodipicolinate synthase(EC:4.2.1.52)	dihydrodipicolinate synthase(EC:4.2.1.52)	dihydrodipicolinate synthase(EC:4.2.1.52)	DapA	DHDPS
278	cbdbA939	DET0976	DehaBAV1_0867	VS849	adenylosuccinate synthetase	adenylosuccinate synthetase(EC:6.3.4.4)	Adenylosuccinate synthase(EC:6.3.4.4)	Adenylosuccinate synthase(EC:6.3.4.4)	PurA	ADSS

Model gene number	CBDB1 core metabolic genes	195 core metabolic genes	BAV1 core metabolic genes	VS core metabolic genes	CBDB1 annotation	195 annotation	BAV1 annotation	VS annotation	Model protein	Model reaction
279	cbdbA940	DET0977	DehaBAV1_0868	VS850	CDP-alcohol phosphatidyltransferase family protein	CDP-alcohol phosphatidyltransferase family protein	CDP-alcohol phosphatidyltransferase	CDP-alcohol phosphatidyltransferase	PssA1, Cls, PgsA	CDGPT, CLPNS, CDPDSP
280	cbdbA942	DET0978	DehaBAV1_0869	VS851	glycosyl transferase, group 1 family protein	glycosyl transferase, group 1 family protein	glycosyl transferase, group 1	glycosyl transferase, group 1	GlgA1	GLCS1
281	cbdbA945	DET0981	DehaBAV1_0872	VS853	tRNA pseudouridine synthase B	tRNA pseudouridine synthase B(EC:4.2.1.70)	tRNA pseudouridine synthase B	tRNA pseudouridine synthase B(EC:4.2.1.70)	RluB	PSUDS
282	cbdbA947	DET0983	DehaBAV1_0874	VS855	translation initiation factor IF-2	translation initiation factor IF-2	translation initiation factor IF-2	translation initiation factor IF-2	CysH	SADT2
283	cbdbA960	DET0997	DehaBAV1_0888	VS869	translation elongation factor Tu	translation elongation factor Tu	translation elongation factor Tu	translation elongation factor Tu	CysH	SADT2
284	cbdbA963	DET1002	DehaBAV1_0890	VS874	glycosyl transferase, group 1 family protein	glycosyl transferase, group 1 family protein	glycosyl transferase, group 1	glycosyl transferase, group 1	GlgA1	GLCS1
285	cbdbA988	DET1015	DehaBAV1_0902	VS886	acyl-CoA synthetase (AMP-forming) / AMP-acid ligase	phenylacetate-CoA ligase(EC:6.2.1.30)	Phenylacetate--CoA ligase(EC:6.2.1.30)	Phenylacetate--CoA ligase(EC:6.2.1.30)	PaaK	PACCOAL
286	cbdbA1006	DET1033	DehaBAV1_0916	VS904	acyl-CoA synthetase (AMP-forming) / AMP-acid ligase	long-chain-fatty-acid--CoA ligase, putative	AMP-dependent synthetase and ligase	AMP-dependent synthetase and ligase	FadD	FACOAL181, FACOAL140, FACOAL160, FACOAL120, FACOAL170(ISO), FACOAL180, FACOAL200
287	cbdbA1008	DET1035	DehaBAV1_0917	VS905	pyridoxal-phosphate dependent TrpB-like enzyme	tryptophan synthase subunit beta	pyridoxal-phosphate dependent TrpB-like enzyme	pyridoxal-phosphate dependent TrpB-like enzyme	TrpB	TRPS1

Model gene number	CBDB1 core metabolic genes	195 core metabolic genes	BAV1 core metabolic genes	VS core metabolic genes	CBDB1 annotation	195 annotation	BAV1 annotation	VS annotation	Model protein	Model reaction
288	cbdbA1012	DET1038	DehaBAV1_0920	VS908	isoleucyl-tRNA synthetase	isoleucyl-tRNA synthetase(EC:6.1.1.5)	isoleucyl-tRNA synthetase	isoleucyl-tRNA synthetase	IleS	ILETRS
289	cbdbA1049	DET1122	DehaBAV1_0951	VS940	glutamine-dependent NAD(+) synthetase	glutamine-dependent NAD(+) synthetase(EC:6.3.5.1)	NAD+ synthetase(EC:6.3.5.1)	NAD+ synthetase(EC:6.3.5.1)	NadE2	NADS2
290	cbdbA1050	DET1123	DehaBAV1_0952	VS941	glutamine synthetase, type I	glutamine synthetase, type I(EC:6.3.1.2)	glutamine synthetase, type I	glutamine synthetase, type I(EC:6.3.1.2)	GlnA	GLNS
291	cbdbA1052	DET1125	DehaBAV1_0954	VS943	ammonium transporter	ammonium transporter	ammonium transporter	ammonium transporter	AmtB	NH4t3, NH4t
292	cbdbA1054	DET1127	DehaBAV1_0956	VS945	conserved hypothetical protein	hypothetical protein	glutamate synthase, alpha subunit domain protein	glutamate synthase, alpha subunit domain protein	GltBD	GLUSy
293	cbdbA1055	DET1128	DehaBAV1_0957	VS946	glutamate synthase, alpha subunit	glutamate synthase, alpha subunit, putative	Glutamate synthase (NADPH)(EC:1.4.1.13)	Glutamate synthase (NADPH)(EC:1.4.1.13)	GltBD	GLUSy
294	cbdbA1057	DET1129	DehaBAV1_0958	VS947	imidazoleglycerol phosphate synthase, cyclase subunit	imidazoleglycerol phosphate synthase, cyclase subunit	imidazoleglycerol phosphate synthase, cyclase subunit	imidazoleglycerol phosphate synthase, cyclase subunit	HisF	IG3PS
295	cbdbA1058	DET1130	DehaBAV1_0959	VS948	glutamine amidotransferase, class II	glutamine amidotransferase, class II	glutamine amidotransferase, class-II	glutamine amidotransferase, class-II	GltB	GLUSy
296	cbdbA1059	DET1131	DehaBAV1_0960	VS949	pyridine nucleotide-disulfide oxidoreductase family protein	pyridine nucleotide-disulfide oxidoreductase family protein	FAD-dependent pyridine nucleotide-disulphide oxidoreductase	FAD-dependent pyridine nucleotide-disulphide oxidoreductase	GltAB, NasDE	GLUSx, NTRIRy, NTRIRx

Model gene number	CBDB1 core metabolic genes	195 core metabolic genes	BAV1 core metabolic genes	VS core metabolic genes	CBDB1 annotation	195 annotation	BAV1 annotation	VS annotation	Model protein	Model reaction
297	cbdbA1060	DET1132	DehaBAV1_0961	VS950	imidazole glycerol phosphate synthase,glutamineamidotransferase subunit	imidazole glycerol phosphate synthase, glutamine amidotransferase subunit(EC:2.4.2.-)	imidazole glycerol phosphate synthase, glutamine amidotransferase subunit	imidazole glycerol phosphate synthase, glutamine amidotransferase subunit	HisF	IG3PS
298	cbdbA1061	DET1133	DehaBAV1_0962	VS951	iron-sulfur cluster-binding protein	iron-sulfur cluster-binding protein	4Fe-4S ferredoxin, iron sulfur binding domain protein	4Fe-4S ferredoxin, iron-sulfur binding domain protein	GltAB	GLUSx
299	cbdbA1063	DET1135	DehaBAV1_0964	VS953	putative O-acetylserine(thiol)-lyase-A related protein	cysteine synthase A(EC:2.5.1.47,EC:4.2.99.8)	cysteine synthase A(EC:2.5.1.47)	cysteine synthase A(EC:2.5.1.47)	CysKec, IlvA, XylA	CYSS, XYLI2i
300	cbdbA1067	DET1138	DehaBAV1_0967	VS956	cobalamin biosynthesis protein	cobalamin biosynthesis protein CobD	cobalamin biosynthesis protein CobD	cobalamin biosynthesis protein CobD	CbiD	ADCOBAS, ADCPS2
301	cbdbA1069	DET1139	DehaBAV1_0968	VS957	cob(I)alamin adenosyltransferase	cob(I)alamin adenosyltransferase(EC:2.5.1.17)	ATP:corrinoid adenosyltransferase BtuR/CobO/CobP	ATP:corrinoid adenosyltransferase BtuR/CobO/CobP	CobA	CBLATr, CYRDAAT, CBIAT
302	cbdbA1070	DET1140	DehaBAV1_0969	VS958	carbon-nitrogen hydrolase family protein	carbon-nitrogen hydrolase family protein	Nitrilase/cyanide hydratase and apolipoprotein N-acyltransferase	Nitrilase/cyanide hydratase and apolipoprotein N-acyltransferase	NadE	NADS1
303	cbdbA1087	DET1180	DehaBAV1_0985	VS964	ABC transporter, ATP-binding/permease protein	ABC transporter, ATP binding/permease protein	ABC transporter, transmembrane region	ABC transporter, transmembrane region	ThrB	HSK
304	cbdbA1092	DET1171	DehaBAV1_0988	VS1360	putative reductive dehalogenase	reductive dehalogenase, putative	4Fe-4S ferredoxin, iron sulfur binding domain protein	4Fe-4S ferredoxin, iron-sulfur binding domain protein	RdhA	RDR1, RDR2
305	cbdbA1094	DET1170	DehaBAV1_0989	VS1359	putative reductive dehalogenase anchoring protein	reductive dehalogenase anchoring protein, putative	hypothetical protein	putative reductive dehalogenase anchoring protein	RdhB	RDR1, RDR2

Model gene number	CBDB1 core metabolic genes	195 core metabolic genes	BAV1 core metabolic genes	VS core metabolic genes	CBDB1 annotation	195 annotation	BAV1 annotation	VS annotation	Model protein	Model reaction
306	cbdbA1098	DET1184	DehaBAV1_0995	VS967	BioY family protein	BioY family protein	BioY protein	BioY protein	BioBec	BTS
307	cbdbA1102	DET1187	DehaBAV1_0998	VS970	6,7-dimethyl-8-ribityllumazine synthase	6,7-dimethyl-8-ribityllumazine synthase(EC:2.5.1.9)	Riboflavin synthase(EC:2.5.1.9)	Riboflavin synthase(EC:2.5.1.9)	RibEH	RBFSa, RBFSb
308	cbdbA1103	DET1188	DehaBAV1_0999	VS971	3,4-dihydroxy-2-butanone 4-phosphatesynthase	3,4-dihydroxy-2-butanone 4-phosphate synthase/GTP cyclohydrolase II	GTP cyclohydrolase II(EC:3.5.4.25)	GTP cyclohydrolase II(EC:3.5.4.25)	RibA	DB4PS, GTPCII
309	cbdbA1104	DET1189	DehaBAV1_1000	VS972	riboflavin synthase, alpha subunit	riboflavin synthase subunit alpha(EC:2.5.1.9)	riboflavin synthase, alpha subunit	riboflavin synthase, alpha subunit	RibEH	RBFSa, RBFSb
310	cbdbA1105	DET1190	DehaBAV1_1001	VS973	riboflavin biosynthesis protein RibD	riboflavin biosynthesis protein RibD	riboflavin biosynthesis protein RibD(EC:1.1.1.193,EC:3.5.4.26)	riboflavin biosynthesis protein RibD(EC:1.1.1.193,EC:3.5.4.26)	RibD	APRAUR, DHPPDA2
311	cbdbA1108	DET1193	DehaBAV1_1004	VS976	orotate phosphoribosyltransferase	orotate phosphoribosyltransferase(EC:2.4.2.10)	orotate phosphoribosyltransferase	orotate phosphoribosyltransferase	Apt, PyrE	ORPT, ADPT
312	cbdbA1111	DET1196	DehaBAV1_1007	VS979	ATP-dependent DNA helicase PcrA	ATP-dependent DNA helicase PcrA(EC:3.6.1.-)	UvrD/REP helicase	UvrD/REP helicase	NtpA, MurAA	DNMPPA, UAGCVT, DNTPPA
313	cbdbA1112	DET1198	DehaBAV1_1008	VS981	pyrimidine operon regulatory protein	pyrimidine regulatory protein PyrR	phosphoribosyltransferase	Uracil phosphoribosyltransferase(EC:2.4.2.9)	Upp2	UPPRT
314	cbdbA1113	DET1199	DehaBAV1_1009	VS982	aspartate carbamoyltransferase	aspartate carbamoyltransferase (EC:2.1.3.2)	aspartate carbamoyltransferase(EC:2.1.3.2)	aspartate carbamoyltransferase(EC:2.1.3.2)	PyrB	ASPCT
315	cbdbA1114	DET1200	DehaBAV1_1010	VS983	dihydroorotase, multifunctional complex type	dihydroorotase(EC:3.5.2.3)	dihydroorotase, multifunctional complex type(EC:3.5.2.3)	dihydroorotase, multifunctional complex type(EC:3.5.2.3)	PyrC	DHORTS

Model gene number	CBDB1 core metabolic genes	195 core metabolic genes	BAV1 core metabolic genes	VS core metabolic genes	CBDB1 annotation	195 annotation	BAV1 annotation	VS annotation	Model protein	Model reaction
316	cbdbA1117	DET1201	DehaBAV1_1011	VS984	carbamoyl-phosphate synthase, small subunit	carbamoyl-phosphate synthase small subunit(EC:6.3.5.5)	carbamoyl-phosphate synthase, small subunit(EC:6.3.5.5)	carbamoyl-phosphate synthase, small subunit(EC:6.3.5.5)	CarAB	CBPSr
317	cbdbA1118	DET1202	DehaBAV1_1012	VS985	carbamoyl-phosphate synthase, large subunit	carbamoyl-phosphate synthase, large subunit(EC:6.3.5.5)	carbamoyl-phosphate synthase, large subunit	carbamoyl-phosphate synthase, large subunit	CarAB	CBPSr
318	cbdbA1119	DET1203	DehaBAV1_1013	VS986	dihydroorotate dehydrogenase, electron transfersubunit	dihydroorotate dehydrogenase, electron transfer subunit	oxidoreductase FAD/NAD(P)-binding domain protein	oxidoreductase FAD/NAD(P)-binding domain protein	PyrK	DHORDf
319	cbdbA1120	DET1204	DehaBAV1_1014	VS987	GTP cyclohydrolase I	GTP cyclohydrolase I(EC:3.5.4.16)	GTP cyclohydrolase I(EC:3.5.4.16)	GTP cyclohydrolase I(EC:3.5.4.16)	FolE	GTPCI
320	cbdbA1122	DET1205	DehaBAV1_1015	VS988	threonine synthase	threonine synthase(EC:4.2.99.2)	threonine synthase	threonine synthase	ThrC, IlvA	SERD_Lr, THRS, THRD_L
321	cbdbA1123	DET1206	DehaBAV1_1016	VS989	homoserine dehydrogenase	homoserine dehydrogenase(EC:1.1.1.3)	Homoserine dehydrogenase(EC:1.1.1.3)	Homoserine dehydrogenase(EC:1.1.1.3)	Hom	HSDy
322	cbdbA1125	DET1208	DehaBAV1_1018	VS991	nucleotidyl transferase family protein	nucleotidyltransferase family protein	Nucleotidyl transferase	Nucleotidyl transferase	Mpg, GlgC	MAN1PT1, GLGC
323	cbdbA1126	DET1209	DehaBAV1_1019	VS992	acetyl-CoA synthetase	acetyl-CoA synthetase(EC:6.2.1.1)	acetate--CoA ligase(EC:6.2.1.1)	acetate--CoA ligase(EC:6.2.1.1)	Acs	ACS
324	cbdbA1136	DET1218	DehaBAV1_1028	VS1001	HAD-superfamily hydrolase, subfamily IIB	HAD-superfamily hydrolase, subfamily IIB	Cof-like hydrolase	Cof-like hydrolase	SerB	PSP_L
325	cbdbA1140	DET1223	DehaBAV1_1032	VS1005	fumarylacetoacetate hydrolase family protein	fumarylacetoacetate hydrolase family protein	5-carboxymethyl-2-hydroxymuconate Delta-isomerase(EC:5.3.3.10)	5-oxopent-3-ene-1,2,5-tricarboxylate decarboxylase(EC:4.1.1.68)	P_3.7.1.5	APYRH

Model gene number	CBDB1 core metabolic genes	195 core metabolic genes	BAV1 core metabolic genes	VS core metabolic genes	CBDB1 annotation	195 annotation	BAV1 annotation	VS annotation	Model protein	Model reaction
326	cbdbA1141	DET1224	DehaBAV1_1033	VS1006	cob(I)alamin adenosyltransferase	cob(I)alamin adenosyltransferase(EC:2.5.1.17)	ATP:corrinoid adenosyltransferase BtuR/CobO/CobP	ATP:corrinoid adenosyltransferase BtuR/CobO/CobP	CobA	CBLATr, CYRDAAT, CBIAT
327	cbdbA1142	DET1225	DehaBAV1_1034	VS1007	conserved hypothetical protein	hypothetical protein	protein of unknown function DUF100	protein of unknown function DUF100	Fbp	FBP
328	cbdbA1143	DET1226	DehaBAV1_1035	VS1008	non-canonical purine NTP pyrophosphatase, RdgB	non-canonical purine NTP pyrophosphatase, RdgB/HAM1 family	non-canonical purine NTP pyrophosphatase, rdgB/HAM1 family	non-canonical purine NTP pyrophosphatase, rdgB/HAM1 family	IxpP	XTPASE, ITPASE
329	cbdbA1158	DET1237	DehaBAV1_1052	VS1023	formyltetrahydrofolate deformylase	formyltetrahydrofolate deformylase(EC:3.5.1.10)	formyltetrahydrofolate deformylase(EC:3.5.1.10)	formyltetrahydrofolate deformylase(EC:3.5.1.10)	PurUec, PurN	FTHFD, GARFT
330	cbdbA1161	DET1241	DehaBAV1_1055	VS1027	peptide methionine sulfoxide reductase MsrA	peptide methionine sulfoxide reductase MsrA(EC:1.8.4.6)	peptide methionine sulfoxide reductase	peptide methionine sulfoxide reductase	MsrA	METSR-S2, METSR-S1
331	cbdbA1179	DET1256	DehaBAV1_1067	VS1039	arginine biosynthesis bifunctional protein ArgJ	bifunctional ornithine acetyltransferase/N-acetylglutamate synthase protein(EC:2.3.1.35)	arginine biosynthesis bifunctional protein ArgJ(EC:2.3.1.1,EC:2.3.1.35)	arginine biosynthesis bifunctional protein ArgJ(EC:2.3.1.35)	ArgJ	ORNTAC, ACGS
332	cbdbA1180	DET1257	DehaBAV1_1068	VS1040	acetylglutamate kinase	acetylglutamate kinase(EC:2.7.2.8)	acetylglutamate kinase	acetylglutamate kinase	ArgB1	ACGK
333	cbdbA1181	DET1258	DehaBAV1_1069	VS1041	acetylornithine aminotransferase	acetylornithine aminotransferase(EC:2.6.1.11)	acetylornithine and succinylornithine aminotransferase(EC:2.6.1.11)	acetylornithine and succinylornithine aminotransferase(EC:2.6.1.11)	GabT, PuuT, ArgD, BioA	AMAOT, ACOTA, SDPTA, ORNTA, ABTA
334	cbdbA1183	DET1260	DehaBAV1_1071	VS1043	argininosuccinate synthase	argininosuccinate synthase(EC:6.3.4.5)	Argininosuccinate synthase(EC:6.3.4.5)	Argininosuccinate synthase(EC:6.3.4.5)	ArgG	ARGSS

Model gene number	CBDB1 core metabolic genes	195 core metabolic genes	BAV1 core metabolic genes	VS core metabolic genes	CBDB1 annotation	195 annotation	BAV1 annotation	VS annotation	Model protein	Model reaction
335	cbdbA1185	DET1261	DehaBAV1_1072	VS1044	argininosuccinate lyase	argininosuccinate lyase(EC:4.3.2.1)	argininosuccinate lyase(EC:4.3.2.1)	argininosuccinate lyase(EC:4.3.2.1)	ArgH, AspA	ASPT, ARGSL
336	cbdbA1194	DET1268	DehaBAV1_1079	VS1051	ATP-dependent DNA helicase RecG	ATP-dependent DNA helicase RecG(EC:3.6.1.-)	ATP-dependent DNA helicase RecG	ATP-dependent DNA helicase RecG	NtpA	DNMPPA, DNTPPA
337	cbdbA1196	DET1270	DehaBAV1_1081	VS1053	arginyl-tRNA synthetase	arginyl-tRNA synthetase(EC:6.1.1.19)	arginyl-tRNA synthetase(EC:6.1.1.19)	arginyl-tRNA synthetase(EC:6.1.1.19)	ArgS	ARGTRS
338	cbdbA1199	DET1272	DehaBAV1_1083	VS1055	putative enoyl-(acyl-carrier-protein) reductase	enoyl-(acyl-carrier-protein) reductase, putative	Enoyl-(acyl-carrier-protein) reductase (NADH)(EC:1.3.1.9)	Enoyl-(acyl-carrier-protein) reductase (NADH)(EC:1.3.1.9)	FabI	OCDMAT8, ICSMAT9, DDMAT5, HEMAT2, DEMAT4, KAS17, OCMAT3, KAS11, BTMAT1, TDMAT6, HDMAT7
339	cbdbA1204	DET1276	DehaBAV1_1087	VS1059	malonyl CoA-acyl carrier protein transacylase	malonyl CoA-acyl carrier protein transacylase(EC:2.3.1.39)	malonyl CoA-acyl carrier protein transacylase	malonyl CoA-acyl carrier protein transacylase	FatB, FabD, FabZ	HOCHL3, HICHL9, FA120ACPH, MACPMT, HODHL8n, FA180ACPH, HBUHL1, HHDHL7, HHYHL2, HDEHL4, HTDHL6, HDDHL5, FA200ACPH, FA160ACPH, FA140ACPH

Model gene number	CBDB1 core metabolic genes	195 core metabolic genes	BAV1 core metabolic genes	VS core metabolic genes	CBDB1 annotation	195 annotation	BAV1 annotation	VS annotation	Model protein	Model reaction
340	cbdbA1205	DET1277	DehaBAV1_ 1088	VS1060	3-oxoacyl-acyl carrier protein reductase	3-oxoacyl-acyl carrier protein reductase(EC:1.1.1.100)	3-oxoacyl-(acyl-carrier-protein) reductase(EC:1.1.1.100)	3-oxoacyl-(acyl-carrier-protein) reductase(EC:1.1.1.100)	FabG1	HDER4, HBUR1, HTDR6, HDDR5, HICR9, HOCR3, HHYR2, HHDR7, HODR8_#1
341	cbdbA1212	DET1282	DehaBAV1_ 1093	VS1065	glutamate 5-kinase	gamma-glutamyl kinase(EC:2.7.2.11)	glutamate 5-kinase(EC:2.7.2.11)	glutamate 5-kinase(EC:2.7.2.11)	ProB	GLU5K
342	cbdbA1224	DET1288	DehaBAV1_ 1099	VS1071	gamma-glutamyl phosphate reductase	gamma-glutamyl phosphate reductase(EC:1.2.1.41)	gamma-glutamyl phosphate reductase(EC:1.2.1.41)	gamma-glutamyl phosphate reductase(EC:1.2.1.41)	ProA, AldH	ALDD8b, G5SD
343	cbdbA1237	DET1298	DehaBAV1_ 1109	VS1081	GTP-binding protein TypA	GTP-binding protein TypA	small GTP-binding protein	small GTP-binding protein	CysH	SADT2
344	cbdbA1238	DET1299	DehaBAV1_ 1110	VS1082	major facilitator family transporter	major facilitator family transporter	General substrate transporter	General substrate transporter	ProPec	GLYBt6, PROt6
345	cbdbA1268	DET1323	DehaBAV1_ 1134	VS1105	dephospho-CoA kinase	dephospho-CoA kinase(EC:2.7.1.24)	dephospho-CoA kinase(EC:2.7.1.24)	dephospho-CoA kinase(EC:2.7.1.24)	CoaE	DPCOAK
346	cbdbA1269	DET1324	DehaBAV1_ 1135	VS1106	conserved hypothetical protein	hypothetical protein	NAD-dependent epimerase/dehydratas e	NAD-dependent epimerase/dehydrat ase	GalE	UDPG4E
347	cbdbA1276	DET1329	DehaBAV1_ 1140	VS1111	phosphoribosyl-AMP cyclohydrolase	phosphoribosyl-AMP cyclohydrolase(EC:3.5.4.19)	Phosphoribosyl-AMP cyclohydrolase(EC:3.5.4.19)	Phosphoribosyl-AMP cyclohydrolase(EC:3.5.4.19)	HisI_N	PRATPP, PRAMPC
348	cbdbA1278	DET1331	DehaBAV1_ 1142	VS1113	phosphoribosylformim ino-5-aminoimidazolecarbox amide ribotide isomerase	phosphoribosylformi mino-5-aminoimidazole carboxamide ribotide isomerase(EC:5.3.1.16)	phosphoribosylformimi no-5-aminoimidazole carboxamide ribotide isomerase(EC:5.3.1.16)	phosphoribosylform imino-5-aminoimidazole carboxamide ribotide isomerase(EC:5.3.1.16)	HisA	PRMICI

Model gene number	CBDB1 core metabolic genes	195 core metabolic genes	BAV1 core metabolic genes	VS core metabolic genes	CBDB1 annotation	195 annotation	BAV1 annotation	VS annotation	Model protein	Model reaction
349	cbdbA1282	DET1334	DehaBAV1_1145	VS1116	glutamyl-tRNA(gln) amidotransferase, C subunit	glutamyl-tRNA(gln) amidotransferase, C subunit(EC:6.3.5.-)	glutamyl-tRNA(Gln) amidotransferase, C subunit	glutamyl-tRNA(Gln) amidotransferase, C subunit	GatABC	GLUADT
350	cbdbA1284	DET1335	DehaBAV1_1146	VS1117	glutamyl-tRNA(Gln) amidotransferase, A subunit	glutamyl-tRNA(Gln) amidotransferase, A subunit(EC:6.3.5.-)	glutamyl-tRNA(Gln) amidotransferase, A subunit(EC:3.5.1.4)	glutamyl-tRNA(Gln) amidotransferase, A subunit	GatABC	GLUADT
351	cbdbA1292	DET1342	DehaBAV1_1153	VS1124	aspartate aminotransferase	aspartate aminotransferase(EC:2.6.1.1)	aminotransferase, class I and II(EC:2.6.1.1)	aminotransferase, class I and II(EC:2.6.1.1)	AspC, AlaT	ALATA_L, ASPTA1
352	cbdbA1293	DET1343	DehaBAV1_1154	VS1125	tryptophanyl-tRNA synthetase	tryptophanyl-tRNA synthetase(EC:6.1.1.2)	tryptophanyl-tRNA synthetase(EC:6.1.1.2)	tryptophanyl-tRNA synthetase(EC:6.1.1.2)	TrpS	TRPTRS
353	cbdbA1294	DET1344	DehaBAV1_1155	VS1126	hydroxymethylbutenyl pyrophosphate reductase	hydroxymethylbutenyl pyrophosphate reductase	hydroxymethylbutenyl pyrophosphate reductase(EC:1.17.1.2)	hydroxymethylbutenyl pyrophosphate reductase(EC:1.17.1.2)	LytB	IPDPS, DMPPS
354	cbdbA1295	DET1345	DehaBAV1_1156	VS1127	putative tagatose 1,6-diphosphate aldolase	tagatose 1,6-diphosphate aldolase	Tagatose-bisphosphate aldolase(EC:4.1.2.40)	Tagatose-bisphosphate aldolase(EC:4.1.2.40)	AgaZ, Fba	TGBPA, FBA
355	cbdbA1296	DET1346	DehaBAV1_1157	VS1128	carbohydrate kinase, PfkB family	carbohydrate kinase, PfkB family	1-phosphofructokinase(EC:2.7.1.56)	1-phosphofructokinase(EC:2.7.1.56)	PfkB, FruK	PFK_2, PFK, F1PK
356	cbdbA1314	DET1363	DehaBAV1_1174	VS1144	putative mannosyl-3-phosphoglycerate synthase	mannosyl-3-phosphoglycerate synthase/mannosyl-3-phosphoglycerate phosphatase(EC:2.4.1.217)	mannosyl-3-phosphoglycerate synthase	mannosyl-3-phosphoglycerate synthase(EC:2.4.1.217,EC:3.1.3.70)	MpgSP	MPGSP
357	cbdbA1316	DET1365	DehaBAV1_1176	VS1146	glutamyl-tRNA synthetase	glutamyl-tRNA synthetase(EC:6.1.1.17)	glutamyl-tRNA synthetase(EC:6.1.1.17)	glutamyl-tRNA synthetase(EC:6.1.1.17)	GlnS, GltX	GLNTRS, GLUTRS

Model gene number	CBDB1 core metabolic genes	195 core metabolic genes	BAV1 core metabolic genes	VS core metabolic genes	CBDB1 annotation	195 annotation	BAV1 annotation	VS annotation	Model protein	Model reaction
358	cbdbA1324	DET1372	DehaBAV1_1183	VS1153	dihydroorotate dehydrogenase	dihydroorotate dehydrogenase(EC:1.3.3.1)	dihydroorotate dehydrogenase family protein	dihydroorotate dehydrogenase family protein	PyrD	DHORDf
359	cbdbA1328	DET1375	DehaBAV1_1186	VS1156	ribosomal large subunit pseudouridine synthase,RluD subfamily	ribosomal large subunit pseudouridine synthase, RluD subfamily	pseudouridine synthase, RluA family(EC:3.2.1.17)	pseudouridine synthase, RluA family(EC:4.2.1.70)	RluD	PSUDS
360	cbdbA1336	DET1382	DehaBAV1_1192	VS1163	nicotinate-nucleotide pyrophosphorylase	nicotinate-nucleotide pyrophosphorylase(EC:2.4.2.19)	nicotinate-nucleotide pyrophosphorylase(EC:2.4.2.19)	nicotinate-nucleotide pyrophosphorylase(EC:2.4.2.19)	NadC1	NNDPR
361	cbdbA1346	DET1390	DehaBAV1_1199	VS1171	PAP2 family protein	PAP2 family protein	phosphoesterase, PA-phosphatase related	phosphoesterase, PA-phosphatase related	PgpA, Pmdpht	PMDPHT, PGPPH
362	cbdbA1353	DET1394	DehaBAV1_1203	VS1176	ornithine carbamoyltransferase	ornithine carbamoyltransferase (EC:2.1.3.3)	ornithine carbamoyltransferase(EC:2.1.3.3)	ornithine carbamoyltransferase(EC:2.1.3.3)	ArgF	OCBT
363	cbdbA1356	DET1397	DehaBAV1_1206	VS1179	glycerol-3-phosphate dehydrogenase,NAD-dependent	glycerol-3-phosphate dehydrogenase, NAD-dependent(EC:1.1.1.94)	Glycerol-3-phosphate dehydrogenase (NAD(P)(+))(EC:1.1.1.94)	Glycerol-3-phosphate dehydrogenase (NAD(P)(+))(EC:1.1.1.94)	GpsA	G3PD1, G3PD2
364	cbdbA1359	DET1400	DehaBAV1_1209	VS1182	co-chaperone protein GrpE	co-chaperone protein GrpE	GrpE protein	GrpE protein	NrdD	RNTR2, RNTR3, RNTR1, RNTR4
365	cbdbA1370	DET1409	DehaBAV1_1216	VS1190	putative transaldolase	transaldolase	putative transaldolase(EC:2.2.1.2)	putative transaldolase(EC:2.2.1.2)	Tal	TAL
366	cbdbA1371	DET1410	DehaBAV1_1217	VS1191	CTP synthase	CTP synthetase(EC:6.3.4.2)	CTP synthase(EC:6.3.4.2)	CTP synthase(EC:6.3.4.2)	PyrG	CTPS1, CTPS2
367	cbdbA1377	DET1415	DehaBAV1_1222	VS1196	amidophosphoribosyltransferase	amidophosphoribosyltransferase(EC:2.4.2.14)	amidophosphoribosyltransferase(EC:2.4.2.14)	amidophosphoribosyltransferase(EC:2.4.2.14)	PurF	GLUPRT

Model gene number	CBDB1 core metabolic genes	195 core metabolic genes	BAV1 core metabolic genes	VS core metabolic genes	CBDB1 annotation	195 annotation	BAV1 annotation	VS annotation	Model protein	Model reaction
368	cbdbA1378	DET1416	DehaBAV1_1223	VS1197	phosphoribosylformylglycinamidine cyclo-ligase	phosphoribosylaminoimidazole synthetase(EC:6.3.3.1)	phosphoribosylformylglycinamidine cyclo-ligase(EC:6.3.3.1)	phosphoribosylformylglycinamidine cyclo-ligase(EC:6.3.3.1)	PurM	PRAIS
369	cbdbA1381	DET1417	DehaBAV1_1225	VS1199	phosphoribosylaminoimidazolecarboxamide formyltransferase	phosphoribosylaminoimidazolecarboxamide formyltransferase/IMP cyclohydrolase(EC:2.1.2.3)	phosphoribosylaminoimidazolecarboxamide formyltransferase/IMP cyclohydrolase(EC:2.1.2.3)	phosphoribosylaminoimidazolecarboxamide formyltransferase/IMP cyclohydrolase(EC:2.1.2.3)	PurH	AICART, IMPCir
370	cbdbA1382	DET1418	DehaBAV1_1226	VS1200	putative nicotinate-nucleotide pyrophosphorylase	nicotinate phosphoribosyltransferase	Quinolinate phosphoribosyl transferase	Quinolinate phosphoribosyl transferase	PncB	NCTPPRT, NAMNPP
371	cbdbA1384	DET1420	DehaBAV1_1228	VS1202	SAM-dependent methyltransferase UbiE/COQ5 family	methyltransferase, UbiE/COQ5 family	Methyltransferase type 11	Methyltransferase type 11	UbiG	DMQMT, OHPHM
372	cbdbA1386	DET1422	DehaBAV1_1230	VS1204	phosphoglycerate mutase family protein	phosphoglycerate mutase family protein	Phosphoglycerate mutase	Phosphoglycerate mutase	GpmA	PGM
373	cbdbA1441	DET1481	DehaBAV1_1271	VS1251	anthranilate synthase component I	anthranilate synthase component I(EC:4.1.3.27)	anthranilate synthase component I(EC:4.1.3.27)	anthranilate synthase component I(EC:4.1.3.27)	TrpE, PabABC	ADCL, ADCS, ANS1
374	cbdbA1442	DET1482	DehaBAV1_1272	VS1252	anthranilate synthase component II	anthranilate synthase component II(EC:4.1.3.27)	glutamine amidotransferase of anthranilate synthase(EC:4.1.3.27)	glutamine amidotransferase of anthranilate synthase(EC:4.1.3.27)	TrpG	ANS1
375	cbdbA1444	DET1483	DehaBAV1_1273	VS1253	anthranilate phosphoribosyltransferase	anthranilate phosphoribosyltransferase(EC:2.4.2.18)	anthranilate phosphoribosyltransferase(EC:2.4.2.18)	anthranilate phosphoribosyltransferase(EC:2.4.2.18)	TrpD	ANPRT
376	cbdbA1445	DET1484	DehaBAV1_1274	VS1254	indole-3-glycerol phosphate synthase	indole-3-glycerol phosphate synthase(EC:4.1.1.48)	Indole-3-glycerol-phosphate synthase(EC:4.1.1.48)	Indole-3-glycerol-phosphate synthase(EC:4.1.1.48)	TrpC	IGPS

Model gene number	CBDB1 core metabolic genes	195 core metabolic genes	BAV1 core metabolic genes	VS core metabolic genes	CBDB1 annotation	195 annotation	BAV1 annotation	VS annotation	Model protein	Model reaction
377	cbdbA1447	DET1485	DehaBAV1_1275	VS1255	N-(5phosphoribosyl)anthranilate isomerase	N-(5'phosphoribosyl)anthranilate isomerase(EC:5.3.1.24)	Phosphoribosylanthranilate isomerase(EC:5.3.1.24)	Phosphoribosylanthranilate isomerase(EC:5.3.1.24)	TrpF	PRAI
378	cbdbA1449	DET1486	DehaBAV1_1276	VS1256	3-deoxy-D-arabino-heptulosonate 7-phosphate (DAHP) synthetase	3-deoxy-7-phosphoheptulonate synthase(EC:2.5.1.54,EC:4.1.2.15)	phospho-2-dehydro-3-deoxyheptonate aldolase(EC:2.5.1.54)	phospho-2-dehydro-3-deoxyheptonate aldolase(EC:2.5.1.54)	AroA2	DAHPS
379	cbdbA1450	DET1487	DehaBAV1_1277	VS1257	tryptophan synthase, beta subunit	tryptophan synthase subunit beta(EC:4.2.1.20)	tryptophan synthase, beta subunit(EC:4.2.1.20)	tryptophan synthase, beta subunit(EC:4.2.1.20)	TrpB	TRPS1
380	cbdbA1451	DET1488	DehaBAV1_1278	VS1258	tryptophan synthase, alpha subunit	tryptophan synthase, alpha subunit(EC:4.2.1.20)	tryptophan synthase, alpha subunit(EC:4.2.1.20)	tryptophan synthase, alpha subunit(EC:4.2.1.20)	TrpA	TRPS1
381	cbdbA1457	DET1489	DehaBAV1_1279	VS1265	thymidylate synthase ThyX	thymidylate synthase, flavin-dependent(EC:2.1.1.148)	thymidylate synthase, flavin-dependent(EC:2.1.1.148)	thymidylate synthase, flavin-dependent(EC:2.1.1.148)	ThyX	FDTS
382	cbdbA1458	DET1490	DehaBAV1_1280	VS1266	peptide ABC transporter, ATP-binding protein	peptide ABC transporter, ATP-binding protein	oligopeptide/dipeptide ABC transporter, ATPase subunit	ABC transporter related	NikA	NIabc
383	cbdbA1459	DET1491	DehaBAV1_1281	VS1267	peptide ABC transporter, ATP-binding protein	peptide ABC transporter, ATP-binding protein	ABC transporter related	oligopeptide/dipeptide ABC transporter, ATPase subunit	NikB	NIabc
384	cbdbA1461	DET1492	DehaBAV1_1282	VS1268	peptide ABC transporter, permease protein	peptide ABC transporter, permease protein	binding-protein-dependent transport systems inner membrane component	binding-protein-dependent transport systems inner membrane component	NikC	NIabc

Model gene number	CBDB1 core metabolic genes	195 core metabolic genes	BAV1 core metabolic genes	VS core metabolic genes	CBDB1 annotation	195 annotation	BAV1 annotation	VS annotation	Model protein	Model reaction
385	cbdbA1462	DET1493	DehaBAV1_1283	VS1269	peptide ABC transporter, permease protein	peptide ABC transporter, permease protein	binding-protein-dependent transport systems inner membrane component	binding-protein-dependent transport systems inner membrane component	NikD	NIabc
386	cbdbA1463	DET1494	DehaBAV1_1284	VS1270	peptide ABC transporter, periplasmic peptide-binding protein	peptide ABC transporter, periplasmic peptide-binding protein	extracellular solute-binding protein, family 5	extracellular solute-binding protein, family 5	NikE	NIabc
387	cbdbA1475	DET1503	DehaBAV1_1293	VS1277	ferrous iron transport protein B	ferrous iron transport protein B, putative	small GTP-binding protein	small GTP-binding protein	FeoB1	FE2abc
388	cbdbA1637	DET1544	DehaBAV1_0282	VS1435	putative reductive dehalogenase anchoring protein	reductive dehalogenase anchoring protein, putative	hypothetical protein	putative reductive dehalogenase anchoring protein	RdhB	RDR1, RDR2
389	cbdbA1639	DET1547	DehaBAV1_1303	VS1437	prephenate dehydratase	prephenate dehydratase(EC:4.2.1.51)	Prephenate dehydratase(EC:4.2.1.51)	Prephenate dehydratase(EC:4.2.1.51)	PheA	PPNDH, CHORM
390	cbdbA1643	DET1550	DehaBAV1_1307	VS1440	glutamyl-tRNA(Gln) amidotransferase, B subunit	aspartyl/glutamyl-tRNA amidotransferase subunit B(EC:6.3.5.-)	glutamyl-tRNA(Gln) amidotransferase, B subunit	glutamyl-tRNA(Gln) amidotransferase, B subunit	GatABC	GLUADT
391	cbdbA1652	DET1570	DehaBAV1_1316	VS1448	hydrogenase, group 4, HycG subunit	hydrogenase, group 4, HycG subunit, putative	NADH ubiquinone oxidoreductase, 20 kDa subunit	NADH ubiquinone oxidoreductase, 20 kDa subunit	Nadhd5	NADH6, NADH7, NADH4, NADH8
392	cbdbA1653	DET1571	DehaBAV1_1317	VS1449	hydrogenase, group 4, HycE subunit	hydrogenase, group 4, HycE subunit, putative	NADH-ubiquinone oxidoreductase, chain 49kDa	NADH-ubiquinone oxidoreductase, chain 49kDa	Nadhd5	NADH6, NADH7, NADH4, NADH8
393	cbdbA1655	DET1572	DehaBAV1_1318	VS1450	hydrogenase, membrane subunit	NADH dehydrogenase subunit N	NADH dehydrogenase (quinone)(EC:1.6.99.5)	NADH dehydrogenase (quinone)(EC:1.6.99.5)	Nadhd5	NADH6, NADH7, NADH4, NADH8

Model gene number	CBDB1 core metabolic genes	195 core metabolic genes	BAV1 core metabolic genes	VS core metabolic genes	CBDB1 annotation	195 annotation	BAV1 annotation	VS annotation	Model protein	Model reaction
394	cbdbA1656	DET1573	DehaBAV1_1319	VS1451	putative hydrogenase subunit	hydrogenase subunit, putative	Hydrogenase 4 membrane component (E)-like protein	hydrogenase subunit, putative	Nadhd5	NADH6, NADH7, NADH4, NADH8
395	cbdbA1658	DET1574	DehaBAV1_1320	VS1452	hydrogenase, HycD subunit	hydrogenase, HycD subunit, putative	Formate hydrogenlyase subunit 4-like protein	hydrogenase, HycD subunit	Nadhd5	NADH6, NADH7, NADH4, NADH8
396	cbdbA1659	DET1575	DehaBAV1_1321	VS1453	hydrogenase, HycC subunit	NADH dehydrogenase subunit N	NADH dehydrogenase (quinone)(EC:1.6.99.5)	NADH dehydrogenase (quinone)(EC:1.6.99.5)	Nadhd5	NADH6, NADH7, NADH4, NADH8
397	cbdbA1661	DET1576	DehaBAV1_1322	VS1454	putative methylglyoxal synthase	methylglyoxal synthase, putative(EC:4.2.3.3)	Methylglyoxal synthase(EC:4.2.3.3)	MGS domain protein(EC:4.2.3.3)	MgsA	MGSA
398	cbdbA1678	DET1588	DehaBAV1_1336	VS1472	serine O-acetyltransferase	serine O-acetyltransferase(EC:2.3.1.30)	serine O-acetyltransferase(EC:2.3.1.30)	serine O-acetyltransferase(EC:2.3.1.30)	CysE	SERAT
399	cbdbA1681	DET1590	DehaBAV1_1338	VS1474	quinolinate synthetase complex, subunit A	quinolinate synthetase	quinolinate synthetase complex, A subunit(EC:2.1.1.63)	quinolinate synthetase complex, A subunit	NadA	QULNS
400	cbdbA1683	DET1591	DehaBAV1_1339	VS1475	SAM-dependent methyltransferase UbiE/COQ5 family	methyltransferase, UbiE/COQ5 family(EC:2.1.1.-)	Methyltransferase type 11	Methyltransferase type 11	UbiE	OMBZLM
401	cbdbA1696	DET1603	DehaBAV1_1349	VS1485	dihydropteroate synthase	dihydropteroate synthase(EC:2.5.1.15)	dihydropteroate synthase(EC:2.5.1.15)	dihydropteroate synthase(EC:2.5.1.15)	FolP, FolB	DHPS1, DHNPA, DHPS3
402	cbdbA1698	DET1604	DehaBAV1_1350	VS1486	2-amino-4-hydroxy-6-hydroxymethyldihydropteridine pyrophosphokinase	2-amino-4-hydroxy-6-hydroxymethyldihydropteridine pyrophosphokinase(EC:2.7.6.3)	2-amino-4-hydroxy-6-hydroxymethyldihydropteridine pyrophosphokinase(EC:2.7.6.3)	2-amino-4-hydroxy-6-hydroxymethyldihydropteridine pyrophosphokinase (EC:2.7.6.3)	FolB, FolK	DHNPA, HPPK

Model gene number	CBDB1 core metabolic genes	195 core metabolic genes	BAV1 core metabolic genes	VS core metabolic genes	CBDB1 annotation	195 annotation	BAV1 annotation	VS annotation	Model protein	Model reaction
403	cbdbA1699	DET1605	DehaBAV1_1351	VS1487	6-pyruvoyl tetrahydrobiopterin synthase	6-pyruvoyl tetrahydrobiopterin synthase, putative	6-pyruvoyl tetrahydropterin synthase and hypothetical protein	6-pyruvoyl tetrahydropterin synthase and hypothetical protein	Ptps	PTHPS
404	cbdbA1708	DET1614	DehaBAV1_1360	VS1496	homocitrate synthase	homocitrate synthase(EC:4.1.3.21)	2-isopropylmalate synthase(EC:2.3.3.13)	2-isopropylmalate synthase(EC:2.3.3.13)	NifV	HCITS
405	cbdbA1722	DET1626	DehaBAV1_1371	VS1508	N-acetyl-gamma-glutamyl-phosphate reductase	N-acetyl-gamma-glutamyl-phosphate reductase(EC:1.2.1.38)	N-acetyl-gamma-glutamyl-phosphate reductase(EC:1.2.1.38)	N-acetyl-gamma-glutamyl-phosphate reductase(EC:1.2.1.38)	ArgC	AGPR
406	cbdbA1724	DET1627	DehaBAV1_1372	VS1509	putative sodium exchanger	sodium/hydrogen exchanger family protein	sodium/hydrogen exchanger	sodium/hydrogen exchanger	KefC	Kt6
407	cbdbA1731	DET1633	DehaBAV1_1378	VS1515	aspartate kinase, monofunctional class	aspartate kinase, monofunctional class(EC:2.7.2.4)	aspartate kinase(EC:2.7.2.4)	aspartate kinase(EC:2.7.2.4)	AspK	ASPK
408	cbdbA1734	DET1635	DehaBAV1_1380	VS1517	bisphosphoglycerate-independent phosphoglycerate mutase	cofactor-independent phosphoglycerate mutase	proposed homoserine kinase(EC:5.4.2.1)	proposed homoserine kinase(EC:5.4.2.1)	Pgml	PGM
409	cbdbA1735	DET1637	DehaBAV1_1381	VS1519	mazG family protein	mazG family protein	MazG family protein	MazG family protein	MazG	NTPP4, NTPP3, NTPP1, NTPP2, NTPP5, NTPP8, NTPP7, NTPP6
410	cbdbA1741	DET1641	DehaBAV1_1385	VS1523	aldolase, class II	L-fuculose phosphate aldolase	class II aldolase/adducin family protein	class II aldolase/adducin family protein	FucA	FCLPA
411	cbdbA1742	DET1642	DehaBAV1_1386	VS1524	methionyl-tRNA formyltransferase	methionyl-tRNA formyltransferase(EC:2.1.2.9)	methionyl-tRNA formyltransferase	methionyl-tRNA formyltransferase	Fmt	FMETTRS

Model gene number	CBDB1 core metabolic genes	195 core metabolic genes	BAV1 core metabolic genes	VS core metabolic genes	CBDB1 annotation	195 annotation	BAV1 annotation	VS annotation	Model protein	Model reaction
412	cbdbA331	DET0386	DehaBAV1_ 0366	VS329	tetrapyrrole methylase family protein	tetrapyrrole methylase family protein	Uroporphyrin-III C/tetrapyrrole (Corrin/Porphyrin) methyltransferase	Uroporphyrin-III C/tetrapyrrole (Corrin/Porphyrin) methyltransferase	HemD	UPP3MT, UPP3S
413	cbdbA637	DET0687	DehaBAV1_ 0623	VS591	conserved hypothetical protein	conserved hypothetical protein	conserved hypothetical protein	conserved hypothetical protein	CbiZ	ADCOBHS

Table 4. Gene Correspondence for Dispensable Genes Mapped to New Reactions of *i* AI549

Model gene number	CBDB1 dispensable metabolic genes	195 dispensable metabolic genes	BAV1 dispensable metabolic genes	VS dispensable metabolic genes	CBDB1 annotation	195 annotation	BAV1 annotation	VS annotation	Model protein	Model reaction
414	cbdbA155	No gene	DehaBAV1_0238	VS140	Ornithine cyclodeaminase(EC:4.3.1.12)	No gene	Ornithine cyclodeaminase(EC:4.3.1.12)	Ornithine cyclodeaminase(EC:4.3.1.12)	Ocd	ORNCD
415	cbdbA181	No gene	DehaBAV1_0218	VS161	Threonine aldolase(EC:4.1.2.5)	No gene	Threonine aldolase(EC:4.1.2.5)	Threonine aldolase(EC:4.1.2.5)	LtaA	THRA, THRLAD
416	cbdbA247	DET0240	No gene	VS78	Uroporphyrin-III C/tetrapyrrole (Corrin/Porphyrin) methyltransferase	tetrapyrrole methylase family protein	No gene	Uroporphyrin-III C/tetrapyrrole (Corrin/Porphyrin) methyltransferase	CbiE	CPC6MT
417	cbdbA257	DET0248	No gene	VS70	aminotransferase, class V(EC:2.8.1.7)	cysteine desulfurase(EC:2.8.1.7)	No gene	aminotransferase, class V(EC:2.8.1.7)	IscS	THZPSN
418	cbdbA260	DET0297	No gene	No gene	Pyridoxamine 5-phosphate oxidase family {Dehalococcoides sp. CBDB1}	hypothetical protein	No gene	No gene	PdxHec	PDX5PO
419	cbdbA1077	No gene	DehaBAV1_0975	No gene	GCN5-related N-acetyltransferase	No gene	GCN5-related N-acetyltransferase	No gene	SucCD1	SUCOAS
420	cbdbA1338	No gene	DehaBAV1_1193	VS1164	L-aspartate oxidase(EC:1.4.3.16)	No gene	L-aspartate oxidase(EC:1.4.3.16)	L-aspartate oxidase(EC:1.4.3.16)	Frd, NadB	ASPO1, ASPO3, ASPO4, ASPO5, ASPO6, FRD5
421	cbdbA1527	DET1510	No gene	No gene	conserved hypothetical protein {Dehalococcoides sp. CBDB1}	hypothetical protein	No gene	No gene	ThiD	PMPK

Model gene number	CBDB1 dispensable metabolic genes	195 dispensable metabolic genes	BAV1 dispensable metabolic genes	VS dispensable metabolic genes	CBDB1 annotation	195 annotation	BAV1 annotation	VS annotation	Model protein	Model reaction
422	cbdbA1557	DET0089	No gene	No gene	radical SAM domain protein {Dehalococcoides sp. CBDB1}	radical SAM domain protein	No gene	No gene	PflC	PFLi, OBTFL
423	No gene	DET0207	DehaBAV1_0146	No gene	No gene	phosphoheptose isomerase	sugar isomerase (SIS)	No gene	GmhA	S7PI
424	No gene	DET0202	DehaBAV1_0151	VS190	No gene	histidinol-phosphate phosphatase family protein/glycosyl transferase, group 2 family protein	histidinol-phosphate phosphatase family protein	histidinol-phosphate phosphatase family protein	HisJ	HISTP

Table 5. Gene Correspondence for Dispensable Genes Mapped to Reactions of *i* AI549 Already Present in Core

Model gene number	CBDB1 dispensable metabolic genes	195 dispensable metabolic genes	BAV1 dispensable metabolic genes	VS dispensable metabolic genes	CBDB1 annotation	195 annotation	BAV1 annotation	VS annotation	Model protein	Model reaction
425	cbdbA80	DET1559	No gene	No gene	reductive dehalogenase homologous protein RdhA7 {Dehalococcoides sp. CBDB1}	reductive dehalogenase, putative	No gene	No gene	RdhA	RDR1, RDR2
426	cbdbA84	No gene	No gene	VS1342	putative reductive dehalogenase	No gene	No gene	reductive dehalogenase	RdhA	RDR1, RDR2
427	cbdbA88	DET0311	DehaBAV1_0104	No gene	putative reductive dehalogenase	reductive dehalogenase, putative	reductive dehalogenase	No gene	RdhA	RDR1, RDR2
428	cbdbA251	DET0244	No gene	VS74	ribonucleoside-diphosphate reductase, adenosylcobalamin-dependent	ribonucleotide reductase(EC:1.17.4.1)	No gene	ribonucleoside-diphosphate reductase, adenosylcobalamin-dependent	NrdEF	RNDR3, RNDR1, RNDR2, RNDR4
429	cbdbA253	DET0245	No gene	VS73	ATP:corrinoid adenosyltransferase BtuR/CobO/CobP	cob(I)alamin adenosyltransferase (EC:2.5.1.17)	No gene	ATP:corrinoid adenosyltransferase BtuR/CobO/CobP	CobA	CBLATr, CYRDAAT, CBIAT
430	cbdbA254	DET0246	No gene	VS72	cobalamin biosynthesis protein CobD	cobalamin biosynthesis protein CobD	No gene	cobalamin biosynthesis protein CobD	CbiD	ADCPS2, ADCOBAS
431	cbdbA259	DET0250	No gene	VS68	precorrin-6y C5,15-methyltransferase (decarboxylating), CbiE subunit	iron ABC transporter, periplasmic iron-binding protein, putative	No gene	precorrin-6y C5,15-methyltransferase (decarboxylating), CbiE subunit	BtuF	CBL1abc
432	cbdbA1034	No gene	DehaBAV1_0939	VS928	oligopeptide/dipeptide ABC transporter, ATPase subunit	No gene	oligopeptide/dipeptide ABC transporter, ATPase subunit	oligopeptide/dipeptide ABC transporter, ATPase subunit	NikA	NIabc

Model gene number	CBDB1 dispensable metabolic genes	195 dispensable metabolic genes	BAV1 dispensable metabolic genes	VS dispensable metabolic genes	CBDB1 annotation	195 annotation	BAV1 annotation	VS annotation	Model protein	Model reaction
433	cbdbA1036	No gene	DehaBAV1_0940	VS929	oligopeptide/dipeptide ABC transporter, ATPase subunit	No gene	oligopeptide/dipeptide ABC transporter, ATPase subunit	oligopeptide/dipeptide ABC transporter, ATPase subunit	NikB	NIabc
434	cbdbA1037	No gene	DehaBAV1_0941	VS930	binding-protein-dependent transport systems inner membrane component	No gene	binding-protein-dependent transport systems inner membrane component	binding-protein-dependent transport systems inner membrane component	NikC	NIabc
435	cbdbA1038	No gene	DehaBAV1_0942	VS931	Nickel-transporting ATPase(EC:3.6.3.24)	No gene	Nickel-transporting ATPase(EC:3.6.3.24)	binding-protein-dependent transport systems inner membrane component	NikD	NIabc
436	cbdbA1039	No gene	DehaBAV1_0943	VS932	extracellular solute-binding protein, family 5	No gene	extracellular solute binding protein, family 5	extracellular solute-binding protein, family 5	NikE	NIabc
437	cbdbA1160	No gene	DehaBAV1_1054	VS1026	arsenical-resistance protein	No gene	arsenical-resistance protein	arsenical-resistance protein	ArsB2	ANTIMt1, ARSNAt1, ARSt1
438	cbdbA1163	No gene	DehaBAV1_1056	VS1028	Methionine sulfoxide reductase B	No gene	Methionine sulfoxide reductase B	methionine-R-sulfoxide reductase	MsrA	METSR-S2, METSR-S1
439	cbdbA1453	DET1535	No gene	VS1427	putative reductive dehalogenase	reductive dehalogenase, putative	No gene	reductive dehalogenase	RdhA	RDR1, RDR2
440	cbdbA1454	No gene	No gene	VS1262	putative reductive dehalogenase anchoring protein	No gene	No gene	putative reductive dehalogenase anchoring protein	RdhB	RDR1, RDR2
441	cbdbA1455	No gene	No gene	VS1263	putative reductive dehalogenase	No gene	No gene	reductive dehalogenase	RdhA	RDR1, RDR2

Model gene number	CBDB1 dispensable metabolic genes	195 dispensable metabolic genes	BAV1 dispensable metabolic genes	VS dispensable metabolic genes	CBDB1 annotation	195 annotation	BAV1 annotation	VS annotation	Model protein	Model reaction
442	cbdbA1490	DET1537	No gene	VS1429	reductive dehalogenase anchoring protein, putative {Dehalococcoides sp. CBDB1}	reductive dehalogenase anchoring protein, putative	No gene	reductive dehalogenase anchoring protein, putative	RdhB	RDR1, RDR2
443	cbdbA1491	DET1538	No gene	VS1430	trichloroethene reductive dehalogenase {Dehalococcoides sp. CBDB1}	reductive dehalogenase, putative	No gene	reductive dehalogenase	RdhA	RDR1, RDR2
444	cbdbB33	DET0307	DehaBAV1_0283	No gene	reductive dehalogenase anchoring protein, putative	reductive dehalogenase anchoring protein, putative	hypothetical protein	No gene	RdhB	RDR1, RDR2
445	cbdbA1495	DET0306	No gene	No gene	trichloroethene reductive dehalogenase {Dehalococcoides sp. CBDB1}	reductive dehalogenase, putative	No gene	No gene	RdhA	RDR1, RDR2
446	cbdbA1507	DET0319	No gene	VS1392	putative reductive dehalogenase anchoring protein	reductive dehalogenase anchoring protein, putative	No gene	reductive dehalogenase anchoring protein, putative	RdhB	RDR1, RDR2
447	cbdbA1536	No gene	No gene	VS1386	putative reductive dehalogenase anchoring protein	No gene	No gene	putative reductive dehalogenase anchoring protein	RdhB	RDR1, RDR2
448	cbdbA1539	No gene	DehaBAV1_0173	No gene	putative reductive dehalogenase	No gene	reductive dehalogenase	No gene	RdhA	RDR1, RDR2
449	cbdbA1541	No gene	DehaBAV1_0297	VS87	reductive dehalogenase anchoring protein, putative {Dehalococcoides sp. CBDB1}	No gene	hypothetical protein	putative reductive dehalogenase anchoring protein	RdhB	RDR1, RDR2

Model gene number	CBDB1 dispensable metabolic genes	195 dispensable metabolic genes	BAV1 dispensable metabolic genes	VS dispensable metabolic genes	CBDB1 annotation	195 annotation	BAV1 annotation	VS annotation	Model protein	Model reaction
450	cbdbA1545	No gene	No gene	VS1363	putative reductive dehalogenase anchoring protein	No gene	No gene	putative reductive dehalogenase anchoring protein	RdhB	RDR1, RDR2
451	cbdbA1546	DET1519	No gene	VS1364	reductive dehalogenase	reductive dehalogenase, putative	No gene	reductive dehalogenase	RdhA	RDR1, RDR2
452	cbdbA1549	DET1521	No gene	VS1377	putative reductive dehalogenase anchoring protein	reductive dehalogenase anchoring protein, putative	No gene	putative reductive dehalogenase anchoring protein	RdhB	RDR1, RDR2
453	cbdbA1550	DET1522	No gene	VS1378	putative reductive dehalogenase	reductive dehalogenase, putative	No gene	reductive dehalogenase	RdhA	RDR1, RDR2
454	cbdbA1568	No gene	No gene	VS1369	Glutamate--ammonia ligase(EC:6.3.1.2)	No gene	No gene	Glutamate--ammonia ligase(EC:6.3.1.2)	GlnA	GLNS
455	cbdbA1569	DET1521	No gene	VS1377	putative reductive dehalogenase anchoring protein	reductive dehalogenase anchoring protein, putative	No gene	putative reductive dehalogenase anchoring protein	RdhB	RDR1, RDR2
456	cbdbA1570	DET1522	No gene	VS1378	putative reductive dehalogenase	reductive dehalogenase, putative	No gene	reductive dehalogenase	RdhA	RDR1, RDR2
457	cbdbA1573	DET1518	No gene	VS1374	putative reductive dehalogenase anchoring protein	reductive dehalogenase anchoring protein, putative	No gene	putative reductive dehalogenase anchoring protein	RdhB	RDR1, RDR2
458	cbdbA1575	DET1519	No gene	VS1375	putative reductive dehalogenase	reductive dehalogenase, putative	No gene	reductive dehalogenase	RdhA	RDR1, RDR2
459	cbdbA1578	No gene	No gene	VS1383	putative reductive dehalogenase	No gene	No gene	reductive dehalogenase	RdhA	RDR1, RDR2

Model gene number	CBDB1 dispensable metabolic genes	195 dispensable metabolic genes	BAV1 dispensable metabolic genes	VS dispensable metabolic genes	CBDB1 annotation	195 annotation	BAV1 annotation	VS annotation	Model protein	Model reaction
460	cbdbA1581	No gene	No gene	VS1386	putative reductive dehalogenase anchoring protein	No gene	No gene	putative reductive dehalogenase anchoring protein	RdhB	RDR1, RDR2
461	cbdbA1582	No gene	DehaBAV1_0112	VS1387	reductive dehalogenase homologous protein RdhA9 {Dehalococcoides sp. CBDB1}	No gene	reductive dehalogenase	reductive dehalogenase	RdhA	RDR1, RDR2
462	cbdbA1587	DET0319	No gene	VS1392	putative reductive dehalogenase anchoring protein	reductive dehalogenase anchoring protein, putative	No gene	reductive dehalogenase anchoring protein, putative	RdhB	RDR1, RDR2
463	cbdbA1588	DET0318	No gene	VS1393	putative reductive dehalogenase	reductive dehalogenase, putative	No gene	reductive dehalogenase	RdhA	RDR1, RDR2
464	cbdbA1594	DET1534	No gene	VS1315	putative reductive dehalogenase anchoring protein	reductive dehalogenase anchoring protein, putative	No gene	putative reductive dehalogenase anchoring protein	RdhB	RDR1, RDR2
465	cbdbA1595	DET1535	No gene	VS1316	reductive dehalogenase	reductive dehalogenase, putative	No gene	reductive dehalogenase	RdhA	RDR1, RDR2
466	cbdbA1597	No gene	No gene	VS1401	putative reductive dehalogenase anchoring protein	No gene	No gene	putative reductive dehalogenase anchoring protein	RdhB	RDR1, RDR2
467	cbdbA1598	No gene	No gene	VS1402	putative reductive dehalogenase	No gene	No gene	reductive dehalogenase	RdhA	RDR1, RDR2

Model gene number	CBDB1 dispensable metabolic genes	195 dispensable metabolic genes	BAV1 dispensable metabolic genes	VS dispensable metabolic genes	CBDB1 annotation	195 annotation	BAV1 annotation	VS annotation	Model protein	Model reaction
468	cbdbA1617	No gene	No gene	VS1420	putative reductive dehalogenase anchoring protein	No gene	No gene	putative reductive dehalogenase anchoring protein	RdhB	RDR1, RDR2
469	cbdbA1618	No gene	No gene	VS1421	putative reductive dehalogenase	No gene	No gene	reductive dehalogenase	RdhA	RDR1, RDR2
470	cbdbA1623	No gene	DehaBAV1_0111	VS1426	putative reductive dehalogenase anchoring protein	No gene	hypothetical protein	putative reductive dehalogenase anchoring protein	RdhB	RDR1, RDR2
471	cbdbA1624	DET1535	No gene	VS1427	putative reductive dehalogenase	reductive dehalogenase, putative	No gene	reductive dehalogenase	RdhA	RDR1, RDR2
472	cbdbA1626	DET1537	No gene	VS1429	putative reductive dehalogenase anchoring protein	reductive dehalogenase anchoring protein, putative	No gene	reductive dehalogenase anchoring protein, putative	RdhB	RDR1, RDR2
473	cbdbA1627	DET1538	No gene	VS1430	putative reductive dehalogenase	reductive dehalogenase, putative	No gene	reductive dehalogenase	RdhA	RDR1, RDR2
474	cbdbA1638	DET1545	No gene	VS1436	reductive dehalogenase homologous protein RdhA2 {Dehalococcoides sp. CBDB1}	reductive dehalogenase, putative	No gene	reductive dehalogenase	RdhA	RDR1, RDR2
475	cbdbA1667	No gene	DehaBAV1_1328	VS1458	NAD-dependent epimerase/dehydratase	No gene	NAD-dependent epimerase/dehydratase		GalE	UDPG4E
476	cbdbA1668	No gene	DehaBAV1_1329	VS1459	NAD-dependent epimerase/dehydratase	No gene	NAD-dependent epimerase/dehydratase	NAD-dependent epimerase/dehydratase	GalE	UDPG4E

Model gene number	CBDB1 dispensable metabolic genes	195 dispensable metabolic genes	BAV1 dispensable metabolic genes	VS dispensable metabolic genes	CBDB1 annotation	195 annotation	BAV1 annotation	VS annotation	Model protein	Model reaction
477	No gene	DET0204	DehaBAV1_0149	No gene	No gene	NAD-dependent epimerase/dehydratase family protein	NAD-dependent epimerase/dehydratase		GalE	UDPG4E
478	No gene	DET0079	DehaBAV1_0847	VS1291	No gene	trichloroethene reductive dehalogenase	reductive dehalogenase	reductive dehalogenase	RdhA	RDR1, RDR2
479	No gene	DET0312	DehaBAV1_0103	No gene	No gene	reductive dehalogenase anchoring protein, putative	hypothetical protein	No gene	RdhB	RDR1, RDR2
480	No gene	DET0296	DehaBAV1_0298	No gene	No gene	precorrin-6Y C5,15-methyltransferase, putative	hypothetical protein	No gene	BtuF	CBL1abc
481	No gene	DET0205	DehaBAV1_0148	No gene	No gene	D-glycero-D-manno-heptose 1-phosphate guanosyltransferase	Nucleotidyl transferase	No gene	Mpg, GlgC	MAN1PT1, GLGC
482	No gene		DehaBAV1_0277	VS103	No gene	No gene	hypothetical protein	reductive dehalogenase anchoring protein, putative	RdhB	RDR1, RDR2
483	No gene	DET0214	DehaBAV1_0139	No gene	No gene	iron-sulfur cluster-binding protein/coenzyme F420-reducing hydrogenase, beta subunit, putative	coenzyme F420 hydrogenase/dehydrogenase beta subunit domain protein	No gene	FrdH	FRHD
484	No gene		DehaBAV1_0276	VS104	No gene	No gene	reductive dehalogenase	reductive dehalogenase	RdhA	RDR1, RDR2
485	No gene		DehaBAV1_0281	VS99	No gene	No gene	reductive dehalogenase	reductive dehalogenase	RdhA	RDR1, RDR2
486	No gene		DehaBAV1_0284	VS96	No gene	No gene	reductive dehalogenase	reductive dehalogenase	RdhA	RDR1, RDR2

Model gene number	CBDB1 dispensable metabolic genes	195 dispensable metabolic genes	BAV1 dispensable metabolic genes	VS dispensable metabolic genes	CBDB1 annotation	195 annotation	BAV1 annotation	VS annotation	Model protein	Model reaction
487	No gene		DehaBAV1_0296	VS88	No gene	No gene	reductive dehalogenase	reductive dehalogenase	RdhA	RDR1, RDR2
488	No gene	DET1565	DehaBAV1_1310	No gene	No gene	iron-sulfur cluster-binding protein	4Fe-4S ferredoxin, iron-sulfur binding domain protein	No gene	Fdx	FRHD

Table 6. Gene Correspondence for Unique Genes Mapped to New Reactions of *i* AI549

Model gene number	CBDB1 unique metabolic genes	195 unique metabolic genes	BAV1 unique metabolic genes	VS unique metabolic genes	CBDB1 annotation	195 annotation	BAV1 annotation	VS annotation	Model protein	Model reaction
489	No gene	DET1150	No gene	No gene	No gene	nitrogenase	No gene	No gene	Nif	NITr
490	No gene	DET1151	No gene	No gene	No gene	dinitrogenase iron-molybdenum cofactor NifB/Y/X family protein	No gene	No gene	Nif	NITr
491	No gene	DET1152	No gene	No gene	No gene	nitrogenase molybdenum-iron protein, beta subunit, putative	No gene	No gene	Nif	NITr
492	No gene	DET1153	No gene	No gene	No gene	nitrogenase MoFe cofactor biosynthesis protein NifE	No gene	No gene	Nif	NITr
493	No gene	DET1154	No gene	No gene	No gene	nitrogenase molybdenum-iron protein, beta subunit(EC:1.18.6.1)	No gene	No gene	Nif	NITr
494	No gene	DET1155	No gene	No gene	No gene	nitrogenase molybdenum-iron protein alpha chain(EC:1.18.6.1)	No gene	No gene	Nif	NITr
495	No gene	DET1158	No gene	No gene	No gene	nitrogenase iron protein(EC:1.18.6.1)	No gene	No gene	Nif	NITr
496	No gene	DET1159	No gene	No gene	No gene	molybdenum ABC transporter, ATP-binding protein	No gene	No gene	ModABCE	MOBDabc

Model gene number	CBDB1 unique metabolic genes	195 unique metabolic genes	BAV1 unique metabolic genes	VS unique metabolic genes	CBDB1 annotation	195 annotation	BAV1 annotation	VS annotation	Model protein	Model reaction
497	No gene	DET1160	No gene	No gene	No gene	molybdenum ABC transporter, permease protein	No gene	No gene	ModABCE	MOBDabc
498	No gene	DET1161	No gene	No gene	No gene	molybdenum ABC transporter, periplasmic molybdate-binding protein	No gene	No gene	ModABCE	MOBDabc

Table 7. Gene Correspondence for Unique Genes Mapped to Reactions of *i* AI549 Already Present in Core

Model gene number	CBDB1 unique metabolic genes	195 unique metabolic genes	BAV1 unique metabolic genes	VS unique metabolic genes	CBDB1 annotation	195 annotation	BAV1 annotation	VS annotation	Model protein	Model reaction
499	cbdbA85	No gene	No gene	No gene	putative reductive dehalogenase anchoring protein	No gene	No gene	No gene	RdhB	RDR1, RDR2
500	cbdbA1195	No gene	No gene	No gene	putative deoxyguanosinetriphosphate triphosphohydrolase	No gene	No gene	No gene	Dgt	NTPTP2, DGTPH
501	cbdbA1452	No gene	No gene	No gene	putative reductive dehalogenase anchoring protein	No gene	No gene	No gene	RdhB	RDR1, RDR2
502	cbdbA1502	No gene	No gene	No gene	putative reductive dehalogenase anchoring protein	No gene	No gene	No gene	RdhB	RDR1, RDR2
503	cbdbA1503	No gene	No gene	No gene	putative reductive dehalogenase	No gene	No gene	No gene	RdhA	RDR1, RDR2
504	cbdbA1508	No gene	No gene	No gene	putative reductive dehalogenase	No gene	No gene	No gene	RdhA	RDR1, RDR2
505	cbdbA1535	No gene	No gene	No gene	putative reductive dehalogenase	No gene	No gene	No gene	RdhA	RDR1, RDR2
506	cbdbA1540	No gene	No gene	No gene	reductive dehalogenase-homologous N-terminal fragment	No gene	No gene	No gene	RdhA	RDR1, RDR2
507	cbdbA1542	No gene	No gene	No gene	putative reductive dehalogenase	No gene	No gene	No gene	RdhA	RDR1, RDR2
508	cbdbA1559	No gene	No gene	No gene	putative reductive dehalogenase anchoring protein	No gene	No gene	No gene	RdhB	RDR1, RDR2
509	cbdbA1560	No gene	No gene	No gene	putative reductive dehalogenase	No gene	No gene	No gene	RdhA	RDR1, RDR2

Model gene number	CBDB1 unique metabolic genes	195 unique metabolic genes	BAV1 unique metabolic genes	VS unique metabolic genes	CBDB1 annotation	195 annotation	BAV1 annotation	VS annotation	Model protein	Model reaction
510	cbdbA1562	No gene	No gene	No gene	putative reductive dehalogenase anchoring protein	No gene	No gene	No gene	RdhB	RDR1, RDR2
511	cbdbA1563	No gene	No gene	No gene	putative reductive dehalogenase	No gene	No gene	No gene	RdhA	RDR1, RDR2
512	cbdbA1577	No gene	No gene	No gene	putative reductive dehalogenase anchoring protein	No gene	No gene	No gene	RdhB	RDR1, RDR2
513	No gene	DET0088	No gene	No gene	No gene	reductive dehalogenase domain protein	No gene	No gene	RdhA	RDR1, RDR2
514	No gene	DET0173	No gene	No gene	No gene	reductive dehalogenase, putative	No gene	No gene	RdhA	RDR1, RDR2
515	No gene	DET0078	No gene	No gene	No gene	trichloroethene reductive dehalogenase anchoring protein, putative	No gene	No gene	RdhB	RDR1, RDR2
516	No gene	DET0876	No gene	No gene	No gene	reductive dehalogenase, putative	No gene	No gene	RdhA	RDR1, RDR2
517	No gene	DET1528	No gene	No gene	No gene	reductive dehalogenase, putative	No gene	No gene	RdhA	RDR1, RDR2
518	No gene	DET0163	No gene	No gene	No gene	reductive dehalogenase anchoring protein, putative	No gene	No gene	RdhB	RDR1, RDR2
519	No gene	DET0175	No gene	No gene	No gene	reductive dehalogenase anchoring protein, putative	No gene	No gene	RdhB	RDR1, RDR2

Model gene number	CBDB1 unique metabolic genes	195 unique metabolic genes	BAV1 unique metabolic genes	VS unique metabolic genes	CBDB1 annotation	195 annotation	BAV1 annotation	VS annotation	Model protein	Model reaction
520	No gene	DET0236	No gene	No gene	No gene	reductive dehalogenase anchoring protein, putative	No gene	No gene	RdhB	RDR1, RDR2
521	No gene	DET0875	No gene	No gene	No gene	reductive dehalogenase anchoring protein, putative	No gene	No gene	RdhB	RDR1, RDR2
522	No gene	DET1527	No gene	No gene	No gene	reductive dehalogenase anchoring protein, putative	No gene	No gene	RdhB	RDR1, RDR2
523	No gene	DET1558	No gene	No gene	No gene	reductive dehalogenase anchoring protein, putative	No gene	No gene	RdhB	RDR1, RDR2
524	No gene	DET1174	No gene	No gene	No gene	Fec-type ABC transporter, periplasmic iron-binding protein	No gene	No gene	BtuF	CBL1abc
525	No gene	DET1175	No gene	No gene	No gene	Fec-type ABC transporter, permease protein	No gene	No gene	BtuC	CBL1abc
526	No gene	DET1176	No gene	No gene	No gene	Fec-type ABC transporter, ATP-binding protein	No gene	No gene	BtuD	CBL1abc
527	No gene	No gene	DehaBAV1_0118	No gene	No gene	No gene	hypothetical protein	No gene	RdhB	RDR1, RDR2
528	No gene	No gene	No gene	VS1260	No gene	No gene	No gene	reductive dehalogenase	RdhA	RDR1, RDR2
529	No gene	No gene	No gene	VS1349	No gene	No gene	No gene	reductive dehalogenase	RdhA	RDR1, RDR2

Model gene number	CBDB1 unique metabolic genes	195 unique metabolic genes	BAV1 unique metabolic genes	VS unique metabolic genes	CBDB1 annotation	195 annotation	BAV1 annotation	VS annotation	Model protein	Model reaction
530	No gene	No gene	No gene	VS1344	No gene	No gene	No gene	reductive dehalogenase	RdhA	RDR1, RDR2
531	No gene	No gene	No gene	VS1340	No gene	No gene	No gene	reductive dehalogenase	RdhA	RDR1, RDR2
532	No gene	No gene	No gene	VS1329	No gene	No gene	No gene	reductive dehalogenase	RdhA	RDR1, RDR2
533	No gene	No gene	No gene	VS1327	No gene	No gene	No gene	reductive dehalogenase	RdhA	RDR1, RDR2
534	No gene	No gene	No gene	VS1320	No gene	No gene	No gene	reductive dehalogenase	RdhA	RDR1, RDR2
535	No gene	No gene	No gene	VS1314	No gene	No gene	No gene	reductive dehalogenase	RdhA	RDR1, RDR2
536	No gene	No gene	No gene	VS82	No gene	No gene	No gene	reductive dehalogenase	RdhA	RDR1, RDR2
537	No gene	No gene	No gene	VS1261	No gene	No gene	No gene	putative reductive dehalogenase	RdhA	RDR1, RDR2
538	No gene	No gene	No gene	VS1382	No gene	No gene	No gene	putative reductive dehalogenase anchoring protein	RdhB	RDR1, RDR2
539	No gene	No gene	No gene	VS1370	No gene	No gene	No gene	putative reductive dehalogenase anchoring protein	RdhB	RDR1, RDR2

Model gene number	CBDB1 unique metabolic genes	195 unique metabolic genes	BAV1 unique metabolic genes	VS unique metabolic genes	CBDB1 annotation	195 annotation	BAV1 annotation	VS annotation	Model protein	Model reaction
540	No gene	No gene	No gene	VS1341	No gene	No gene	No gene	putative reductive dehalogenase anchoring protein	RdhB	RDR1, RDR2
541	No gene	No gene	No gene	VS1339	No gene	No gene	No gene	putative reductive dehalogenase anchoring protein	RdhB	RDR1, RDR2
542	No gene	No gene	No gene	VS1334	No gene	No gene	No gene	putative reductive dehalogenase anchoring protein	RdhB	RDR1, RDR2
543	No gene	No gene	No gene	VS1328	No gene	No gene	No gene	putative reductive dehalogenase anchoring protein	RdhB	RDR1, RDR2
544	No gene	No gene	No gene	VS1326	No gene	No gene	No gene	putative reductive dehalogenase anchoring protein	RdhB	RDR1, RDR2
545	No gene	No gene	No gene	VS1323	No gene	No gene	No gene	putative reductive dehalogenase anchoring protein	RdhB	RDR1, RDR2
546	No gene	No gene	No gene	VS1319	No gene	No gene	No gene	putative reductive dehalogenase anchoring protein	RdhB	RDR1, RDR2

Model gene number	CBDB1 unique metabolic genes	195 unique metabolic genes	BAV1 unique metabolic genes	VS unique metabolic genes	CBDB1 annotation	195 annotation	BAV1 annotation	VS annotation	Model protein	Model reaction
547	No gene	No gene	No gene	VS1313	No gene	No gene	No gene	putative reductive dehalogenase anchoring protein	RdhB	RDR1, RDR2
548	No gene	No gene	No gene	VS1307	No gene	No gene	No gene	putative reductive dehalogenase anchoring protein	RdhB	RDR1, RDR2
549	No gene	No gene	No gene	VS81	No gene	No gene	No gene	putative reductive dehalogenase anchoring protein	RdhB	RDR1, RDR2

Table 8. Detailed List of Proteins of *i* AI549

No	Abbreviation	Full Name	Model gene number	Reaction
1	AccC	acetyl-CoA carboxylase, biotin carboxylase	24+25	ACCOAC
2	Aco3	aconitate hydratase	219+218+94+93	MICITH, ACONT
3	AcpS	holo-[acyl-carrier-protein] synthase	90	ACPS1
4	Acs	acetyl-CoA synthetase	323	ACS
5	AdeC	adenine deaminase	208	ADD
6	AdhC	Alcohol dehydrogenase	15, 27	ALCD19, ALCD2x, ALCD3
7	Adk	adenylate kinase	111	DADK, ADNK1, ADK1, ADK2, ADK4, ADK3
8	AgaZ	tagatose 6-phosphate aldolase I	354	TGBPA
9	AhcY	adenosylhomocysteinase	116	AHC
10	AlaS	alanyl-tRNA synthetase	14	ALATRS
11	AlaT	alanine transaminase	351	ALATA_L
12	AldH	Aldehyde dehydrogenase	342	ALDD8b
13	AmtB	Ammonium transporter	291	NH4t, NH4t3
14	Apt	adenine phosphoribosyltransferase	311	ADPT
15	ArgA	N-acetylglutamate synthase	119, 100	ACGS
16	ArgB1	Acetylglutamate kinase	332	ACGK
17	ArgC	N-acetyl-g-glutamyl-phosphate reductase	405	AGPR
18	ArgD	N-acetylornithine aminotransferase	333	SDPTA, ACOTA
19	ArgF	ornithine carbamoyltransferase	362	OCBT
20	ArgG	argininosuccinate synthase	334	ARGSS
21	ArgH	argininosuccinate lyase	335	ARGSL
22	ArgJ	ornithine transacetylase	331	ORNTAC, ACGS
23	ArgS	Arginyl-tRNA synthetase	337	ARGTRS
24	AroA	3-phosphoshikimate 1-carboxyvinyltransferase	105	PSCVT
25	AroA2	phospho-2-dehydro-3-deoxyheptonate aldolase	378	DAHPS
26	AroB	3-dehydroquinate synthase	109, 72	DHQS
27	AroC1	Chorismate synthase	104	CHORS
28	AroD1	3-dehydroquinate dehydratase	108	DHQD
29	AroE1	Shikimate 5-dehydrogenase	107	SHK3D
30	AroG	phospho-2-dehydro-3-deoxyheptonate aldolase	110	DAHPS
31	AroK	shikimate kinase	106	SHKK
32	ArsB	Arsenical pump membrane protein	248	ARSt1

No	Abbreviation	Full Name	Model gene number	Reaction
33	ArsB2	Arsenical pump membrane protein	437	ANTIMt1, ARSNAt1, ARSt1
34	Asd	aspartate-semialdehyde dehydrogenase	276	ASAD
35	AsnA	asparagine synthetase A	42	ASNS2
36	AspA	aspartate ammonia-lyase	335	ASPT
37	AspC	aspartate aminotransferase	139, 351, 190	ASPTA1
38	AspK	Aspartate Kinase	407	ASPK
39	AspS	Aspartyl-tRNA synthetase	177	ASPTRS
40	AtpABCDEFGHI	ATP synthase	136+137+135+133+132+130+134+131	ATPS3r
41	AzlCD	branched-chain amino acid transporter	265+266+263+264+267	ILEabc, VALabc, LEUabc
42	BioA	adenosylmethionine-8-amino-7-oxononanoate aminotransferase	333	AMAOT
43	BioBec	Biotin synthase	306	BTS
44	BirA	Biotin protein ligase	235	BACCL
45	BraB	branched-chain amino acid transporter	261	ILEabc, LEUabc, VALabc
46	BtuC	ABC vitamin B12 transporter (membrane)	525, 162	CBL1abc
47	BtuD	ABC vitamin B12 transporter (atp_bind)	526, 163	CBL1abc
48	BtuF	ABC vitamin B12 transporter (peri_bind)	431, 524, 161, 480	CBL1abc
49	CarAB	carbamoyl-phosphate synthase	317+316	CBPSr
50	CbiB	Adenosyl cobinamide synthase	164	ADCPS2, ADCOBAS
51	CbiD	Cobalamin biosynthetic protein CbiD	430, 300	ADCOBAS, ADCPS2
52	CbiE	precorrin-6Y C5,15-methyltransferase	416	CPC6MT
53	CbiZ	Adenosylcobinamide hydrolase	413	ADCOBHS
54	RdhA	Reductive dehalogenase	537, 453, 51, 507, 304, 467, 514, 458, 443, 463, 535, 509, 532, 484, 486, 49, 469, 487, 505, 531, 534, 448, 511, 474, 461, 529, 427, 517, 471, 465, 40, 536, 425, 473, 478, 441, 533, 426, 445, 528, 516, 19, 439, 451, 513, 485, 530, 459, 456, 504, 503	RDR1, RDR2

No	Abbreviation	Full Name	Model gene number	Reaction
55	RdhB	Reductive dehalogenase anchoring protein	519, 472, 527, 447, 523, 539, 499, 449, 506, 470, 468, 547, 452, 544, 538, 501, 540, 464, 50, 450, 520, 518, 440, 512, 522, 546, 442, 460, 444, 446, 482, 466, 508, 457, 462, 541, 542, 548, 549, 388, 41, 502, 521, 543, 545, 479, 515, 305, 510, 455	RDR1, RDR2
56	CdsA	phosphatidate cytidylyltransferase	59	PHCYT
57	CimA	Citramalate synthase	216	CITRMS
58	Cls	cardiolipin synthetase	279	CLPNS
59	Cmk	cytidylate kinase	105	CYTK2, CYTK1, UMPK
60	CoaA	pantothenate kinase	83	PNTK
61	CoaD	pantetheine-phosphate adenylyltransferase	43	PTPAT
62	CoaE	dephospho-CoA kinase	345	DPCOAK
63	CobA	cob(I)alamin adenosyltransferase	326, 301, 429	CBIAT, CYRDAAT, CBLATr
64	CobB	cobyrinic acid a,c-diamide synthase	29	CYRDAS
65	CobC	alpha-ribazole-5-phosphatase	168	RBZP
66	CobQ	cobyric acid synthase	260	CPC3MT, ADCOBHEXS
67	CobQ-CobD	cobyric acid synthase/L-threonine-O-3-phosphate decarboxylase	165	THRPD
68	CobS	cobalamin 5'-phosphate synthase	167	COBPS, COBPS2
69	CobT	nicotinate-nucleotide--dimethylbenzimidazole phosphoribosyltransferase (cobT)	166	NNDMBRT3, NNDMBRT
70	CobU	cobinamide kinase/cobinamide phosphate guanylyltransferase	169	ACBIPGT, ADCOBAK
71	ComEB	dCMP deaminase	76	DCMPDA2ir, DCMPDA3ir
72	CooS	carbon monoxide dehydrogenase	171, 172, 170, 174	CODHr
73	CorA1	Magnesium and cobalt transport protein CorA	82, 142	MGt5, COBALTt5
74	Cudp	copper translocating ATPase	271	Cuabc
75	CyaA	Adenylate cyclase	152	ADNCYC
76	CysAP	Sulfate transport ATP-binding protein cysA	213	TSULabc, SULabc
77	CysE	serine acetyltransferase	398	SERAT
78	CysH	phosphoadenylyl-sulfate reductase	343, 283, 282, 237	SADT2
79	CysKec	Cysteine synthase A	299	CYSS

No	Abbreviation	Full Name	Model gene number	Reaction
80	CysS	cysteinyl-tRNA synthetase	18	CYSTRS
81	DapA	dihydrodipicolinate synthase	277	DHDPS
82	DapAT	diaminopimelate aminotransferase	190	DAPAT
83	DapB	dihydrodipicolinate reductase	275	DHDPRy
84	DapD	tetrahydropicolinate succinylase	121	THDPS
85	DapE	succinyl-diaminopimelate desuccinylase	127, 198	SDPDS
86	DapF	diaminopimelate epimerase	191	DAPE
87	Ddl	D-alanyl-D-alanine ligase	154	ALAALA
88	Def	polypeptide deformylase	199	FMETDF
89	Dfp	phosphopantothenoylcysteine decarboxylase	84	PPCDC, PPNCL
90	DfrA	dihydrofolate reductase	214	DHFOR2, DHFOR3, DHFR
91	DgkA	Diacylglycerol kinase	77	DAGK
92	Dgt	dGTPase	128, 500	NTPTP2, DGTPH
93	Dutec	dUTP pyrophosphatase	78	DUTPDP
94	Dxr	1-deoxy-D-xylulose-5-phosphate reductoisomerase	58	DXPRI
95	Dxs	1-deoxyxylulose-5-phosphate synthase	195	DXPS
96	Eno	enolase	144	ENO
97	Epd	D-erythrose 4-phosphate dehydrogenase	143	E4PD
98	FabD	malonyl CoA-acyl carrier protein transacylase	339	MACPMT
99	FabF	beta-ketoacyl-acyl carrier protein synthase II	274	MCMAT8, MCMAT6, MCMAT4, MCMAT5, MCMAT2, MCMAT7, MCMAT9, AACPAT, ACMAT1, MCMAT3
100	FabG1	3-oxoacyl-[acyl-carrier-protein] reductase	340, 189	HOCR3, HDER4, HDDR5, HTDR6, HBUR1, HHDR7, HICR9, HODR8_#1, HHYR2
101	FabI	enoyl-(acyl-carrier-protein) reductase	338	KAS17, DEMAT4, OCMAT3, OCDMAT8, DDMAT5, HDMAT7, ICSMAT9, KAS11, BTMAT1, TDMAT6, HEMAT2
102	FabZ	(3R)-hydroxymyriostoyl-(acyl carrier protein) dehydratase	339	HHYHL2, HDDHL5, HODHL8n, HTDHL6, HOCHL3, HICHL9, HBUHL1, HDEHL4, HHDHL7
103	FadD	Long-chain-fatty-acid--CoA ligase	268, 286	FACOAL180, FACOAL170(ISO), FACOAL200, FACOAL160, FACOAL140, FACOAL181, FACOAL120
104	FatB	Acyl ACP thioesterase	339	FA140ACPH, FA120ACPH, FA200ACPH, FA160ACPH, FA180ACPH
105	Fba	fructose-1,6-bisphosphate aldolase	354	FBA

No	Abbreviation	Full Name	Model gene number	Reaction
106	Fbp	fructose-1,6-bisphosphatase	327	FBP
107	Fdx	Ferredoxin	44+47+488+243+187+37	FRHD
108	FeoB1	ferrous iron transport protein B	387	FE2abc
109	Fhs	Formate--tetrahydrofolate ligase	175	MTHFC, FTHFLr, MTHFD
110	Fmt	Methionyl-tRNA formyltransferase	411	FMETTRS
111	FolB	dihydroneopterin aldolase	401, 402	DHNPA
112	FolC	folylpolyglutamate synthase	7	DHFS
113	FolD	methylenetetrahydrofolate dehydrogenase / methenyltetrahydrofolate cyclohydrolase	173	MTHFD, MTHFC
114	FolE	GTP cyclohydrolase I	319	GTPCI
115	FolK	7,8-dihydro-6-hydroxymethylpterin pyrophosphokinase	402	HPPK
116	FolP	dihydropteroate synthase	401	DHPS3, DHPS1
117	Frd	fumarate reductase, cytoplasmic	420	FRD5
118	FrdH	Ferredoxin hydrogenase	23+20+22+21+483+150+240+246+245+238+239	FRHD
119	FruK	fructose 1-phosphate kinase	355	F1PK
120	FucA	L-fuculose phosphate aldolase	410	FCLPA
121	FumB	fumarase B (fumarate hydratase, Class I)	98	FUM
122	GabT	4-aminobutyrate aminotransferase	333	ABTA
123	GalE	UDP-glucose 4-epimerase	475, 477, 346, 476	UDPG4E
124	GalF	glucose-1-phosphate uridyltransferase	121, 122	GALU
125	Gap	glyceraldehyde-3-phosphate dehydrogenase	143	GAPD
126	GatABC	glutamyl-tRNA(Gln) amidotransferase	350+390+349	GLUADT
127	GlcK	Glucokinase	13	HEX1
128	GlgA1	Glycogen synthase	284, 280	GLCS1
129	GlgC	glucose-1-phosphate adenylyltransferase	215, 322, 89, 481	GLGC
130	GlmM	phosphoglucosamine mutase	114, 120	PGAMT
131	GlmS	glutamine-fructose-6-phosphate transaminase	123	GF6PTA
132	GlmU	Glucosamine-1-phosphate acetyltransferase/ UDP-N-acetylglucosamine pyrophosphorylase	121, 122	UAGDP, G1PACT
133	GlnA	glutamine synthetase	290, 454	GLNS
134	GlnH	Glutamine-binding protein GlnH	81	GLNabc
135	GlnP	Glutamine transport system permease protein GlnP	80	GLNabc

No	Abbreviation	Full Name	Model gene number	Reaction
136	GlnQ	Glutamine transport ATP-binding protein GlnQ	79	GLNabc
137	GlnS	glutaminyl-tRNA synthetase	357	GLNTRS
138	GloB	hydroxyacylglutathione hydrolase	88, 205	GLYOX
139	GltAB	glutamate synthase	298, 296	GLUSx
140	GltB	Glutamate synthase [nadph] small chain	26, 295, 244	GLUSy
141	GltBD	Glutamate synthase [nadph]	293+292+11	GLUSy
142	GltX	glutamyl-tRNA synthetase	357	GLUTRS
143	GlyA	glycine hydroxymethyltransferase	87	ALATA_D2, ALATA_L2, GHMT
144	GlyQS	glycine-tRNA synthetase	206	GLYTRS
145	GmhA	Phosphoheptose isomerase	424	S7PI
146	Gmk	guanylate kinase	9	DGK1, GK1
147	GpmA	Phosphoglycerate mutase	372	PGM
148	GpsA	NAD(P)H-dependent glycerol-3-phosphate dehydrogenase	363	G3PD2, G3PD1
149	GuaA	GMP synthase	225	GMPS2
150	GuaB	IMP dehydrogenase	65	IMPD
151	GuaC	GMP reductase	65	GMPR
152	HemD	uroporphyrinogen III cosynthase	412	UPP3S, UPP3MT
153	HemN	coproporphyrinogen III oxidase	236	CPPPGOAN2
154	HepT	Heptaprenyl diphosphate synthase component II	67	PPTT, OCTT, HEPTT, GGTT, FRTT, HEXTT
155	HisA	phosphoribosylformimino-5-aminoimidazole carboxamide ribotide isomerase	348	PRMICI
156	HisB	imidazoleglycerol-phosphate dehydratase	230	IGPDH
157	HisC	histidinol-phosphate aminotransferase / tyrosine and phenylalanine aminotransferase	231	PHETA1, HSTPT, TYRTA
158	HisD	histidinol dehydrogenase	232	HISTD
159	HisF	imidazoleglycerol-phosphate synthase	294, 273, 297	IG3PS
160	HisG	ATP phosphoribosyltransferase	233	ATPPRT
161	HisI_N	phosphoribosyl-AMP cyclohydrolase	347	PRAMPC, PRATPP
162	HisJ	histidinol phosphate phosphatase	425	HISTP
163	HisS	histidyl-tRNA synthetase	3	HISTRS
164	HisS2	Histidyl-tRNA synthetase	234	HISTRS
165	Hom	homoserine dehydrogenase	321	HSDy
166	Hpt	Hypoxanthine-guanine phosphoribosyltransferase	196, 209	GUAPRT, HXPRT

No	Abbreviation	Full Name	Model gene number	Reaction
167	Icd	isocitrate dehydrogenase	95	ICDHy
168	IleS	Isoleucyl-tRNA synthetase	288	ILETRS
169	IlvA	L-threonine deaminase	320	THRD_L, SERD_Lr
170	IlvBH	acetolactate synthase	222+223	ACLS, ACHBS
171	IlvC	ketol-acid reductoisomerase	221	KARA1, KARA2
172	IlvD	dihydroxy-acid dehydratase	224	DHAD2, DHAD1, DHAD3
173	IlvE	branched-chain amino acid aminotransferase	5	VALTA, ILETA, LEUTA
174	IorAB1	Indolepyruvate ferredoxin oxidoreductase	269+270	IOR, IOR3, IOR2
175	IscS	cysteine desulfurase	417	THZPSN
176	IspA	Geranyltranstransferase	67	GRTT, DMATT
177	IspD	2-C-methyl-D-erythritol 4-phosphate cytidylyltransferase	16	MEPCT
178	IspE	4-(cytidine 5'-diphospho)-2-C-methyl-D-erythritol kinase	74	CDPMEK
179	IspF	2-C-methyl-D-erythritol 2,4-cyclodiphosphate synthase	17	MECDPS
180	IspG	1-hydroxy-2-methyl-2-(E)-butenyl 4-diphosphate synthase	57	MECDPDH
181	IxpP	dITP/XTP pyrophosphatase	328	ITPASE, XTPASE
182	KamA	lysine 2,3-aminomutase	48, 153	LYSAM
183	KefC	Glutathione-regulated potassium-efflux system protein KefC	406	Kt6
184	KefC2	Glutathione-regulated potassium-efflux system protein KefC	180	Kt6
185	Ldh	L-lactate dehydrogenase	96	LDH_L
186	LeuA	2-isopropylmalate synthase	220, 216	IPPS
187	LeuB	3-isopropylmalate dehydrogenase	217	IPMD, IPMDH
188	LeuCD	3-isopropylmalate dehydratase	219+218+94+93	IPPMIb, IPPMIa, IPMI2, IPMI1
189	LeuS	Leucyl-tRNA synthetase	46	LEUTRS
190	LpdA	dihydrolipoamide dehydrogenase	188	GCCc
191	LpxB	lipid A disaccharide synthase (lpxB)	176	LPADSS
192	LtaA	threonine aldolase	415	THRA, THRLAD
193	Lys1	Saccharopine dehydrogenase (NAD, L-lysine forming)	4	SACCD2
194	LysA	diaminopimelate decarboxylase	124	DAPDC
195	LysS	Lysyl-tRNA synthetase	141, 247	LYSTRS

No	Abbreviation	Full Name	Model gene number	Reaction
196	LytB	isoprenyl-pyrophosphate synthase and dimethylallyl diphosphate	353	IPDPS, DMPPS
197	ManA	mannose-6-phosphate isomerase	113	MAN6PI
198	ManC	mannose 1-phosphate guanyltransferase	75	MAN1PT2
199	MazG	Nucleotide triphosphate pyrophosphohydrolase	409	NTPP2, NTPP5, NTPP6, NTPP4, NTPP3, NTPP8, NTPP1, NTPP7
200	Mdh	malate dehydrogenase	96	MDH
201	MenA	1,4-dihydroxy-2-naphthoate octaprenyltransferase	73	DHNAOT
202	MerA	mercuric reductase	188	HGR
203	MetA1	homoserine O-acetyltransferase	7	HSAT
204	MetG	methionyl-tRNA synthetase	66	METTRS
205	MetK	S-adenosylmethionine synthetase	115	METAT
206	MgsA	methylglyoxal synthase	397, 30	MGSA
207	ModABC	molybdenum ABC transporter	496+498+497	MOBDabc
208	Mpg	mannose-1-phosphate guanyltransferase	322, 481	MAN1PT1
209	MpgSP	Mannosyl-3-phosphoglycerate synthase/mannosyl-3-phosphoglycerate phosphatase	356	MPGSP
210	MsrA	methionine sulfoxide reductase	438, 330	METSR-S1, METSR-S2
211	MtaP	5'-methylthioadenosine phosphorylase	117	MTAP
212	MurAA	UDP-N-acetylglucosamine 1-carboxyvinyltransferase	312	UAGCVT
213	MurB	UDP-N-acetylenolpyruvoylglucosamine reductase	154	UAPGR
214	NadA	quinolinate synthetase	399	QULNS
215	NadB	L-aspartate oxidase	420	ASPO1, ASPO6, ASPO4, ASPO3, ASPO5
216	NadC1	nicotinate-nucleotide pyrophosphorylase	360	NNDPR
217	NadD	Nicotinate-nucleotide adenlyltransferase	1	NNATr, NMNATr
218	NadE	NH3-dependent NAD+ synthetase	302	NADS1
219	NadE2	Glutamine-dependent NAD(+) synthetase	289	NADS2
220	Nadhd5	NADH dehydrogenase	149+249+259+254+255+256+251+252+258+257+253+250+242+241+243+393+394+396+391+392+395+91+186+185+187+37+35+36+38	NADH4, NADH7, NADH8, NADH6
221	NadK	NAD Kinase	101	NADK
222	Nadphd	NADPH dehydrogenase	37+243+187	NADPH12

No	Abbreviation	Full Name	Model gene number	Reaction
223	NasDE	nitrite reductase	296	NTRIRy, NTRIRx
224	Ndk	nucleoside diphosphate kinase	68	NDPK6, NDPK1, NDPK3, NDPK5, NDPK8, NDPK4, NDPK7, NDPK2
225	Nif	Nitrogenase	495, 492, 493, 490, 489, 491, 494	NITr
226	NifV	homocitrate synthase	404	HCITS
227	NikA	nickel transport system substrate-binding protein A	138, 436, 382	NIabc
228	NikB	nickel transport system substrate-binding protein B	383, 435	NIabc
229	NikC	nickel transport system substrate-binding protein C	434, 384	NIabc
230	NikD	nickel transport system substrate-binding protein D	433, 385	NIabc
231	NikE	nickel transport system substrate-binding protein E	432, 386	NIabc
232	NrdD	ribonucleoside-triphosphate reductase	52, 364	RNTR3, RNTR4, RNTR1, RNTR2
233	NrdEF	ribonucleoside-diphosphate reductase	428, 151, 52	RNDR3, RNDR2, RNDR4, RNDR1
234	NtpA	Dihydroneopterin triphosphate pyrophosphorylase	125, 336, 312	DNMPPA, DNTPPA
235	NudF	ADP-ribose diphosphatase	99	ADPRDP
236	OadD	Oxaloacetate decarboxylase	24	OXAD2
237	Ocd	ornithine cyclodeaminase	414	ORNCD
238	Ofo	2-oxoglutarate synthase	181+182+183+184	OOR2r
239	P_1.18.1.2	ferredoxin-NADP reductase	11	FNOR2
240	P_3.7.1.5	acylpyruvate hydrolase	325	APYRH
241	PaaK	phenylacetyl-CoA ligase	285, 262	PACCOAL
242	PabABC	para-aminobenzoate synthase component I /4-amino-4-deoxychorismate lyase	373	ADCS, ADCL
243	PanB	3-methyl-2-oxobutanoate hydroxymethyltransferase	211	MOHMT
244	PanC	pantothenate synthase	212	PANTS
245	PanD	aspartate 1-decarboxylase	210	ASP1DC
246	PcaC2	4-carboxymuconolactone decarboxylase	39	CMLDC
247	PdhAB	pyruvate dehydrogenase (E1 subunit)	183	PDH
248	PdxAJ	Pyridoxine 5-phosphate biosynthetic protein	64	PDX5PS
249	PdxHec	Pyridoxine 5-phosphate oxidase	418	PDX5PO, PYAM5PO
250	PfkB	6-phosphofructokinase II	355	PFK, PFK_2

No	Abbreviation	Full Name	Model gene number	Reaction
251	PflC	pyruvate formate lyase II	422	OBTFL, PFLi
252	Pfor	Pyruvic-ferredoxin oxidoreductase	181+182+183+184	POR2
253	Pgi	glucose-6-phosphate isomerase	113	PGI
254	Pgk	phosphoglycerate kinase	194	PGK
255	PgmI	Phosphoglycerate mutase, cofactor independent	408, 193	PGM
256	PgpA	Phosphatidylglycerophosphate phosphohydrolase	361	PGPPH
257	PgsA	CDPdiacylglycerol-glycerol-3-phosphate 3-phosphatidyltransferase	279	CDGPT
258	PheA	prephenate dehydratase	103, 389	PPNDH, CHORM
259	PheRS	phenylalanyl-tRNA synthetase	53+54	PHETRS
260	Phyd	Pantetheine hydrolase (ECC 3.5.1.-)	127	PNTEH
261	PitA	Low-affinity inorganic phosphate transporter	8	PIt6
262	Pmdpht	Pyrimidine phosphatase	361	PMDPHT
263	Pmgm1	phosphoglucomutase/phosphomannomutase	120, 114	PMANM, PGMT
264	PncB	nicotinate phosphoribosyltransferase	370	NAMNPP, NCTPPRT
265	Ppa	inorganic pyrophosphatase	55	PPA
266	Ppa1	V-type H(+)-translocating pyrophosphatase	200, 204	PPA_1
267	PpdK	pyruvate phosphate dikinase	129	PPDK
268	Ppsa	phosphoenolpyruvate synthase	129	PPS
269	ProA	glutamate-5-semialdehyde dehydrogenase	342	G5SD
270	ProB	glutamate 5-kinase	341	GLU5K
271	ProC	pyrroline-5-carboxylate reductase	45	P5CR
272	ProPec	MFS, proline proton symport	344	PROt6, GLYBt6
273	ProS	Prolyl-tRNA synthetase	56	PROTRS
274	Prs	phosphoribosylpyrophosphate synthetase	86	PRPPS
275	PssA1	CDP-diacylglycerol-serine O-phosphatidyltransferase	279	CDPDSP
276	PstABCS	phosphate ABC transporter	34+31+33+32	PIabc
277	Ptps	6-pyruvoyltetrahydropterin synthase	403	PTHPS
278	PurA	adenylosuccinate synthetase	278	ADSS
279	PurB	adenylosuccinate lyase	228	ADSL1, ADSL2
280	PurC	phosphoribosylaminoimidazolesuccinocarboxamide synthase	229	PRASCS
281	PurD	phosphoribosylglycinamide synthetase	226	PRAGS

No	Abbreviation	Full Name	Model gene number	Reaction
282	PurE	phosphoribosylaminoimidazole carboxylase	227	AIRC
283	PurF	glutamine phosphoribosyldiphosphate amidotransferase	367	GLUPRT
284	PurH	phosphoribosylaminoimidazole carboxy formyl formyltransferase / inosine-monophosphate cyclohydrolase	369	AICART, IMPCir
285	PurL	phosphoribosylformylglycinamidine synthase II	63	PRFGS
286	PurM	phosphoribosylaminoimidazole synthetase	368	PRAIS
287	PurN	phosphoribosylglycinamide formyltransferase	179, 329	GARFT
288	PurQ	Phosphoribosylformylglycinamidine synthase I	62	PRFGS
289	PurUec	Formyltetrahydrofolate deformylase	329	FTHFD
290	PuuT	putrescine:2-oxoglutaric acid aminotransferase	333	ORNTA
291	Pyc	pyruvate carboxylase	24+25	PC
292	Pyk	pyruvate kinase	129	PYK
293	PyrB	aspartate carbamoyltransferase	314	ASPCT
294	PyrC	dihydroorotase	315	DHORTS
295	PyrD	Dihydroorotate dehydrogenase	358	DHORDf
296	PyrE	orotate phosphoribosyltransferase	311	ORPT
297	PyrF	orotidine 5'-phosphate decarboxylase	6	OMPDC
298	PyrG	CTP synthase	366	CTPS1, CTPS2
299	PyrH	uridylate kinase	61	URIDK2, URIDK1
300	PyrK	Dihydroorotate dehydrogenase electron transfer subunit	10, 318	DHORDf
301	RbsK	ribokinase	178	RBK
302	RelA	GTP pyrophosphokinase	2	GTPDK
303	RfbA	glucose-1-phosphate thymidylyltransferase	121, 122	G1PTMT
304	RibA	GTP cyclohydrolase II / 3,4-dihydroxy-2-butanone 4-phosphate synthase	308	DB4PS, GTPCII
305	RibD	diaminohydroxyphosphoribosylaminopyrimidine deaminase	310	DHPPDA2, APRAUR
306	RibEH	riboflavin synthase	307+309	RBFSa, RBFSb
307	RibF	Riboflavin kinase / FMN adenylyltransferase	148	FMNAT, RBFK
308	RluB	Pseudouridine synthase	112, 207, 281	PSUDS

No	Abbreviation	Full Name	Model gene number	Reaction
309	RluD	ribosomal large subunit pseudouridine synthase D	359	PSUDS
310	RpiB	Ribose 5-phosphate isomerase	159	RPI
311	SerA2	D-3-phosphoglycerate dehydrogenase	145	PGCD, PGCDr
312	SerB	phosphoserine phosphatase	324	PSP_L
313	SerC	phosphoserine transaminase	146	PSERTr
314	SerS	Seryl-tRNA synthetase	140	SERTRS
315	SgcE	putative ribulose-phosphate 3-epimerase	158	RPE
316	SodA	Superoxide dismutase (mn)	272	SOD
317	SpeA	arginine decarboxylase	124	ARGDC
318	SpeC	Ornithine decarboxylase	124	ORNDC
319	SucCD1	Succinyl CoA synthetase	419	SUCOAS
320	Tal	Transaldolase	365	TAL
321	ThiC	biosynthesis of the pyrimidine moiety of thiamin	201	AHMMPS, AMPMS
322	ThiD	phosphomethylpyrimidine kinase	421	PMPK
323	ThiE	thiamine-phosphate pyrophosphorylase	203	HMPK4, HMPK3, TMPPP, HMPK1, HMPK2
324	ThiL	thiamine-monophosphate kinase	69	TMPKr
325	ThrB	homoserine kinase	303, 74	HSK
326	ThrC	threonine synthase	320	THRS
327	ThrS	Threonyl-tRNA synthetase	197	THRTRS
328	ThyA	thymidylate synthase	214	TMDS
329	ThyX	Thymidylate synthase (FAD)	381	FDTS
330	TktB	Transketolase	160	TKT2, TKT1
331	Tmk	thymidylate kinase	202	DTMPK, TMDK1
332	TpiA	triose phosphate isomerase	192	TPI
333	TrpA	Tryptophan synthase alpha chain	380	TRPS1
334	TrpB	Tryptophan synthase beta chain	379, 287	TRPS1
335	TrpC	indole-3-glycerol-phosphate synthase	376	IGPS
336	TrpD	anthranilate phosphoribosyltransferase/anthranilate component II	375, 28	ANPRT
337	TrpE	anthranilate synthase component I	373	ANS1
338	TrpF	phosphoribosyl anthranilate isomerase	377	PRAI
339	TrpG	Anthranilate synthase component II	374	ANS1
340	TrpS	tryptophanyl-tRNA synthetase	352	TRPTRS
341	TrxB	thioredoxin reductase	126	TRDR

No	Abbreviation	Full Name	Model gene number	Reaction
342	TtdA	Fumarate hydratase	97	FUM
343	TyrA	prephenate dehydrogenase	102	PPND
344	TyrS	tyrosyl-tRNA synthetase	147	TYRTRS
345	UbiE	ubiquinone biosynthesis methyltransferase UbiE	12, 71, 400, 70	OMBZLM
346	UbiG	3-demethylubiquinone-9 3-methyltransferase	371	DMQMT, OHPHM
347	Upp2	uracil phosphoribosyltransferase/ pyrimidine operon regulatory protein pyrr	313	UPPRT
348	UppS	undecaprenyl-diphosphate synthase	60	UDPDPS
349	ValS	Valyl-tRNA synthetase	85	VALTRS
350	XylA	xylose isomerase	299	XYLI2i
351	YkrS	5-methylthioribose kinase	118	MTRI
352	YlbQ	2-dehydropantoate 2-reductase	221, 102	DPR
353	YngE	Propionyl-CoA carboxylase	92	PPCOAC
354	ZnuA	High-affinity zinc uptake system protein znuA precursor	155	ZN2abc2, MNabc
355	ZnuB	High-affinity zinc uptake system membrane protein ZnuB	157	ZN2abc2, MNabc
356	ZnuC	High-affinity zinc uptake system atp-binding protein ZnuC	156	ZN2abc2, MNabc

Table 9. List of Reactions of *i* AI549 Associated with Core Genes (Core Reactions)

No	Abbreviation	Name	Equation	Subsystem	Model gene number	Protein	Protein Classification
1	ACGK	acetylglutamate kinase	[c] : acglu + atp --> acg5p + adp	Amino Acid Metabolism	332	ArgB1	EC-2.7.2.8
2	ACGS	N-acetylglutamate synthase	[c] : accoa + glu-L --> acglu + coa + h	Amino Acid Metabolism	331, 119, 100	ArgA, ArgJ	EC-2.3.1.1
3	ACHBS	2-aceto-2-hydroxybutanoate synthase	[c] : 2obut + h + pyr --> 2ahbut + co2	Amino Acid Metabolism	222+223	IlvBH	
4	ACLS	acetolactate synthase (Also catalyzes ACHBS)	[c] : h + (2) pyr --> alac-S + co2	Amino Acid Metabolism	222+223	IlvBH	EC-4.1.3.18
5	ACOTA	acetylornithine transaminase	[c] : acorn + akg <==> acg5sa + glu-L	Amino Acid Metabolism	333	ArgD	EC-2.6.1.11
6	AGPR	N-acetyl-g-glutamyl-phosphate reductase	[c] : acg5sa + nadp + pi <==> acg5p + h + nadph	Amino Acid Metabolism	405	ArgC	EC-1.2.1.38
7	ALAALA	D-alanine-D-alanine ligase (reversible)	[c] : (2) ala-D + atp <==> adp + alaala + h + pi	Amino Acid Metabolism	154	Ddl	EC-6.3.2.4
8	ALATA_L	L-alanine transaminase	[c] : akg + ala-L <==> glu-L + pyr	Amino Acid Metabolism	351	AlaT	EC-2.6.1.2
9	ALATRS	Alanyl-tRNA synthetase	[c] : ala-L + atp + trnaala --> alatrna + amp + ppi	Amino Acid Metabolism	14	AlaS	EC-6.1.1.7
10	ANPRT	anthranilate phosphoribosyltransferase	[c] : anth + prpp --> ppi + pran	Amino Acid Metabolism	375, 28	TrpD	EC-2.4.2.18
11	ANS1	anthranilate synthase	[c] : chor + gln-L --> anth + glu-L + h + pyr	Amino Acid Metabolism	373, 374	TrpG+TrpE	EC-4.1.3.27
12	APYRH	3-Fumarylpyruvate acylhydrolase	[c] : 3fpyr + h2o --> fum + h + pyr	Amino Acid Metabolism	325	P_3.7.1.5	EC-3.7.1.5
13	ARGDC	arginine decarboxylase	[c] : arg-L + h --> agm + co2	Amino Acid Metabolism	124	SpeA	EC-4.1.1.19
14	ARGSL	argininosuccinate lyase	[c] : argsuc <==> arg-L + fum	Amino Acid Metabolism	335	ArgH	EC-4.3.2.1
15	ARGSS	argininosuccinate synthase	[c] : asp-L + atp + citr-L --> amp + argsuc + h + ppi	Amino Acid Metabolism	334	ArgG	EC-6.3.4.5
16	ARGTRS	Arginyl-tRNA synthetase	[c] : arg-L + atp + trnaarg --> amp + argtrna + ppi	Amino Acid Metabolism	337	ArgS	EC-6.1.1.19
17	ASAD	aspartate-semialdehyde dehydrogenase	[c] : aspsa + nadp + pi <==> 4pasp + h + nadph	Amino Acid Metabolism	276	Asd	EC-1.2.1.11
18	ASNS2	asparagine synthetase	[c] : asp-L + atp + nh4 --> amp + asn-L + h + ppi	Amino Acid Metabolism	42	AsnA	EC-6.3.1.1
19	ASPK	aspartate kinase	[c] : asp-L + atp <==> 4pasp + adp	Amino Acid Metabolism	407	AspK	EC-2.7.2.4
20	ASPT	L-aspartase	[c] : asp-L --> fum + nh4	Amino Acid Metabolism	335	AspA	EC-4.3.1.1

No	Abbreviation	Name	Equation	Subsystem	Model gene number	Protein	Protein Classification
21	ASPTA1	aspartate transaminase	[c] : akg + asp-L <==> glu-L + oaa	Amino Acid Metabolism	139, 351, 190	AspC	EC-2.6.1.1
22	ASPTRS	Aspartyl-tRNA synthetase	[c] : asp-L + atp + trnaasp --> amp + asptrna + ppi	Amino Acid Metabolism	177	AspS	EC-6.1.1.12
23	ATPPRT	ATP phosphoribosyltransferase	[c] : atp + prpp --> ppi + prbatp	Amino Acid Metabolism	233	HisG	EC-2.4.2.17
24	CHORM	chorismate mutase	[c] : chor --> pphn	Amino Acid Metabolism	103, 389	PheA	EC-5.4.99.5
25	CHORS	chorismate synthase	[c] : 3psme --> chor + pi	Amino Acid Metabolism	104	AroC1	EC-4.2.3.5
26	CITRMS	(R)-citramalate synthase	[c] : accoa + h + h2o + pyr <==> citrmal + coa	Amino Acid Metabolism	216	CimA	EC-2.3.1
27	CYSS	cysteine synthase	[c] : acser + h2s --> ac + cys-L	Amino Acid Metabolism	299	CysKec	EC-4.2.99.8
28	CYSTRS	Cysteinyl-tRNA synthetase	[c] : atp + cys-L + trnacys --> amp + cystrna + ppi	Amino Acid Metabolism	18	CysS	EC-6.1.1.16
29	DAHPS	3-deoxy-D-arabino-heptulosonate 7-phosphate synthetase	[c] : e4p + h2o + pep --> 2dda7p + pi	Amino Acid Metabolism	110, 378	AroG, AroA2	EC-4.1.2.15
30	DAPAT	LL-Diaminopimelate aminotransferase	[c] : 26dap-LL + akg <==> glu-L + h + h2o + thdp	Amino Acid Metabolism	190	DapAT	
31	DAPDC	diaminopimelate decarboxylase	[c] : 26dap-M + h --> co2 + lys-L	Amino Acid Metabolism	124	LysA	EC-4.1.1.20
32	DAPE	diaminopimelate epimerase	[c] : 26dap-LL <==> 26dap-M	Amino Acid Metabolism	191	DapF	EC-5.1.1.7
33	DHAD1	dihydroxy-acid dehydratase (2,3-dihydroxy-3-methylbutanoate)	[c] : 23dhmb --> 3mob + h2o	Amino Acid Metabolism	224	IlvD	EC-4.2.1.9
34	DHAD2	Dihydroxy-acid dehydratase (2,3-dihydroxy-3-methylpentanoate)	[c] : 23dhmp --> 3mop + h2o	Amino Acid Metabolism	224	IlvD	
35	DHAD3	dihydroxy-acid dehydratase (2,3-dihydroxy-3-methylpentanoate)	[c] : 23dhmp --> 2o3mpt + h2o	Amino Acid Metabolism	224	IlvD	EC-4.2.1.9
36	DHDPRy	dihydrodipicolinate reductase (NADPH)	[c] : 23dhdp + h + nadph --> nadp + thdp	Amino Acid Metabolism	275	DapB	EC-1.3.1.26
37	DHDPS	dihydrodipicolinate synthase	[c] : aspsa + pyr --> 23dhdp + h + (2) h2o	Amino Acid Metabolism	277	DapA	EC-4.2.1.52
38	DHQD	3-dehydroquinate dehydratase	[c] : 3dhq <==> 3dhsk + h2o	Amino Acid Metabolism	108	AroD1	EC-4.2.1.10

No	Abbreviation	Name	Equation	Subsystem	Model gene number	Protein	Protein Classification
39	DHQS	3-dehydroquinate synthase	[c] : 2dda7p --> 3dhq + pi	Amino Acid Metabolism	109, 72	AroB	EC-4.2.3.4
40	FMETDF	formylmethionine deformylase	[c] : fmet + h2o --> for + met-L	Amino Acid Metabolism	199	Def	EC-3.5.1.31
41	FMETTRS	Methionyl-tRNA formyltransferase	[c] : 10fthf + mettrna --> fmettrna + thf	Amino Acid Metabolism	411	Fmt	EC-2.1.2.9
42	FTHFLr	formate-tetrahydrofolate ligase	[c] : atp + for + thf <==> 10fthf + adp + pi	Amino Acid Metabolism	175	Fhs	EC-6.3.4.3
43	G5SD	glutamate-5-semialdehyde dehydrogenase	[c] : glu5p + h + nadph --> glu5sa + nadp + pi	Amino acid Metabolism	342	ProA	EC-1.2.1.41
44	GCCc	glycine-cleavage complex	[c] : dhlpro + nad --> h + lpro + nadh	Amino Acid Metabolism	188	LpdA	EC-1.8.1.4
45	GHMT	glycine hydroxymethyltransferase	[c] : ser-L + thf <==> gly + h2o + mlthf	Amino Acid Metabolism	87	GlyA	EC-2.1.2.1
46	GLNS	glutamine synthetase	[c] : atp + glu-L + nh4 --> adp + gln-L + h + pi	Amino Acid Metabolism	290, 454	GlnA	EC-6.3.1.2
47	GLNTRS	Glutaminyl-tRNA synthetase	[c] : atp + gln-L + trnagln --> amp + glntrna + ppi	Amino Acid Metabolism	357	GlnS	EC-6.1.1.18
48	GLU5K	glutamate 5-kinase	[c] : atp + glu-L --> adp + glu5p	Amino Acid Metabolism	341	ProB	EC-2.7.2.11
49	GLUADT	glutaminyl-tRNA synthase (glutamine-hydrolysing)	[c] : atp + gln-L + glutrna + h2o --> adp + glntrna + glu-L + h + pi	Amino Acid Metabolism	350+390+349	GatABC	
50	GLUSy	glutamate synthase (NADPH)	[c] : akg + gln-L + h + nadph --> (2) glu-L + nadp	Amino Acid Metabolism	26+295+244+293+292+11	GltBD, GltB	EC-1.4.1.13
51	GLUTRS	Glutamyl-tRNA synthetase	[c] : atp + glu-L + trnaglu --> amp + glutrna + ppi	Amino Acid Metabolism	357	GltX	EC-6.1.1.17
52	GLYTRS	Glycyl-tRNA synthetase	[c] : atp + gly + trnagly --> amp + glytrna + ppi	Amino Acid Metabolism	206	GlyQS	EC-6.1.1.14
53	HCITS	homocitrate synthase	[c] : accoa + akg + h2o --> coa + h + hcit	Amino Acid Metabolism	404	NifV	EC-4.1.3.21
54	HISTD	histidinol dehydrogenase	[c] : h2o + histd + (2) nad --> (3) h + his-L + (2) nadh	Amino Acid Metabolism	232	HisD	EC-1.1.1.23
55	HISTRS	Histidyl-tRNA synthetase	[c] : atp + his-L + trnahis --> amp + histrna + ppi	Amino Acid Metabolism	3, 234	HisS2, HisS	EC-6.1.1.21
56	HSAT	Acetyl-CoA:L-homoserine O-acetyltransferase	[c] : accoa + hom-L <==> achms + coa	Amino Acid Metabolism	7	MetA1	EC-2.3.1.31
57	HSDy	homoserine dehydrogenase (NADPH)	[c] : hom-L + nadp <==> aspsa + h + nadph	Amino Acid Metabolism	321	Hom	EC-1.1.1.3
58	HSK	homoserine kinase	[c] : atp + hom-L --> adp + h + phom	Amino Acid Metabolism	303, 74	ThrB	EC-2.7.1.39
59	HSTPT	histidinol-phosphate transaminase	[c] : glu-L + imacp --> akg + h + hisp	Amino Acid Metabolism	231	HisC	EC-2.6.1.9

No	Abbreviation	Name	Equation	Subsystem	Model gene number	Protein	Protein Classification
60	IG3PS	Imidazole-glycerol-3-phosphate synthase	[c] : gln-L + prlp --> aicar + eig3p + glu-L + (2) h	Amino Acid Metabolism	294, 273, 297	HisF	
61	IGPDH	imidazoleglycerol-phosphate dehydratase	[c] : eig3p + h --> h2o + imacp	Amino Acid Metabolism	230	HisB	EC-4.2.1.19
62	IGPS	indole-3-glycerol-phosphate synthase	[c] : 2cpr5p + h --> 3ig3p + co2 + h2o	Amino Acid Metabolism	376	TrpC	EC-4.1.1.48
63	ILETA	isoleucine transaminase	[c] : akg + ile-L <==> 3mop + glu-L	Amino Acid Metabolism	5	IlvE	EC-2.6.1.42
64	ILETRS	Isoleucyl-tRNA synthetase	[c] : atp + ile-L + trnaile --> amp + iletrna + ppi	Amino Acid Metabolism	288	IleS	EC-6.1.1.5
65	IPMD	3-isopropylmalate dehydrogenase	[c] : 3c2hmp + nad --> 3c4mop + h + nadh	Amino Acid Metabolism	217	LeuB	EC-1.1.1.85
66	IPMDH	Isopropylmalate dehydrogenase	[c] : de3mlt + nad <==> 2obut + co2 + (2) h + nadh	Amino Acid Metabolism	217	LeuB	
67	IPMI1	Isopropylmalate isomerase	[c] : citrmal <==> ccnt + h2o	Amino Acid Metabolism	219+218+94+93	LeuCD	EC-4.2.1.35
68	IPMI2	Isopropylmalate isomerase	[c] : ccnt + h2o <==> de3mlt	Amino Acid Metabolism	219+218+94+93	LeuCD	
69	IPPMIa	3-isopropylmalate dehydratase	[c] : 3c2hmp <==> 2ippm + h2o	Amino Acid Metabolism	219+218+94+93	LeuCD	EC-4.2.1.33
70	IPPMIb	2-isopropylmalate hydratase	[c] : 2ippm + h2o <==> 3c3hmp	Amino Acid Metabolism	219+218+94+93	LeuCD	
71	IPPS	2-isopropylmalate synthase	[c] : 3mob + accoa + h2o --> 3c3hmp + coa + h	Amino Acid Metabolism	220, 216	LeuA	EC-4.1.3.12
72	KARA1	ketol-acid reductoisomerase (2,3-dihydroxy-3-methylbutanoate)	[c] : 23dhmb + nadp <==> alac-S + h + nadph	Amino Acid Metabolism	221	IlvC	EC-1.1.1.86
73	KARA2	ketol-acid reductoisomerase (2-Acetolactate)	[c] : 2ahbut + h + nadph <==> 23dhmp + nadp	Amino Acid Metabolism	221	IlvC	EC-1.1.1.86
74	LEUTA	leucine transaminase	[c] : akg + leu-L <==> 4mop + glu-L	Amino Acid Metabolism	5	IlvE	EC-2.6.1.42
75	LEUTRS	Leucyl-tRNA synthetase	[c] : atp + leu-L + trnaleu --> amp + leutrna + ppi	Amino Acid Metabolism	46	LeuS	EC-6.1.1.4
76	LYSAM	lysine 2,3-aminomutase	[c] : lys-L <==> 36dahx	Amino Acid Metabolism	48, 153	KamA	EC-5.4.3.2
77	LYSTRS	Lysyl-tRNA synthetase	[c] : atp + lys-L + trnalys --> amp + lystrna + ppi	Amino Acid Metabolism	141, 247	LysS	EC-6.1.1.6
78	METAT	methionine adenosyltransferase	[c] : atp + h2o + met-L --> amet + pi + ppi	Amino Acid Metabolism	115	MetK	EC-2.5.1.6
79	METSR-S1	L-methionine-S-oxide reductase (trdrd)	[c] : metox + trdrd --> h2o + met-L + trdox	Amino Acid Metabolism	438, 330	MsrA	

No	Abbreviation	Name	Equation	Subsystem	Model gene number	Protein	Protein Classification
80	METSR-S2	L-methionine S-oxide reductase (H2O2)	[c] : h2o2 + met-L --> h2o + metox	Amino Acid Metabolism	438, 330	MsrA	
81	METTRS	Methionyl-tRNA synthetase	[c] : atp + met-L + trnamet --> amp + mettrna + ppi	Amino Acid Metabolism	66	MetG	EC-6.1.1.10
82	MTRI	5-methylthioribose-1-phosphate isomerase	[c] : 5mdr1p <==> 5mdru1p	Amino Acid Metabolism	118	YkrS	EC-5.3.1.23
83	OCBT	ornithine carbamoyltransferase	[c] : cbp + orn-L <==> citr-L + h + pi	Amino Acid Metabolism	362	ArgF	EC-2.1.3.3
84	ORNDC	ornithine decarboxylase	[c] : h + orn-L --> co2 + ptrc	Amino Acid Metabolism	124	SpeC	EC-4.1.1.17
85	ORNTA	ornithine transaminase	[c] : akg + orn-L --> glu-L + glu5sa	Amino Acid Metabolism	333	PuuT	EC-2.6.1.13
86	ORNTAC	ornithine transacetylase	[c] : acorn + glu-L <==> acglu + orn-L	Amino Acid Metabolism	331	ArgJ	EC-2.3.1.35
87	P5CR	pyrroline-5-carboxylate reductase	[c] : 1pyr5c + (2) h + nadph --> nadp + pro-L	Amino Acid Metabolism	45	ProC	EC-1.5.1.2
88	PGCD	phosphoglycerate dehydrogenase	[c] : 3pg + nad --> 3php + h + nadh	Amino Acid Metabolism	145	SerA2	EC-1.1.1.95
89	PGCDr	phosphoglycerate dehydrogenase	[c] : 3pg + nad <==> 3php + h + nadh	Amino Acid Metabolism	145	SerA2	EC-1.1.1.95
90	PHETA1	phenylalanine transaminase	[c] : akg + phe-L <==> glu-L + phpyr	Amino Acid Metabolism	231	HisC	EC-2.6.1.58
91	PHETRS	Phenylalanyl-tRNA synthetase	[c] : atp + phe-L + trnaphe --> amp + phetrna + ppi	Amino Acid Metabolism	53+54	PheRS	EC-6.1.1.20
92	PPND	prephenate dehydrogenase	[c] : nad + pphn --> 34hpp + co2 + nadh	Amino Acid Metabolism	102	TyrA	EC-1.3.1.12
93	PPNDH	prephenate dehydratase	[c] : h + pphn --> co2 + h2o + phpyr	Amino Acid Metabolism	103, 389	PheA	EC-4.2.1.51
94	PRAI	phosphoribosylanthranilate isomerase	[c] : pran <==> 2cpr5p	Amino Acid Metabolism	377	TrpF	EC-5.3.1.24
95	PRAMPC	phosphoribosyl-AMP cyclohydrolase	[c] : h + h2o + prbamp --> prfp	Amino Acid Metabolism	347	HisI_N	EC-3.5.4.19
96	PRATPP	phosphoribosyl-ATP pyrophosphatase	[c] : h2o + prbatp --> h + ppi + prbamp	Amino Acid Metabolism	347	HisI_N	EC-3.6.1.31
97	PRMICI	1-(5-phosphoribosyl)-5-[(5-phosphoribosylamino)methylideneamino)imidazole-4-carboxamide isomerase	[c] : prfp <==> prlp	Amino Acid Metabolism	348	HisA	EC-5.3.1.16
98	PROTRS	Prolyl-tRNA synthetase	[c] : atp + pro-L + trnapro --> amp + ppi + protrna	Amino Acid Metabolism	56	ProS	EC-6.1.1.15
99	PSCVT	3-phosphoshikimate 1-carboxyvinyltransferase	[c] : pep + skm5p <==> 3psme + pi	Amino Acid Metabolism	105	AroA	EC-2.5.1.19

No	Abbreviation	Name	Equation	Subsystem	Model gene number	Protein	Protein Classification
100	PSERTr	3-phosphoserine aminotransferase	[c] : 3php + glu-L <==> akg + pser-L	Amino Acid Metabolism	146	SerC	EC-2.6.1.52
101	PSP_L	phosphoserine phosphatase (L-serine)	[c] : h2o + pser-L --> pi + ser-L	Amino Acid Metabolism	324	SerB	EC-3.1.3.3
102	SACCD2	saccharopine dehydrogenase (NAD, L-lysine forming)	[c] : Lsacchrp + h2o + nad <==> akg + h + lys-L + nadh	Amino Acid Metabolism	4	Lys1	EC-1.5.1.7
103	SADT2	sulfate adenylyltransferase	[c] : atp + gtp + h2o + so4 --> aps + gdp + pi + ppi	Amino Acid Metabolism	343, 283, 282, 237	CysH	EC-2.7.7.4
104	SDPDS	succinyl-diaminopimelate desuccinylase	[c] : h2o + sl26da --> 26dap-LL + succ	Amino Acid Metabolism	127, 198	DapE	EC-3.5.1.18
105	SDPTA	succinyldiaminopimelate transaminase	[c] : akg + sl26da <==> glu-L + sl2a6o	Amino Acid Metabolism	333	ArgD	EC-2.6.1.17
106	SERAT	serine O-acetyltransferase	[c] : accoa + ser-L <==> acser + coa	Amino Acid Metabolism	398	CysE	EC-2.3.1.30
107	SERD_Lr	L-serine deaminase	[c] : ser-L <==> nh4 + pyr	Amino Acid Metabolism	320	IlvA	EC-4.3.1.17
108	SERTRS	Seryl-tRNA synthetase	[c] : atp + ser-L + trnaser --> amp + ppi + sertrna	Amino Acid Metabolism	140	SerS	EC-6.1.1.11
109	SHK3D	shikimate dehdrogenase	[c] : 3dhsk + h + nadph <==> nadp + skm	Amino Acid Metabolism	107	AroE1	EC-1.1.1.25
110	SHKK	shikimate kinase	[c] : atp + skm --> adp + h + skm5p	Amino Acid Metabolism	106	AroK	EC-2.7.1.71
111	SOD	superoxide dismutase	[c] : (2) h + (2) o2- --> h2o2 + o2	Amino Acid Metabolism	272	SodA	EC-1.15.1.1
112	THDPS	tetrahydropicolinate succinylase	[c] : h2o + succoa + thdp --> coa + sl2a6o	Amino Acid Metabolism	121	DapD	EC-2.3.1.117
113	THRD_L	L-threonine deaminase	[c] : thr-L --> 2obut + nh4	Amino Acid Metabolism	320	IlvA	
114	THRPD	threonine-phosphate decarboxylase	[c] : h + thrp --> 1ap2olp + co2	Amino Acid Metabolism	165	CobQ-CobD	
115	THRS	threonine synthase	[c] : h2o + phom --> pi + thr-L	Amino Acid Metabolism	320	ThrC	EC-4.2.3.1
116	THRTRS	Threonyl-tRNA synthetase	[c] : atp + thr-L + trnathr --> amp + ppi + thrtrna	Amino Acid Metabolism	197	ThrS	EC-6.1.1.3
117	TRPS1	tryptophan synthase (indoleglycerol phosphate)	[c] : 3ig3p + ser-L --> g3p + h2o + trp-L	Amino Acid Metabolism	379, 287, 380	TrpA+TrpB	EC-4.2.1.20
118	TRPTRS	Tryptophanyl-tRNA synthetase	[c] : atp + trnatrp + trp-L --> amp + ppi + trptrna	Amino Acid Metabolism	352	TrpS	EC-6.1.1.2
119	TYRTA	tyrosine transaminase	[c] : akg + tyr-L <==> 34hpp + glu-L	Amino Acid Metabolism	231	HisC	EC-2.6.1.5
120	TYRTRS	Tyrosyl-tRNA synthetase	[c] : atp + trnatyr + tyr-L --> amp + ppi + tyrtrna	Amino Acid Metabolism	147	TyrS	EC-6.1.1.1
121	VALTA	valine transaminase	[c] : akg + val-L <==> 3mob + glu-L	Amino Acid Metabolism	5	IlvE	EC-2.6.1.42

No	Abbreviation	Name	Equation	Subsystem	Model gene number	Protein	Protein Classification
122	VALTRS	Valyl-tRNA synthetase	[c] : atp + trnaval + val-L --> amp + ppi + valtrna	Amino Acid Metabolism	85	ValS	EC-6.1.1.9
123	ABTA	4-aminobutyrate transaminase	[c] : 4abut + akg --> glu-L + sucsal	Carbohydrate Metabolism	333	GabT	EC-2.6.1.19
124	ACS	acetyl-CoA synthetase	[c] : ac + atp + coa --> accoa + amp + ppi	Carbohydrate Metabolism	323	Acs	EC-6.2.1.1
125	GALU	UTP-glucose-1-phosphate uridylyltransferase	[c] : g1p + h + utp <==> ppi + udpg	Carbohydrate Metabolism	121, 122	GalF	EC-2.7.7.9
126	PPCOAC	Propionyl-CoA carboxylase	[c] : atp + hco3 + ppcoa --> adp + h + mmcoa-S + pi	Carbohydrate Metabolism	92	YngE	EC-6.4.1.3
127	TGBPA	Tagatose-bisphosphate aldolase	[c] : tagdp-D <==> dhap + g3p	Carbohydrate Metabolism	354	AgaZ	EC-4.1.2.40
128	UDPG4E	UDPglucose 4-epimerase	[c] : udpg <==> udpgal	Carbohydrate Metabolism	475, 426, 346, 476	GalE	EC-5.1.3.2
129	XYLI2i	xylose isomerase	[c] : fru --> glc-D	Carbohydrate Metabolism	299	XylA	EC-5.3.1.5
130	G1PACT	glucosamine-1-phosphate N-acetyltransferase	[c] : accoa + gam1p --> acgam1p + coa + h	Cell Envelope Biosynthesis	121, 122	GlmU	EC-2.3.1.157
131	GF6PTA	glutamine-fructose-6-phosphate transaminase	[c] : f6p + gln-L --> gam6p + glu-L	Cell Envelope Biosynthesis	123	GlmS	EC-2.6.1.16
132	GLCS1	glycogen synthase (ADPGlc)	[c] : adpglc --> adp + glycogen + h	Cell Envelope Biosynthesis	284, 280	GlgA1	EC-2.4.1.21
133	GLGC	glucose-1-phosphate adenylyltransferase	[c] : atp + g1p + h --> adpglc + ppi	Cell Envelope Biosynthesis	215, 322, 89, 481	GlgC	EC-2.7.7.27
134	LPADSS	Lipid A disaccaride synthase	[c] : lipidX + u23ga --> h + lipidAds + udp	Cell Envelope Biosynthesis	176	LpxB	EC-2.4.1.182
135	PGAMT	phosphoglucosamine mutase	[c] : gam1p <==> gam6p	Cell Envelope Biosynthesis	114, 120	GlmM	EC-5.4.2.10
136	UAGCVT	UDP-N-acetylglucosamine 1-carboxyvinyltransferase	[c] : pep + uacgam --> pi + uaccg	Cell Envelope Biosynthesis	312	MurAA	EC-2.5.1.7
137	UAGDP	UDP-N-acetylglucosamine diphosphorylase	[c] : acgam1p + h + utp --> ppi + uacgam	Cell Envelope Biosynthesis	121, 122	GlmU	EC-2.7.7.23
138	UAPGR	UDP-N-acetylenolpyruvoylglucosamine reductase	[c] : h + nadph + uaccg --> nadp + uamr	Cell Envelope Biosynthesis	154	MurB	EC-1.1.1.158
139	ACONT	aconitase	[c] : cit <==> icit	Citrate Cycle (TCA)	219+218+94+93	Aco3	EC-4.2.1.3
140	FUM	fumarase	[c] : fum + h2o <==> mal-L	Citrate Cycle (TCA)	98, 97	TtdA+FumB	EC-4.2.1.2
141	ICDHy	isocitrate dehydrogenase (NADP)	[c] : icit + nadp <==> akg + co2 + nadph	Citrate Cycle (TCA)	95	Icd	EC-1.1.1.42

No	Abbreviation	Name	Equation	Subsystem	Model gene number	Protein	Protein Classification
142	MDH	malate dehydrogenase	[c] : mal-L + nad <==> h + nadh + oaa	Citrate Cycle (TCA)	96	Mdh	EC-1.1.1.37
143	MICITH	2-methylisocitrate hydratase	[c] : 2mcacn + h2o --> micit	Citrate Cycle (TCA)	219+218+94+93	Aco3	EC-4.2.1.99
144	OOR2r	2-oxoglutarate synthase	[c] : akg + coa + (2) fdox <==> co2 + (2) fdred + h + succoa	Citrate Cycle (TCA)	181+182+183+184	Ofo	EC-1.2.7.3
145	POR2	pyruvate synthase	[c] : coa + (2) fdox + pyr <==> accoa + co2 + (2) fdred + h	Citrate Cycle (TCA)	181+182+183+184	Pfor	EC-1.2.7.1
146	ACBIPGT	Adenosyl cobainamide GTP transferase	[c] : adcobap + gtp + h --> adgcoba + ppi	Cofactor and Prosthetic Group Biosynthesis	169	CobU	
147	ADCL	4-aminobenzoate synthase	[c] : 4adcho --> 4abz + h + pyr	Cofactor and Prosthetic Group Biosynthesis	373	PabABC	
148	ADCOBAK	Adenosyl cobinamide kinase	[c] : adcoba + atp --> adcobap + adp + h	Cofactor and Prosthetic Group Biosynthesis	169	CobU	
149	ADCOBAS	adenosylcobinamide-phosphate synthase	[c] : 1ap2ol + adcobhex --> adcoba + h2o	Cofactor and Prosthetic Group Biosynthesis	430, 300, 164	CbiB, CbiD	
150	ADCOBHEXS	adenosylcobyric acid synthase	[c] : adcobdam + (4) atp + (4) gln-L + (4) h2o --> adcobhex + (4) adp + (4) glu-L + (4) h + (4) pi	Cofactor and Prosthetic Group Biosynthesis	260	CobQ	
151	ADCPS2	adenosylcobinamide-phosphate synthase	[c] : 1ap2olp + adcobhex + atp --> adcobap + adp + h + pi	Cofactor and Prosthetic Group Biosynthesis	430, 300, 164	CbiD, CbiB	
152	ADCS	4-amino-4-deoxychorismate synthase	[c] : chor + gln-L --> 4adcho + glu-L	Cofactor and Prosthetic Group Biosynthesis	373	PabABC	
153	AHMMPS	4-amino-5-hydroxymethyl-2-methylpyrimidine synthetase	[c] : air + h2o --> 4ahmmp + gcald + (0.5) o2 + pi	Cofactor and Prosthetic Group Biosynthesis	201	ThiC	
154	ALATA_D2	D-alanine transaminase	[c] : ala-D + pydx5p --> pyam5p + pyr	Cofactor and Prosthetic Group Biosynthesis	87	GlyA	
155	ALATA_L2	alanine transaminase	[c] : ala-L + pydx5p --> pyam5p + pyr	Cofactor and Prosthetic Group Biosynthesis	87	GlyA	
156	AMAOT	adenosylmethionine-8-amino-7-oxononanoate transaminase	[c] : 8aonn + amet <==> amob + dann	Cofactor and Prosthetic Group Biosynthesis	333	BioA	EC-2.6.1.62

No	Abbreviation	Name	Equation	Subsystem	Model gene number	Protein	Protein Classification
157	AMPMS	4-amino-2-methyl-5-phosphomethylpyrimidine synthetase	[c] : air + h2o --> 4ampm + (2) for + (4) h	Cofactor and Prosthetic Group Biosynthesis	201	ThiC	
158	APRAUR	5-amino-6-(5-phosphoribosylamino)uracil reductase	[c] : 5apru + h + nadph --> 5aprbu + nadp	Cofactor and Prosthetic Group Biosynthesis	310	RibD	EC-1.1.1.193
159	ASP1DC	aspartate 1-decarboxylase	[c] : asp-L + h --> ala-B + co2	Cofactor and Prosthetic Group Biosynthesis	210	PanD	EC-4.1.1.11
160	BACCL	biotin-[acetyl-CoA-carboxylase] ligase	[c] : atp + btn + h --> btamp + ppi	Cofactor and Prosthetic Group Biosynthesis	235	BirA	EC-6.3.4.15
161	BTS	Biotin synthase	[c] : dtbt + (2) s --> btn + h + h2s	Cofactor and Prosthetic Group Biosynthesis	306	BioBec	EC-2.8.1.6
162	CBIAT	Cobinamide adenyltransferase	[c] : atp + cbi + h2o <==> adcoba + pi + ppi	Cofactor and Prosthetic Group Biosynthesis	326, 301, 429	CobA	EC-2.5.1.17
163	CBLATr	cob(I)alamin adenosyltransferase	[c] : atp + cbl1 + h <==> adocbl + pppi	Cofactor and Prosthetic Group Biosynthesis	326, 301, 429	CobA	EC-2.5.1.17
164	CDPMEK	4-(cytidine 5'-diphospho)-2-C methyl-D-erythritol kinase	[c] : 4c2me + atp --> 2p4c2me + adp + h	Cofactor and Prosthetic Group Biosynthesis	74	IspE	
165	COBPS	cobalamin-5'-phosphate synthase	[c] : adgcoba + rdmbzi --> cobamcoa + gmp + h	Cofactor and Prosthetic Group Biosynthesis	167	CobS	
166	COBPS2	cobalamin-5'-phosphate synthase	[c] : adgcoba + rdmbzi <==> adocbl + gmp + h	Cofactor and Prosthetic Group Biosynthesis	167	CobS	
167	CPC3MT	precorrin-3B C17-methyltransferase	[c] : amet + copre3 + h --> ahcys + copre4	Cofactor and Prosthetic Group Biosynthesis	260	CobQ	EC-2.1.1.131
168	CPPPGOAN2	Oxygen Independent coproporphyrinogen-III oxidase	[c] : (2) amet + cpppg3 + (4) fdox + (2) nadph --> (2) co2 + (2) dad-5 + (4) fdred + (2) h + (2) met-L + (2) nadp + pppg9	Cofactor and Prosthetic Group Biosynthesis	236	HemN	
169	CYRDAAT	cob(I)yrinic acid a,c-diamide adenosyltransferase	[c] : atp + co1dam + h --> adcobdam + pppi	Cofactor and Prosthetic Group Biosynthesis	326, 301, 429	CobA	EC-2.5.1.17

No	Abbreviation	Name	Equation	Subsystem	Model gene number	Protein	Protein Classification
170	CYRDAS	Cob(II)yrinate a,c-diamide synthase (glutamine-hydrolysing)	[c] : (4) atp + (2) cobrnt + (4) gln-L + (2) h2o --> (4) adp + (2) co2dam + (4) glu-L + (2) h + (2) ppi	Cofactor and Prosthetic Group Biosynthesis	29	CobB	
171	DB4PS	3,4-Dihydroxy-2-butanone-4-phosphate	[c] : ru5p-D --> db4p + for + h	Cofactor and Prosthetic Group Biosynthesis	308	RibA	
172	DHFOR2	dihydrofolate reductase	[c] : dhf + nadp <==> fol + nadph	Cofactor and Prosthetic Group Biosynthesis	214	DfrA	EC-1.5.1.3
173	DHFOR3	dihydrofolate reductase	[c] : fol + h + (2) nadph <==> (2) nadp + thf	Cofactor and Prosthetic Group Biosynthesis	214	DfrA	EC-1.5.1.3
174	DHFR	dihydrofolate reductase	[c] : dhf + h + nadph <==> nadp + thf	Cofactor and Prosthetic Group Biosynthesis	214	DfrA	EC-1.5.1.3
175	DHFS	dihydrofolate synthase	[c] : atp + dhpt + glu-L --> adp + dhf + h + pi	Cofactor and Prosthetic Group Biosynthesis	7	FolC	EC-6.3.2.12
176	DHNAOT	1,4-dihydroxy-2-naphthoate octaprenyltransferase	[c] : dhna + nad + octdp --> 2dmmq8 + co2 + nadh + ppi	Cofactor and Prosthetic Group Biosynthesis	73	MenA	
177	DHNPA	dihydroneopterin aldolase	[c] : dhnpt --> 2ahhmp + gcald	Cofactor and Prosthetic Group Biosynthesis	401, 402	FolB	EC-4.1.2.25
178	DHPPDA2	diaminohydroxyphosphoribosylaminopyrimidine deaminase	[c] : 25dhpp + h + h2o --> 5apru + nh4	Cofactor and Prosthetic Group Biosynthesis	310	RibD	EC-3.5.4.26
179	DHPS1	dihydropteroate synthase	[c] : 2ahhmp + 4abz --> dhpt + h2o	Cofactor and Prosthetic Group Biosynthesis	401	FolP	EC-2.5.1.15
180	DHPS3	dihydropteroate synthase	[c] : 2ahhmd + 4abz --> dhpt + ppi	Cofactor and Prosthetic Group Biosynthesis	401	FolP	EC-2.5.1.15
181	DMPPS	1-hydroxy-2-methyl-2-(E)-butenyl 4-diphosphate reductase (dmpp)	[c] : h + h2mb4p + nadh --> dmpp + h2o + nad	Cofactor and Prosthetic Group Biosynthesis	353	LytB	
182	DMQMT	3-Dimethylubiquinonol 3-methyltransferase	[c] : 2omhmbl + amet --> ahcys + h + ubq8h2	Cofactor and Prosthetic Group Biosynthesis	371	UbiG	

No	Abbreviation	Name	Equation	Subsystem	Model gene number	Protein	Protein Classification
183	DNMPPA	Dihydroneopterin monophosphate dephosphorylase	[c] : dhpmp + h2o --> dhnpt + pi	Cofactor and Prosthetic Group Biosynthesis	125, 336, 312	NtpA	
184	DNTPPA	Dihydroneopterin triphosphate pyrophosphatase	[c] : ahdt + h2o --> dhpmp + h + ppi	Cofactor and Prosthetic Group Biosynthesis	125, 336, 312	NtpA	
185	DPCOAK	dephospho-CoA kinase	[c] : atp + dpcoa --> adp + coa + h	Cofactor and Prosthetic Group Biosynthesis	345	CoaE	EC-2.7.1.24
186	DPR	2-dehydropantoate 2-reductase	[c] : 2dhp + h + nadph --> nadp + pant-R	Cofactor and Prosthetic Group Biosynthesis	221, 102	YlbQ	EC-1.1.1.169
187	DXPRI	1-deoxy-D-xylulose-5-phosphate reductoisomerase	[c] : dxyl5p + h + nadph <==> 2me4p + nadp	Cofactor and Prosthetic Group Biosynthesis	58	Dxr	
188	DXPS	1-deoxy-D-xylulose 5-phosphate synthase	[c] : g3p + h + pyr --> co2 + dxyl5p	Cofactor and Prosthetic Group Biosynthesis	195	Dxs	
189	FMNAT	FMN adenylyltransferase	[c] : atp + fmn + h --> fad + ppi	Cofactor and Prosthetic Group Biosynthesis	148	RibF	EC-2.7.7.2
190	GTPCI	GTP cyclohydrolase I	[c] : gtp + h2o --> ahdt + for + h	Cofactor and Prosthetic Group Biosynthesis	319	FolE	EC-3.5.4.16
191	GTPCII	GTP cyclohydrolase II	[c] : gtp + (3) h2o --> 25dhpp + for + (2) h + ppi	Cofactor and Prosthetic Group Biosynthesis	308	RibA	EC-3.5.4.25
192	HMPK1	hydroxymethylpyrimidine kinase (ATP)	[c] : 4ahmmp + atp --> 4ampm + adp + h	Cofactor and Prosthetic Group Biosynthesis	203	ThiE	EC-2.7.1.49
193	HMPK2	hydroxymethylpyrimidine kinase (CTP)	[c] : 4ahmmp + ctp --> 4ampm + cdp + h	Cofactor and Prosthetic Group Biosynthesis	203	ThiE	EC-2.7.1.49
194	HMPK3	hydroxymethylpyrimidine kinase (UTP)	[c] : 4ahmmp + utp --> 4ampm + h + udp	Cofactor and Prosthetic Group Biosynthesis	203	ThiE	EC-2.7.1.49
195	HMPK4	hydroxymethylpyrimidine kinase (GTP)	[c] : 4ahmmp + gtp --> 4ampm + gdp + h	Cofactor and Prosthetic Group Biosynthesis	203	ThiE	EC-2.7.1.49

No	Abbreviation	Name	Equation	Subsystem	Model gene number	Protein	Protein Classification
196	HPPK	2-amino-4-hydroxy-6-hydroxymethyldihydropteridine diphosphokinase	[c] : 2ahhmp + atp --> 2ahhmd + amp + h	Cofactor and Prosthetic Group Biosynthesis	402	FolK	EC-2.7.6.3
197	IPDPS	1-hydroxy-2-methyl-2-(E)-butenyl 4-diphosphate reductase (ipdp)	[c] : h + h2mb4p + nadh --> h2o + ipdp + nad	Cofactor and Prosthetic Group Biosynthesis	353	LytB	
198	MAN1PT1	mannose-1-phosphate guanylyltransferase	[c] : gtp + h + man1p --> gdpman + ppi	Cofactor and Prosthetic Group Biosynthesis	322, 481	Mpg	EC-2.7.7.13
199	MECDPDH	2C-methyl-D-erythritol 2,4 cyclodiphosphate dehydratase	[c] : 2mecdp + nadh --> h2mb4p + h2o + nad	Cofactor and Prosthetic Group Biosynthesis	57	IspG	
200	MECDPS	2-C-methyl-D-erythritol 2,4-cyclodiphosphate synthase	[c] : 2p4c2me --> 2mecdp + cmp	Cofactor and Prosthetic Group Biosynthesis	17	IspF	
201	MEPCT	2-C-methyl-D-erythritol 4-phosphate cytidylyltransferase	[c] : 2me4p + ctp + h --> 4c2me + ppi	Cofactor and Prosthetic Group Biosynthesis	16	IspD	
202	MOHMT	3-methyl-2-oxobutanoate hydroxymethyltransferase	[c] : 3mob + h2o + mlthf --> 2dhp + thf	Cofactor and Prosthetic Group Biosynthesis	211	PanB	EC-2.1.2.11
203	MTHFC	methenyltetrahydrofolate cyclohydrolase	[c] : h2o + methf <==> 10fthf + h	Cofactor and Prosthetic Group Biosynthesis	173, 175	FolD , Fhs	EC-3.5.4.9
204	MTHFD	methylenetetrahydrofolate dehydrogenase (NADP)	[c] : mlthf + nadp <==> methf + nadph	Cofactor and Prosthetic Group Biosynthesis	173, 175	FolD , Fhs	EC-1.5.1.5
205	NADK	NAD kinase	[c] : atp + nad --> adp + h + nadp	Cofactor and Prosthetic Group Biosynthesis	101	NadK	EC-2.7.1.23
206	NADS1	NAD synthase (nh4)	[c] : atp + dnad + nh4 --> amp + h + nad + ppi	Cofactor and Prosthetic Group Biosynthesis	302	NadE	EC-6.3.1.5
207	NADS2	NAD(+) synthase (glutamine-hydrolyzing)	[c] : atp + dnad + gln-L + h2o --> amp + glu-L + h + nad + ppi	Cofactor and Prosthetic Group Biosynthesis	289	NadE2	EC-6.3.5.1
208	NMNATr	nicotinamide-nucleotide adenylyltransferase	[c] : atp + h + nmn <==> nad + ppi	Cofactor and Prosthetic Group Biosynthesis	1	NadD	EC-2.7.7.1

No	Abbreviation	Name	Equation	Subsystem	Model gene number	Protein	Protein Classification
209	NNATr	nicotinate-nucleotide adenylyltransferase	[c] : atp + h + nicrnt <==> dnad + ppi	Cofactor and Prosthetic Group Biosynthesis	1	NadD	EC-2.7.7.18
210	NNDMBRT	nicotinate-nucleotide-dimethylbenzimidazole phosphoribosyltransferase	[c] : dmbzid + nicrnt --> 5prdmbz + h + nac	Cofactor and Prosthetic Group Biosynthesis	166	CobT	EC-2.4.2.21
211	NNDMBRT3	nicotinate-nucleotide-dimethylbenzimidazole phosphoribosyltransferase	[c] : ribflv --> dmbzid	Cofactor and Prosthetic Group Biosynthesis	166	CobT	EC-Undetermined
212	NNDPR	nicotinate-nucleotide diphosphorylase (carboxylating)	[c] : (2) h + prpp + quln --> co2 + nicrnt + ppi	Cofactor and Prosthetic Group Biosynthesis	360	NadC1	EC-2.4.2.19
213	OHPHM	R04988	[c] : 2ohph + amet --> 2omph + ahcys + h	Cofactor and Prosthetic Group Biosynthesis	371	UbiG	
214	OMBZLM	2-Octaprenyl-6-methoxy-benzoquinol methylase	[c] : 2ombzl + amet --> 2ommbl + ahcys + h	Cofactor and Prosthetic Group Biosynthesis	12, 71, 400, 70	UbiE	
215	PACCOAL	phenylacetate-CoA ligase	[c] : atp + coa + pheac --> amp + phaccoa + ppi	Cofactor and Prosthetic Group Biosynthesis	285, 262	PaaK	EC-6.2.1.30
216	PANTS	pantothenate synthase	[c] : ala-B + atp + pant-R --> amp + h + pnto-R + ppi	Cofactor and Prosthetic Group Biosynthesis	212	PanC	EC-6.3.2.1
217	PDX5PS	Pyridoxine 5'-phosphate synthase	[c] : dxyl5p + nad + phthr --> co2 + h + (2) h2o + nadh + pdx5p + pi	Cofactor and Prosthetic Group Biosynthesis	64	PdxAJ	
218	PMDPHT	pyrimidine phosphatase	[c] : 5aprbu + h2o --> 4r5au + pi	Cofactor and Prosthetic Group Biosynthesis	361	Pmdpht	
219	PNTEH	Hydrolase Class (RXN R02973)	[c] : h2o + ptth --> cysam + pnto-R	Cofactor and Prosthetic Group Biosynthesis	127	Phyd	
220	PNTK	pantothenate kinase	[c] : atp + pnto-R --> 4ppan + adp + h	Cofactor and Prosthetic Group Biosynthesis	83	CoaA	EC-2.7.1.33

No	Abbreviation	Name	Equation	Subsystem	Model gene number	Protein	Protein Classification
221	PPCDC	phosphopantothenoylcysteine decarboxylase	[c] : 4ppcys + h --> co2 + pan4p	Cofactor and Prosthetic Group Biosynthesis	84	Dfp	EC-4.1.1.36
222	PPNCL	phosphopantothenate-cysteine ligase	[c] : 4ppan + ctp + cys-L --> 4ppcys + cdp + h + pi	Cofactor and Prosthetic Group Biosynthesis	84	Dfp	EC-6.3.2.5
223	PTHPS	6-pyruvoyltetrahydropterin synthase	[c] : ahdt --> 6pthp + pppi	Cofactor and Prosthetic Group Biosynthesis	403	Ptps	EC-4.2.3.12
224	PTPAT	pantetheine-phosphate adenylyltransferase	[c] : atp + h + pan4p <==> dpcoa + ppi	Cofactor and Prosthetic Group Biosynthesis	43	CoaD	EC-2.7.7.3
225	PYAM5PO	pyridoxamine-phosphate oxidase	[c] : h2o + o2 + pyam5p --> h2o2 + nh4 + pydx5p	Cofactor and Prosthetic Group Biosynthesis	418	PdxHec	EC-1.4.3.5
226	QULNS	quinolinate synthase	[c] : dhap + iasp --> (2) h2o + pi + quln	Cofactor and Prosthetic Group Biosynthesis	399	NadA	
227	RBFK	riboflavin kinase	[c] : atp + ribflv --> adp + fmn + h	Cofactor and Prosthetic Group Biosynthesis	148	RibF	EC-2.7.1.26
228	RBFSa	riboflavin synthase	[c] : 4r5au + db4p --> dmlz + (2) h2o + pi	Cofactor and Prosthetic Group Biosynthesis	307+309	RibEH	EC-2.5.1.9
229	RBFSb	riboflavin synthase	[c] : (2) dmlz --> 4r5au + ribflv	Cofactor and Prosthetic Group Biosynthesis	307+309	RibEH	EC-2.5.1.9
230	TMPKr	thiamine-phosphate kinase	[c] : atp + thmmp <==> adp + thmpp	Cofactor and Prosthetic Group Biosynthesis	69	ThiL	EC-2.7.4.16
231	TMPPP	thiamine-phosphate diphosphorylase	[c] : 2mahmp + 4mpetz + h --> ppi + thmmp	Cofactor and Prosthetic Group Biosynthesis	203	ThiE	EC-2.5.1.3
232	UDPDPS	undecaprenyl-diphosphate synthase	[c] : decdp + ipdp --> ppi + udcpdp	Cofactor and Prosthetic Group Biosynthesis	60	UppS	EC-2.5.1.31
233	ADCOBHS	Adenosylcobinamide hydrolase	[c] : 1ap2ol + adcobhex <==> adcoba + h2o	Cofactor and Prosthetic Group Biosynthesis	413	CbiZ	EC-3.5.1

No	Abbreviation	Name	Equation	Subsystem	Model gene number	Protein	Protein Classification
234	MAN1PT2	mannose-1-phosphate guanylyltransferase (GDP)	[c] : gdp + h + man1p --> gdpman + pi	Cofactor and Prosthetic Group Biosynthesis	75	ManC	EC-2.7.7.22
235	RBZP	alpha-ribazole-5-phosphatase	[c] : 5prdmbz + h2o --> pi + rdmbzi	Cofactor and Prosthetic Group Biosynthesis	168	CobC	EC-Undetermined
236	UPP3MT	uroporphyrinogen methyltransferase	[c] : (2) amet + uppg3 --> (2) ahcys + (2) h + shcl	Cofactor and Prosthetic Group Biosynthesis	412	HemD	EC-2.1.1.107
237	UPP3S	uroporphyrinogen-III synthase	[c] : hmbil --> h2o + uppg3	Cofactor and Prosthetic Group Biosynthesis	412	HemD	EC-4.2.1.75
238	AHC	adenosylhomocysteinase	[c] : ahcys + h2o <==> adn + hcys-L	Energy Metabolism	116	AhcY	EC-3.3.1.1
239	ALCD2x	alcohol dehydrogenase (ethanol: NAD)	[c] : etoh + nad <==> acald + h + nadh	Energy Metabolism	15, 27	AdhC	EC-1.1.1.1
240	ALCD3	alcohol dehydrogenase (propanol: NAD)	[c] : nad + ppoh <==> h + nadh + ppal	Energy Metabolism	15, 27	AdhC	EC-1.1.1.1

No	Abbreviation	Name	Equation	Subsystem	Model gene number	Protein	Protein Classification
241	RDR1	Chlorobenzene reductive dechlorination	(8) fdred[c] + (4) h[c] + hcb[e] --> (4) cl[e] + dcb[e] + (8) fdox[c]	Energy Metabolism	519, 472, 527, 447, 523, 539, 499, 449, 506, 470, 468, 547, 452, 544, 538, 501, 540, 464, 50, 450, 520, 518, 440, 512, 522, 546, 442, 460, 444, 446, 482, 466, 508, 457, 462, 541, 542, 548, 549, 388, 41, 502, 521, 543, 545, 479, 515, 305, 510, 455, 537, 453, 51, 507, 304, 467, 514, 458, 443, 463, 535, 509, 532, 484, 486, 49, 469, 487, 505, 531, 534, 448, 511, 474, 461, 529, 427, 517, 471, 465, 40, 536, 425, 473, 478, 441, 533, 426, 445, 528, 516, 19, 439, 451, 513, 485, 530, 459, 456, 504, 503	RdhA+RdhB	

No	Abbreviation	Name	Equation	Subsystem	Model gene number	Protein	Protein Classification
242	RDR2	Chloroethene reductive dechlorination	(8) fdred[c] + (4) h[c] + pce[e] --> (4) cl[e] + etl[e] + (8) fdox[c]	Energy Metabolism	519, 472, 527, 447, 523, 539, 499, 449, 506, 470, 468, 547, 452, 544, 538, 501, 540, 464, 50, 450, 520, 518, 440, 512, 522, 546, 442, 460, 444, 446, 482, 466, 508, 457, 462, 541, 542, 548, 549, 388, 41, 502, 521, 543, 545, 479, 515, 305, 510, 455, 537, 453, 51, 507, 304, 467, 514, 458, 443, 463, 535, 509, 532, 484, 486, 49, 469, 487, 505, 531, 534, 448, 511, 474, 461, 529, 427, 517, 471, 465, 40, 536, 425, 473, 478, 441, 533, 426, 445, 528, 516, 19, 439, 451, 513, 485, 530, 459, 456, 504, 503	RdhA+RdhB	
243	CMLDC	4-carboxymuconolactone decarboxylase	[c] : 2c25dho + h --> 5odhf2a + co2	Energy Metabolism	39	PcaC2	EC-4.1.1.44
244	CODHr	carbon-monoxide dehydrogenase	[c] : co + (2) fdox + h2o <==> co2 + (2) fdred + (2) h	Energy Metabolism	171, 172, 170, 174	CooS	EC-1.2.99.2
245	DGK1	deoxyguanylate kinase (dGMP:ATP)	[c] : atp + dgmp <==> adp + dgdp	Energy Metabolism	9	Gmk	
246	F1PK	fructose-1-phosphate kinase	[c] : atp + f1p --> adp + fdp + h	Energy Metabolism	355	FruK	EC-2.7.1.56
247	FNOR2	ferredoxin-NADP reductase	[c] : (2) fdred + h + nadp <==> (2) fdox + nadph	Energy Metabolism	11	P_1.18.1.2	EC-1.18.1.2
248	FRHD	Ferredoxin hydrogenase	(2) fdox[c] + h2[e] <==> (2) fdred[c] + (2) h[e]	Energy Metabolism	23+20+22+21+483+150+240+246+245+238+239+44+47+488+243+187+37	Fdx, FrdH	EC-1.12.7,

No	Abbreviation	Name	Equation	Subsystem	Model gene number	Protein	Protein Classification
249	G3PD1	glycerol-3-phosphate dehydrogenase (NAD)	[c] : glyc3p + nad <==> dhap + h + nadh	Energy Metabolism	363	GpsA	EC-1.1.1.94
250	G3PD2	glycerol-3-phosphate dehydrogenase (NADP)	[c] : glyc3p + nadp <==> dhap + h + nadph	Energy Metabolism	363	GpsA	EC-1.1.1.94
251	GK1	guanylate kinase (GMP:ATP)	[c] : atp + gmp <==> adp + gdp	Energy Metabolism	9	Gmk	EC-2.7.4.8
252	GLUSx	glutamate synthase (NADH2)	[c] : akg + gln-L + h + nadh --> (2) glu-L + nad	Energy Metabolism	298, 296	GltAB	EC-1.4.1.14
253	GLYOX	hydroxyacylglutathione hydrolase	[c] : h2o + lgt-S --> gthrd + h + lac-D	Energy Metabolism	88, 205	GloB	EC-3.1.2.6
254	HGR	mercury(II) reductase	[c] : hg2 + nadph <==> h + hg0 + nadp	Energy Metabolism	188	MerA	EC-1.16.1.1
255	IOR	indolepyruvate ferredoxin oxidoreductase	[c] : coa + (2) fdox + indpyr <==> co2 + (2) fdred + h + indaccoa	Energy Metabolism	269+270	IorAB1	EC-Undetermined
256	IOR2	indolepyruvate ferredoxin oxidoreductase (phenylpyvurate)	[c] : coa + (2) fdox + phpyr <==> co2 + (2) fdred + h + phaccoa	Energy Metabolism	269+270	IorAB1	EC-Undetermined
257	IOR3	indolepyruvate ferredoxin oxidoreductase (hydroxyphenylpyvurate)	[c] : 34hpp + coa + (2) fdox <==> 4hphaccoa + co2 + (2) fdred + h	Energy Metabolism	269+270	IorAB1	
258	MAN6PI	mannose-6-phosphate isomerase	[c] : man6p <==> f6p	Energy Metabolism	113	ManA	EC-5.3.1.8
259	MGSA	methylglyoxal synthase	[c] : dhap --> mthgxl + pi	Energy Metabolism	397, 30	MgsA	EC-4.2.3.3
260	NADH4	NADH dehydrogenase (Menaquinone 7 & no proton)	[c] : h + mqn7 + nadh --> mql7 + nad	Energy Metabolism	149+249+259+254+255+256+251+252+258+257+253+250+242+241+243+393+394+396+391+392+395+91+186+185+187+37+35+36+38	Nadhd5	EC-1.6.5.3
261	NADH6	NADH dehydrogenase (ubiquinone-8 & 3.5 protons)	(4.5) h[c] + nadh[c] + ubq8[c] --> (3.5) h[e] + nad[c] + ubq8h2[c]	Energy Metabolism	149+249+259+254+255+256+251+252+258+257+253+250+242+241+243+393+394+396+391+392+395+91+186+185+187+37+35+36+38	Nadhd5	EC-1.6.5.3,

No	Abbreviation	Name	Equation	Subsystem	Model gene number	Protein	Protein Classification
262	NADH7	NADH dehydrogenase (menaquinone-8 & 2 protons)	(3) h[c] + mqn8[c] + nadh[c] --> (2) h[e] + mql8[c] + nad[c]	Energy Metabolism	149+249+259+254+255+256+251+252+258+257+253+250+242+241+243+393+394+396+391+392+395+91+186+185+187+37+35+36+38	Nadhd5	EC-1.6.5.3,
263	NADH8	NADH dehydrogenase (demethylmenaquinone-8 & 2.8 protons)	2dmmq8[c] + (3.8) h[c] + nadh[c] --> 2dmmql8[c] + (2.8) h[e] + nad[c]	Energy Metabolism	149+249+259+254+255+256+251+252+258+257+253+250+242+241+243+393+394+396+391+392+395+91+186+185+187+37+35+36+38	Nadhd5	EC-1.6.5.3,
264	NADPH12	Quinone oxidoreductase	[c] : h + nadph + ubq8 --> nadp + ubq8h2	Energy Metabolism	37+243+187	Nadphd	EC-1.6.5.5
265	NTRIRx	nitrite reductase (NADH)	[c] : (4) h + (3) nadh + no2 --> h2o + (3) nad + nh4oh	Energy Metabolism	296	NasDE	EC-1.7.1.4
266	NTRIRy	nitrite reductase (NADPH)	[c] : (4) h + (3) nadph + no2 --> h2o + (3) nadp + nh4oh	Energy Metabolism	296	NasDE	EC-1.7.1.4
267	OXAD2	Oxaloacetate decarboxylase	[c] : h + oaa --> co2 + pyr	Energy Metabolism	24	OadD	EC-4.1.1.3
268	PC	pyruvate carboxylase	[c] : atp + hco3 + pyr --> adp + h + oaa + pi	Energy Metabolism	24+25	Pyc	EC-6.4.1.1
269	PFK	phosphofructokinase	[c] : atp + f6p --> adp + fdp + h	Energy Metabolism	355	PfkB	EC-2.7.1.11
270	PFK_2	Phosphofructokinase	[c] : atp + tag6p-D --> adp + h + tagdp-D	Energy Metabolism	355	PfkB	EC-2.7.1.11
271	PGMT	phosphoglucomutase	[c] : g1p <==> g6p	Energy Metabolism	120, 114	Pmgm1	EC-5.4.2.2
272	PMANM	phosphomannomutase	[c] : man1p <==> man6p	Energy Metabolism	120, 114	Pmgm1	EC-5.4.2.8
273	PPA	inorganic diphosphatase	[c] : h2o + ppi --> h + (2) pi	Energy Metabolism	55	Ppa	EC-3.6.1.1
274	RBK	ribokinase	[c] : atp + rib-D --> adp + h + r5p	Energy Metabolism	178	RbsK	EC-2.7.1.15
275	TRDR	thioredoxin reductase (NADPH)	[c] : h + nadph + trdox --> nadp + trdrd	Energy Metabolism	126	TrxB	EC-1.8.1.9
276	ENO	enolase	[c] : 2pg <==> h2o + pep	Glycolysis/Gluconeogenesis	144	Eno	EC-4.2.1.11
277	FBA	fructose-bisphosphate aldolase	[c] : fdp <==> dhap + g3p	Glycolysis/Gluconeogenesis	354	Fba	EC-4.1.2.13
278	FBP	fructose-bisphosphatase	[c] : fdp + h2o --> f6p + pi	Glycolysis/Gluconeogenesis	327	Fbp	EC-3.1.3.11

No	Abbreviation	Name	Equation	Subsystem	Model gene number	Protein	Protein Classification
279	GAPD	glyceraldehyde-3-phosphate dehydrogenase (NAD)	[c] : g3p + nad + pi <==> 13dpg + h + nadh	Glycolysis/Gluconeogenesis	143	Gap	EC-1.2.1.12
280	HEX1	hexokinase (D-glucose:ATP)	[c] : atp + glc-D --> adp + g6p + h	Glycolysis/Gluconeogenesis	13	GlcK	EC-2.7.1.2
281	LDH_L	L-lactate dehydrogenase	[c] : lac-L + nad <==> h + nadh + pyr	Glycolysis/Gluconeogenesis	96	Ldh	EC-1.1.1.27
282	PDH	pyruvate dehydrogenase	[c] : coa + nad + pyr --> accoa + co2 + nadh	Glycolysis/Gluconeogenesis	183	PdhAB	EC-1.2.1.51
283	PGI	glucose-6-phosphate isomerase	[c] : g6p <==> f6p	Glycolysis/Gluconeogenesis	113	Pgi	EC-5.3.1.9
284	PGK	phosphoglycerate kinase	[c] : 13dpg + adp <==> 3pg + atp	Glycolysis/Gluconeogenesis	194	Pgk	EC-2.7.2.3
285	PGM	phosphoglycerate mutase	[c] : 3pg <==> 2pg	Glycolysis/Gluconeogenesis	372, 408, 193	PgmI, GpmA	EC-5.4.2.1
286	PPDK	pyruvate phosphate dikinase	[c] : atp + pi + pyr --> amp + h + pep + ppi	Glycolysis/Gluconeogenesis	129	PpdK	EC-2.7.9.1
287	PPS	phosphoenolpyruvate synthase	[c] : atp + h2o + pyr --> amp + (2) h + pep + pi	Glycolysis/Gluconeogenesis	129	Ppsa	EC-2.7.9.2
288	PYK	pyruvate kinase	[c] : adp + h + pep --> atp + pyr	Glycolysis/Gluconeogenesis	129	Pyk	EC-2.7.1.40
289	TPI	triose-phosphate isomerase	[c] : dhap <==> g3p	Glycolysis/Gluconeogenesis	192	TpiA	EC-5.3.1.1
290	AACPAT	Acetyl-CoA:[acyl-carrier-protein] S-acetyltransferase	[c] : ACP + accoa --> acACP + coa	Lipid Metabolism	274	FabF	EC-2.3.1.38
291	ACCOAC	acetyl-CoA carboxylase	[c] : accoa + atp + hco3 --> adp + h + malcoa + pi	Lipid Metabolism	24+25	AccC	EC-6.4.1.2
292	ACMAT1	Acyl-[acyl-carrier-protein]:malonyl-[acyl-carrier-protein] C-acyltransferase (decarboxylating)	[c] : acACP + h + malACP --> ACP + actACP + co2	Lipid Metabolism	274	FabF	EC-2.3.1.41
293	ACPS1	acyl-carrier protein synthase	[c] : apoACP + coa --> ACP + h + pap	Lipid Metabolism	90	AcpS	EC-2.7.8.7
294	ALCD19	alcohol dehydrogenase (glycerol)	[c] : glyald + h + nadh <==> glyc + nad	Lipid Metabolism	15, 27	AdhC	EC-1.1.1.1
295	ALDD8b	aldehyde dehydrogenase (glyceraldehyde, NAD)	[c] : glyald + h2o + nad <==> glyc-R + (2) h + nadh	Lipid Metabolism	342	AldH	EC-1.2.1.3

No	Abbreviation	Name	Equation	Subsystem	Model gene number	Protein	Protein Classification
296	BTMAT1	Butyryl-[acyl-carrier protein]:malonyl-CoA C-acyltransferase	[c] : 2beACP + h + nadh --> butACP + nad	Lipid Metabolism	338	FabI	EC-1.3.1.10
297	CDGPT	CDPdiacylglycerol:sn-glycerol-3-phosphate 3-phosphatidyltransferase	[c] : cdpdag + glyc3p --> cmp + h + pglyp	Lipid Metabolism	279	PgsA	
298	CDPDSP	CDPdiacylglycerol-serine O-phosphatidyltransferase	[c] : cdpdag + ser-L --> cmp + h + ps	Lipid Metabolism	279	PssA1	EC-2.7.8.8
299	CLPNS	cardiolipin synthetase	[c] : cdpdag + pgly --> cdlp + cmp + h	Lipid Metabolism	279	Cls	
300	DAGK	diacylglycerol kinase	[c] : 12dgr + atp --> 12dag3p + adp + h	Lipid Metabolism	77	DgkA	EC-2.7.1.107
301	DDMAT5	Dodecanoyl-[acyl-carrier protein]: malonyl-CoA C-acyltransferase	[c] : 2tddACP + h + nadh --> ddcaACP + nad	Lipid Metabolism	338	FabI	EC-1.3.1.10
302	DEMAT4	Decanoyl-[acyl-carrier protein]:malonyl-CoA C-acyltransferase	[c] : 2tdeACP + h + nadh --> decACP + nad	Lipid Metabolism	338	FabI	EC-1.3.1.10
303	DMATT	dimethylallyltranstransferase	[c] : dmpp + ipdp --> grdp + ppi	Lipid Metabolism	67	IspA	EC-2.5.1.1
304	FA120ACPH	fatty-acyl-ACP hydrolase	[c] : ddcaACP + h2o <==> ACP + ddca + h	Lipid Metabolism	339	FatB	EC-3.1.2.14
305	FA140ACPH	fatty-acyl-ACP hydrolase	[c] : h2o + myrsACP <==> ACP + h + ttdca	Lipid Metabolism	339	FatB	EC-3.1.2.14
306	FA160ACPH	fatty-acyl-ACP hydrolase	[c] : h2o + palmACP <==> ACP + h + hdca	Lipid Metabolism	339	FatB	EC-3.1.2.14
307	FA180ACPH	fatty-acyl-ACP hydrolase	[c] : h2o + ocdACP <==> ACP + h + ocdca	Lipid Metabolism	339	FatB	EC-3.1.2.14
308	FA200ACPH	fatty-acyl-ACP hydrolase	[c] : h2o + icsACP <==> ACP + h + icsa	Lipid Metabolism	339	FatB	EC-3.1.2.14
309	FACOAL120	fatty-acid--CoA ligase (dodecanoate)	[c] : atp + coa + ddca <==> amp + ddcoa + ppi	Lipid Metabolism	268, 286	FadD	EC-6.2.1.3
310	FACOAL140	fatty-acid--CoA ligase (tetradecanoate)	[c] : atp + coa + ttdca <==> amp + ppi + tdcoa	Lipid Metabolism	268, 286	FadD	EC-6.2.1.3
311	FACOAL160	fatty-acid--CoA ligase (hexadecanoate)	[c] : atp + coa + hdca <==> amp + pmtcoa + ppi	Lipid Metabolism	268, 286	FadD	EC-6.2.1.3
312	FACOAL170(ISO)	fatty-acid--CoA ligase (Iso-C17:0)	[c] : atp + coa + fa11 <==> amp + fa11coa + ppi	Lipid Metabolism	268, 286	FadD	EC-6.2.1.3
313	FACOAL180	fatty-acid--CoA ligase (octadecanoate)	[c] : atp + coa + ocdca <==> amp + ppi + strcoa	Lipid Metabolism	268, 286	FadD	EC-6.2.1.3
314	FACOAL181	fatty-acid--CoA ligase (octadecenoate)	[c] : atp + coa + ocdcea <==> amp + odecoa + ppi	Lipid Metabolism	268, 286	FadD	EC-6.2.1.3

No	Abbreviation	Name	Equation	Subsystem	Model gene number	Protein	Protein Classification
315	FACOAL200	fatty-acid--CoA ligase (icosanoate)	[c] : atp + coa + icsa <==> amp + icscoa + ppi	Lipid Metabolism	268, 286	FadD	EC-6.2.1.3
316	FCLPA	L-fuculose 1-phosphate aldolase	[c] : fc1p <==> dhap + lald-L	Lipid Metabolism	410	FucA	EC-4.1.2.17
317	FRTT	farnesyltranstransferase	[c] : frdp + ipdp --> ggdp + ppi	Lipid Metabolism	67	HepT	EC-2.5.1.29
318	GGTT	geranylgeranyltranstransferase	[c] : ggdp + ipdp --> pendp + ppi	Lipid Metabolism	67	HepT	
319	GRTT	geranyltranstransferase	[c] : grdp + ipdp --> frdp + ppi	Lipid Metabolism	67	IspA	EC-2.5.1.10
320	HBUHL1	(3R)-3-Hydroxybutanoyl-[acyl carrier-protein] hydro-lyase	[c] : 3hbACP --> 2beACP + h2o	Lipid Metabolism	339	FabZ	EC-4.2.1.58
321	HBUR1	(3R)-3-Hydroxybutanoyl-[acyl carrier protein]:NADP+ oxidoreductase	[c] : actACP + h + nadph --> 3hbACP + nadp	Lipid Metabolism	340, 189	FabG1	EC-1.1.1.100
322	HDDHL5	(3R)-3-Hydroxybutanoyl-[acyl carrier-protein] hydro-lyase	[c] : 3hddACP --> 2tddACP + h2o	Lipid Metabolism	339	FabZ	EC-4.2.1.58
323	HDDR5	3R)-3-Hydroxydodecanoyl-[acyl-carrier-protein]:NADP+ oxidoreductase	[c] : 3oxddACP + h + nadph --> 3hddACP + nadp	Lipid Metabolism	340, 189	FabG1	EC-1.1.1.100
324	HDEHL4	(3R)-3-Hydroxybutanoyl-[acyl carrier-protein] hydro-lyase	[c] : 3hdeACP --> 2tdeACP + h2o	Lipid Metabolism	339	FabZ	EC-4.2.1.58
325	HDER4	(3R)-3-Hydroxydecanoyl-[acyl-carrier-protein]:NADP+ oxidoreductase	[c] : 3oxdeACP + h + nadph --> 3hdeACP + nadp	Lipid Metabolism	340, 189	FabG1	EC-1.1.1.100
326	HDMAT7	Hexadecanoyl-[acyl-carrier protein]:malonyl-CoA C-acyltransferase	[c] : nad + palmACP <==> h + hdeACP + nadh	Lipid Metabolism	338	FabI	EC-1.3.1.10
327	HEMAT2	Hexanoyl-[acyl-carrier protein]:malonyl-CoA C-acyltransferase	[c] : 2theACP + h + nadh --> hexACP + nad	Lipid Metabolism	338	FabI	EC-1.3.1.10
328	HEPTT	trans-heptaprenyltranstransferase	[c] : hepdp + ipdp --> octdp + ppi	Lipid Metabolism	67	HepT	

No	Abbreviation	Name	Equation	Subsystem	Model gene number	Protein	Protein Classification
329	HEXTT	trans-hexaprenyltranstransferase	[c] : hexdp + ipdp --> hepdp + ppi	Lipid Metabolism	67	HepT	EC-2.5.1.30
330	HHDHL7	(3R)-3-Hydroxypalmitoyl-[acyl-carrier-protein] hydro-lyase	[c] : 3hpaACP --> h2o + hdeACP	Lipid Metabolism	339	FabZ	EC-4.2.1.58
331	HHDR7	(3R)-3-Hydroxypalmitoyl-[acyl-carrier-protein]:NADP+ oxidoreductase	[c] : 3oxhdACP + h + nadph --> 3hpaACP + nadp	Lipid Metabolism	340, 189	FabG1	EC-1.1.1.100
332	HHYHL2	(3R)-3-Hydroxybutanoyl-[acyl carrier-protein] hydro-lyase	[c] : 3hhACP --> 2theACP + h2o	Lipid Metabolism	339	FabZ	EC-4.2.1.58
333	HHYR2	(3R)-3-Hydroxyhexanoyl-[acyl-carrier-protein]:NADP+ oxidoreductase	[c] : 3oxhACP + h + nadph --> 3hhACP + nadp	Lipid Metabolism	340, 189	FabG1	EC-1.1.1.100
334	HICHL9	(3R)-3-Hydroxyicosanoyl-[acyl-carrier-protein] hydro-lyase	[c] : 3hicsacp --> 2ticsacp + h2o	Lipid Metabolism	339	FabZ	EC-4.2.1.58
335	HICR9	(3R)-3-Hydroxyicosanoyl-[acyl-carrier-protein]:NADP+ oxidoreductase	[c] : 3oxicsacp + h + nadph --> 3hicsacp + nadp	Lipid Metabolism	340, 189	FabG1	EC-1.1.1.100
336	HOCHL3	((3R)-3-Hydroxybutanoyl-[acyl-carrier-protein] hydro-lyase	[c] : 3hocACP --> 2toceACP + h2o	Lipid Metabolism	339	FabZ	EC-4.2.1.58
337	HOCR3	(3R)-3-Hydroxyoctanoyl-[acyl carrier-protein]:NADP+ oxidoreductase	[c] : 3oxocACP + h + nadph --> 3hocACP + nadp	Lipid Metabolism	340, 189	FabG1	EC-1.1.1.100
338	HODHL8n	(3R)-3-Hydroxyoctadecanoyl-[acyl-carrier-protein] hydro-lyase	[c] : 3hocdacp --> 2tocdACP + h2o	Lipid Metabolism	339	FabZ	EC-4.2.1.58
339	HODR8_#1	(3R)-3-Hydroxyoctadecanoyl-[acyl-carrier-protein]:NADP+ oxidoreductase	[c] : 3oxocdacp + h + nadph --> 3hocdacp + nadp	Lipid Metabolism	340, 189	FabG1	EC-1.1.1.100

No	Abbreviation	Name	Equation	Subsystem	Model gene number	Protein	Protein Classification
340	HTDHL6	(3R)-3-Hydroxypalmitoyl-[acyl-carrier-protein] hydro-lyase	[c] : 3htdACP --> h2o + tdeACP	Lipid Metabolism	339	FabZ	EC-4.2.1.58
341	HTDR6	3R)-3-Hydroxytetradecanoyl-[acyl-carrier-protein]:NADP+ oxidoreductase	[c] : 3oxtdACP + h + nadph --> 3htdACP + nadp	Lipid Metabolism	340, 189	FabG1	EC-1.1.1.100
342	ICSMAT9	Icosanoyl-[acyl-carrier protein]:malonyl-CoA C-acyltransferase	[c] : 2ticsacp + h + nadh --> icsACP + nad	Lipid Metabolism	338	FabI	EC-1.3.1.10
343	KAS11	b-ketoacyl synthetase (Iso-C17:0)	[c] : (17) h + ivcoa + (6) malcoa + (12) nadph --> (6) co2 + (7) coa + fa11 + (5) h2o + (12) nadp	Lipid Metabolism	338	FabI	EC-2.3.1.41
344	KAS17	b-ketoacyl synthetase (n-C18:1)	[c] : accoa + (22) h + (8) malcoa + (15) nadph --> (8) co2 + (9) coa + (7) h2o + (15) nadp + ocdcea	Lipid Metabolism	338	FabI	EC-2.3.1.41
345	MACPMT	Malonyl-CoA:[acyl-carrier-protein] S-malonyltransferase	[c] : ACP + malcoa --> coa + malACP	Lipid Metabolism	339	FabD	EC-2.3.1.39
346	MCMAT2	Butyryl-[acyl-carrier protein]:malonyl-[acyl-carrier-protein]C-acyltransferase (decarboxylating)	[c] : butACP + h + malACP --> 3oxhACP + ACP + co2	Lipid Metabolism	274	FabF	EC-2.3.1.41
347	MCMAT3	Hexanoyl-[acyl-carrier protein]:malonyl-[acyl-carrier-protein] C-acyltransferase	[c] : h + hexACP + malACP --> 3oxocACP + ACP + co2	Lipid Metabolism	274	FabF	EC-2.3.1.41
348	MCMAT4	Octanoyl-[acyl-carrier protein]:malonyl-[acyl-carrier-protein] C-acyltransferase (decarboxylating)	[c] : h + malACP + octACP --> 3oxdeACP + ACP + co2	Lipid Metabolism	274	FabF	EC-2.3.1.41
349	MCMAT5	Decanoyl-[acyl-carrier protein]:malonyl-[acyl-carrier-protein] C-acyltransferase	[c] : decACP + h + malACP --> 3oxddACP + ACP + co2	Lipid Metabolism	274	FabF	EC-2.3.1.41

No	Abbreviation	Name	Equation	Subsystem	Model gene number	Protein	Protein Classification
350	MCMAT6	Dodecanoyl-[acyl-carrier-protein]:malonyl-[acyl-carrier-protein] C-acyltransferase	[c] : ddcaACP + h + malACP --> 3oxtdACP + ACP + co2	Lipid Metabolism	274	FabF	EC-2.3.1.41
351	MCMAT7	Tetradecanoyl-[acyl-carrier-protein]:malonyl-[acyl-carrier-protein] C-acyltransferase	[c] : h + malACP + myrsACP --> 3oxhdACP + ACP + co2	Lipid Metabolism	274	FabF	EC-2.3.1.41
352	MCMAT8	Hexadecanoyl-[acyl-carrier-protein]:malonyl-[acyl-carrier-protein] C-acyltransferase	[c] : h + malACP + palmACP --> 3oxocdacp + ACP + co2	Lipid Metabolism	274	FabF	EC-2.3.1.41
353	MCMAT9	Octadecanoyl-[acyl-carrier-protein]:malonyl-[acyl-carrier-protein] C-acyltransferase	[c] : h + malACP + ocdACP --> 3oxicsacp + ACP + co2	Lipid Metabolism	274	FabF	EC-2.3.1.41
354	MPGSP	Mannosyl-3-phosphoglycerate synthase	[c] : 3pg + gdpman + (2) h <==> 2m3pg + gdp	Lipid Metabolism	356	MpgSP	EC-2.4.1.217
355	OCDMAT8	Octadecanoyl-[acyl-carrier protein]:malonyl-CoA C-acyltransferase	[c] : 2tocdACP + h + nadh --> nad + ocdACP	Lipid Metabolism	338	FabI	EC-1.3.1.10
356	OCMAT3	Octanoyl-[acyl-carrier protein]:malonyl-CoA C-acyltransferase	[c] : 2toceACP + h + nadh --> nad + octACP	Lipid Metabolism	338	FabI	EC-1.3.1.10
357	OCTT	trans-octaprenyltranstransferase	[c] : ipdp + octdp --> nondp + ppi	Lipid Metabolism	67	HepT	EC-2.5.1.11
358	PGPPH	Phosphatidylglycerophosphate phosphohydrolase	[c] : h2o + pglyp --> pgly + pi	Lipid Metabolism	361	PgpA	EC-3.1.3.27
359	PHCYT	phosphatidate cytidylyltransferase	[c] : 12dag3p + ctp + h --> cdpdag + ppi	Lipid Metabolism	59	CdsA	EC-2.7.7.41
360	PPTT	trans-pentaprenyltranstransferase	[c] : ipdp + pendp --> hexdp + ppi	Lipid Metabolism	67	HepT	EC-2.5.1.33
361	TDMAT6	Tetradecanoyl-[acyl-carrier protein]:malonyl-CoA C-acyltransferase	[c] : myrsACP + nad <==> h + nadh + tdeACP	Lipid Metabolism	338	FabI	EC-1.3.1.10
362	ADD	adenine deaminase	[c] : ade + h + h2o --> hxan + nh4	Nucleotide Metabolism	208	AdeC	EC-3.5.4.2
363	ADK1	adenylate kinase	[c] : amp + atp <==> (2) adp	Nucleotide Metabolism	111	Adk	EC-2.7.4.3

No	Abbreviation	Name	Equation	Subsystem	Model gene number	Protein	Protein Classification
364	ADK2	adenylate kinase (Inorganic triphosphate)	[c] : amp + pppi <==> adp + ppi	Nucleotide Metabolism	111	Adk	EC-2.7.4.3
365	ADK3	guanylate kinase (aMP:gTP)	[c] : amp + gtp <==> adp + gdp	Nucleotide Metabolism	111	Adk	EC-2.7.4.8
366	ADK4	adentylate kinase (ITP)	[c] : amp + itp <==> adp + idp	Nucleotide Metabolism	111	Adk	
367	ADNCYC	adenylate cyclase	[c] : atp --> camp + ppi	Nucleotide Metabolism	152	CyaA	EC-4.6.1.1
368	ADNK1	adenosine kinase	[c] : adn + atp --> adp + amp + h	Nucleotide Metabolism	111	Adk	EC-2.7.1.20
369	ADPRDP	ADPribose diphosphatase	[c] : adprib + h2o --> amp + (2) h + r5p	Nucleotide Metabolism	99	NudF	EC-3.6.1.13
370	ADPT	adenine phosphoribosyltransferase	[c] : ade + prpp --> amp + ppi	Nucleotide Metabolism	311	Apt	EC-2.4.2.7
371	ADSL1	adenylosuccinate lyase	[c] : dcamp --> amp + fum	Nucleotide Metabolism	228	PurB	EC-4.3.2.2
372	ADSL2	adenylosuccinate lyase	[c] : 25aics --> aicar + fum	Nucleotide Metabolism	228	PurB	EC-4.3.2.2
373	ADSS	adenylosuccinate synthetase	[c] : asp-L + gtp + imp --> dcamp + gdp + (2) h + pi	Nucleotide Metabolism	278	PurA	EC-6.3.4.4
374	AICART	phosphoribosylaminoimidazolecarboxamide formyltransferase	[c] : 10fthf + aicar <==> fprica + thf	Nucleotide Metabolism	369	PurH	EC-2.1.2.3
375	AIRC	phosphoribosylaminoimidazole carboxylase	[c] : air + co2 --> 5aizc + h	Nucleotide Metabolism	227	PurE	EC-4.1.1.21
376	ASPCT	aspartate carbamoyltransferase	[c] : asp-L + cbp --> cbasp + h + pi	Nucleotide Metabolism	314	PyrB	EC-2.1.3.2
377	CBPSr	carbamoyl-phosphate synthase (glutamine-hydrolysing)	[c] : (2) atp + gln-L + h2o + hco3 <==> (2) adp + cbp + glu-L + (2) h + pi	Nucleotide Metabolism	317+316	CarAB	EC-6.3.5.5
378	CTPS1	CTP synthase (NH3)	[c] : atp + nh4 + utp --> adp + ctp + (2) h + pi	Nucleotide Metabolism	366	PyrG	EC-6.3.4.2
379	CTPS2	CTP synthase (glutamine)	[c] : atp + gln-L + h2o + utp --> adp + ctp + glu-L + (2) h + pi	Nucleotide Metabolism	366	PyrG	EC-6.3.4.2
380	CYTK1	cytidylate kinase (CMP)	[c] : atp + cmp <==> adp + cdp	Nucleotide Metabolism	105	Cmk	EC-2.7.4.14
381	CYTK2	cytidylate kinase (dCMP)	[c] : atp + dcmp <==> adp + dcdp	Nucleotide Metabolism	105	Cmk	EC-2.7.4.14
382	DADK	deoxyadenylate kinase	[c] : atp + damp <==> adp + dadp	Nucleotide Metabolism	111	Adk	EC-2.7.4.11
383	DCMPDA2ir	dCMP deaminase	[c] : dcmp + h + h2o --> dump + nh4	Nucleotide Metabolism	76	ComEB	EC-3.5.4.12
384	DCMPDA3ir	dCMP deaminase (CMP)	[c] : cmp + h + h2o --> nh4 + ump	Nucleotide Metabolism	76	ComEB	EC-3.5.4.12
385	DGTPH	dGTPase	[c] : dgtp + h2o --> dgsn + pppi	Nucleotide Metabolism	128, 500	Dgt	EC-3.1.5.1
386	DHORDf	dihydroorotate dehydrogenase	[c] : dhor-S + (2) fdox <==> (2) fdred + (2) h + orot	Nucleotide Metabolism	10, 318, 358	PyrD, PyrK	EC-1.3.99.11
387	DHORTS	dihydroorotase	[c] : dhor-S + h2o <==> cbasp + h	Nucleotide Metabolism	315	PyrC	EC-3.5.2.3
388	DTMPK	dTMP kinase	[c] : atp + dtmp <==> adp + dtdp	Nucleotide Metabolism	202	Tmk	EC-2.7.4.9

No	Abbreviation	Name	Equation	Subsystem	Model gene number	Protein	Protein Classification
389	DUTPDP	dUTP diphosphatase	[c] : dutp + h2o --> dump + h + ppi	Nucleotide Metabolism	78	Dutec	EC-3.6.1.23
390	FDTS	Thymidylate synthase (FAD)	[c] : dump + fadh2 + mlthf --> dtmp + fad + thf	Nucleotide Metabolism	381	ThyX	EC-Undetermined
391	FTHFD	formyltetrahydrofolate deformylase	[c] : 10fthf + h2o --> for + h + thf	Nucleotide Metabolism	329	PurUec	EC-3.5.1.10
392	G1PTMT	glucose-1-phosphate thymidylyltransferase	[c] : dttp + g1p + h --> dtdpglc + ppi	Nucleotide Metabolism	121, 122	RfbA	EC-2.7.7.24
393	GARFT	phosphoribosylglycinamide formyltransferase	[c] : 10fthf + gar <==> fgam + h + thf	Nucleotide Metabolism	179, 329	PurN	EC-2.1.2.2
394	GLUPRT	glutamine phosphoribosyldiphosphate amidotransferase	[c] : gln-L + h2o + prpp --> glu-L + ppi + pram	Nucleotide Metabolism	367	PurF	EC-2.4.2.14
395	GMPR	GMP reductase	[c] : gmp + (2) h + nadph --> imp + nadp + nh4	Nucleotide Metabolism	65	GuaC	EC-1.7.1.7
396	GMPS2	GMP synthase (glutamine-hydrolysing)	[c] : atp + gln-L + h2o + xmp --> amp + glu-L + gmp + (2) h + ppi	Nucleotide Metabolism	225	GuaA	EC-6.3.5.2
397	GUAPRT	guanine phosphoribosyltransferase	[c] : gua + prpp --> gmp + ppi	Nucleotide Metabolism	196, 209	Hpt	
398	HXPRT	hypoxanthine phosphoribosyltransferase (Hypoxanthine)	[c] : hxan + prpp --> imp + ppi	Nucleotide Metabolism	196, 209	Hpt	EC-2.4.2.8
399	IMPCir	IMP cyclohydrolase	[c] : fprica --> h2o + imp	Nucleotide Metabolism	369	PurH	EC-3.5.4.10
400	IMPD	IMP dehydrogenase	[c] : h2o + imp + nad --> h + nadh + xmp	Nucleotide Metabolism	65	GuaB	EC-1.1.1.205
401	ITPASE	dITP pyrophosphatase	[c] : ditp + h2o --> dimp + ppi	Nucleotide Metabolism	328	IxpP	EC-Undetermined
402	MTAP	5'-methylthioadenosine phosphorylase	[c] : 5mta + pi --> 5mdr1p + ade	Nucleotide Metabolism	117	MtaP	EC-2.4.2.28
403	NAMNPP	nicotinic acid mononucleotide pyrophosphorylase	[c] : atp + h2o + nac + prpp --> adp + nicrnt + pi + ppi	Nucleotide Metabolism	370	PncB	
404	NCTPPRT	nicotinate phosphoribosyltransferase	[c] : nicrnt + ppi <==> h + nac + prpp	Nucleotide Metabolism	370	PncB	EC-2.4.2.11
405	NDPK1	nucleoside-diphosphate kinase (ATP:GDP)	[c] : atp + gdp <==> adp + gtp	Nucleotide Metabolism	68	Ndk	EC-2.7.4.6
406	NDPK2	nucleoside-diphosphate kinase (ATP:UDP)	[c] : atp + udp <==> adp + utp	Nucleotide Metabolism	68	Ndk	EC-2.7.4.6
407	NDPK3	nucleoside-diphosphate kinase (ATP:CDP)	[c] : atp + cdp <==> adp + ctp	Nucleotide Metabolism	68	Ndk	EC-2.7.4.6

No	Abbreviation	Name	Equation	Subsystem	Model gene number	Protein	Protein Classification
408	NDPK4	nucleoside-diphosphate kinase (ATP:dTDP)	[c] : atp + dtdp <==> adp + dttp	Nucleotide Metabolism	68	Ndk	EC-2.7.4.6
409	NDPK5	nucleoside-diphosphate kinase (ATP:dGDP)	[c] : atp + dgdp <==> adp + dgtp	Nucleotide Metabolism	68	Ndk	EC-2.7.4.6
410	NDPK6	nucleoside-diphosphate kinase (ATP:dUDP)	[c] : atp + dudp <==> adp + dutp	Nucleotide Metabolism	68	Ndk	EC-2.7.4.6
411	NDPK7	nucleoside-diphosphate kinase (ATP:dCDP)	[c] : atp + dcdp <==> adp + dctp	Nucleotide Metabolism	68	Ndk	EC-2.7.4.6
412	NDPK8	nucleoside-diphosphate kinase (ATP:dADP)	[c] : atp + dadp <==> adp + datp	Nucleotide Metabolism	68	Ndk	EC-2.7.4.6
413	NTPP1	Nucleoside triphosphate pyrophosphorylase (dgtp)	[c] : dgtp + h2o --> dgmp + h + ppi	Nucleotide Metabolism	409	MazG	
414	NTPP2	Nucleoside triphosphate pyrophosphorylase (gtp)	[c] : gtp + h2o --> gmp + h + ppi	Nucleotide Metabolism	409	MazG	
415	NTPP3	dCTP diphosphatase	[c] : dctp + h2o --> dcmp + h + ppi	Nucleotide Metabolism	409	MazG	EC-3.6.1.12
416	NTPP4	Nucleoside triphosphate pyrophosphorylase (ctp)	[c] : ctp + h2o --> cmp + h + ppi	Nucleotide Metabolism	409	MazG	
417	NTPP5	Nucleoside triphosphate pyrophosphorylase (datp)	[c] : datp + h2o --> damp + h + ppi	Nucleotide Metabolism	409	MazG	
418	NTPP6	Nucleoside triphosphate pyrophosphorylase (atp)	[c] : atp + h2o --> amp + h + ppi	Nucleotide Metabolism	409	MazG	
419	NTPP7	Nucleoside triphosphate pyrophosphorylase (dttp)	[c] : dttp + h2o --> dtmp + h + ppi	Nucleotide Metabolism	409	MazG	
420	NTPP8	Nucleoside triphosphate pyrophosphorylase (utp)	[c] : h2o + utp --> h + ppi + ump	Nucleotide Metabolism	409	MazG	
421	NTPTP2	Nucleoside triphosphate tripolyhydrolase	[c] : gtp + h2o --> gsn + pppi	Nucleotide Metabolism	128, 500	Dgt	EC-3.1.5.1
422	OMPDC	orotidine-5'-phosphate decarboxylase	[c] : h + orot5p --> co2 + ump	Nucleotide Metabolism	6	PyrF	EC-4.1.1.23
423	ORPT	orotate phosphoribosyltransferase	[c] : orot5p + ppi <==> orot + prpp	Nucleotide Metabolism	311	PyrE	EC-2.4.2.10
424	PRAGS	phosphoribosylglycinamide synthetase	[c] : atp + gly + pram --> adp + gar + h + pi	Nucleotide Metabolism	226	PurD	EC-6.3.4.13
425	PRAIS	phosphoribosylaminoimidazole synthetase	[c] : atp + fpram --> adp + air + h + pi	Nucleotide Metabolism	368	PurM	EC-6.3.3.1
426	PRASCS	phosphoribosylaminoimidazolesuccinocarboxamide synthase	[c] : 5aizc + asp-L + atp <==> 25aics + adp + h + pi	Nucleotide Metabolism	229	PurC	EC-6.3.2.6

No	Abbreviation	Name	Equation	Subsystem	Model gene number	Protein	Protein Classification
427	PRFGS	phosphoribosylformylglycinamidine synthase	[c] : atp + fgam + gln-L + h2o --> adp + fpram + glu-L + (2) h + pi	Nucleotide Metabolism	63, 62	PurQ+PurL	EC-6.3.5.3
428	PRPPS	phosphoribosylpyrophosphate synthetase	[c] : atp + r5p <==> amp + h + prpp	Nucleotide Metabolism	86	Prs	EC-2.7.6.1
429	PSUDS	pseudouridylate synthase	[c] : r5p + ura --> h2o + psd5p	Nucleotide Metabolism	359, 112, 207, 281	RluB, RluD	EC-4.2.1.70
430	RNDR1	ribonucleoside-diphosphate reductase (ADP)	[c] : adp + trdrd --> dadp + h2o + trdox	Nucleotide Metabolism	428, 151, 52	NrdEF	EC-1.17.4.1
431	RNDR2	ribonucleoside-diphosphate reductase (GDP)	[c] : gdp + trdrd --> dgdp + h2o + trdox	Nucleotide Metabolism	428, 151, 52	NrdEF	EC-1.17.4.1
432	RNDR3	ribonucleoside-diphosphate reductase (CDP)	[c] : cdp + trdrd --> dcdp + h2o + trdox	Nucleotide Metabolism	428, 151, 52	NrdEF	EC-1.17.4.1
433	RNDR4	ribonucleoside-diphosphate reductase (UDP)	[c] : trdrd + udp --> dudp + h2o + trdox	Nucleotide Metabolism	428, 151, 52	NrdEF	EC-1.17.4.1
434	RNTR1	ribonucleoside-triphosphate reductase (ATP)	[c] : atp + trdrd --> datp + h2o + trdox	Nucleotide Metabolism	52, 364	NrdD	EC-1.17.4.2
435	RNTR2	ribonucleoside-triphosphate reductase (GTP)	[c] : gtp + trdrd --> dgtp + h2o + trdox	Nucleotide Metabolism	52, 364	NrdD	EC-1.17.4.2
436	RNTR3	ribonucleoside-triphosphate reductase (CTP)	[c] : ctp + trdrd --> dctp + h2o + trdox	Nucleotide Metabolism	52, 364	NrdD	EC-1.17.4.2
437	RNTR4	ribonucleoside-triphosphate reductase (UTP)	[c] : trdrd + utp --> dutp + h2o + trdox	Nucleotide Metabolism	52, 364	NrdD	EC-1.17.4.2
438	TMDK1	thymidine kinase (ATP:thymidine)	[c] : atp + thymd --> adp + dtmp + h	Nucleotide Metabolism	202	Tmk	EC-2.7.1.21
439	TMDS	thymidylate synthase	[c] : dump + mlthf --> dhf + dtmp	Nucleotide Metabolism	214	ThyA	EC-2.1.1.45
440	UMPK	UMP kinase	[c] : atp + ump <==> adp + udp	Nucleotide Metabolism	105	Cmk	
441	UPPRT	uracil phosphoribosyltransferase	[c] : prpp + ura --> ppi + ump	Nucleotide Metabolism	313	Upp2	EC-2.4.2.9
442	URIDK1	uridylate kinase (UMP)	[c] : atp + ump --> adp + udp	Nucleotide Metabolism	61	PyrH	
443	URIDK2	uridylate kinase (dUMP)	[c] : atp + dump --> adp + dudp	Nucleotide Metabolism	61	PyrH	
444	XTPASE	XTP pyrophosphatase	[c] : h2o + xtp --> h + ppi + xmp	Nucleotide Metabolism	328	IxpP	EC-Undetermined

No	Abbreviation	Name	Equation	Subsystem	Model gene number	Protein	Protein Classification
445	E4PD	Erythrose 4-phosphate dehydrogenase	[c] : e4p + h2o + nad <==> 4per + (2) h + nadh	Pentose Phosphate Pathway	143	Epd	
446	GTPDK	GTP diphosphokinase	[c] : atp + gtp --> amp + gdptp + h	Pentose Phosphate Pathway	2	RelA	EC-2.7.6.5
447	RPE	ribulose 5-phosphate 3-epimerase	[c] : ru5p-D <==> xu5p-D	Pentose Phosphate Pathway	158	SgcE	EC-5.1.3.1
448	RPI	ribose-5-phosphate isomerase	[c] : r5p <==> ru5p-D	Pentose Phosphate Pathway	159	RpiB	EC-5.3.1.6
449	TAL	transaldolase	[c] : g3p + s7p <==> e4p + f6p	Pentose Phosphate Pathway	365	Tal	EC-2.2.1.2
450	TKT1	transketolase	[c] : r5p + xu5p-D <==> g3p + s7p	Pentose Phosphate Pathway	160	TktB	EC-2.2.1.1
451	TKT2	transketolase	[c] : e4p + xu5p-D <==> f6p + g3p	Pentose Phosphate Pathway	160	TktB	EC-2.2.1.1
452	ANTIMt1	antimonite transporter via uniport	antim[c] --> antim[e]	Transport	437	ArsB2	TC-2.A.59
453	ARSNAt1	Arsenate transporter	arsna[c] --> arsna[e]	Transport	437	ArsB2	
454	ARSt1	arsenite transporter via uniport	arsni2[c] --> arsni2[e]	Transport	437, 248	ArsB, ArsB2	
455	ATPS3r	ATPase	adp[c] + (3) h[e] + pi[c] <==> atp[c] + (2) h[c] + h2o[c]	Transport	136+137+135+133+132+130+134+131	AtpABCDEFGHI	EC-Undetermined, TC-Undetermined
456	CBL1abc	Cob(1)alamin transport via ABC system	atp[c] + cbl1[e] + h2o[c] --> adp[c] + cbl1[c] + h[c] + pi[c]	Transport	525, 162, 431, 524, 161, 480, 526, 163	BtuF+BtuD+BtuC	
457	COBALTt5	cobalt transport in/out via permease (no H+)	cobalt2[c] <==> cobalt2[e]	Transport	82, 142	CorA1	TC-1.A.35
458	Cuabc	Copper transport via ABC system	atp[c] + cu2[e] + h2o[c] --> adp[c] + cu2[c] + h[c] + pi[c]	Transport	271	Cudp	TC-3.A.3
459	FE2abc	iron (II) transport via ABC system	atp[c] + fe2[e] + h2o[c] --> adp[c] + fe2[c] + h[c] + pi[c]	Transport	387	FeoB1	
460	GLNabc	L-glutamine transport via ABC system	atp[c] + gln-L[e] + h2o[c] --> adp[c] + gln-L[c] + h[c] + pi[c]	Transport	81, 80, 79	GlnP+GlnH+GlnQ	TC-3.A.1.3
461	GLYBt6	betaine (glycine betaine) transport in/out via proton symport	glyb[e] + h[e] <==> glyb[c] + h[c]	Transport	344	ProPec	TC-2.A.1.6
462	ILEabc	L-isoleucine transport via ABC system	atp[c] + h2o[c] + ile-L[e] --> adp[c] + h[c] + ile-L[c] + pi[c]	Transport	261+265+266+263+264+267	BraB, AzlCD	
463	Kt6	potassium transport in/out via proton symport	h[e] + k[e] <==> h[c] + k[c]	Transport	406, 180	KefC2, KefC	TC-2.A.38

No	Abbreviation	Name	Equation	Subsystem	Model gene number	Protein	Protein Classification
464	LEUabc	L-leucine transport via ABC system	atp[c] + h2o[c] + leu-L[e] --> adp[c] + h[c] + leu-L[c] + pi[c]	Transport	261+265+266+263+264+267	AzlCD, BraB	
465	MGt5	magnesium transport in/out via permease (no H+)	mg2[c] <==> mg2[e]	Transport	82, 142	CorA1	TC-1.A.35
466	MNabc	manganese transport via ABC system	atp[c] + h2o[c] + mn2[e] --> adp[c] + h[c] + mn2[c] + pi[c]	Transport	156, 157, 155	ZnuC+ZnuA+ZnuB	TC-3.A.1.15
467	NH4t	ammonium transport via diffusion	nh4[e] <==> nh4[c]	Transport	291	AmtB	
468	NH4t3	ammonium transport out via K+ antiport	k[c] + nh4[e] --> k[e] + nh4[c]	Transport	291	AmtB	TC-2.A.49
469	NIabc	nickel transport via ABC system	atp[c] + h2o[c] + ni2[e] --> adp[c] + h[c] + ni2[c] + pi[c]	Transport	432, 386, 434, 384, 383, 435, 138, 436, 382, 433, 385	NikA+NikC+NikD+NikB+NikE	
470	PIabc	phosphate transport via ABC system	atp[c] + h2o[c] + pi[e] --> adp[c] + h[c] + (2) pi[c]	Transport	34+31+33+32	PstABCS	TC-3.A.1.7
471	PIt6	phosphate transport in/out via proton symporter	h[e] + pi[e] <==> h[c] + pi[c]	Transport	8	PitA	TC-2.A.20
472	PPA_1	inorganic diphosphatase (one proton translocation)	h2o[c] + ppi[c] --> h[e] + (2) pi[c]	Transport	200, 204	Ppa1	EC-3.6.1.1, TC-3.A.10
473	PROt6	L-proline transport in/out via proton symport	h[e] + pro-L[e] <==> h[c] + pro-L[c]	Transport	344	ProPec	TC-2.A.3.1
474	SULabc	sulfate transport via ABC system	atp[c] + h2o[c] + so4[e] --> adp[c] + h[c] + pi[c] + so4[c]	Transport	213	CysAP	TC-3.A.1.6
475	TSULabc	thiosulfate transport via ABC system	atp[c] + h2o[c] + tsul[e] --> adp[c] + h[c] + pi[c] + tsul[c]	Transport	213	CysAP	TC-3.A.1.6
476	VALabc	L-valine transport via ABC system	atp[c] + h2o[c] + val-L[e] --> adp[c] + h[c] + pi[c] + val-L[c]	Transport	261+265+266+263+264+267	BraB, AzlCD	
477	ZN2abc2	zinc transport in via ABC system	atp[c] + h2o[c] + zn2[e] --> adp[c] + h[c] + pi[c] + zn2[c]	Transport	156, 157, 155	ZnuC+ZnuA+ZnuB	TC-3.A.1.15

Table 10. List of Reactions of *i* AI549 Associated with Dispensable Genes (Dispensable Reactions)

No	Abbreviation	Name	Equation	Subsystem	Gene	Protein	Protein Classification
1	ASPO1	L-aspartate oxidase	[c] : asp-L + nad --> (2) h + iasp + nadh	Cofactor and Prosthetic Group Biosynthesis	420	NadB	
2	ASPO3	L-aspartate oxidase	[c] : asp-L + ubq8 --> h + iasp + ubq8h2	Cofactor and Prosthetic Group Biosynthesis	420	NadB	
3	ASPO4	L-aspartate oxidase	[c] : asp-L + mqn8 --> h + iasp + mql8	Cofactor and Prosthetic Group Biosynthesis	420	NadB	
4	ASPO5	L-aspartate oxidase	[c] : asp-L + fum --> h + iasp + succ	Cofactor and Prosthetic Group Biosynthesis	420	NadB	
5	ASPO6	L-aspartate oxidase	[c] : asp-L + o2 --> h + h2o2 + iasp	Cofactor and Prosthetic Group Biosynthesis	420	NadB	
6	ORNCD	ornithine cyclodeaminase	[c] : orn-L --> nh4 + pro-L	Cofactor and Prosthetic Group Biosynthesis	414	Ocd	EC-4.3.1.12
7	THZPSN	thiazole phosphate synthesis	[c] : atp + cys-L + dxyl5p + tyr-L --> 4hba + 4mpetz + ala-L + amp + co2 + h + h2o + ppi	Cofactor and Prosthetic Group Biosynthesis	417	IscS	
8	CPC6MT	precorrin-6Y C5,15-methyltransferase (decarboxylating)	[c] : (2) amet + codhpre6 --> (2) ahcys + co2 + copre8 + h	Cofactor and Prosthetic Group Biosynthesis	416	CbiE	EC-2.1.1.132
9	PDX5PO	pyridoxine 5'-phosphate oxidase	[c] : o2 + pdx5p <==> h + h2o2 + pydx5p	Cofactor and Prosthetic Group Biosynthesis	418	PdxHec	EC-1.4.3.5
10	PMPK	phosphomethylpyrimidine kinase	[c] : 4ampm + atp --> 2mahmp + adp	Cofactor and Prosthetic Group Biosynthesis	421	ThiD	EC-2.7.4.7
11	HISTP	histidinol-phosphatase	[c] : h2o + hisp --> histd + pi	Amino Acid Metabolism	425	HisJ	EC-3.1.3.15
12	THRA	threonine aldolase	[c] : thr-L <==> acald + gly	Amino Acid Metabolism	415	LtaA	EC-4.1.2.5
13	THRLAD	L-allo-threonine aldolase	[c] : thr-LA --> acald + gly	Amino Acid Metabolism	415	LtaA	
14	SUCOAS	succinyl-CoA synthetase (ADP-forming)	[c] : atp + coa + succ <==> adp + pi + succoa	Citrate Cycle (TCA)	419	SucCD1	EC-6.2.1.5

No	Abbreviation	Name	Equation	Subsystem	Gene	Protein	Protein Classification
15	FRD5	succinate dehydrogenase (menaquinone 7)	[c] : mqn7 + succ <==> fum + mql7	Citrate Cycle (TCA)	420	Frd	EC-1.3.5.1
16	PFLi	pyruvate formate lyase	[c] : coa + pyr --> accoa + for	Carbohydrate Metabolism	422	PflC	EC-Undetermined
17	S7PI	sedoheptulose 7-phosphate isomerase	[c] : s7p <==> gmh7p	Pentose Phosphate Pathway	424	GmhA	
18	OBTFL	2-Oxobutanoate formate lyase	[c] : 2obut + coa --> for + ppcoa	Energy Metabolism	422	PflC	

Table 11. List of Reactions of *i* AI549 Associated with Unique Genes (Unique Reactions)

No	Abbreviation	Name	Equation	Subsystem	Gene	Protein	Protein Classification
1	NITr	nitrogenase	[c] : (16) atp + (8) fdred + (16) h2o + n2 <==> (16) adp + (8) fdox + (6) h + h2 + (2) nh4 + (16) pi	Energy Metabolism	495, 492, 493, 490, 489, 491, 494	Nif	EC-1.18.6.1
2	MOBDabc	molybdate transport via ABC system	atp[c] + h2o[c] + mobd[e] --> adp[c] + h[c] + mobd[c] + pi[c]	Transport	496+498+497	ModABC	TC-3.A.1.8

Table 12. Detailed List of Metabolites of *i* AI549

No	Abbreviation	Name	Formula	KEGG ID
1	10fthf	10-Formyltetrahydrofolate	C20H21N7O7	C00234
2	12dag3p	1,2-Diacyl-sn-glycerol 3-phosphate	C5H7O8PR2	C00416
3	12dgr	1,2-Diacylglycerol	C5H6O5R2	C00641
4	13dpg	3-Phospho-D-glyceroyl phosphate	C3H4O10P2	C00236
5	1ap2ol	1-Aminopropan-2-ol	C3H9NO	C05771
6	1ap2olp	D-1-Aminopropan-2-ol O-phosphate	C3H10NO4P	C04122
7	1pyr5c	1-Pyrroline-5-carboxylate	C5H6NO2	C03912
8	23dhdp	2,3-Dihydrodipicolinate	C7H5NO4	C03340
9	23dhmb	(R)-2,3-Dihydroxy-3-methylbutanoate	C5H9O4	C04272
10	23dhmp	(R)-2,3-Dihydroxy-3-methylpentanoate	C6H11O4	C06007
11	25aics	(S)-2-[5-Amino-1-(5-phospho-D-ribosyl)imidazole-4-carboxamido]succinate	C13H15N4O12P	C04823
12	25dhpp	2,5-Diamino-6-hydroxy-4-(5'-phosphoribosylamino)-pyrimidine	C9H16N5O8P	C01304
13	26dap-LL	LL-2,6-Diaminoheptanedioate	C7H14N2O4	C00666
14	26dap-M	meso-2,6-Diaminoheptanedioate	C7H14N2O4	C00680
15	2ahbut	(S)-2-Aceto-2-hydroxybutanoate	C6H9O4	C06006
16	2ahhmd	2-Amino-4-hydroxy-6-hydroxymethyl-7,8-dihydropteridine diphosphate	C7H11N5O8P2	C04807

No	Abbreviation	Name	Formula	KEGG ID
17	2ahhmp	2-Amino-4-hydroxy-6-hydroxymethyl-7,8-dihydropteridine	C7H9N5O2	C01300
18	2beACP	But-2-enoyl-[acyl-carrier protein]	C27H44O	C04246
19	2c25dho	2-Carboxy-2,5-dihydro-5-oxofuran-2-acetate	C7H6O6	C01278
20	2cpr5p	1-(2-Carboxyphenylamino)-1-deoxy-D-ribulose 5-phosphate	C12H13NO9P	C01302
21	2dda7p	2-Dehydro-3-deoxy-D-arabino-heptonate 7-phosphate	C7H10O10P	C04691
22	2dhp	2-Dehydropantoate	C6H9O4	C00966
23	2dmmq8	2-Demethylmenaquinone 8	C50H70O2	C05818
24	2dmmql8	2-Demethylmenaquinol 8	C50H72O2	0
25	2ippm	2-Isopropylmaleate	C7H8O4	C02631
26	2m3pg	2-(alpha-D-Mannosyl)-3-phosphoglycerate	C9H17O12P	C11516
27	2mahmp	2-Methyl-4-amino-5-hydroxymethylpyrimidine diphosphate	C6H8N3O7P2	C04752
28	2mbcoa	2-Methylbutanoyl-CoA	C26H44N7O17P3S	C01033
29	2mcacn	cis-2-Methylaconitate	C7H5O6	C04225
30	2me4p	2-C-methyl-D-erythritol 4-phosphate	C5H11O7P	C11434
31	2mecdp	2-C-methyl-D-erythritol 2,4-cyclodiphosphate	C5H10O9P2	C11453

No	Abbreviation	Name	Formula	KEGG ID
32	2o3mpt	(R)-2-Oxo-3-methylpentanoate	C6H10O3	C06008
33	2obut	2-Oxobutanoate	C4H5O3	C00109
34	2ohph	2-Octaprenyl-6-hydroxyphenol	C46H70O2	C05811
35	2ombzl	2-Octaprenyl-6-methoxy-1,4-benzoquinol	C47H72O3	0
36	2omhmbl	2-Octaprenyl-3-methyl-5-hydroxy-6-methoxy-1,4-benzoquinol	C48H74O4	0
37	2ommbl	2-Octaprenyl3-methyl-6-methoxy- 1,4-benzoquinol	C48H74O3	0
38	2omph	2-Octaprenyl-6-methoxyphenol	C47H72O2	C05812
39	2p4c2me	2-phospho-4-(cytidine 5'-diphospho)-2-C-methyl-D-erythritol	C14H22N3O17P3	0
40	2pg	D-Glycerate 2-phosphate	C3H4O7P	C00631
41	2tddACP	trans-Dodec-2-enoyl-[acyl-carrier protein]	C12H21OSR	C05758
42	2tdeACP	trans-Dec-2-enoyl-[acyl-carrier protein]	C10H17OSR	C05754
43	2theACP	trans-Hex-2-enoyl-[acp]	C6H9OSR	C05748
44	2ticsacp	trans-Icos-2-enoyl [aceyl-carrier protein]	C20H37OX	0
45	2tocdACP	trans-Octadec-2-enoyl-[acyl-carrier-protein]	C18H33OSR	C16221
46	2toceACP	trans-Oct-2-enoyl-[acp]	C8H13OSR	C05751
47	34hpp	3-(4-Hydroxyphenyl)pyruvate	C9H7O4	C01179
48	36dahx	(3S)-3,6-Diaminohexanoate	C6H14N2O2	C01142

No	Abbreviation	Name	Formula	KEGG ID
49	3c2hmp	3-Carboxy-2-hydroxy-4-methylpentanoate	C7H10O5	C04411
50	3c3hmp	3-Carboxy-3-hydroxy-4-methylpentanoate	C7H10O5	C02504
51	3c4mop	3-Carboxy-4-methyl-2-oxopentanoate	C7H8O5	C04236
52	3dhq	3-Dehydroquinate	C7H9O6	C00944
53	3dhsk	3-Dehydroshikimate	C7H7O5	C02637
54	3fpyr	3-Fumarylpyruvate	C7H6O6	C02514
55	3hbACP	(3R)-3-Hydroxybutanoyl-[acyl-carrier protein]	C4H7O2SR	C04618
56	3hddACP	(R)-3-Hydroxydodecanoyl-[acyl-carrier protein]	C12H23O2SR	C05757
57	3hdeACP	(3R)-3-Hydroxydecanoyl-[acyl-carrier protein]	C10H19O2SR	C04619
58	3hhACP	(R)-3-Hydroxyhexanoyl-[acp]	C6H11O2SR	C05747
59	3hicsacp	(3R)-3-Hydroxyicosanoyl [aceyl-carrier protein]	C20H39O2X	0
60	3hocACP	(R)-3-Hydroxyoctanoyl-[acyl-carrier protein]	C8H15O2SR	C04620
61	3hocdacp	(3R)-3-Hydroxyoctadecanoyl-[acyl-carrier protein]	C18H35O2X	0
62	3hpaACP	(3R)-3-Hydroxypalmitoyl-[acyl-carrier protein]	C16H31O2SR	C04633

No	Abbreviation	Name	Formula	KEGG ID
63	3htdACP	(3R)-3-Hydroxytetradecanoyl-[acyl-carrier protein]	C14H27O2SR	C04688
64	3ig3p	C'-(3-Indolyl)-glycerol 3-phosphate	C11H12NO6P	C03506
65	3mob	3-Methyl-2-oxobutanoate	C5H7O3	C00141
66	3mop	(S)-3-Methyl-2-oxopentanoate	C6H9O3	C00671
67	3oxddACP	3-Oxododecanoyl-[acyl-carrier protein]	C12H21O2SR	C5756
68	3oxdeACP	3-Oxodecanoyl-[acyl-carrier protein]	C10H17O2SR	C05753
69	3oxhACP	3-Oxohexanoyl-[acyl-carrier protein]	C6H9O2SR	C05746
70	3oxhdACP	3-Oxohexadecanoyl-[acp]	C16H29O2SR	C05762
71	3oxicsacp	3-Oxoicosanoyl [acyl-carrier protein]	C20H37O2X	0
72	3oxocACP	3-Oxooctanoyl-[acyl-carrier protein]	C8H13O2SR	C05750
73	3oxocdacp	3-Oxooctadecanoyl-[acp]	C18H33O2X	0
74	3oxtdACP	3-Oxotetradecanoyl-[acyl-carrier protein]	C14H25O2SR	C05759
75	3pg	3-Phospho-D-glycerate	C3H4O7P	C00197
76	3php	3-Phosphohydroxypyruvate	C3H2O7P	C03232
77	3psme	5-O-(1-Carboxyvinyl)-3-phosphoshikimate	C10H9O10P	C01269

No	Abbreviation	Name	Formula	KEGG ID
78	4abut	4-Aminobutanoate	C4H9NO2	C00334
79	4abz	4-Aminobenzoate	C7H6NO2	C00568
80	4adcho	4-amino-4-deoxychorismate	C10H10NO5	C11355
81	4ahmmp	4-Amino-5-hydroxymethyl-2-methylpyrimidine	C6H9N3O	C01279
82	4ampm	4-Amino-2-methyl-5-phosphomethylpyrimidine	C6H8N3O4P	C04556
83	4c2me	4-(cytidine 5'-diphospho)-2-C-methyl-D-erythritol	C14H23N3O14P2	C11435
84	4hba	4-Hydroxy-benzyl alcohol	C7H8O2	0
85	4hphaccoa	4-Hydroxyphenylacetyl-CoA	C29H42N7O18P3S	C05338
86	4mop	4-Methyl-2-oxopentanoate	C6H9O3	C00233
87	4mpetz	4-Methyl-5-(2-phosphoethyl)-thiazole	C6H8NO4PS	C04327
88	4pasp	4-Phospho-L-aspartate	C4H6NO7P	C03082
89	4per	4-Phospho-D-erythronate	C4H6O8P	C03393
90	4ppan	D-4'-Phosphopantothenate	C9H15NO8P	C03492
91	4ppcys	N-((R)-4-Phosphopantothenoyl)-L-cysteine	C12H20N2O9PS	C04352
92	4r5au	4-(1-D-Ribitylamino)-5-aminouracil	C9H16N4O6	C04732
93	5aizc	5-amino-1-(5-phospho-D-ribosyl)imidazole-4-carboxylate	C9H11N3O9P	C04751
94	5aprbu	5-Amino-6-(5'-phosphoribitylamino)uracil	C9H15N4O9P	C04454
95	5apru	5-Amino-6-(5'-phosphoribosylamino)uracil	C9H13N4O9P	C01268

No	Abbreviation	Name	Formula	KEGG ID
96	5mdr1p	5-Methylthio-5-deoxy-D-ribose 1-phosphate	C6H11O7PS	C04188
97	5mdru1p	5-Methylthio-5-deoxy-D-ribulose 1-phosphate	C6H11O7PS	C04582
98	5mta	5-Methylthioadenosine	C11H15N5O3S	C00170
99	5mthf	5-Methyltetrahydrofolate	C20H24N7O6	C00440
100	5odhf2a	5-Oxo-4,5-dihydrofuran-2-acetate	C6H6O4	C03586
101	5prdmbz	N1-(5-Phospho-alpha-D-ribosyl)-5,6-dimethylbenzimidazole	C14H17N2O7P	C04778
102	6pthp	6-Pyruvoyl-5,6,7,8-tetrahydropterin	C9H11N5O3	C03684
103	8aonn	8-Amino-7-oxononanoate	C9H17NO3	C01092
104	ac	Acetate	C2H3O2	C00033
105	ac	Acetate	C2H3O2	C00033
106	acACP	Acetyl-ACP	C13H23N2O8PRS	C03939
107	acald	Acetaldehyde	C2H4O	C00084
108	accoa	Acetyl-CoA	C23H34N7O17P3S	C00024
109	acg5p	N-Acetyl-L-glutamyl 5-phosphate	C7H9NO8P	C04133
110	acg5sa	N-Acetyl-L-glutamate 5-semialdehyde	C7H10NO4	C01250
111	acgam1p	N-Acetyl-D-glucosamine 1-phosphate	C8H14NO9P	C04256
112	acglu	N-Acetyl-L-glutamate	C7H9NO5	C00624
113	achms	O-Acetyl-L-homoserine	C6H11NO4	C01077

No	Abbreviation	Name	Formula	KEGG ID
114	acorn	N2-Acetyl-L-ornithine	C7H14N2O3	C00437
115	ACP	acyl carrier protein	C11H21N2O7PRS	C00229
116	acser	O-Acetyl-L-serine	C5H9NO4	C00979
117	actACP	Acetoacetyl-ACP	C15H25N2O9PRS	C05744
118	adcoba	Adenosyl cobinamide	C58H84CoN16O11	C06508
119	adcobap	Adenosyl cobinamide phosphate	C58H85CoN16O14P	C06509
120	adcobdam	Adenosyl cobyrinate diamide	C55H73CoN11O15	C06506
121	adcobhex	adenosyl-cobyric acid	C55H77CoN15O11	C06507
122	ade	Adenine	C5H5N5	C00147
123	adgcoba	Adenosine-GDP-cobinamide	C68H97CoN21O21P2	C06510
124	adn	Adenosine	C10H13N5O4	C00212
125	adocbl	Adenosylcobalamin	C72H100CoN18O17P	C00194
126	adp	ADP	C10H12N5O10P2	C00008
127	adpglc	ADPglucose	C16H23N5O15P2	C00498
128	adprib	ADPribose	C15H21N5O14P2	C00301
129	agm	Agmatine	C5H16N4	C00179
130	ahcys	S-Adenosyl-L-homocysteine	C14H20N6O5S	C00021
131	ahdt	2-Amino-4-hydroxy-6-(erythro-1,2,3-trihydroxypropyl)dihydropteridine triphosphate	C9H12N5O13P3	C04895

No	Abbreviation	Name	Formula	KEGG ID
132	aicar	5-Amino-1-(5-Phospho-D-ribosyl)imidazole-4-carboxamide	C9H13N4O8P	C04677
133	air	5-amino-1-(5-phospho-D-ribosyl)imidazole	C8H12N3O7P	C03373
134	akg	2-Oxoglutarate	C5H4O5	C00026
135	ala-B	beta-Alanine	C3H7NO2	C00099
136	ala-D	D-Alanine	C3H7NO2	C00133
137	ala-L	L-Alanine	C3H7NO2	C00041
138	alaala	D-Alanyl-D-alanine	C6H12N2O3	C00993
139	alac-S	(S)-2-Acetolactate	C5H7O4	C06010
140	alatrna	L-Alanyl-tRNA(Ala)	C3H6NOR	C00886
141	amet	S-Adenosyl-L-methionine	C15H23N6O5S	C00019
142	amob	S-Adenosyl-4-methylthio-2-oxobutanoate	C15H19N5O6S	C04425
143	amp	AMP	C10H12N5O7P	C00020
144	anth	Anthranilate	C7H6NO2	C00108
145	antim	Antimonite	Sb	0
146	antim	Antimonite	Sb	0
147	apoACP	apoprotein [acyl carrier protein]	RHO	0

No	Abbreviation	Name	Formula	KEGG ID
148	aps	Adenosine 5'-phosphosulfate	C10H12N5O10PS	C00224
149	arg-L	L-Arginine	C6H15N4O2	C00062
150	argsuc	N(omega)-(L-Arginino)succinate	C10H17N4O6	C03406
151	argtrna	L-Arginyl-tRNA(Arg)	C6H14N4OR	C02163
152	arsna	Arsenate	AsH3O4	C01478
153	arsna	Arsenate	AsH3O5	C01479
154	arsni2	arsenite	AsH3O3	C06697
155	arsni2	arsenite	AsH3O4	C06698
156	asn-L	L-Asparagine	C4H8N2O3	C00152
157	asp-L	L-Aspartate	C4H6NO4	C00049
158	aspsa	L-Aspartate 4-semialdehyde	C4H7NO3	C00441
159	asptrna	L-Aspartyl-tRNA(Asp)	C4H5NO3R	C02984
160	atp	ATP	C10H12N5O13P3	C00002
161	btamp	Biotinyl-5'-AMP	C20H28N7O9PS	C05921
162	btn	Biotin	C10H15N2O3S	C00120
163	butACP	Butyryl-[acyl-carrier protein]	C15H27N2O8PRS	C05745
164	camp	cAMP	C10H11N5O6P	C00575
165	cbasp	N-Carbamoyl-L-aspartate	C5H6N2O5	C00438
166	cbi	Cobinamide	C48H72CoN11O8	C05774
167	cbl1	Cob(I)alamin	C62H88CoN13O14P	C00853

No	Abbreviation	Name	Formula	KEGG ID
168	cbl1	Cob(I)alamin	C62H88CoN13O14P	C00853
169	cbp	Carbamoyl phosphate	CH2NO5P	C00169
170	ccnt	Citraconate	C5H6O4	C02226
171	cdlp	Cardiolipin	C13H18O17P2R4	C05980
172	cdp	CDP	C9H12N3O11P2	C00112
173	cdpdag	CDPdiacylglycerol	C14H19N3O15P2R2	C00269
174	chor	Chorismate	C10H8O6	C00251
175	cit	Citrate	C6H5O7	C00158
176	cit	Citrate	C6H5O7	C00158
177	citr-L	L-Citrulline	C6H13N3O3	C00327
178	citrmal	(3R)-citramalate	C5H8O5	C02612
179	cl	Chloride	Cl	C00115
180	cmp	CMP	C9H12N3O8P	C00055
181	co	Carbon monoxide	CO	C00237
182	co1dam	Cob(I)yrinate a,c diamide	C45H61CoN6O12	C06505
183	co2	CO2	CO2	C00011
184	co2	CO2	CO2	C00011
185	co2dam	Cob(II)yrinate a,c diamide	C45H61CoN6O12	C06504
186	coa	Coenzyme A	C21H32N7O16P3S	C00010
187	cobalt2	Co2+	Co	C00175
188	cobalt2	Co2+	Co	C00175
189	cobamcoa	Cobamide coenzyme	C72H101CoN18O17P	C00194
190	cobrnt	Cobyrinate	C45H59CoN4O14	C05773
191	codhpre6	Cobalt-dihydro-precorrin 6	C44H55CoN4O16	0
192	copre3	cobalt-precorrin 3	C43H47CoN4O16	C11539
193	copre4	cobalt-precorrin 4	C44H50CoN4O16	C11540

No	Abbreviation	Name	Formula	KEGG ID
194	copre8	Cobalt-precorrin 8	C45H59CoN4O14	C11545
195	cpppg3	Coproporphyrinogen III	C36H40N4O8	C03263
196	ctp	CTP	C9H12N3O14P3	C00063
197	cu2	Cu2+	Cu	C00070
198	cu2	Cu2+	Cu	C00071
199	cys-L	L-Cysteine	C3H7NO2S	C00097
200	cysam	Cysteamine	C2H7NS	C01678
201	cystrna	L-Cysteinyl-tRNA(Cys)	C3H6NOSR	C03125
202	dad-5	5'-Deoxyadenosine	C10H13N5O3	C05198
203	dadp	dADP	C10H12N5O9P2	C00206
204	damp	dAMP	C10H12N5O6P	C00360
205	dann	7,8-Diaminononanoate	C9H21N2O2	C01037
206	datp	dATP	C10H12N5O12P3	C00131
207	db4p	3,4-dihydroxy-2-butanone 4-phosphate	C4H7O6P	0
208	dcamp	N6-(1,2-Dicarboxyethyl)-AMP	C14H14N5O11P	C03794
209	dcb	1,4-Dichlorobenzene (para-Dichlorobenzene)	C6H4Cl2	C07092
210	dcdp	dCDP	C9H12N3O10P2	C00705
211	dcmp	dCMP	C9H12N3O7P	C00239
212	dctp	dCTP	C9H12N3O13P3	C00458
213	ddca	dodecanoate (C12:0)	C12H23O2	C02679
214	ddcaACP	Dodecanoyl-ACP (n-C12:0ACP)	C23H43N2O8PRS	C05223
215	ddcoa	Dodecanoyl-CoA (n-C12:0CoA)	C33H58N7O17P3S	C01832
216	de3mlt	D-erythro-3-Methylmalate	C5H8O5	C06032
217	decACP	Decanoyl-[acyl-carrier protein]	C10H19OSR	C05755

No	Abbreviation	Name	Formula	KEGG ID
218	decdp	all-trans-Decaprenyl diphosphate	C50H84O7P2	C04509
219	dgdp	dGDP	C10H12N5O10P2	C00361
220	dgmp	dGMP	C10H12N5O7P	C00362
221	dgsn	Deoxyguanosine	C10H13N5O4	C00330
222	dgtp	dGTP	C10H12N5O13P3	C00286
223	dhap	Dihydroxyacetone phosphate	C3H5O6P	C00111
224	dhf	7,8-Dihydrofolate	C19H19N7O6	C00415
225	dhlpro	Dihydrolipolprotein	C8H16NOS2R	C02972
226	dhna	1,4-Dihydroxy-2-naphthoate	C11H7O4	C03657
227	dhnpt	2-Amino-4-hydroxy-6-(D-erythro-1,2,3-trihydroxypropyl)-7,8-dihydropteridine	C9H13N5O4	C04874
228	dhor-S	(S)-Dihydroorotate	C5H5N2O4	C00337
229	dhpmp	Dihydroneopterin monophosphate	C9H12N5O7P	C05925
230	dhpt	Dihydropteroate	C14H13N6O3	C00921
231	dimp	dIMP	C10H11N4O7P	C06196
232	ditp	dITP	C10H11N4O13P3	C01345
233	dmbzid	5,6-Dimethylbenzimidazole	C9H10N2	C03114
234	dmlz	6,7-Dimethyl-8-(1-D-ribityl)lumazine	C13H18N4O6	C04332
235	dmpp	Dimethylallyl diphosphate	C5H9O7P2	C00235
236	dnad	Deamino-NAD+	C21H24N6O15P2	C00857
237	dpcoa	Dephospho-CoA	C21H33N7O13P2S	C00882
238	dtbt	Dethiobiotin	C10H17N2O3	C01909
239	dtdp	dTDP	C10H13N2O11P2	C00363

No	Abbreviation	Name	Formula	KEGG ID
240	dtdpglc	dTDPglucose	C16H26N2O16P2	C00842
241	dtmp	dTMP	C10H13N2O8P	C00364
242	dttp	dTTP	C10H13N2O14P3	C00459
243	dudp	dUDP	C9H11N2O11P2	C01346
244	dump	dUMP	C9H11N2O8P	C00365
245	dutp	dUTP	C9H11N2O14P3	C00460
246	dxyl5p	1-deoxy-D-xylulose 5-phosphate	C5H9O7P	C11437
247	e4p	D-Erythrose 4-phosphate	C4H7O7P	C00279
248	eig3p	D-erythro-1-(Imidazol-4-yl)glycerol 3-phosphate	C6H9N2O6P	C04666
249	etl	Ethylene	C2H4	0
250	etoh	Ethanol	C2H6O	C00469
251	f1p	D-Fructose 1-phosphate	C6H11O9P	C02976
252	f6p	D-Fructose 6-phosphate	C6H11O9P	C00085
253	fa11	Fatty acid (Iso-C17:0)	C17H34O2	0
254	fa11coa	Iso-C17:0 CoA	C38H68N7O17P3S1	0
255	fad	FAD	C27H31N9O15P2	C00016
256	fadh2	FADH2	C27H33N9O15P2	C01352
257	fc1p	L-Fuculose 1-phosphate	C6H11O8P	C01099
258	fdox	ferredoxin (oxidized) 2[4Fe-4S]	Fe8S8X	0

No	Abbreviation	Name	Formula	KEGG ID
259	fdp	D-Fructose 1,6-bisphosphate	C6H10O12P2	C00354
260	fdred	ferredoxin (reduced) 2[4Fe-4S]	Fe8S8X	0
261	fe2	Fe2+	Fe	C00023
262	fe2	Fe2+	Fe	C00023
263	fgam	N2-Formyl-N1-(5-phospho-D-ribosyl)glycinamide	C8H13N2O9P	C04376
264	fmet	N-Formyl-L-methionine	C6H11NO3S	C03145
265	fmettrna	N-Formylmethionyl-tRNA	C6H9NO2SR	C03294
266	fmn	flavin mononucleotide	C17H19N4O9P	C00061
267	fol	Folate	C19H19N7O6	C00504
268	for	Formate	CH1O2	C00058
269	fpram	2-(Formamido)-N1-(5-phospho-D-ribosyl)acetamidine	C8H15N3O8P	C04640
270	fprica	5-Formamido-1-(5-phospho-D-ribosyl)imidazole-4-carboxamide	C10H13N4O9P	C04734
271	frdp	Farnesyl diphosphate	C15H25O7P2	C00448
272	fru	D-Fructose	C6H12O6	C00095
273	fum	Fumarate	C4H2O4	C00122
274	g1p	D-Glucose 1-phosphate	C6H11O9P	C00103
275	g3p	Glyceraldehyde 3-phosphate	C3H5O6P	C00661
276	g6p	D-Glucose 6-phosphate	C6H11O9P	C00092
277	gam1p	D-Glucosamine 1-phosphate	C6H13NO8P	C06156

No	Abbreviation	Name	Formula	KEGG ID
278	gam6p	D-Glucosamine 6-phosphate	C6H13NO8P	C00352
279	gar	N1-(5-Phospho-D-ribosyl)glycinamide	C7H14N2O8P	C03838
280	gcald	Glycolaldehyde	C2H4O2	C00266
281	gdp	GDP	C10H12N5O11P2	C00035
282	gdpman	GDP-D-mannose	C16H25N5O16P2	C00096
283	gdptp	Guanosine 3'-diphosphate 5'-triphosphate	C10H11N5O20P5	C04494
284	ggdp	Geranylgeranyl diphosphate	C20H36O7P2	C00353
285	glc-D	D-Glucose	C6H12O6	C00031
286	gln-L	L-Glutamine	C5H10N2O3	C00064
287	gln-L	L-Glutamine	C5H10N2O3	C00064
288	glntrna	L-Glutaminyl-tRNA(Gln)	C5H9N2O2R	C02282
289	glu-L	L-Glutamate	C5H8NO4	C00025
290	glu5p	L-Glutamate 5-phosphate	C5H8NO7P	C03287
291	glu5sa	L-Glutamate 5-semialdehyde	C5H9NO3	C01165
292	glutrna	L-Glutamyl-tRNA(Glu)	C5H7NO3R	C02987
293	gly	Glycine	C2H5NO2	C00037
294	glyald	D-Glyceraldehyde	C3H6O3	C00577
295	glyb	Glycine betaine	C5H11NO2	C00719
296	glyb	Glycine betaine	C5H11NO2	C00719
297	glyc	Glycerol	C3H8O3	C00116
298	glyc-R	(R)-Glycerate	C3H5O4	C00258
299	glyc3p	sn-Glycerol 3-phosphate	C3H7O6P	C00093

No	Abbreviation	Name	Formula	KEGG ID
300	glycogen	glycogen	C6H10O5	C00182
301	glytrna	Glycyl-tRNA(Gly)	C2H4NOR	C02412
302	gmp	GMP	C10H12N5O8P	C00144
303	grdp	Geranyl diphosphate	C10H17O7P2	C00341
304	gsn	Guanosine	C10H13N5O5	C00387
305	gthrd	Reduced glutathione	C10H16N3O6S	C00051
306	gtp	GTP	C10H12N5O14P3	C00044
307	gua	Guanine	C5H5N5O	C00242
308	h	H+	H	C00080
309	h	H+	H	C00080
310	h2	H2	H2	C00282
311	h2	H2	H2	C00282
312	h2mb4p	1-hydroxy-2-methyl-2-(E)-butenyl 4-diphosphate	C5H9O8P2	C11811
313	h2o	H2O	H2O	C00001
314	h2o	H2O	H2O	C00001
315	h2o2	Hydrogen peroxide	H2O2	C00027
316	h2s	Hydrogen sulfide	H2S	C00283
317	hcb	Hexachlorobenzene	C6Cl6	C11042
318	hcit	Homocitrate	C7H10O7	C01251
319	hco3	Bicarbonate	CHO3	C00288
320	hcys-L	L-Homocysteine	C4H9NO2S	C00155
321	hdca	Hexadecanoate (n-C16:0)	C16H31O2	C00249
322	hdeACP	Hexadecenoyl-ACP (n-C16:1ACP)	C27H49N2O8PRS	0
323	hepdp	all-trans-Heptaprenyl diphosphate	C35H60O7P2	C04216
324	hexACP	Hexanoyl-[acyl-carrier protein]	C17H31N2O8PRS	C05749
325	hexdp	all-trans-Hexaprenyl diphosphate	C30H52O7P2	C01230

No	Abbreviation	Name	Formula	KEGG ID
326	hg0	Mercury (uncharged)	Hg	C01319
327	hg2	Mercury (charged +2)	Hg	C00703
328	his-L	L-Histidine	C6H9N3O2	C00135
329	hisp	L-Histidinol phosphate	C6H11N3O4P	C01100
330	histd	L-Histidinol	C6H12N3O	C00860
331	histrna	L-Histidyl-tRNA(His)	C6H8N3OR	C02988
332	hmbil	Hydroxymethylbilane	C40H38N4O17	C01024
333	hom-L	L-Homoserine	C4H9NO3	C00263
334	hspmd	Homospermidine	C8H21N3	C06366
335	hxan	Hypoxanthine	C5H4N4O	C00262
336	iasp	Iminoaspartate	C4H3NO4	C05840
337	ibcoa	Isobutyryl-CoA	C25H42N7O17P3S	C00630
338	icit	Isocitrate	C6H5O7	C00311
339	icsa	Icosanoate (n-C20:0)	C20H40O2	C06425
340	icsACP	Icosanoyl-ACP (n-C20:0ACP)	C20H39OX	0
341	icscoa	Icosanoyl-CoA (n-C20:0-CoA)	C41H74N7O17P3S	C16180
342	idp	IDP	C10H11N4O11P2	C00104
343	ile-L	L-Isoleucine	C6H13NO2	C00407
344	ile-L	L-Isoleucine	C6H13NO2	C00407
345	iletrna	L-Isoleucyl-tRNA(Ile)	C6H12NOR	C03127
346	imacp	3-(Imidazol-4-yl)-2-oxopropyl phosphate	C6H7N2O5P	C01267
347	imp	IMP	C10H11N4O8P	C00130
348	indaccoa	S-2-(indol-3-yl)acetyl-CoA	C31H43N8O17P3S	0

No	Abbreviation	Name	Formula	KEGG ID
349	indpyr	Indolepyruvate	C11H9NO3	C00331
350	ipdp	Isopentenyl diphosphate	C5H9O7P2	C00129
351	itp	ITP	C10H11N4O14P3	C00081
352	ivcoa	Isovaleryl-CoA	C26H44N7O17P3S	C02939
353	k	K+	K	C00238
354	k	K+	K	C00238
355	lac-D	D-Lactate	C3H5O3	C00256
356	lac-L	L-Lactate	C3H5O3	C00186
357	lald-L	L-Lactaldehyde	C3H6O2	C00424
358	leu-L	L-Leucine	C6H13NO2	C00123
359	leu-L	L-Leucine	C6H13NO2	C00123
360	leutrna	L-Leucyl-tRNA(Leu)	C6H12NOR	C02047
361	lgt-S	(R)-S-Lactoylglutathione	C13H20N3O8S	C03451
362	lipidAds	Lipid A Disaccharide	C68H127N2O20P	C04932
363	lipidX	2,3-Bis(3-hydroxytetradecanoyl)-beta-D-glucosaminyl 1-phosphate	C34H64NO12P	C04824
364	lpro	Lipoylprotein	C8H14NOS2R	C02051
365	Lsacchrp	L-Saccharopine	C11H20N2O6	C00449
366	lys-L	L-Lysine	C6H15N2O2	C00047
367	lystrna	L-Lysine-tRNA (Lys)	C6H14N2OR	C01931
368	mal-L	L-Malate	C4H4O5	C00149
369	malACP	Malonyl-[acyl-carrier protein]	C14H22N2O10PRS	C01209
370	malcoa	Malonyl-CoA	C24H33N7O19P3S	C00083
371	man1p	D-Mannose 1-phosphate	C6H11O9P	C03812
372	man6p	D-Mannose 6-phosphate	C6H11O9P	C00275

No	Abbreviation	Name	Formula	KEGG ID
373	met-L	L-Methionine	C5H11NO2S	C00073
374	methf	5,10-Methenyltetrahydrofolate	C20H20N7O6	C00445
375	metox	L-Methionine S-oxide	C5H11NO3S	C02989
376	mettrna	L-Methionyl-tRNA (Met)	C5H10NOSR	C02430
377	mg2	Mg	Mg	C00305
378	mg2	Mg	Mg	C00305
379	micit	Methylisocitrate	C7H7O7	C04593
380	mlthf	5,10-Methylenetetrahydrofolate	C20H21N7O6	C00143
381	mmcoa-S	(S)-Methylmalonyl-CoA	C25H35N7O19P3S	C00683
382	mn2	Mn2+	Mn	C00034
383	mn2	Mn2+	Mn	C00034
384	mobd	Molybdate	MoO4	C06232
385	mobd	Molybdate	MoO4	C06232
386	mql7	Menaquinol 7	C46H66O2	0
387	mql8	Menaquinol 8	C51H74O2	C05819
388	mqn7	Menaquinone 7	C46H64O2	0
389	mqn8	Menaquinone 8	C51H72O2	C00828
390	mthgxl	Methylglyoxal	C3H4O2	C00546
391	myrsACP	Myristoyl-ACP (n-C14:0ACP)	C25H47N2O8PRS	C05761
392	nac	Nicotinate	C6H4NO2	C00253
393	nad	Nicotinamide adenine dinucleotide	C21H26N7O14P2	C00003
394	nadh	Nicotinamide adenine dinucleotide - reduced	C21H27N7O14P2	C00004
395	nadp	Nicotinamide adenine dinucleotide phosphate	C21H25N7O17P3	C00006
396	nadph	Nicotinamide adenine dinucleotide phosphate - reduced	C21H26N7O17P3	C00005

No	Abbreviation	Name	Formula	KEGG ID
397	nh4	Ammonium	H4N	C01342
398	nh4	Ammonium	H4N	C01342
399	nh4oh	Ammonium hydroxide	H5NO	C01358
400	ni2	Ni2+	Ni	C00291
401	ni2	Ni2+	Ni	C00291
402	nicrnt	Nicotinate D-ribonucleotide	C11H12NO9P	C01185
403	nmn	NMN	C11H14N2O8P	C00455
404	no2	Nitrite	NO2	C00088
405	nondp	all-trans-Nonaprenyl diphosphate	C45H76O7P2	C04145
406	o2	O2	O2	C00007
407	o2-	Superoxide	O2	C00704
408	oaa	Oxaloacetate	C4H2O5	C00036
409	ocdACP	Octadecanoyl-ACP (n-C18:0ACP)	C18H35OX	0
410	ocdca	octadecanoate (n-C18:0)	C18H35O2	C01530
411	ocdcea	octadecenoate (n-C18:1)	C18H33O2	0
412	octACP	Octanoyl-[acyl-carrier protein]	C8H15OSR	C05752
413	octdp	all-trans-Octaprenyl diphosphate	C40H65O7P2	C04146
414	odecoa	Octadecenoyl-CoA (n-C18:1CoA)	C39H64N7O17P3S	0
415	orn-L	L-Ornithine	C5H12N2O2	C01602
416	orot	Orotate	C5H3N2O4	C00295
417	orot5p	Orotidine 5'-phosphate	C10H10N2O11P	C01103
418	palmACP	Palmitoyl-ACP (n-C16:0ACP)	C27H51N2O8PRS	C05764
419	pan4p	Pantetheine 4'-phosphate	C11H21N2O7PS	C01134
420	pant-R	(R)-Pantoate	C6H11O4	C00522
421	pap	Adenosine 3',5'-bisphosphate	C10H11N5O10P2	C00054

No	Abbreviation	Name	Formula	KEGG ID
422	pce	Tetrachloroethylene	C2Cl4	C06789
423	pdx5p	Pyridoxine 5'-phosphate	C8H10NO6P	C00627
424	pendp	all-trans-Pentaprenyl diphosphate	C25H44O7P2	C04217
425	pep	Phosphoenolpyruvate	C3H2O6P	C00074
426	pgly	Phosphatidylglycerol	C8H13O10PR2	C00344
427	pglyp	Phosphatidylglycerophosphate	C8H14O13P2R2	C03892
428	phaccoa	Phenylacetyl-CoA	C29H38N7O17P3S	C00582
429	phe-L	L-Phenylalanine	C9H11NO2	C00079
430	pheac	Phenylacetate	C8H8O2	C07086
431	phetrna	L-Phenylalanyl-tRNA(Phe)	C9H10NOR	C03511
432	phom	O-Phospho-L-homoserine	C4H8NO6P	C01102
433	phpyr	Phenylpyruvate	C9H7O3	C00166
434	phthr	O-Phospho-4-hydroxy-L-threonine	C4H8NO7P	C06055
435	pi	Phosphate	HO4P	C00009
436	pi	Phosphate	HO4P	C00009
437	pmtcoa	Palmitoyl-CoA (n-C16:0CoA)	C37H62N7O17P3S	C00154
438	pnto-R	(R)-Pantothenate	C9H16NO5	C00864
439	ppal	Propanal	C3H6O	C00479
440	ppcoa	Propanoyl-CoA	C24H36N7O17P3S	C00100
441	pphn	Prephenate	C10H8O6	C00254
442	ppi	Diphosphate	HO7P2	C00013
443	ppoh	Propanol	C3H8O	C05979
444	pppg9	Protoporphyrinogen IX	C34H38N4O4	C01079

No	Abbreviation	Name	Formula	KEGG ID
445	pppi	Inorganic triphosphate	HO10P3	C03279
446	pram	5-Phospho-beta-D-ribosylamine	C5H11NO7P	C03090
447	pran	N-(5-Phospho-D-ribosyl)anthranilate	C12H13NO9P	C04302
448	prbamp	1-(5-Phosphoribosyl)-AMP	C15H19N5O14P2	C02741
449	prbatp	1-(5-Phosphoribosyl)-ATP	C15H19N5O20P4	C02739
450	prfp	1-(5-Phosphoribosyl)-5-[(5-phosphoribosylamino)methylideneamino]imidazole-4-carboxamide	C15H21N5O15P2	C04896
451	prlp	5-[(5-phospho-1-deoxyribulos-1-ylamino)methylideneamino]-1-(5-phosphoribosyl)imidazole-4-carboxamide	C15H21N5O15P2	C04916
452	pro-L	L-Proline	C5H9NO2	C00148
453	pro-L	L-Proline	C5H9NO2	C00148
454	protrna	L-Prolyl-tRNA(Pro)	C5H8NOR	C02702
455	prpp	5-Phospho-alpha-D-ribose 1-diphosphate	C5H8O14P3	C00119
456	ps	Phosphatidylserine	C8H12NO10PR2	C02737
457	psd5p	Pseudouridine 5'-phosphate	C9H13N2O9P	C01168
458	pser-L	O-Phospho-L-serine	C3H6NO6P	C01005
459	ptrc	Putrescine	C4H14N2	C00134
460	ptth	Pantetheine	C11H22N2O4S	C00831
461	pyam5p	Pyridoxamine 5'-phosphate	C8H12N2O5P	C00647
462	pydx5p	Pyridoxal 5'-phosphate	C8H8NO6P	C00018
463	pyr	Pyruvate	C3H3O3	C00022
464	quln	Quinolinate	C7H3NO4	C03722
465	r5p	alpha-D-Ribose 5-phosphate	C5H9O8P	C00117

No	Abbreviation	Name	Formula	KEGG ID
466	rdmbzi	N1-(alpha-D-ribosyl)-5,6-dimethylbenzimidazole	C14H18N2O4	C05775
467	rib-D	D-Ribose	C5H10O5	C00121
468	ribflv	Riboflavin	C17H20N4O6	C00255
469	ru5p-D	D-Ribulose 5-phosphate	C5H9O8P	C00199
470	s	Sulfur	S	C00087
471	s7p	Sedoheptulose 7-phosphate	C7H13O10P	C00281
472	ser-L	L-Serine	C3H7NO3	C00065
473	sertrna	L-Seryl-tRNA(Ser)	C3H6NO2R	C02553
474	shcl	dihydrosirohydrochlorin	C42H48N4O16	C02463
475	skm	Shikimate	C7H9O5	C00493
476	skm5p	Shikimate 5-phosphate	C7H8O8P	C03175
477	sl26da	N-Succinyl-LL-2,6-diaminoheptanedioate	C11H16N2O7	C04421
478	sl2a6o	N-Succinyl-2-L-amino-6-oxoheptanedioate	C11H12NO8	C04462
479	so3	Sulfite	O3S	C00094
480	so4	Sulfate	O4S	C00059
481	so4	Sulfate	O4S	C00059
482	strcoa	Stearyl-CoA (n-C18:0CoA)	C39H70N7O17P3S	C00412
483	succ	Succinate	C4H4O4	C00042
484	succoa	Succinyl-CoA	C25H35N7O19P3S	C00091
485	sucsal	Succinic semialdehyde	C4H5O3	C00232
486	tag6p-D	D-Tagatose 6-phosphate	C6H11O9P	C01097
487	tagdp-D	D-Tagatose 1,6-biphosphate	C6H10O12P2	C03785
488	tdcoa	Tetradecanoyl-CoA (n-C14:0CoA)	C35H58N7O17P3S	C02593
489	tdeACP	Tetradecenoyl-ACP (n-C14:1ACP)	C25H45N2O8PRS	0
490	thdp	2,3,4,5-Tetrahydrodipicolinate	C7H7NO4	C03972
491	thf	5,6,7,8-Tetrahydrofolate	C19H21N7O6	C00101

No	Abbreviation	Name	Formula	KEGG ID
492	thmmp	Thiamin monophosphate	C12H16N4O4PS	C01081
493	thmpp	Thiamine diphosphate	C12H16N4O7P2S	C00068
494	thr-L	L-Threonine	C4H9NO3	C00188
495	thr-LA	L-Allo-threonine	C4H9NO3	C05519
496	thrp	L-Threonine O-3-phosphate	C4H8NO6P	C12147
497	thrtrna	L-Threonyl-tRNA(Thr)	C4H8NO2R	C02992
498	thymd	Thymidine	C10H14N2O5	C00214
499	trdox	Oxidized thioredoxin	X	C00343
500	trdrd	Reduced thioredoxin	XH2	C00342
501	trnaala	tRNA(Ala)	R	C01635
502	trnaarg	tRNA(Arg)	R	C01636
503	trnaasp	tRNA(Asp)	R	C01638
504	trnacys	tRNA(Cys)	R	C01639
505	trnagln	tRNA(Gln)	R	C01640
506	trnaglu	tRNA (Glu)	R	C01641
507	trnagly	tRNA(Gly)	R	C01642
508	trnahis	tRNA(His)	R	C01643
509	trnaile	tRNA(Ile)	R	C01644
510	trnaleu	tRNA(Leu)	R	C01645
511	trnalys	tRNA(Lys)	R	C01646
512	trnamet	tRNA(Met)	R	C01647
513	trnaphe	tRNA(Phe)	R	C01648
514	trnapro	tRNA(Pro)	R	C01649
515	trnaser	tRNA(Ser)	R	C01650

No	Abbreviation	Name	Formula	KEGG ID
516	trnathr	tRNA(Thr)	R	C01651
517	trnatrp	tRNA(Trp)	R	C01652
518	trnatyr	tRNA(Tyr)	R	C00787
519	trnaval	tRNA(Val)	R	C01653
520	trp-L	L-Tryptophan	C11H12N2O2	C00078
521	trptrna	L-Tryptophanyl-tRNA(Trp)	C11H11N2OR	C03512
522	tsul	Thiosulfate	O3S2	C00320
523	tsul	Thiosulfate	O3S2	C00320
524	ttdca	tetradecanoate (C14:0)	C14H27O2	C06424
525	tyr-L	L-Tyrosine	C9H11NO3	C00082
526	tyrtrna	L-Tyrosyl-tRNA(Tyr)	C9H10NO2R	C02839
527	u23ga	UDP-2,3-bis(3-hydroxytetradecanoyl)glucosamine	C43H75N3O20P2	C04652
528	uaccg	UDP-N-acetyl-3-O-(1-carboxyvinyl)-D-glucosamine	C20H26N3O19P2	C04631
529	uacgam	UDP-N-acetyl-D-glucosamine	C17H25N3O17P2	C00043
530	uamr	UDP-N-acetylmuramate	C20H28N3O19P2	C01050
531	ubq8	Ubiquinone-8	C49H74O4	C00399
532	ubq8h2	Ubiquinol-8	C49H76O4	C00399
533	udcpdp	Undecaprenyl diphosphate	C55H89O7P2	C03543
534	udp	UDP	C9H11N2O12P2	C00015
535	udpg	UDPglucose	C15H22N2O17P2	C00029

No	Abbreviation	Name	Formula	KEGG ID
536	udpgal	UDPgalactose	C15H22N2O17P2	C00052
537	ump	UMP	C9H11N2O9P	C00105
538	uppg3	Uroporphyrinogen III	C40H36N4O16	C01051
539	ura	Uracil	C4H4N2O2	C00106
540	urea	Urea	CH4N2O	C00086
541	utp	UTP	C9H11N2O15P3	C00075
542	val-L	L-Valine	C5H11NO2	C00183
543	val-L	L-Valine	C5H11NO2	C00183
544	valtrna	L-Valyl-tRNA(Val)	C5H10NOR	C02554
545	xmp	Xanthosine 5'-phosphate	C10H11N4O9P	C00655
546	xtp	XTP	C10H11N4O15P3	C00700
547	xu5p-D	D-Xylulose 5-phosphate	C5H9O8P	C00231
548	zn2	Zinc	Zn	C00038
549	zn2	Zinc	Zn	C00038

Table 13. Core Reductive Dehalogenase Homologous (*rdh*) Genes of *i* AI549

No	Annotation	CBDB1 gene locus	195 gene locus	BAV1 gene locus	VS gene locus
1	putative reductive dehalogenase	cbdbA187	DET0180	DehaBAV1_0173	VS169
2	putative reductive dehalogenase anchoring protein	cbdbA188	DET0181	DehaBAV1_0172	VS170
3	putative reductive dehalogenase	cbdbA238	DET0302	DehaBAV1_0121	VS1353
4	putative reductive dehalogenase anchoring protein	cbdbA239	DET0303	DehaBAV1_0120	VS1352
5	putative reductive dehalogenase	cbdbA1092	DET1171	DehaBAV1_0988	VS1360
6	putative reductive dehalogenase anchoring protein	cbdbA1094	DET1170	DehaBAV1_0989	VS1359
7	putative reductive dehalogenase	cbdbA96	DET0318	DehaBAV1_0112	VS1387
8	putative reductive dehalogenase	cbdbA243	DET0235	DehaBAV1_0119	VS1347
9	putative reductive dehalogenase anchoring protein	cbdbA1637	DET1544	DehaBAV1_0282	VS1435

Table 14. Dispensable Reductive Dehalogenase Homologous (*rdh*) Genes of *i* AI549

No	Annotation	CBDB1 gene locus	195 gene locus	BAV1 gene locus	VS gene locus
1	putative reductive dehalogenase	cbdbA1453	DET1535	No gene	VS1427
2	putative reductive dehalogenase anchoring protein	cbdbA1454	No gene	No gene	VS1262
3	reductive dehalogenase anchoring protein, putative	cbdbA1490	DET1537	No gene	VS1429
4	trichloroethene reductive dehalogenase	cbdbA1491	DET1538	No gene	VS1430
5	reductive dehalogenase anchoring protein, putative	cbdbB33	DET0307	DehaBAV1_0283	No gene
6	trichloroethene reductive dehalogenase	cbdbA1495	DET0306	No gene	No gene
7	putative reductive dehalogenase anchoring protein	cbdbA1545	No gene	No gene	VS1363
8	reductive dehalogenase	cbdbA1546	DET1519	No gene	VS1364
9	putative reductive dehalogenase anchoring protein	cbdbA1549	DET1521	No gene	VS1377
10	putative reductive dehalogenase	cbdbA1550	DET1522	No gene	VS1378
11	putative reductive dehalogenase anchoring protein	cbdbA1569	DET1521	No gene	VS1377

No	Annotation	CBDB1 gene locus	195 gene locus	BAV1 gene locus	VS gene locus
12	putative reductive dehalogenase	cbdbA1570	DET1522	No gene	VS1378
13	putative reductive dehalogenase anchoring protein	cbdbA1573	DET1518	No gene	VS1374
14	putative reductive dehalogenase	cbdbA1575	DET1519	No gene	VS1375
15	putative reductive dehalogenase anchoring protein	cbdbA1581	No gene	No gene	VS1386
16	reductive dehalogenase homologous protein	cbdbA1582	No gene	DehaBAV1_0112	VS1387
17	putative reductive dehalogenase anchoring protein	cbdbA1587	DET0319	No gene	VS1392
18	putative reductive dehalogenase	cbdbA1588	DET0318	No gene	VS1393
19	putative reductive dehalogenase anchoring protein	cbdbA1594	DET1534	No gene	VS1315
20	reductive dehalogenase	cbdbA1595	DET1535	No gene	VS1316
21	putative reductive dehalogenase anchoring protein	cbdbA1597	No gene	No gene	VS1401
22	putative reductive dehalogenase	cbdbA1598	No gene	No gene	VS1402
23	putative reductive dehalogenase anchoring protein	cbdbA1617	No gene	No gene	VS1420
24	putative reductive dehalogenase	cbdbA1618	No gene	No gene	VS1421

No	Annotation	CBDB1 gene locus	195 gene locus	BAV1 gene locus	VS gene locus
25	putative reductive dehalogenase anchoring protein	cbdbA1623	No gene	DehaBAV1_0111	VS1426
26	putative reductive dehalogenase	cbdbA1624	DET1535	No gene	VS1427
27	putative reductive dehalogenase anchoring protein	cbdbA1626	DET1537	No gene	VS1429
28	putative reductive dehalogenase	cbdbA1627	DET1538	No gene	VS1430
29	hypothetical protein	No gene	No gene	DehaBAV1_0277	VS103
30	reductive dehalogenase	No gene	No gene	DehaBAV1_0276	VS104
31	reductive dehalogenase homologous protein	cbdbA80	DET1559	No gene	No gene
32	putative reductive dehalogenase	cbdbA84	No gene	No gene	VS1342
33	putative reductive dehalogenase	cbdbA88	DET0311	DehaBAV1_0104	No gene
34	putative reductive dehalogenase	cbdbA1455	No gene	No gene	VS1263
35	putative reductive dehalogenase anchoring protein	cbdbA1507	DET0319	No gene	VS1392
36	putative reductive dehalogenase anchoring protein	cbdbA1536	No gene	No gene	VS1386
37	putative reductive dehalogenase	cbdbA1539	No gene	DehaBAV1_0173	No gene
38	reductive dehalogenase anchoring protein, putative	cbdbA1541	No gene	DehaBAV1_0297	VS87

No	Annotation	CBDB1 gene locus	195 gene locus	BAV1 gene locus	VS gene locus
39	putative reductive dehalogenase	cbdbA1578	No gene	No gene	VS1383
40	reductive dehalogenase homologous protein	cbdbA1638	DET1545	No gene	VS1436
41	trichloroethene reductive dehalogenase	No gene	DET0079	DehaBAV1_0847	VS1291
42	reductive dehalogenase anchoring protein, putative	No gene	DET0312	DehaBAV1_0103	No gene
43	reductive dehalogenase	No gene	No gene	DehaBAV1_0281	VS99
44	reductive dehalogenase	No gene	No gene	DehaBAV1_0284	VS96
45	reductive dehalogenase	No gene	No gene	DehaBAV1_0296	VS88

Table 15. Unique Reductive Dehalogenase Homologous (*rdh*) Genes of *i* AI549

No	Annotation	CBDB1 gene locus	195 gene locus	BAV1 gene locus	VS gene locus
1	putative reductive dehalogenase anchoring protein	cbdbA1502	No gene	No gene	No gene
2	putative reductive dehalogenase	cbdbA1503	No gene	No gene	No gene
3	putative reductive dehalogenase anchoring protein	cbdbA1559	No gene	No gene	No gene
4	putative reductive dehalogenase	cbdbA1560	No gene	No gene	No gene
5	putative reductive dehalogenase anchoring protein	cbdbA1562	No gene	No gene	No gene
6	putative reductive dehalogenase	cbdbA1563	No gene	No gene	No gene
7	reductive dehalogenase	No gene	No gene	No gene	VS1340
8	putative reductive dehalogenase anchoring protein	No gene	No gene	No gene	VS1341
9	putative reductive dehalogenase anchoring protein	No gene	No gene	No gene	VS1319
10	reductive dehalogenase	No gene	No gene	No gene	VS1320
11	putative reductive dehalogenase anchoring protein	No gene	No gene	No gene	VS81
12	reductive dehalogenase	No gene	No gene	No gene	VS82
13	putative reductive dehalogenase anchoring protein	cbdbA85	No gene	No gene	No gene

No	Annotation	CBDB1 gene locus	195 gene locus	BAV1 gene locus	VS gene locus
14	putative reductive dehalogenase anchoring protein	cbdbA1452	No gene	No gene	No gene
15	putative reductive dehalogenase	cbdbA1508	No gene	No gene	No gene
16	putative reductive dehalogenase	cbdbA1535	No gene	No gene	No gene
17	reductive dehalogenase-homologous N-terminal fragment	cbdbA1540	No gene	No gene	No gene
18	putative reductive dehalogenase	cbdbA1542	No gene	No gene	No gene
19	putative reductive dehalogenase anchoring protein	cbdbA1577	No gene	No gene	No gene
20	reductive dehalogenase domain protein	No gene	DET0088	No gene	No gene
21	reductive dehalogenase, putative	No gene	DET0173	No gene	No gene
22	trichloroethene reductive dehalogenase anchoring protein, putative	No gene	DET0078	No gene	No gene
23	reductive dehalogenase, putative	No gene	DET0876	No gene	No gene
24	reductive dehalogenase, putative	No gene	DET1528	No gene	No gene
25	reductive dehalogenase anchoring protein, putative	No gene	DET0163	No gene	No gene

No	Annotation	CBDB1 gene locus	195 gene locus	BAV1 gene locus	VS gene locus
26	reductive dehalogenase anchoring protein, putative	No gene	DET0175	No gene	No gene
27	reductive dehalogenase anchoring protein, putative	No gene	DET0236	No gene	No gene
28	reductive dehalogenase anchoring protein, putative	No gene	DET0875	No gene	No gene
29	reductive dehalogenase anchoring protein, putative	No gene	DET1527	No gene	No gene
30	reductive dehalogenase anchoring protein, putative	No gene	DET1558	No gene	No gene
31	hypothetical protein	No gene	No gene	DehaBAV1_0118	No gene
32	reductive dehalogenase	No gene	No gene	No gene	VS1260
33	putative reductive dehalogenase	No gene	No gene	No gene	VS1261
34	reductive dehalogenase	No gene	No gene	No gene	VS1349
35	reductive dehalogenase	No gene	No gene	No gene	VS1344
36	reductive dehalogenase	No gene	No gene	No gene	VS1329
37	reductive dehalogenase	No gene	No gene	No gene	VS1327
38	reductive dehalogenase	No gene	No gene	No gene	VS1314

No	Annotation	CBDB1 gene locus	195 gene locus	BAV1 gene locus	VS gene locus
39	putative reductive dehalogenase anchoring protein	No gene	No gene	No gene	VS1382
40	putative reductive dehalogenase anchoring protein	No gene	No gene	No gene	VS1370
41	putative reductive dehalogenase anchoring protein	No gene	No gene	No gene	VS1339
42	putative reductive dehalogenase anchoring protein	No gene	No gene	No gene	VS1334
43	putative reductive dehalogenase anchoring protein	No gene	No gene	No gene	VS1328
44	putative reductive dehalogenase anchoring protein	No gene	No gene	No gene	VS1326
45	putative reductive dehalogenase anchoring protein	No gene	No gene	No gene	VS1323
46	putative reductive dehalogenase anchoring protein	No gene	No gene	No gene	VS1313
47	putative reductive dehalogenase anchoring protein	No gene	No gene	No gene	VS1307

Table 16. List of Core Hypothetical Genes of *Dehalococcoides* Pan-genome

No	CBDB1 core hypothetical genes	195 core hypothetical genes	BAV1 core hypothetical genes	VS core hypothetical genes	CBDB1 annotation	195 annotation	BAV1 annotation	VS annotation
1	cbdbA2	DET0002	DehaBAV1_0002	VS2	spo0b-associated GTP-binding protein	GTP-binding protein, GTP1/OBG family	small GTP-binding protein	small GTP-binding protein
2	cbdbA10	DET0008	DehaBAV1_0008	VS8	conserved hypothetical membrane protein	hypothetical protein	protein of unknown function DUF205	protein of unknown function DUF205
3	cbdbA13	DET0012	DehaBAV1_0011	VS11	conserved hypothetical protein	hypothetical protein	protein of unknown function DUF951	protein of unknown function DUF951
4	cbdbA15	DET0014	DehaBAV1_0013	VS13	hypothetical protein	hypothetical protein	hypothetical protein	conserved hypothetical protein
5	cbdbA17	DET0015	DehaBAV1_0014	VS14	putative lipoprotein	lipoprotein, putative	hypothetical protein	putative lipoprotein
6	cbdbA19	DET0017	DehaBAV1_0016	VS16	hypothetical protein	hypothetical protein	hypothetical protein	conserved hypothetical protein
7	cbdbA20	DET0018	DehaBAV1_0017	VS17	hypothetical protein	hypothetical protein	hypothetical protein	conserved hypothetical protein
8	cbdbA26	DET0021	DehaBAV1_0020	VS20	conserved hypothetical protein	hypothetical protein	hypothetical protein	hypothetical protein
9	cbdbA25	DET0022	DehaBAV1_0021	VS21	hypothetical protein	hypothetical protein	hypothetical protein	conserved hypothetical protein
10	cbdbA27	DET0023	DehaBAV1_0022	VS22	hypothetical protein	hypothetical protein	hypothetical protein	conserved hypothetical protein
11	cbdbA32	DET0026	DehaBAV1_0025	VS25	putative potassium uptake protein, TrkA homolog	potassium uptake protein, putative	TrkA-N domain protein	TrkA-N domain protein
12	cbdbA33	DET0027	DehaBAV1_0026	VS26	putative potassium uptake protein, fragment	potassium uptake protein, putative	TrkA-N domain protein	TrkA-N domain protein

No	CBDB1 core hypothetical genes	195 core hypothetical genes	BAV1 core hypothetical genes	VS core hypothetical genes	CBDB1 annotation	195 annotation	BAV1 annotation	VS annotation
13	cbdbA35	DET0029	DehaBAV1_0028	VS28	putative Trk system potassium uptake protein	Trk system potassium uptake protein, putative	cation transporter	cation transporter
14	cbdbA37	DET0030	DehaBAV1_0029	VS29	putative potassium uptake protein, TrkA homologe	potassium uptake protein TrkA, putative	TrkA-N domain protein	TrkA-N domain protein
15	cbdbA40	DET0033	DehaBAV1_0030	VS31	conserved hypothetical protein	hypothetical protein	protein of unknown function DUF47	protein of unknown function DUF47
16	cbdbA41	DET0034	DehaBAV1_0031	VS32	putative phosphate transporter	phosphate transporter	phosphate transporter	phosphate transporter
17	cbdbA43	DET0036	DehaBAV1_0033	VS34	conserved hypothetical protein	hypothetical protein	protein of unknown function DUF370	protein of unknown function DUF370
18	cbdbA48	DET0039	DehaBAV1_0036	VS37	conserved domain protein	hypothetical protein	hypothetical protein	hypothetical protein
19	cbdbA50	DET0040	DehaBAV1_0037	VS40	hypothetical protein	hypothetical protein	hypothetical protein	conserved hypothetical protein
20	cbdbA54	DET0043	DehaBAV1_0039	VS42	conserved hypothetical membrane protein	hypothetical protein	hypothetical protein	hypothetical protein
21	cbdbA55	DET0044	DehaBAV1_0040	VS43	conserved hypothetical protein	hypothetical protein	protein of unknown function DUF1025	hypothetical protein
22	cbdbA56	DET0045	DehaBAV1_0041	VS44	redox family protein	redox family protein	C_GCAxxG_C_C family protein	redox family protein
23	cbdbA58	DET0046	DehaBAV1_0042	VS45	putative GTP-binding protein	GTP-binding protein, putative	Protein of unknown function DUF933	Radical SAM domain protein
24	cbdbA62	DET0048	DehaBAV1_0045	VS48	conserved hypothetical membrane protein	hypothetical protein	hypothetical protein	conserved hypothetical protein
25	cbdbA67	DET0051	DehaBAV1_0048	VS51	conserved hypothetical protein	conserved hypothetical protein TIGR00250	Holliday junction resolvase YqgF	Holliday junction resolvase YqgF
26	cbdbA1485	DET0253	DehaBAV1_0074	VS1298	hypothetical protein	DNA primase domain protein	hypothetical protein	conserved hypothetical protein
27	cbdbA87	DET1556	DehaBAV1_0102	VS1356	conserved hypothetical protein	hypothetical protein	protein of unknown function DUF105	protein of unknown function DUF105
28	cbdbA95	DET0238	DehaBAV1_0110	VS1384	conserved hypothetical protein	hypothetical protein	putative ATP binding protein	putative ATP binding protein

No	CBDB1 core hypothetical genes	195 core hypothetical genes	BAV1 core hypothetical genes	VS core hypothetical genes	CBDB1 annotation	195 annotation	BAV1 annotation	VS annotation
29	cbdbA97	DET0308	DehaBAV1_0113	VS1385	conserved hypothetical protein probable peptidase M14, carboxypeptidase A	hypothetical protein	protein of unknown function UPF0153	conserved hypothetical protein
30	cbdbA232	DET0230	DehaBAV1_0125	VS204	hypothetical protein	TM2 domain protein	TM2 domain containing protein+B7201	TM2 domain containing protein+B7201
31	cbdbA229	DET0228	DehaBAV1_0127	VS203	hypothetical protein	hypothetical protein	hypothetical protein	conserved hypothetical protein
32	cbdbA228	DET0226	DehaBAV1_0128	VS202	conserved hypothetical protein	hypothetical protein	HI0933 family protein	HI0933 family protein
33	cbdbA227	DET0225	DehaBAV1_0129	VS201	hypothetical protein	lipoprotein, putative	hypothetical protein	conserved hypothetical protein
34	cbdbA226	DET0224	DehaBAV1_0130	VS200	ABC transporter, ATP-binding protein	ABC transporter, ATP-binding protein	ABC transporter related	ABC transporter related
35	cbdbA225	DET0223	DehaBAV1_0131	VS199	Hly-III family related protein	hemolysin, putative	Hly-III family protein	Hly-III family protein
36	cbdbA215	DET0217	DehaBAV1_0136	VS194	oxidoreductase, aldo reductase	oxidoreductase, aldo/keto reductase family	aldo/keto reductase	aldo/keto reductase
37	cbdbA212	DET0201	DehaBAV1_0152	VS189	probable glycosyltransferase	glycosyl hydrolase domain protein	glycosyl transferase, group 1	glycosyl transferase, group 1
38	cbdbA209	DET0200	DehaBAV1_0153	VS188	hypothetical membrane protein	hypothetical protein	Protein of unknown function DUF1616	Protein of unknown function DUF1616
39	cbdbA207	DET0198	DehaBAV1_0155	VS186	glutaredoxin family protein	glutaredoxin family protein	glutaredoxin	glutaredoxin
40	cbdbA206	DET0197	DehaBAV1_0156	VS185	conserved hypothetical protein	hypothetical protein	hypothetical protein	conserved hypothetical protein
41	cbdbA203	DET0195	DehaBAV1_0158	VS183	conserved hypothetical protein	hypothetical protein	HI0933 family protein	HI0933 family protein
42	cbdbA190	DET0184	DehaBAV1_0168	VS173	membrane protein, TerC family	membrane protein, TerC family	Integral membrane protein TerC	Integral membrane protein TerC
43	cbdbA186	DET0179	DehaBAV1_0174	VS168	conserved hypothetical protein	hypothetical protein	hypothetical protein	conserved hypothetical protein

No	CBDB1 core hypothetical genes	195 core hypothetical genes	BAV1 core hypothetical genes	VS core hypothetical genes	CBDB1 annotation	195 annotation	BAV1 annotation	VS annotation
44	cbdbA180	DET0152	DehaBAV1_0219	VS160	conserved hypothetical membrane protein	hypothetical protein	protein of unknown function DUF6, transmembrane	protein of unknown function DUF6, transmembrane
45	cbdbA178	DET0151	DehaBAV1_0220	VS159	conserved hypothetical protein	hypothetical protein	protein of unknown function DUF45	protein of unknown function DUF45
46	cbdbA176	DET0150	DehaBAV1_0221	VS158	conserved hypothetical protein	hypothetical protein	hypothetical protein	conserved hypothetical protein
47	cbdbA168	DET0143	DehaBAV1_0227	VS151	arsenate reductase	arsenate reductase(EC:1.20.4.1)	protein tyrosine phosphatase	protein tyrosine phosphatase
48	cbdbA156	DET0133	DehaBAV1_0237	VS141	hypothetical protein	hypothetical protein	hypothetical protein	conserved hypothetical protein
49	cbdbA150	DET0129	DehaBAV1_0242	VS136	conserved hypothetical protein	conserved hypothetical protein TIGR00149	protein of unknown function UPF0047	protein of unknown function UPF0047
50	cbdbA142	DET0121	DehaBAV1_0248	VS130	conserved hypothetical protein	hypothetical protein	hypothetical protein	conserved hypothetical protein
51	cbdbA124	DET0105	DehaBAV1_0262	VS116	degV family protein	degV family protein	degV family protein	degV family protein
52	cbdbA116	DET0100	DehaBAV1_0268	VS110	hypothetical membrane protein	hypothetical protein	hypothetical protein	conserved hypothetical membrane protein
53	cbdbA113	DET0098	DehaBAV1_0270	VS108	conserved hypothetical protein	hypothetical protein	hypothetical protein	conserved hypothetical protein
54	cbdbA263	DET0325	DehaBAV1_0304	VS266	patatin-like phospholipase family protein	patatin-like phospholipase family protein	Patatin	Patatin
55	cbdbA264	DET0144	DehaBAV1_0305	VS267	acetyltransferase, GNAT family	acetyltransferase, GNAT family	GCN5-related N-acetyltransferase	GCN5-related N-acetyltransferase
56	cbdbA266	DET0326	DehaBAV1_0306	VS268	conserved hypothetical protein	hypothetical protein	protein of unknown function DUF87	protein of unknown function DUF87
57	cbdbA267	DET0327	DehaBAV1_0307	VS269	conserved hypothetical protein	hypothetical protein	hypothetical protein	conserved hypothetical protein
58	cbdbA268	DET0328	DehaBAV1_0308	VS270	conserved hypothetical protein	hypothetical protein	hypothetical protein	conserved hypothetical protein
59	cbdbA269	DET0329	DehaBAV1_0309	VS271	transporter, LysE family	transporter, LysE family	Lysine exporter protein (LYSE/YGGA)	Lysine exporter protein (LYSE/YGGA)
60	cbdbA270	DET0330	DehaBAV1_0310	VS272	histone deacetylase family protein	histone deacetylase family protein	histone deacetylase superfamily	histone deacetylase superfamily

No	CBDB1 core hypothetical genes	195 core hypothetical genes	BAV1 core hypothetical genes	VS core hypothetical genes	CBDB1 annotation	195 annotation	BAV1 annotation	VS annotation
61	cbdbA280	DET0340	DehaBAV1_0319	VS281	conserved hypothetical protein	MraZ	MraZ protein	MraZ protein
62	cbdbA286	DET0344	DehaBAV1_0323	VS285	conserved hypothetical protein	conserved hypothetical protein TIGR00244	ATP-cone domain protein	ATP-cone domain protein
63	cbdbA289	DET0347	DehaBAV1_0326	VS287	conserved hypothetical protein	hypothetical protein	protein of unknown function DUF88	protein of unknown function DUF88
64	cbdbA290	DET0348	DehaBAV1_0327	VS288	radical SAM domain protein	radical SAM domain protein	Radical SAM domain protein	Radical SAM domain protein
65	cbdbA294	DET0352	DehaBAV1_0330	VS291	conserved hypothetical protein	hypothetical protein	hypothetical protein	conserved hypothetical protein
66	cbdbA296	DET0357	DehaBAV1_0339	VS300	hypothetical protein	hypothetical protein	hypothetical protein	conserved hypothetical protein
67	cbdbA297	DET0358	DehaBAV1_0340	VS301	hypothetical protein	hypothetical protein	hypothetical protein	conserved hypothetical protein
68	cbdbA298	DET0359	DehaBAV1_0341	VS302	conserved hypothetical protein	hypothetical protein	protein of unknown function DUF164	protein of unknown function DUF164
69	cbdbA300	DET0362	DehaBAV1_0343	VS305	hypothetical protein	hypothetical protein	hypothetical protein	conserved hypothetical protein
70	cbdbA303	DET0363	DehaBAV1_0345	VS307	hypothetical protein	hypothetical protein	hypothetical protein	conserved hypothetical protein
71	cbdbA328	DET0381	DehaBAV1_0363	VS325	iron-sulfur cluster-binding protein, Rieske domain	iron-sulfur cluster-binding protein, Rieske family	Rieske (2Fe-2S) domain protein	Rieske (2Fe-2S) domain protein
72	cbdbB11	DET0383	DehaBAV1_0364	VS327	hypothetical protein	hypothetical protein	hypothetical protein	conserved hypothetical protein
73	cbdbA331	DET0386	DehaBAV1_0366	VS329	tetrapyrrole methylase family protein	tetrapyrrole methylase family protein	Uroporphyrin-III C/tetrapyrrole (Corrin/Porphyrin) methyltransferase	Uroporphyrin-III C/tetrapyrrole (Corrin/Porphyrin) methyltransferase
74	cbdbA332	DET0387	DehaBAV1_0367	VS330	hypothetical protein	hypothetical protein	hypothetical protein	conserved hypothetical protein
75	cbdbA346	DET0396	DehaBAV1_0375	VS339	conserved hypothetical protein	conserved hypothetical protein TIGR00150	protein of unknown function UPF0079	protein of unknown function UPF0079
76	cbdbA353	DET0402	DehaBAV1_0381	VS345	SAM-dependent methyltransferase UbiE/COQ5 family	methyltransferase, UbiE/COQ5 family	Methyltransferase type 11	Methyltransferase type 11

No	CBDB1 core hypothetical genes	195 core hypothetical genes	BAV1 core hypothetical genes	VS core hypothetical genes	CBDB1 annotation	195 annotation	BAV1 annotation	VS annotation
77	cbdbA354	DET0403	DehaBAV1_0382	VS346	serine/threonine protein phosphatase family protein	Ser/Thr protein phosphatase family protein	metallophosphoesterase	metallophosphoesterase
78	cbdbA355	DET0404	DehaBAV1_0383	VS347	dimethyladenosine transferase	dimethyladenosine transferase	dimethyladenosine transferase(EC:2.1.1.48)	dimethyladenosine transferase(EC:2.1.1.48)
79	cbdbA358	DET0406	DehaBAV1_0385	VS349	rubrerythrin	rubrerythrin/rubredoxin	Rubrerythrin	Rubrerythrin
80	cbdbA362	DET0410	DehaBAV1_0389	VS353	ABC transporter, ATP-binding protein	nodulation ATP-binding protein I	ABC transporter related	ABC transporter related
81	cbdbA363	DET0411	DehaBAV1_0390	VS354	ABC transporter, permease protein	putative NodJ	ABC-2 type transporter	ABC-2 type transporter
82	cbdbA364	DET0412	DehaBAV1_0391	VS355	conserved hypothetical protein	hypothetical protein	Dephospho-CoA kinase-like protein	conserved hypothetical protein
83	cbdbA372	DET0421	DehaBAV1_0399	VS363	degV family protein	degV family protein	degV family protein	degV family protein
84	cbdbA376	DET0425	DehaBAV1_0401	VS366	conserved hypothetical membrane protein	hypothetical protein	hypothetical protein	conserved hypothetical integral membrane protein
85	cbdbA377	DET0426	DehaBAV1_0402	VS367	conserved hypothetical protein	metallo-beta-lactamase family protein	hypothetical protein	metallo-beta-lactamase family protein
86	cbdbA380	DET0427	DehaBAV1_0404	VS369	carbohydrate kinase, yjeF-family	carbohydrate kinase family protein	carbohydrate kinase, YjeF related protein	carbohydrate kinase, YjeF related protein
87	cbdbA387	DET0433	DehaBAV1_0410	VS375	hypothetical protein	hypothetical protein	hypothetical protein	conserved hypothetical protein
88	cbdbA394	DET0440	DehaBAV1_0417	VS382	hypothetical protein	hypothetical protein	hypothetical protein	conserved hypothetical protein
89	cbdbA410	DET0452	DehaBAV1_0429	VS394	hypothetical protein	hypothetical protein	hypothetical protein	conserved hypothetical protein
90	cbdbA415	DET0455	DehaBAV1_0432	VS397	HIT domain protein	HIT domain protein	histidine triad (HIT) protein	histidine triad (HIT) protein
91	cbdbA420	DET0459	DehaBAV1_0436	VS402	hypothetical membrane protein	hypothetical protein	hypothetical protein	conserved hypothetical protein
92	cbdbA460	DET0496	DehaBAV1_0473	VS438	methionine aminopeptidase, type I	methionine aminopeptidase, type I(EC:3.4.11.18)	methionine aminopeptidase, type I(EC:3.4.11.18)	methionine aminopeptidase, type I(EC:3.4.11.18)

No	CBDB1 core hypothetical genes	195 core hypothetical genes	BAV1 core hypothetical genes	VS core hypothetical genes	CBDB1 annotation	195 annotation	BAV1 annotation	VS annotation
93	cbdbA471	DET0507	DehaBAV1_0484	VS449	hypothetical protein	hypothetical protein	hypothetical protein	conserved hypothetical protein
94	cbdbA475	DET0511	DehaBAV1_0487	VS452	conserved domain protein	hypothetical protein	hypothetical protein	conserved hypothetical protein
95	cbdbA478	DET0514	DehaBAV1_0490	VS455	conserved hypothetical protein	hypothetical protein	Uncharacterized protein-like protein	conserved hypothetical protein
96	cbdbA479	DET0515	DehaBAV1_0491	VS456	conserved hypothetical protein	hypothetical protein	hypothetical protein	conserved hypothetical protein
97	cbdbA481	DET0516	DehaBAV1_0492	VS457	conserved hypothetical protein	hypothetical protein	hypothetical protein	conserved hypothetical protein
98	cbdbA485	DET0519	DehaBAV1_0495	VS460	ABC transporter, permease protein	ABC transporter, permease protein, putative	ABC-2 type transporter	ABC-2 type transporter
99	cbdbA488	DET0521	DehaBAV1_0497	VS462	conserved hypothetical protein	hypothetical protein	hypothetical protein	conserved hypothetical protein
100	cbdbA489	DET0522	DehaBAV1_0498	VS463	conserved hypothetical protein	hypothetical protein	MaoC domain protein dehydratase	MaoC domain protein dehydratase
101	cbdbA493	DET0525	DehaBAV1_0501	VS466	conserved hypothetical protein	hypothetical protein	protein of unknown function DUF75	protein of unknown function DUF75
102	cbdbA504	DET0532	DehaBAV1_0508	VS473	putative phosphatidylethanolamine-binding protein	phosphatidylethanolamine-binding protein, putative	PEBP family protein	PEBP family protein
103	cbdbA505	DET0533	DehaBAV1_0509	VS474	HAD-superfamily hydrolase, subfamily IA	HAD-superfamily hydrolase, subfamily IA	HAD-superfamily hydrolase, subfamily IA, variant 1	HAD-superfamily hydrolase, subfamily IA, variant 1
104	cbdbA510	DET0536	DehaBAV1_0512	VS477	conserved hypothetical protein	hypothetical protein	PSP1 domain protein	PSP1 domain protein
105	cbdbA517	DET0543	DehaBAV1_0519	VS484	fatty acid/phospholipid synthesis protein PlsX	fatty acid/phospholipid synthesis protein	fatty acid/phospholipid synthesis protein PlsX	fatty acid/phospholipid synthesis protein PlsX
106	cbdbA519	DET0544	DehaBAV1_0520	VS485	activator of 2-hydroxyglutaryl-CoA dehydratase	BadF/BadG/BcrA/BcrD ATPase family protein	putative CoA-substrate-specific enzyme activase	putative CoA-substrate-specific enzyme activase
107	cbdbA531	DET0557	DehaBAV1_0531	VS496	hypothetical protein	hypothetical protein	hypothetical protein	hypothetical protein
108	cbdbA541	DET0567	DehaBAV1_0541	VS506	conserved hypothetical protein	hypothetical protein	RNA-binding protein (contains KH domain)-like protein	conserved hypothetical protein

No	CBDB1 core hypothetical genes	195 core hypothetical genes	BAV1 core hypothetical genes	VS core hypothetical genes	CBDB1 annotation	195 annotation	BAV1 annotation	VS annotation
109	cbdbA545	DET0571	DehaBAV1_0545	VS510	hypothetical protein	hypothetical protein	hypothetical protein	conserved hypothetical protein
110	cbdbA549	DET0575	DehaBAV1_0548	VS514	hypothetical protein	hypothetical protein	hypothetical protein	conserved hypothetical protein
111	cbdbA559	DET0581	DehaBAV1_0555	VS520	conserved hypothetical protein	hypothetical protein	DNA alkylation repair enzyme-like protein	conserved hypothetical protein
112	cbdbA562	DET0584	DehaBAV1_0558	VS523	conserved hypothetical protein	hypothetical protein	conserved hypothetical protein 103	conserved hypothetical protein 103
113	cbdbA565	DET0587	DehaBAV1_0561	VS526	conserved hypothetical protein	hypothetical protein	hypothetical protein	conserved hypothetical protein
114	cbdbA567	DET0588	DehaBAV1_0563	VS528	conserved hypothetical protein	hypothetical protein	Uncharacterized protein-like protein	conserved hypothetical protein
115	cbdbA568	DET0589	DehaBAV1_0564	VS529	GTPase domain protein	GTPase domain, tubulin/FtsZ family protein	Tubulin/FtsZ, GTPase	GTPase domain, tubulin/FtsZ family protein
116	cbdbA570	DET0591	DehaBAV1_0566	VS532	conserved hypothetical protein	hypothetical protein	2-hydroxyglutaryl-CoA dehydratase, D-component	2-hydroxyglutaryl-CoA dehydratase, D-component
117	cbdbA574	DET0594	DehaBAV1_0569	VS534	conserved hypothetical protein	conserved hypothetical protein TIGR00725	Rossmann fold nucleotide-binding protein-like protein	conserved hypothetical protein
118	cbdbA578	DET0597	DehaBAV1_0572	VS537	conserved hypothetical protein	hypothetical protein	uncharacterised conserved protein UCP033563	Uncharacterised conserved protein UCP033563
119	cbdbA579	DET0598	DehaBAV1_0573	VS538	SNO glutamine amidotransferase family protein	SNO glutamine amidotransferase family	SNO glutamine amidotransferase	SNO glutamine amidotransferase
120	cbdbA589	DET0607	DehaBAV1_0581	VS546	conserved hypothetical protein	hypothetical protein	protein of unknown function DUF208	protein of unknown function DUF208
121	cbdbA592	DET0609	DehaBAV1_0583	VS548	hypothetical protein	hypothetical protein	hypothetical protein	conserved hypothetical protein
122	cbdbA593	DET0610	DehaBAV1_0584	VS549	conserved hypothetical membrane protein	hypothetical protein	hypothetical protein	conserved hypothetical protein
123	cbdbA594	DET0611	DehaBAV1_0585	VS550	conserved hypothetical protein	hypothetical protein	Methyltransferase type 11	Methyltransferase type 11
124	cbdbA599	DET0617	DehaBAV1_0590	VS555	hypothetical protein	hypothetical protein	hypothetical protein	conserved hypothetical protein

No	CBDB1 core hypothetical genes	195 core hypothetical genes	BAV1 core hypothetical genes	VS core hypothetical genes	CBDB1 annotation	195 annotation	BAV1 annotation	VS annotation
125	cbdbA600	DET0618	DehaBAV1_0591	VS556	putative thiol-disulfide oxidoreductase	thiol-disulfide oxidoreductase, putative	alkyl hydroperoxide reductase/ Thiol specific antioxidant/ Mal allergen	alkyl hydroperoxide reductase/ Thiol specific antioxidant/ Mal allergen
126	cbdbA602	DET0620	DehaBAV1_0593	VS558	conserved domain protein	hypothetical protein	RecB family exonuclease-like protein	conserved hypothetical protein
127	cbdbA604	DET0621	DehaBAV1_0594	VS562	conserved hypothetical membrane protein	hypothetical protein	hypothetical protein	conserved hypothetical protein
128	cbdbA604	DET0621	DehaBAV1_0594	VS562	conserved hypothetical membrane protein	hypothetical protein	hypothetical protein	conserved hypothetical protein
129	cbdbA606	DET0622	DehaBAV1_0595	VS563	radical SAM domain protein	radical SAM domain protein	Radical SAM domain protein	Radical SAM domain protein
130	cbdbA613	DET0626	DehaBAV1_0599	VS567	conserved domain protein	hypothetical protein	protein of unknown function UPF0153	protein of unknown function UPF0153
131	cbdbA614	DET0627	DehaBAV1_0600	VS568	hypothetical protein	hypothetical protein	hypothetical protein	conserved hypothetical protein
132	cbdbA621	DET0635	DehaBAV1_0607	VS575	hypothetical protein	hypothetical protein	hypothetical protein	conserved hypothetical protein
133	cbdbA624	DET0639	DehaBAV1_0611	VS579	hypothetical protein	hypothetical protein	hypothetical protein	conserved hypothetical protein
134	cbdbA630	DET0645	DehaBAV1_0617	VS585	nitroreductase family protein	nitroreductase family protein	nitroreductase	nitroreductase
135	cbdbA631	DET0646	DehaBAV1_0618	VS586	conserved hypothetical protein, nucleotide (SAM) binding motif	hypothetical protein	Methyltransferase type 12	Methyltransferase type 12
136	cbdbA632	DET0648	DehaBAV1_0619	VS587	conserved domain protein	hypothetical protein	hypothetical protein	conserved hypothetical protein
137	cbdbA637	DET0653	DehaBAV1_0623	VS591	conserved hypothetical protein	hypothetical protein	protein of unknown function DUF105	protein of unknown function DUF105
138	cbdbA645	DET0661	DehaBAV1_0630	VS599	thioredoxin	thioredoxin	thioredoxin	thioredoxin
139	cbdbA649	DET0664	DehaBAV1_0633	VS602	hypothetical protein	hypothetical protein	hypothetical protein	conserved hypothetical protein
140	cbdbA659	DET0670	DehaBAV1_0639	VS608	iron-sulfur cluster binding protein	iron-sulfur cluster binding protein	ferredoxin	ferredoxin
141	cbdbA661	DET0672	DehaBAV1_0641	VS610	hypothetical protein	hypothetical protein	hypothetical protein	conserved hypothetical protein
142	cbdbA688	DET0734	DehaBAV1_0665	VS640	conserved domain protein	hypothetical protein	putative regulatory protein, FmdB family	putative regulatory protein, FmdB family

No	CBDB1 core hypothetical genes	195 core hypothetical genes	BAV1 core hypothetical genes	VS core hypothetical genes	CBDB1 annotation	195 annotation	BAV1 annotation	VS annotation
143	cbdbA691	DET0737	DehaBAV1_0667	VS643	putative GTP-binding protein	GTP-binding protein, putative	GTP-binding protein, HSR1-related	GTP-binding protein, HSR1-related
144	cbdbA692	DET0738	DehaBAV1_0668	VS644	hypothetical protein	hypothetical protein	hypothetical protein	conserved hypothetical protein
145	cbdbA727	DET0754	DehaBAV1_0682	VS660	hypothetical protein	hypothetical protein	hypothetical protein	conserved hypothetical protein
146	cbdbA728	DET0755	DehaBAV1_0683	VS661	hypothetical protein	hypothetical protein	hypothetical protein	conserved hypothetical protein
147	cbdbA729	DET0757	DehaBAV1_0684	VS663	conserved hypothetical protein	hypothetical protein	hypothetical protein	conserved hypothetical protein
148	cbdbA734	DET0762	DehaBAV1_0688	VS668	hypothetical membrane protein	hypothetical protein	hypothetical protein	conserved hypothetical membrane protein
149	cbdbA735	DET0763	DehaBAV1_0689	VS669	hypothetical membrane protein	hypothetical protein	hypothetical protein	conserved hypothetical membrane protein
150	cbdbA736	DET0764	DehaBAV1_0690	VS670	conserved hypothetical protein	hypothetical protein	dienelactone hydrolase	dienelactone hydrolase
151	cbdbA737	DET0765	DehaBAV1_0691	VS671	hypothetical protein	hypothetical protein	hypothetical protein	conserved hypothetical protein
152	cbdbA739	DET0767	DehaBAV1_0693	VS673	ATPase, C subunit family protein	ATPase, C subunit family protein	hypothetical protein	ATPase, C subunit family protein
153	cbdbA740	DET0768	DehaBAV1_0694	VS674	conserved membrane protein	hypothetical protein	protein of unknown function UPF0132	hypothetical protein
154	cbdbA744	DET0771	DehaBAV1_0697	VS677	conserved hypothetical protein	hypothetical protein	FAD dependent oxidoreductase	FAD dependent oxidoreductase
155	cbdbA745	DET0772	DehaBAV1_0698	VS678	hypothetical protein	hypothetical protein	hypothetical protein	conserved hypothetical protein
156	cbdbA747	DET0773	DehaBAV1_0699	VS679	conserved membrane protein	CAAX amino terminal protease family protein	Abortive infection protein	Abortive infection protein
157	cbdbA750	DET0776	DehaBAV1_0702	VS682	methyltransferase GidB	methyltransferase GidB	methyltransferase GidB	methyltransferase GidB
158	cbdbA757	DET0781	DehaBAV1_0707	VS687	conserved hypothetical protein	conserved hypothetical protein TIGR00252	protein of unknown function UPF0102	protein of unknown function UPF0102
159	cbdbA763	DET0786	DehaBAV1_0712	VS692	hypothetical membrane protein	hypothetical protein	hypothetical protein	conserved hypothetical protein

No	CBDB1 core hypothetical genes	195 core hypothetical genes	BAV1 core hypothetical genes	VS core hypothetical genes	CBDB1 annotation	195 annotation	BAV1 annotation	VS annotation
160	cbdbA768	DET0790	DehaBAV1_0716	VS696	putative phosphoribosyltransferase	phosphoribosyl transferase domain protein	phosphoribosyltransferase	phosphoribosyltransferase
161	cbdbA778	DET0800	DehaBAV1_0724	VS704	hypothetical protein	hypothetical protein	hypothetical protein	hypothetical protein
162	cbdbA779	DET0801	DehaBAV1_0725	VS705	conserved hypothetical protein	pyrroline-5-carboxylate reductase, putative	NADP oxidoreductase, coenzyme F420-dependent	NADP oxidoreductase, coenzyme F420-dependent
163	cbdbA783	DET0805	DehaBAV1_0729	VS709	major facilitator family transporter	major facilitator family transporter	major facilitator superfamily MFS_1	major facilitator superfamily MFS_1
164	cbdbA785	DET0811	DehaBAV1_0731	VS715	phytoene dehydrogenase family protein	phytoene dehydrogenase family	Phytoene dehydrogenase and related protein-like protein	FAD dependent oxidoreductase
165	cbdbA789	DET0813	DehaBAV1_0733	VS717	ABC transporter, ATP-binding protein	ABC transporter, ATP-binding protein	ABC transporter related	ABC transporter related
166	cbdbA797	DET0820	DehaBAV1_0739	VS724	hypothetical protein	hypothetical protein	hypothetical protein	conserved hypothetical protein
167	cbdbA799	DET0821	DehaBAV1_0740	VS725	conserved domain protein	hypothetical protein	hypothetical protein	conserved hypothetical protein
168	cbdbA800	DET0822	DehaBAV1_0741	VS726	conserved hypothetical protein	hypothetical protein	protein of unknown function DUF101	protein of unknown function DUF101
169	cbdbA801	DET0823	DehaBAV1_0742	VS727	conserved hypothetical protein	hypothetical protein	protein of unknown function UPF0027	protein of unknown function UPF0027
170	cbdbA807	DET0829	DehaBAV1_0748	VS733	membrane protein, MgtC	membrane protein, MgtC / SapB family	MgtC/SapB transporter	MgtC/SapB transporter
171	cbdbA818	DET0837	DehaBAV1_0756	VS741	hypothetical protein	hypothetical protein	hypothetical protein	conserved hypothetical protein
172	cbdbA830	DET0847	DehaBAV1_0766	VS751	conserved domain protein	hypothetical protein	Membrane-bound serine protease (ClpP class)-like protein	conserved hypothetical protein
173	cbdbA831	DET0848	DehaBAV1_0767	VS752	SPFH domain protein	SPFH domain/band 7 family domain protein	band 7 protein	band 7 protein
174	cbdbA833	DET0850	DehaBAV1_0769	VS754	conserved hypothetical protein	hypothetical protein	hypothetical protein	conserved hypothetical protein
175	cbdbA834	DET0851	DehaBAV1_0770	VS755	ABC transproter, ATP-binding protein	sodium extrusion protein NatA, putative	ABC transporter related	ABC transporter related

No	CBDB1 core hypothetical genes	195 core hypothetical genes	BAV1 core hypothetical genes	VS core hypothetical genes	CBDB1 annotation	195 annotation	BAV1 annotation	VS annotation
176	cbdbA835	DET0852	DehaBAV1_0771	VS756	ABC transporter, permease protein	sodium extrusion protein NatB, putative	ABC-type Na+ efflux pump permease component-like protein	ABC transporter, permease protein
177	cbdbA837	DET0854	DehaBAV1_0772	VS758	conserved hypothetical protein	hypothetical protein	hypothetical protein	conserved hypothetical protein
178	cbdbA856	DET0872	DehaBAV1_0790	VS775	membrane protein, MmpL domain	membrane protein, MmpL family	MMPL domain protein	MMPL domain protein
179	cbdbA858	DET0907	DehaBAV1_0792	VS779	conserved hypothetical protein	hypothetical protein	transcriptional regulator, TrmB	transcriptional regulator, TrmB
180	cbdbA860	DET0909	DehaBAV1_0794	VS781	conserved hypothetical membrane protein	hypothetical protein	major facilitator superfamily MFS_1	major facilitator superfamily MFS_1
181	cbdbA861	DET0911	DehaBAV1_0795	VS782	aminopeptidase protein	aminopeptidase protein(EC:3.4.11.-)	peptidase M29, aminopeptidase II	peptidase M29, aminopeptidase II
182	cbdbA863	DET0913	DehaBAV1_0797	VS784	conserved hypothetical protein	hypothetical protein	Rubrerythrin	Rubrerythrin
183	cbdbA864	DET0914	DehaBAV1_0798	VS785	hypothetical protein	hypothetical protein	hypothetical protein	conserved hypothetical protein
184	cbdbA865	DET0915	DehaBAV1_0799	VS786	hypothetical protein	hypothetical protein	hypothetical protein	conserved hypothetical protein
185	cbdbA867	DET0916	DehaBAV1_0800	VS787	conserved hypothetical protein	hypothetical protein	protein of unknown function DUF75	protein of unknown function DUF75
186	cbdbA868	DET0917	DehaBAV1_0801	VS788	hypothetical protein	hypothetical protein	hypothetical protein	conserved hypothetical protein
187	cbdbA869	DET0918	DehaBAV1_0802	VS789	hypothetical protein	hypothetical protein	hypothetical protein	conserved hypothetical protein
188	cbdbA870	DET0919	DehaBAV1_0803	VS790	conserved hypothetical protein	hypothetical protein	protein of unknown function DUF375	protein of unknown function DUF375
189	cbdbA871	DET0920	DehaBAV1_0804	VS791	iron-sulfur cluster-binding protein	iron-sulfur cluster-binding protein	4Fe-4S ferredoxin, iron-sulfur binding domain protein	4Fe-4S ferredoxin, iron-sulfur binding domain protein
190	cbdbA872	DET0921	DehaBAV1_0805	VS792	conserved hypothetical protein	hypothetical protein	protein of unknown function DUF39	protein of unknown function DUF39
191	cbdbA891	DET0937	DehaBAV1_0821	VS808	thioesterase family protein	thioesterase family protein	thioesterase superfamily protein	thioesterase superfamily protein
192	cbdbA893	DET0939	DehaBAV1_0823	VS810	hypothetical protein	hypothetical protein	hypothetical protein	conserved hypothetical protein

No	CBDB1 core hypothetical genes	195 core hypothetical genes	BAV1 core hypothetical genes	VS core hypothetical genes	CBDB1 annotation	195 annotation	BAV1 annotation	VS annotation
193	cbdbA904	DET0949	DehaBAV1_0833	VS820	CBS domain protein	CBS domain protein	CBS domain containing protein	CBS domain containing protein
194	cbdbA905	DET0950	DehaBAV1_0834	VS821	conserved domain protein	hypothetical protein	hypothetical protein	conserved hypothetical protein
195	cbdbA906	DET0951	DehaBAV1_0835	VS822	nifU domain protein	nifU domain protein	nitrogen-fixing NifU domain protein	nitrogen-fixing NifU domain protein
196	cbdbA907	DET0952	DehaBAV1_0836	VS823	conserved hypothetical protein	hypothetical protein	hypothetical protein	conserved hypothetical protein
197	cbdbA909	DET0954	DehaBAV1_0838	VS825	Hsp20	Hsp20/alpha crystallin family protein	heat shock protein Hsp20	heat shock protein Hsp20
198	cbdbA914	DET1511	DehaBAV1_0841	VS829	hypothetical protein	hypothetical protein	hypothetical protein	conserved hypothetical protein
199	cbdbA915	DET0959	DehaBAV1_0850	VS832	Smr domain protein	Smr domain protein	Smr protein/MutS2	Smr protein/MutS2
200	cbdbA918	DET0961	DehaBAV1_0852	VS834	conserved hypothetical protein	hypothetical protein	Protein of unknown function DUF128	Protein of unknown function DUF128
201	cbdbA924	DET0965	DehaBAV1_0856	VS838	conserved hypothetical membrane protein	hypothetical protein	protein of unknown function DUF205	protein of unknown function DUF205
202	cbdbA929	DET0968	DehaBAV1_0859	VS841	conserved hypothetical protein	hypothetical protein	protein of unknown function DUF503	protein of unknown function DUF503
203	cbdbA936	DET0974	DehaBAV1_0865	VS847	puromycin N-acetyltransferase, homolog	puromycin N-acetyltransferase, homolog	hypothetical protein	puromycin N-acetyltransferase-like protein
204	cbdbA937	DET0975	DehaBAV1_0866	VS848	acetyltransferase, GNAT family	acetyltransferase, GNAT family	GCN5-related N-acetyltransferase	GCN5-related N-acetyltransferase
205	cbdbA948	DET0984	DehaBAV1_0875	VS856	conserved hypothetical protein	hypothetical protein	protein of unknown function DUF448	protein of unknown function DUF448
206	cbdbA949	DET0985	DehaBAV1_0876	VS857	N utilization substance protein A	transcription elongation factor NusA	NusA antitermination factor	NusA antitermination factor
207	cbdbA950	DET0986	DehaBAV1_0877	VS858	Mg chelatase, homolog	Mg chelatase-like protein	Mg chelatase, subunit ChlI	Mg chelatase, subunit ChlI
208	cbdbA951	DET0987	DehaBAV1_0878	VS859	acetyltransferase, GNAT family	acetyltransferase, GNAT family	GCN5-related N-acetyltransferase	acetyltransferase, GNAT family
209	cbdbA952	DET0988	DehaBAV1_0879	VS860	acetyltransferase, GNAT family	acetyltransferase, GNAT family	GCN5-related N-acetyltransferase	GCN5-related N-acetyltransferase
210	cbdbA953	DET0989	DehaBAV1_0880	VS861	conserved hypothetical protein	hypothetical protein	protein of unknown function DUF502	protein of unknown function DUF502

No	CBDB1 core hypothetical genes	195 core hypothetical genes	BAV1 core hypothetical genes	VS core hypothetical genes	CBDB1 annotation	195 annotation	BAV1 annotation	VS annotation
211	cbdbA962	DET1001	DehaBAV1_0889	VS873	conserved hypothetical protein	hypothetical protein	LmbE family protein	LmbE family protein
212	cbdbA964	DET1003	DehaBAV1_0891	VS875	conserved hypothetical protein	hypothetical protein	hypothetical protein	conserved hypothetical protein
213	cbdbA969	DET1007	DehaBAV1_0894	VS878	redox-active disulfide protein 2	redox-active disulfide protein 2	redox-active disulfide protein 2	redox-active disulfide protein 2
214	cbdbA971	DET1008	DehaBAV1_0895	VS879	conserved domain protein	hypothetical protein	hypothetical protein	conserved hypothetical protein
215	cbdbA981	DET1010	DehaBAV1_0898	VS882	hypothetical membrane protein	hypothetical protein	hypothetical protein	conserved hypothetical membrane protein
216	cbdbA982	DET1011	DehaBAV1_0899	VS883	hypothetical protein	hypothetical protein	hypothetical protein	conserved hypothetical protein
217	cbdbA983	DET1013	DehaBAV1_0900	VS884	hypothetical protein	hypothetical protein	hypothetical protein	conserved hypothetical protein
218	cbdbA985	DET1014	DehaBAV1_0901	VS885	ACT domain protein	ACT domain protein	ACT domain-containing protein-like protein	ACT domain protein
219	cbdbA990	DET1018	DehaBAV1_0904	VS888	conserved hypothetical protein	hypothetical protein	hypothetical protein	conserved hypothetical protein
220	cbdbA992	DET1021	DehaBAV1_0905	VS894	hypothetical protein	hypothetical protein	hypothetical protein	conserved hypothetical protein
221	cbdbA995	DET1026	DehaBAV1_0909	VS898	hypothetical protein	hypothetical protein	hypothetical protein	conserved hypothetical protein
222	cbdbA998	DET1027	DehaBAV1_0910	VS899	putative acylphosphatase	acylphosphatase, putative	acylphosphatase	acylphosphatase
223	cbdbA999	DET1028	DehaBAV1_0911	VS900	conserved hypothetical protein	hypothetical protein	hypothetical protein	conserved hypothetical protein
224	cbdbA1001	DET1030	DehaBAV1_0913	VS901	cytosol aminopeptidase	cytosol aminopeptidase family protein(EC:3.4.11.1)	Leucyl aminopeptidase(EC:3.4.11.1)	Leucyl aminopeptidase(EC:3.4.11.1)
225	cbdbA1002	DET1031	DehaBAV1_0914	VS902	hypothetical protein	hypothetical protein	kinase-like protein	conserved hypothetical protein
226	cbdbA1004	DET1032	DehaBAV1_0915	VS903	degV family protein	degV family protein	degV family protein	degV family protein
227	cbdbA1014	DET1039	DehaBAV1_0921	VS909	putative SpoIIIJ-associated protein Jag	SpoIIIJ-associated protein Jag, putative	single-stranded nucleic acid binding R3H domain protein	single-stranded nucleic acid binding R3H domain protein

No	CBDB1 core hypothetical genes	195 core hypothetical genes	BAV1 core hypothetical genes	VS core hypothetical genes	CBDB1 annotation	195 annotation	BAV1 annotation	VS annotation
228	cbdbA1016	DET1041	DehaBAV1_0923	VS911	hypothetical protein	PQQ enzyme repeat domain protein	Pyrrolo-quinoline quinone	Pyrrolo-quinoline quinone
229	cbdbA1021	DET1051	DehaBAV1_0930	VS919	conserved domain protein	hypothetical protein	hypothetical protein	conserved hypothetical protein
230	cbdbA1024	DET1053	DehaBAV1_0932	VS921	conserved domain protein	hypothetical protein	hypothetical protein	conserved hypothetical protein
231	cbdbA1028	DET1056	DehaBAV1_0935	VS924	hypothetical transport protein	hypothetical protein	protein of unknown function DUF6, transmembrane	protein of unknown function DUF6, transmembrane
232	cbdbA1030	DET1057	DehaBAV1_0936	VS925	conserved hypothetical protein	hypothetical protein	hypothetical protein	conserved hypothetical protein
233	cbdbA1040	DET1061	DehaBAV1_0944	VS933	metallo-beta-lactamase family protein	metallo-beta-lactamase family protein	beta-lactamase domain protein	beta-lactamase domain protein
234	cbdbA1046	DET1120	DehaBAV1_0949	VS938	maf-like protein	septum formation protein MaF	maf protein	maf protein
235	cbdbA1048	DET1121	DehaBAV1_0950	VS939	conserved hypothetical protein	hypothetical protein	hypothetical protein	conserved hypothetical protein
236	cbdbA1062	DET1134	DehaBAV1_0963	VS952	conserved hypothetical membrane protein	hypothetical protein	protein of unknown function DUF204	hypothetical protein
237	cbdbA1064	DET1136	DehaBAV1_0965	VS954	hypothetical protein	hypothetical protein	hypothetical protein	conserved hypothetical protein
238	cbdbA1066	DET1137	DehaBAV1_0966	VS955	radical SAM domain protein	radical SAM domain protein	Radical SAM domain protein	Radical SAM domain protein
239	cbdbA1071	DET1141	DehaBAV1_0970	VS959	glycosyl transferase	glycosyl transferase/polysaccharide deacetylase family protein	polysaccharide deacetylase	polysaccharide deacetylase
240	cbdbA1072	DET1142	DehaBAV1_0971	VS960	hypothetical protein	hypothetical protein	hypothetical protein	conserved hypothetical protein
241	cbdbA1073	DET1143	DehaBAV1_0972	VS961	conserved hypothetical membrane protein	hypothetical protein	membrane protein-like protein	conserved hypothetical protein
242	cbdbA1088	DET1164	DehaBAV1_0986	VS965	iron-sulfur cluster-binding protein	iron-sulfur cluster-binding protein	4Fe-4S ferredoxin, iron-sulfur binding domain protein	4Fe-4S ferredoxin, iron-sulfur binding domain protein
243	cbdbA1095	DET1169	DehaBAV1_0990	VS1358	conserved hypothetical protein	hypothetical protein	putative regulatory protein, FmdB family	putative regulatory protein, FmdB family
244	cbdbB24	DET1168	DehaBAV1_0991	VS1357	conserved hypothetical protein	hypothetical protein	hypothetical protein	conserved hypothetical protein

No	CBDB1 core hypothetical genes	195 core hypothetical genes	BAV1 core hypothetical genes	VS core hypothetical genes	CBDB1 annotation	195 annotation	BAV1 annotation	VS annotation
245	cbdbA1099	DET1185	DehaBAV1_0996	VS968	hypothetical protein	hypothetical protein	hypothetical protein	conserved hypothetical protein
246	cbdbA1100	DET1186	DehaBAV1_0997	VS969	conserved domain protein	hypothetical protein	hypothetical protein	conserved hypothetical protein
247	cbdbA1106	DET1191	DehaBAV1_1002	VS974	hypothetical periplasmic protein	lipoprotein, putative	protein of unknown function DUF541	protein of unknown function DUF541
248	cbdbA1109	DET1194	DehaBAV1_1005	VS977	conserved hypothetical protein	hypothetical protein	secreted endonuclease distantly related to Holliday junction resolvase-like protein	conserved hypothetical protein
249	cbdbA1128	DET1211	DehaBAV1_1021	VS994	SAM-dependent methyltransferase HemK family	modification methylase, HemK family	modification methylase, HemK family	modification methylase, HemK family
250	cbdbA1131	DET1213	DehaBAV1_1023	VS996	hypothetical membrane protein	hypothetical protein	protein of unknown function DUF95, transmembrane	protein of unknown function DUF95, transmembrane
251	cbdbA1132	DET1214	DehaBAV1_1024	VS997	hypothetical protein	hypothetical protein	hypothetical protein	conserved hypothetical protein
252	cbdbA1135	DET1217	DehaBAV1_1027	VS1000	acetyltransferase, GNAT family	acetyltransferase, GNAT family	GCN5-related N-acetyltransferase	GCN5-related N-acetyltransferase
253	cbdbA1138	DET1220	DehaBAV1_1030	VS1003	hypothetical protein	hypothetical protein	hypothetical protein	conserved hypothetical protein
254	cbdbA1139	DET1222	DehaBAV1_1031	VS1004	conserved hypothetical protein	hypothetical protein	hypothetical protein	conserved hypothetical protein
255	cbdbA1151	DET1235	DehaBAV1_1049	VS1020	conserved hypothetical protein	hypothetical protein	hypothetical protein	conserved hypothetical protein
256	cbdbA1159	DET1240	DehaBAV1_1053	VS1025	probable peptidylprolyl isomerase	peptidyl-prolyl cis-trans isomerase, cyclophilin-type(EC:5.2.1.8)	Peptidylprolyl isomerase(EC:5.2.1.8)	Peptidylprolyl isomerase(EC:5.2.1.8)
257	cbdbA1164	DET1244	DehaBAV1_1057	VS1029	putative iron-sulfur flavoprotein	NADPH-dependent FMN reductase	NADPH-dependent FMN reductase	NADPH-dependent FMN reductase
258	cbdbA1165	DET1245	DehaBAV1_1058	VS1030	TfoX domain protein	TfoX domain protein	TfoX, N-terminal domain protein	TfoX, N-terminal domain protein
259	cbdbA1167	DET1246	DehaBAV1_1059	VS1031	hypothetical protein	hypothetical protein	hypothetical protein	conserved hypothetical protein

No	CBDB1 core hypothetical genes	195 core hypothetical genes	BAV1 core hypothetical genes	VS core hypothetical genes	CBDB1 annotation	195 annotation	BAV1 annotation	VS annotation
260	cbdbA1169	DET1248	DehaBAV1_1061	VS1033	conserved hypothetical membrane protein	hypothetical protein	protein of unknown function DUF1648	protein of unknown function DUF1648
261	cbdbA1171	DET1250	DehaBAV1_1062	VS1034	hypothetical membrane protein	hypothetical protein	hypothetical protein	conserved hypothetical protein
262	cbdbA1172	DET1251	DehaBAV1_1063	VS1035	NADPH-dependent FMN reductase	NADPH-dependent FMN reductase	NADPH-dependent FMN reductase	NADPH-dependent FMN reductase
263	cbdbA1174	DET1252	DehaBAV1_1064	VS1036	hypothetical protein	hypothetical protein	hypothetical protein	conserved hypothetical protein
264	cbdbA1176	DET1253	DehaBAV1_1065	VS1037	hypothetical protein	hypothetical protein	hypothetical protein	conserved hypothetical protein
265	cbdbA1182	DET1259	DehaBAV1_1070	VS1042	hypothetical protein	hypothetical protein	hypothetical protein	conserved hypothetical protein
266	cbdbA1190	DET1264	DehaBAV1_1075	VS1047	degV family protein	degV family protein	degV family protein	degV family protein
267	cbdbA1191	DET1265	DehaBAV1_1076	VS1048	degV family protein	degV family protein	degV family protein	degV family protein
268	cbdbA1192	DET1266	DehaBAV1_1077	VS1049	degV family protein	degV family protein	degV family protein	degV family protein
269	cbdbA1193	DET1267	DehaBAV1_1078	VS1050	degV family protein	degV family protein	degV family protein	degV family protein
270	cbdbA1198	DET1271	DehaBAV1_1082	VS1054	conserved hypothetical protein	hypothetical protein	putative transcriptional regulator, AsnC family	putative transcriptional regulator, AsnC family
271	cbdbA1201	DET1273	DehaBAV1_1084	VS1056	hypothetical protein	hypothetical protein	hypothetical protein	conserved hypothetical protein
272	cbdbA1202	DET1274	DehaBAV1_1085	VS1057	conserved hypothetical protein	hypothetical protein	metal-binding possibly nucleic acid-binding protein-like protein	conserved hypothetical protein
273	cbdbA1208	DET1279	DehaBAV1_1090	VS1062	acyl carrier protein	acyl carrier protein	acyl carrier protein	acyl carrier protein
274	cbdbA1210	DET1280	DehaBAV1_1091	VS1063	radical SAM domain protein	radical SAM/B12 binding domain protein	Radical SAM domain protein	Radical SAM domain protein
275	cbdbA1213	DET1283	DehaBAV1_1094	VS1066	hypothetical protein	hypothetical protein	hypothetical protein	conserved hypothetical protein
276	cbdbA1215	DET1284	DehaBAV1_1095	VS1067	hypothetical protein	hypothetical protein	hypothetical protein	conserved hypothetical protein
277	cbdbA1227	DET1290	DehaBAV1_1101	VS1073	NUDIX domain protein	NUDIX domain protein	NUDIX hydrolase	NUDIX hydrolase
278	cbdbA1235	DET1295	DehaBAV1_1107	VS1079	hypothetical protein	hypothetical protein	dienelactone hydrolase-like protein	hypothetical protein

No	CBDB1 core hypothetical genes	195 core hypothetical genes	BAV1 core hypothetical genes	VS core hypothetical genes	CBDB1 annotation	195 annotation	BAV1 annotation	VS annotation
279	cbdbA1239	DET1300	DehaBAV1_1111	VS1083	universal stress protein family	universal stress protein family	UspA domain protein	UspA domain protein
280	cbdbA1241	DET1303	DehaBAV1_1113	VS1085	fasciclin domain protein	fasciclin domain protein	beta-Ig-H3/fasciclin	beta-Ig-H3/fasciclin
281	cbdbA1244	DET1305	DehaBAV1_1114	VS1086	conserved hypothetical protein	hypothetical protein	hypothetical protein	conserved hypothetical protein
282	cbdbA1245	DET1306	DehaBAV1_1115	VS1087	hypothetical protein	hypothetical protein	hypothetical protein	conserved hypothetical protein
283	cbdbA1247	DET1307	DehaBAV1_1117	VS1089	conserved hypothetical protein	hypothetical protein	protein of unknown function DUF1508	protein of unknown function DUF1508
284	cbdbA1248	DET1308	DehaBAV1_1118	VS1090	putative hydrolase	hydrolase, alpha/beta fold family	alpha/beta hydrolase fold	alpha/beta hydrolase fold
285	cbdbA1249	DET1309	DehaBAV1_1119	VS1091	ATPase, AAA family	ATPase, AAA family	AAA ATPase, central domain protein	AAA ATPase, central domain protein
286	cbdbA1251	DET1310	DehaBAV1_1120	VS1092	conserved domain protein	hypothetical protein	hypothetical protein	conserved hypothetical protein
287	cbdbA1252	DET1311	DehaBAV1_1121	VS1093	hypothetical protein	hypothetical protein	hypothetical protein	hypothetical protein
288	cbdbA1253	DET1313	DehaBAV1_1122	VS1094	conserved hypothetical protein	protein of unknown function /conserved hypothetical protein TIGR00296	protein of unknown function DUF52	protein of unknown function DUF52
289	cbdbA1254	DET1314	DehaBAV1_1123	VS1095	radical SAM domain protein	radical SAM domain protein	Radical SAM domain protein	Radical SAM domain protein
290	cbdbA1255	DET1315	DehaBAV1_1124	VS1096	hypothetical membrane protein	hypothetical protein	protein of unknown function DUF6, transmembrane	protein of unknown function DUF6, transmembrane
291	cbdbB27	DET1317	DehaBAV1_1125	VS1098	conserved domain protein	hypothetical protein	hypothetical protein	hypothetical protein
292	cbdbA1256	DET1318	DehaBAV1_1126	VS1099	conserved hypothetical protein	hypothetical protein	protein of unknown function DUF520	protein of unknown function DUF520
293	cbdbA1260	DET1320	DehaBAV1_1129	VS1101	conserved hypothetical protein	hypothetical protein	AAA ATPase	AAA ATPase
294	cbdbA1264	DET1321	DehaBAV1_1131	VS1103	acetyltransferase, GNAT family	acetyltransferase, GNAT family	GCN5-related N-acetyltransferase	GCN5-related N-acetyltransferase
295	cbdbA1266	DET1322	DehaBAV1_1133	VS1104	hypothetical protein	hypothetical protein	hypothetical protein	conserved hypothetical protein
296	cbdbA1275	DET1328	DehaBAV1_1139	VS1110	putative membrane protein	hypothetical protein	protein of unknown function DUF1385	protein of unknown function DUF1385

No	CBDB1 core hypothetical genes	195 core hypothetical genes	BAV1 core hypothetical genes	VS core hypothetical genes	CBDB1 annotation	195 annotation	BAV1 annotation	VS annotation
297	cbdbA1277	DET1330	DehaBAV1_1141	VS1112	acetyltransferase, GNAT family	acetyltransferase, GNAT family	GCN5-related N-acetyltransferase	GCN5-related N-acetyltransferase
298	cbdbA1279	DET1332	DehaBAV1_1143	VS1114	conserved hypothetical protein	hypothetical protein	hypothetical protein	conserved hypothetical protein
299	cbdbA1281	DET1333	DehaBAV1_1144	VS1115	conserved hypothetical protein	hypothetical protein	protein of unknown function DUF107	protein of unknown function DUF107
300	cbdbA1285	DET1336	DehaBAV1_1147	VS1118	hypothetical protein	hypothetical protein	hypothetical protein	conserved hypothetical protein
301	cbdbA1287	DET1337	DehaBAV1_1148	VS1119	hypothetical membrane protein	hypothetical protein	hypothetical protein	conserved hypothetical protein
302	cbdbA1297	DET1347	DehaBAV1_1158	VS1129	hypothetical protein	hypothetical protein	hypothetical protein	conserved hypothetical protein
303	cbdbA1306	DET1355	DehaBAV1_1167	VS1137	hypothetical protein	hypothetical protein	hypothetical protein	conserved hypothetical protein
304	cbdbA1307	DET1356	DehaBAV1_1168	VS1138	hypothetical protein	hypothetical protein	hypothetical protein	conserved hypothetical protein
305	cbdbA1308	DET1357	DehaBAV1_1169	VS1139	hypothetical protein	hypothetical protein	hypothetical protein	conserved hypothetical protein
306	cbdbA1315	DET1364	DehaBAV1_1175	VS1145	conserved hypothetical protein	hypothetical protein	RDD domain containing protein	RDD domain containing protein
307	cbdbA1318	DET1367	DehaBAV1_1178	VS1148	hypothetical protein	hypothetical protein	hypothetical protein	conserved hypothetical protein
308	cbdbA1319	DET1368	DehaBAV1_1179	VS1149	radical SAM domain protein	radical SAM domain protein	Radical SAM domain protein	Radical SAM domain protein
309	cbdbA1320	DET1369	DehaBAV1_1180	VS1150	hypothetical protein	hypothetical protein	hypothetical protein	hypothetical protein
310	cbdbA1322	DET1370	DehaBAV1_1181	VS1151	conserved hypothetical protein	conserved hypothetical protein TIGR00043	protein of unknown function UPF0054	protein of unknown function UPF0054
311	cbdbA1323	DET1371	DehaBAV1_1182	VS1152	NADPH-dependent FMN reductase	NADPH-dependent FMN reductase	NADPH-dependent FMN reductase	NADPH-dependent FMN reductase
312	cbdbA1330	DET1376	DehaBAV1_1187	VS1157	conserved hypothetical protein	hypothetical protein	hypothetical protein	conserved hypothetical protein
313	cbdbA1331	DET1377	DehaBAV1_1188	VS1158	hypothetical protein	hypothetical protein	hypothetical protein	conserved hypothetical protein
314	cbdbA1332	DET1378	DehaBAV1_1189	VS1159	hypothetical membrane protein	hypothetical protein	hypothetical protein	conserved hypothetical membrane protein

No	CBDB1 core hypothetical genes	195 core hypothetical genes	BAV1 core hypothetical genes	VS core hypothetical genes	CBDB1 annotation	195 annotation	BAV1 annotation	VS annotation
315	cbdbA1333	DET1379	DehaBAV1_1190	VS1160	auxin-responsive GH3 protein homolog	auxin-responsive GH3 protein homolog, putative	GH3 auxin-responsive promoter	auxin-responsive GH3 protein-like protein
316	cbdbA1335	DET1381	DehaBAV1_1191	VS1162	GGDEF domain	GGDEF domain/HD domain protein	diguanylate cyclase and metal dependent phosphohydrolase	metal dependent phosphohydrolase
317	cbdbA1349	DET1392	DehaBAV1_1201	VS1173	rhodanese-like domain protein	rhodanese-like domain protein	Rhodanese domain protein	Rhodanese domain protein
318	cbdbA1350	DET1393	DehaBAV1_1202	VS1174	hypothetical protein	hypothetical protein	hypothetical protein	conserved hypothetical protein
319	cbdbA1354	DET1395	DehaBAV1_1204	VS1177	GTP-binding protein EngA	GTP-binding protein	small GTP-binding protein	small GTP-binding protein
320	cbdbA1355	DET1396	DehaBAV1_1205	VS1178	conserved hypothetical membrane protein	conserved hypothetical protein TIGR00023	protein of unknown function DUF205	protein of unknown function DUF205
321	cbdbA1363	DET1402	DehaBAV1_1211	VS1184	conserved hypothetical protein	hypothetical protein	Zn-dependent hydrolase of the beta-lactamase fold-like protein	conserved hypothetical protein
322	cbdbA1365	DET1403	DehaBAV1_1212	VS1185	conserved domain protein	hypothetical protein	hypothetical protein	peptidase C14, caspase catalytic subunit p20
323	cbdbA1366	DET1404	DehaBAV1_1213	VS1186	conserved hypothetical protein	conserved hypothetical protein, truncation	exonuclease-like protein	conserved hypothetical protein
324	cbdbA1368	DET1407	DehaBAV1_1214	VS1188	conserved domain protein	BNR/Asp-box repeat domain protein	hypothetical protein	Fibronectin, type III domain protein
325	cbdbA1375	DET1414	DehaBAV1_1221	VS1195	hypothetical membrane protein	hypothetical protein	hypothetical protein	conserved hypothetical protein
326	cbdbA1383	DET1419	DehaBAV1_1227	VS1201	conserved hypothetical protein	transcriptional regulator, AbrB family	transcriptional regulator, AbrB family	transcriptional regulator, AbrB family
327	cbdbA1384	DET1420	DehaBAV1_1228	VS1202	SAM-dependent methyltransferase UbiE/COQ5 family	methyltransferase, UbiE/COQ5 family	Methyltransferase type 11	Methyltransferase type 11
328	cbdbA1385	DET1421	DehaBAV1_1229	VS1203	PP-loop family protein	PP-loop family protein	tRNA(Ile)-lysidine synthetase	tRNA(Ile)-lysidine synthetase

No	CBDB1 core hypothetical genes	195 core hypothetical genes	BAV1 core hypothetical genes	VS core hypothetical genes	CBDB1 annotation	195 annotation	BAV1 annotation	VS annotation
329	cbdbA1387	DET1423	DehaBAV1_1231	VS1205	peptidase, M48 family	peptidase, M48 family	Ste24 endopeptidase(EC:3.4.24.84)	Ste24 endopeptidase(EC:3.4.24.84)
330	cbdbA1388	DET1424	DehaBAV1_1232	VS1206	conserved hypothetical protein	hypothetical protein	Uncharacterized protein-like protein	conserved hypothetical protein
331	cbdbA1389	DET1425	DehaBAV1_1233	VS1207	probable hemolysin-related protein	hypothetical protein	protein of unknown function DUF21	protein of unknown function DUF21
332	cbdbA1394	DET1429	DehaBAV1_1237	VS1211	peptidase, M16 family	peptidase, M16 family	peptidase M16 domain protein	peptidase M16 domain protein
333	cbdbA1405	DET1438	DehaBAV1_1245	VS1219	hypothetical protein	hypothetical protein	hypothetical protein	conserved hypothetical protein
334	cbdbA1414	DET1449	DehaBAV1_1251	VS1228	membrane transporter, MarC family	membrane protein, MarC family	multiple antibiotic resistance (MarC)-related protein	multiple antibiotic resistance (MarC)-related protein
335	cbdbA1416	DET1450	DehaBAV1_1252	VS1229	conserved hypothetical protein	hypothetical protein	cyclase/dehydrase	cyclase/dehydrase
336	cbdbA1417	DET1451	DehaBAV1_1253	VS1230	hypothetical protein	hypothetical protein	hypothetical protein	conserved hypothetical protein
337	cbdbA1418	DET1452	DehaBAV1_1254	VS1231	conserved hypothetical protein	hypothetical protein	hypothetical protein	conserved hypothetical protein
338	cbdbA1420	DET1455	DehaBAV1_1256	VS1233	conserved hypothetical protein	hypothetical protein	amidohydrolase 2	amidohydrolase 2
339	cbdbA1422	DET1457	DehaBAV1_1258	VS1235	hypothetical protein	hypothetical protein	hypothetical protein	conserved hypothetical protein
340	cbdbA1424	DET1458	DehaBAV1_1259	VS1236	putative comF family protein	comF family protein, putative	amidophosphoribosyltransferase-like protein	ComF family protein, putative
341	cbdbA1430	DET1461	DehaBAV1_1262	VS1239	hypothetical periplasmic protein	lipoprotein, putative	secreted protein-like protein	lipoprotein, putative
342	cbdbA1433	DET1463	DehaBAV1_1264	VS1241	hypothetical protein	hypothetical protein	hypothetical protein	conserved hypothetical protein
343	cbdbA1437	DET1465	DehaBAV1_1266	VS1243	integral membrane protein TIGR01906	integral membrane protein TIGR01906	integral membrane protein TIGR01906	integral membrane protein TIGR01906
344	cbdbA1438	DET1466	DehaBAV1_1267	VS1244	hypothetical protein	hypothetical protein	hypothetical protein	conserved hypothetical protein
345	cbdbA1440	DET1468	DehaBAV1_1269	VS1245	conserved hypothetical protein	hypothetical protein	putative regulatory protein, FmdB family	putative regulatory protein, FmdB family
346	cbdbA1465	DET1495	DehaBAV1_1285	VS1271	conserved hypothetical protein	hypothetical protein	Methyltransferase type 12	Methyltransferase type 12

No	CBDB1 core hypothetical genes	195 core hypothetical genes	BAV1 core hypothetical genes	VS core hypothetical genes	CBDB1 annotation	195 annotation	BAV1 annotation	VS annotation
347	cbdbA1470	DET1499	DehaBAV1_1289	VS1274	conserved hypothetical protein	hypothetical protein	Helix-turn-helix, type 11 domain protein	Helix-turn-helix, type 11 domain protein
348	cbdbA1471	DET1500	DehaBAV1_1290	VS1275	conserved hypothetical protein	hypothetical protein	hypothetical protein	conserved hypothetical protein
349	cbdbA1474	DET1502	DehaBAV1_1292	VS1276	major facilitator family transporter	major facilitator family protein	major facilitator superfamily MFS_1	major facilitator superfamily MFS_1
350	cbdbA1477	DET1504	DehaBAV1_1294	VS1278	iron(II) transport protein A	ferrous iron transport protein A, putative	FeoA family protein	FeoA family protein
351	cbdbA1479	DET1506	DehaBAV1_1296	VS1280	SsrA-binding protein	SsrA-binding protein	SsrA-binding protein	SsrA-binding protein
352	cbdbA1646	DET1564	DehaBAV1_1309	VS1443	hypothetical protein	hypothetical protein	hypothetical protein	conserved hypothetical protein
353	cbdbA1649	DET1568	DehaBAV1_1313	VS1446	hypothetical membrane protein	hypothetical protein	hypothetical protein	conserved hypothetical membrane protein
354	cbdbA1650	DET1569	DehaBAV1_1314	VS1447	hypothetical protein	hypothetical protein	hypothetical protein	conserved hypothetical protein
355	cbdbA1670	DET1582	DehaBAV1_1331	VS1461	conserved domain protein	hypothetical protein	hypothetical protein	conserved hypothetical protein
356	cbdbA1671	DET1584	DehaBAV1_1332	VS1468	hypothetical protein	hypothetical protein	hypothetical protein	conserved hypothetical protein
357	cbdbA1673	DET1585	DehaBAV1_1333	VS1469	conserved hypothetical protein	hypothetical protein	hypothetical protein	conserved hypothetical protein
358	cbdbA1675	DET1586	DehaBAV1_1334	VS1470	lemA family protein	lemA family protein	LemA family protein	LemA family protein
359	cbdbA1677	DET1587	DehaBAV1_1335	VS1471	putative peptidase	peptidase, M28 family	peptidase M48, Ste24p	peptidase M48, Ste24p
360	cbdbA1684	DET1592	DehaBAV1_1340	VS1476	AIR synthase domain protein	AIR synthase domain protein	AIR synthase related protein domain protein	AIR synthase related protein domain protein
361	cbdbA1686	DET1595	DehaBAV1_1342	VS1478	ApbE	ApbE/NosX family protein	ApbE family lipoprotein	ApbE family lipoprotein
362	cbdbA1702	DET1608	DehaBAV1_1354	VS1490	KH domain protein	KH domain/HDIG domain protein	metal dependent phosphohydrolase	metal dependent phosphohydrolase
363	cbdbA1703	DET1609	DehaBAV1_1355	VS1491	conserved hypothetical protein	Ser/Thr protein phosphatase family protein	metallophosphoestera se	metallophosphoesteras e

No	CBDB1 core hypothetical genes	195 core hypothetical genes	BAV1 core hypothetical genes	VS core hypothetical genes	CBDB1 annotation	195 annotation	BAV1 annotation	VS annotation
364	cbdbA1704	DET1610	DehaBAV1_1356	VS1492	PHP domain N-terminal region family protein	PHP domain N-terminal region family protein	PHP C-terminal domain protein	PHP C-terminal domain protein
365	cbdbA1705	DET1611	DehaBAV1_1357	VS1493	conserved hypothetical membrane protein	hypothetical protein	protein of unknown function DUF205	protein of unknown function DUF205
366	cbdbA1707	DET1613	DehaBAV1_1359	VS1495	conserved hypothetical protein TIGR00104	conserved hypothetical protein TIGR00104	protein of unknown function UPF0066	protein of unknown function UPF0066
367	cbdbA1709	DET1615	DehaBAV1_1361	VS1497	HIT domain protein	HIT domain protein	histidine triad (HIT) protein	histidine triad (HIT) protein
368	cbdbA1711	DET1617	DehaBAV1_1363	VS1499	conserved hypothetical protein	hypothetical protein	protein of unknown function DUF74	protein of unknown function DUF74
369	cbdbA1717	DET1621	DehaBAV1_1367	VS1503	hypothetical protein	hypothetical protein	cell wall/surface repeat protein	cell wall/surface repeat protein
370	cbdbA1718	DET1622	DehaBAV1_1368	VS1504	conserved hypothetical protein	hypothetical protein	DNA-binding protein with PD1-like DNA-binding-like protein	conserved hypothetical protein
371	cbdbA1720	DET1623	DehaBAV1_1369	VS1505	putative desulforedoxin	desulforedoxin, putative	Desulfoferrodoxin Dfx domain protein	Desulfoferrodoxin Dfx domain protein
372	cbdbA1721	DET1624	DehaBAV1_1370	VS1506	hypothetical protein	hypothetical protein	hypothetical protein	conserved hypothetical protein
373	cbdbA1727	DET1629	DehaBAV1_1374	VS1511	radical SAM domain protein	radical SAM domain protein	Radical SAM domain protein	Radical SAM domain protein
374	cbdbA1729	DET1631	DehaBAV1_1376	VS1513	TPR domain protein	TPR domain protein	TPR repeat-containing protein	TPR repeat-containing protein
375	cbdbA1730	DET1632	DehaBAV1_1377	VS1514	nifU protein, homolog	NifU-like protein	nitrogen-fixing NifU domain protein	HesB/YadR/YfhF-family protein
376	cbdbA1733	DET1634	DehaBAV1_1379	VS1516	hypothetical protein	hypothetical protein	hypothetical protein	conserved hypothetical protein
377	cbdbA1735	DET1637	DehaBAV1_1381	VS1519	mazG family protein	mazG family protein	MazG family protein	MazG family protein
378	cbdbA1737	DET1638	DehaBAV1_1382	VS1520	conserved hypothetical protein	hypothetical protein	peptidase, membrane zinc metallopeptidase, putative	peptidase, membrane zinc metallopeptidase, putative
379	cbdbA1015	DET1040	DehaBAV1_0922	VS910	putative inner membrane protein, 60 kDa	inner membrane protein, 60 kDa	putative inner membrane protein, 60 kDa	60 kDa inner membrane insertion protein
380	cbdbA1640	DET1548	DehaBAV1_1304	VS1438	conserved hypothetical protein	hypothetical protein	conserved hypothetical protein	protein of unknown function DUF62

No	CBDB1 core hypothetical genes	195 core hypothetical genes	BAV1 core hypothetical genes	VS core hypothetical genes	CBDB1 annotation	195 annotation	BAV1 annotation	VS annotation
381	cbdbA195	DET0187	DehaBAV1_0165	VS176	formate dehydrogenase, major subunit	formate dehydrogenase, alpha subunit(EC:1.2.1.2)	formate dehydrogenase, alpha subunit(EC:1.2.1.2)	formate dehydrogenase, alpha subunit(EC:1.2.1.2)
382	cbdbA193	DET0186	DehaBAV1_0166	VS175	formate dehydrogenase, membrane subunit	formate dehydrogenase, membrane subunit, putative	Polysulphide reductase, NrfD	Polysulphide reductase, NrfD
383	cbdbA191	DET0185	DehaBAV1_0167	VS174	formate dehydrogenase accessory protein	formate dehydrogenase accessory protein FdhE, putative	Uncharacterized protein involved in formate dehydrogenase formation-like protein	formate dehydrogenase accessory protein FdhE, putative
384	cbdbA121	DET0103	DehaBAV1_0265	VS113	putative molybdopterin oxidoreductase, iron-sulfur binding subunit	molybdopterin oxidoreductase, iron-sulfur binding subunit, putative	4Fe-4S ferredoxin, iron-sulfur binding domain protein	4Fe-4S ferredoxin, iron-sulfur binding domain protein
385	cbdbA120	DET0102	DehaBAV1_0266	VS112	putative molybdopterin oxidoreductase, membrane subunit	molybdopterin oxidoreductase, membrane subunit, putative	Polysulphide reductase, NrfD	Polysulphide reductase, NrfD
386	cbdbA118	DET0101	DehaBAV1_0267	VS111	molybdopterin oxidoreductase	molybdopterin oxidoreductase	molybdopterin oxidoreductase Fe4S4 region	molybdopterin oxidoreductase
387	cbdbA115	DET0099	DehaBAV1_0269	VS109	putative molybdopterin-guanine dinucleotide biosynthesis protein A	mobA family protein	4-diphosphocytidyl-2C-methyl-D-erythritol synthase	MobA family protein
388	cbdbA134	DET0115	DehaBAV1_0254	VS124	ABC transporter, permease protein	ABC transporter, permease protein, putative	ABC-2 type transporter	ABC-2 type transporter
389	cbdbA133	DET0114	DehaBAV1_0255	VS123	ABC transporter, permease protein	ABC transporter, substrate-binding protein, putative	ABC-2 type transporter	ABC-2 type transporter
390	cbdbA271	DET0331	DehaBAV1_0311	VS273	cation efflux family protein	cation efflux family protein	cation diffusion facilitator family transporter	cation diffusion facilitator family transporter

No	CBDB1 core hypothetical genes	195 core hypothetical genes	BAV1 core hypothetical genes	VS core hypothetical genes	CBDB1 annotation	195 annotation	BAV1 annotation	VS annotation
391	cbdbA273	DET0332	DehaBAV1_0312	VS274	protein-export membrane protein SecF	protein-export membrane protein SecF	protein-export membrane protein SecF	protein-export membrane protein SecF
392	cbdbA274	DET0333	DehaBAV1_0313	VS275	protein-export membrane protein SecD	protein-export membrane protein SecD	protein-export membrane protein SecD	protein-export membrane protein SecD
393	cbdbA360	DET0408	DehaBAV1_0387	VS351	cation channel family protein	cation channel family protein	Ion transport protein	Ion transport protein
394	cbdbA1715	DET1619	DehaBAV1_1365	VS1501	ABC transporter, ATP-binding protein	ABC transporter, ATP-binding protein	ABC transporter related	ABC transporter related
395	cbdbA1716	DET1620	DehaBAV1_1366	VS1502	putative ABC-transporter, permease component	hypothetical protein	hypothetical protein	membrane protein, putative
396	cbdbA1289	DET1339	DehaBAV1_1150	VS1121	ABC transporter, ATP-binding protein	ABC transporter, ATP-binding protein	ABC transporter related	ABC transporter related
397	cbdbA1290	DET1340	DehaBAV1_1151	VS1122	ABC transporter, permease protein	ABC transporter, permease protein, putative	ABC-2 type transporter	ABC transporter, permease protein
398	cbdbA772	DET0793	DehaBAV1_0719	VS699	ABC transporter associated protein with signal peptide, putative periplasmic substrate binding subunit	hypothetical protein	hypothetical protein	ABC transporter associated protein with signal peptide, putative periplasmic substrate binding subunit
399	cbdbA773	DET0794	DehaBAV1_0720	VS700	ABC transporter, ATP-binding protein	ABC transporter, ATP-binding protein	ABC transporter related	ABC transporter related
400	cbdbA774	DET0795	DehaBAV1_0721	VS701	ABC transporter, permease protein	ABC transporter, permease protein	ABC-2 type transporter	ABC-2 type transporter
401	cbdbA791	DET0814	DehaBAV1_0734	VS718	ABC transporter, permease protein	ABC transporter, permease protein	ABC-2 type transporter	ABC-2 type transporter
402	cbdbA793	DET0815	DehaBAV1_0735	VS719	iron-regulated ABC transporter, atp-binding protein, SufC	FeS assembly ATPase SufC, putative	ABC transporter related	ABC transporter related
403	cbdbA794	DET0816	DehaBAV1_0736	VS720	iron-regulated ABC transporter, subunit SufB-related	sufB/sufD domain protein	SufBD protein	SufBD protein

Table 17. List of Exchange Reactions of *i* AI549

No	Abbreviation	Equation
1	EX_ac(e)	[e] : ac <==>
2	EX_antim(e)	[e] : antim <==>
3	EX_arsna(e)	[e] : arsna <==>
4	EX_arsni2(e)	[e] : arsni2 <==>
5	EX_cbl1(e)	[e] : cbl1 <==>
6	EX_cit(e)	[e] : cit <==>
7	EX_cl(e)	[e] : cl <==>
8	EX_co2(e)	[e] : co2 <==>
9	EX_cobalt2(e)	[e] : cobalt2 <==>
10	EX_cu2(e)	[e] : cu2 <==>
11	EX_dcb(e)	[e] : dcb <==>
12	EX_etl(e)	[e] : etl <==>
13	EX_fe2(e)	[e] : fe2 <==>
14	EX_gln-L(e)	[e] : gln-L <==>
15	EX_glyb(e)	[e] : glyb <==>
16	EX_h(e)	[e] : h <==>
17	EX_h2(e)	[e] : h2 <==>
18	EX_h2o(e)	[e] : h2o <==>
19	EX_hcb(e)	[e] : hcb <==>
20	EX_ile-L(e)	[e] : ile-L <==>
21	EX_k(e)	[e] : k <==>
22	EX_leu-L(e)	[e] : leu-L <==>
23	EX_mg2(e)	[e] : mg2 <==>
24	EX_mn2(e)	[e] : mn2 <==>
25	EX_mobd(e)	[e] : mobd <==>
26	EX_n2(e)	[e] : n2 <==>
27	EX_nh4(e)	[e] : nh4 <==>
28	EX_ni2(e)	[e] : ni2 <==>
29	EX_pce(e)	[e] : pce <==>
30	EX_pi(e)	[e] : pi <==>
31	EX_pro-L(e)	[e] : pro-L <==>
32	EX_so4(e)	[e] : so4 <==>
33	EX_tsul(e)	[e] : tsul <==>

No	Abbreviation	Equation
34	EX_val-L(e)	[e] : val-L <==>
35	EX_zn2(e)	[e] : zn2 <==>

Table 18. Set of Constraints for Simulating *Dehalococcoides* Growth using *i* AI549

Abbreviation	Equation	LB	UB	Flux (mmol/gDCW/h)	EC Number
AACPAT	[c] : ACP + accoa --> acACP + coa	0	Infinity	0.00	2.3.1.38
ABTA	[c] : 4abut + akg --> glu-L + sucsal	0	Infinity	0.00	2.6.1.19
ACBIPGT	[c] : adcobap + gtp + h --> adgcoba + ppi	0	Infinity	0.00	
ACCOAC	[c] : accoa + atp + hco3 --> adp + h + malcoa + pi	0	Infinity	0.02	6.4.1.2
ACGK	[c] : acglu + atp --> acg5p + adp	0	Infinity	0.01	2.7.2.8
ACGS	[c] : accoa + glu-L --> acglu + coa + h	0	Infinity	0.00	2.3.1.1
ACHBS	[c] : 2obut + h + pyr --> 2ahbut + co2	0	Infinity	0.00	
ACLS	[c] : h + (2) pyr --> alac-S + co2	0	Infinity	0.01	4.1.3.18
ACMAT1	[c] : acACP + h + malACP --> ACP + actACP + co2	0	Infinity	0.00	2.3.1.41
ACONT	[c] : cit <==> icit	-Infinity	Infinity	0.00	4.2.1.3
ACOTA	[c] : acorn + akg <==> acg5sa + glu-L	-Infinity	Infinity	-0.01	2.6.1.11
ACPS1	[c] : apoACP + coa --> ACP + h + pap	0	Infinity	0.00	2.7.8.7
ACS	[c] : ac + atp + coa --> accoa + amp + ppi	0	Infinity	0.19	6.2.1.1
ACt6	ac[e] + h[e] <==> ac[c] + h[c]	-Infinity	Infinity	0.18	
ADCL	[c] : 4adcho --> 4abz + h + pyr	0	Infinity	0.00	
ADCOBAK	[c] : adcoba + atp --> adcobap + adp + h	0	Infinity	0.00	
ADCOBAS	[c] : 1ap2ol + adcobhex --> adcoba + h2o	0	Infinity	0.00	
ADCOBHEXS	[c] : adcobdam + (4) atp + (4) gln-L + (4) h2o --> adcobhex + (4) adp + (4) glu-L + (4) h + (4) pi	0	Infinity	0.00	
ADCOBHS	[c] : 1ap2ol + adcobhex <==> adcoba + h2o	-Infinity	Infinity	0.00	3.5.1
ADCPS2	[c] : 1ap2olp + adcobhex + atp --> adcobap + adp + h + pi	0	Infinity	0.00	
ADCS	[c] : chor + gln-L --> 4adcho + glu-L	0	Infinity	0.00	
ADD	[c] : ade + h + h2o --> hxan + nh4	0	Infinity	0.00	3.5.4.2
ADK1	[c] : amp + atp <==> (2) adp	-Infinity	Infinity	0.26	2.7.4.3
ADK2	[c] : amp + pppi <==> adp + ppi	-Infinity	Infinity	0.00	2.7.4.3
ADK3	[c] : amp + gtp <==> adp + gdp	-Infinity	Infinity	0.00	2.7.4.8
ADK4	[c] : amp + itp <==> adp + idp	-Infinity	Infinity	0.00	
ADNCYC	[c] : atp --> camp + ppi	0	Infinity	0.00	4.6.1.1
ADNK1	[c] : adn + atp --> adp + amp + h	0	Infinity	0.00	2.7.1.20
ADPRDP	[c] : adprib + h2o --> amp + (2) h + r5p	0	Infinity	0.00	3.6.1.13
ADPT	[c] : ade + prpp --> amp + ppi	0	Infinity	0.00	2.4.2.7
ADSL1	[c] : dcamp --> amp + fum	0	Infinity	0.00	4.3.2.2
ADSL2	[c] : 25aics --> aicar + fum	0	Infinity	0.00	4.3.2.2
ADSS	[c] : asp-L + gtp + imp --> dcamp + gdp + (2) h + pi	0	Infinity	0.00	6.3.4.4
AGMT	[c] : agm + h2o --> ptrc + urea	0	Infinity	0.00	3.5.3.11
AGPR	[c] : acg5sa + nadp + pi <==> acg5p + h + nadph	-Infinity	Infinity	-0.01	1.2.1.38

Abbreviation	Equation	LB	UB	Flux (mmol/gDCW/h)	EC Number
AHC	[c] : ahcys + h2o <==> adn + hcys-L	-Infinity	Infinity	0.00	3.3.1.1
AHMMPS	[c] : air + h2o --> 4ahmmp + gcald + (0.5) o2 + pi	0	Infinity	0.00	
AICART	[c] : 10fthf + aicar <==> fprica + thf	-Infinity	Infinity	0.01	2.1.2.3
AIRC	[c] : air + co2 --> 5aizc + h	0	Infinity	0.00	4.1.1.21
ALAALA	[c] : (2) ala-D + atp <==> adp + alaala + h + pi	-Infinity	Infinity	0.00	6.3.2.4
ALAR	[c] : ala-L <==> ala-D	-Infinity	Infinity	0.00	5.1.1.1
ALATA_D2	[c] : ala-D + pydx5p --> pyam5p + pyr	0	Infinity	0.00	
ALATA_L	[c] : akg + ala-L <==> glu-L + pyr	-Infinity	Infinity	-0.01	2.6.1.2
ALATA_L2	[c] : ala-L + pydx5p --> pyam5p + pyr	0	Infinity	0.00	
ALATRS	[c] : ala-L + atp + trnaala --> alatrna + amp + ppi	0	Infinity	0.00	6.1.1.7
ALCD19	[c] : glyald + h + nadh <==> glyc + nad	-Infinity	Infinity	0.00	1.1.1.1
ALCD2x	[c] : etoh + nad <==> acald + h + nadh	-Infinity	Infinity	0.00	1.1.1.1
ALCD3	[c] : nad + ppoh <==> h + nadh + ppal	-Infinity	Infinity	0.00	1.1.1.1
ALDD8b	[c] : glyald + h2o + nad <==> glyc-R + (2) h + nadh	-Infinity	Infinity	0.00	1.2.1.3
AMAOT	[c] : 8aonn + amet <==> amob + dann	-Infinity	Infinity	0.00	2.6.1.62
AMPMS	[c] : air + h2o --> 4ampm + (2) for + (4) h	0	Infinity	0.00	
ANPRT	[c] : anth + prpp --> ppi + pran	0	Infinity	0.00	2.4.2.18
ANS1	[c] : chor + gln-L --> anth + glu-L + h + pyr	0	Infinity	0.00	4.1.3.27
ANTIMt1	antim[c] --> antim[e]	0	Infinity	0.00	
APRAUR	[c] : 5apru + h + nadph --> 5aprbu + nadp	0	Infinity	0.00	1.1.1.193
APSPT	[c] : aps + trdrd <==> amp + (2) h + so3 + trdox	-Infinity	Infinity	0.00	
APYRH	[c] : 3fpyr + h2o --> fum + h + pyr	0	Infinity	0.00	3.7.1.5
ARGDC	[c] : arg-L + h --> agm + co2	0	Infinity	0.00	4.1.1.19
ARGSL	[c] : argsuc <==> arg-L + fum	-Infinity	Infinity	0.00	4.3.2.1
ARGSS	[c] : asp-L + atp + citr-L --> amp + argsuc + h + ppi	0	Infinity	0.00	6.3.4.5
ARGTRS	[c] : arg-L + atp + trnaarg --> amp + argtrna + ppi	0	Infinity	0.00	6.1.1.19
ARSNAt1	arsna[c] --> arsna[e]	0	Infinity	0.00	
ARSt1	arsni2[c] --> arsni2[e]	0	Infinity	0.00	
ASAD	[c] : aspsa + nadp + pi <==> 4pasp + h + nadph	-Infinity	Infinity	-0.01	1.2.1.11
ASNS2	[c] : asp-L + atp + nh4 --> amp + asn-L + h + ppi	0	Infinity	0.00	6.3.1.1
ASP1DC	[c] : asp-L + h --> ala-B + co2	0	Infinity	0.00	4.1.1.11
ASPCT	[c] : asp-L + cbp --> cbasp + h + pi	0	Infinity	0.01	2.1.3.2
ASPK	[c] : asp-L + atp <==> 4pasp + adp	-Infinity	Infinity	0.01	2.7.2.4
ASPO1	[c] : asp-L + nad --> (2) h + iasp + nadh	0	Infinity	0.00	
ASPO3	[c] : asp-L + ubq8 --> h + iasp + ubq8h2	0	Infinity	0.00	
ASPO4	[c] : asp-L + mqn8 --> h + iasp + mql8	0	Infinity	0.00	
ASPO5	[c] : asp-L + fum --> h + iasp + succ	0	Infinity	0.00	
ASPO6	[c] : asp-L + o2 --> h + h2o2 + iasp	0	Infinity	0.00	

Abbreviation	Equation	LB	UB	Flux (mmol/gDCW/h)	EC Number
ASPT	[c] : asp-L --> fum + nh4	0	Infinity	0.00	4.3.1.1
ASPTA1	[c] : akg + asp-L <==> glu-L + oaa	-Infinity	Infinity	-0.04	2.6.1.1
ASPTRS	[c] : asp-L + atp + trnaasp --> amp + asptrna + ppi	0	Infinity	0.00	6.1.1.12
ATPM	[c] : atp + h2o --> adp + h + pi	1.8	1.8	1.80	
ATPPRT	[c] : atp + prpp --> ppi + prbatp	0	Infinity	0.00	2.4.2.17
ATPS3r	adp[c] + (3) h[e] + pi[c] <==> atp[c] + (2) h[c] + h2o[c]	-Infinity	Infinity	3.50	
BACCL	[c] : atp + btn + h --> btamp + ppi	0	Infinity	0.00	6.3.4.15
BTMAT1	[c] : 2beACP + h + nadh --> butACP + nad	0	Infinity	0.00	1.3.1.10
BTS	[c] : dtbt + (2) s --> btn + h + h2s	0	Infinity	0.00	2.8.1.6
CBIAT	[c] : atp + cbi + h2o <==> adcoba + pi + ppi	-Infinity	Infinity	0.00	2.5.1.17
CBL1abc	atp[c] + cbl1[e] + h2o[c] --> adp[c] + cbl1[c] + h[c] + pi[c]	0	Infinity	0.00	
CBLATr	[c] : atp + cbl1 + h <==> adocbl + pppi	-Infinity	Infinity	0.00	2.5.1.17
CBPSr	[c] : (2) atp + gln-L + h2o + hco3 <==> (2) adp + cbp + glu-L + (2) h + pi	-Infinity	Infinity	0.01	6.3.5.5
CDGPT	[c] : cdpdag + glyc3p --> cmp + h + pglyp	0	Infinity	0.00	
CDPDSP	[c] : cdpdag + ser-L --> cmp + h + ps	0	Infinity	0.00	2.7.8.8
CDPMEK	[c] : 4c2me + atp --> 2p4c2me + adp + h	0	Infinity	0.00	
CERD	(8) fdred[c] + (4) h[c] + pce[e] --> (4) cl[e] + etl[e] + (8) fdox[c]	0	Infinity	0.00	
CHORM	[c] : chor --> pphn	0	Infinity	0.00	5.4.99.5
CHORS	[c] : 3psme --> chor + pi	0	Infinity	0.01	4.2.3.5
CITRMS	[c] : accoa + h + h2o + pyr <==> citrmal + coa	-Infinity	Infinity	0.00	2.3.1
CITt6	cit[e] + h[e] <==> cit[c] + h[c]	-Infinity	Infinity	0.00	
CLPNS	[c] : cdpdag + pgly --> cdlp + cmp + h	0	Infinity	0.00	
CMLDC	[c] : 2c25dho + h --> 5odhf2a + co2	0	Infinity	0.00	4.1.1.44
CO2t	co2[e] <==> co2[c]	-Infinity	Infinity	0.17	
COBALTt5	cobalt2[c] <==> cobalt2[e]	-Infinity	Infinity	0.00	
COBPS	[c] : adgcoba + rdmbzi --> cobamcoa + gmp + h	0	Infinity	0.00	
COBPS2	[c] : adgcoba + rdmbzi <==> adocbl + gmp + h	-Infinity	Infinity	0.00	
CODHr	[c] : co + (2) fdox + h2o <==> co2 + (2) fdred + (2) h	-Infinity	Infinity	0.00	1.2.99.2
CPC3MT	[c] : amet + copre3 + h --> ahcys + copre4	0	Infinity	0.00	2.1.1.131
CPC6MT	[c] : (2) amet + codhpre6 --> (2) ahcys + co2 + copre8 + h	0	Infinity	0.00	2.1.1.132
CPPPGOAN2	[c] : (2) amet + cpppg3 + (4) fdox + (2) nadph --> (2) co2 + (2) dad-5 + (4) fdred + (2) h + (2) met-L + (2) nadp + pppg9	0	Infinity	0.00	
CTPS1	[c] : atp + nh4 + utp --> adp + ctp + (2) h + pi	0	Infinity	0.00	6.3.4.2

Abbreviation	Equation	LB	UB	Flux (mmol/gDCW/h)	EC Number
CTPS2	[c] : atp + gln-L + h2o + utp --> adp + ctp + glu-L + (2) h + pi	0	Infinity	0.00	6.3.4.2
Cuabc	atp[c] + cu2[e] + h2o[c] --> adp[c] + cu2[c] + h[c] + pi[c]	0	Infinity	0.00	
CYRDAAT	[c] : atp + co1dam + h --> adcobdam + pppi	0	Infinity	0.00	2.5.1.17
CYRDAS	[c] : (4) atp + (2) cobrnt + (4) gln-L + (2) h2o --> (4) adp + (2) co2dam + (4) glu-L + (2) h + (2) ppi	0	Infinity	0.00	
CYSS	[c] : acser + h2s --> ac + cys-L	0	Infinity	0.00	4.2.99.8
CYSTRS	[c] : atp + cys-L + trnacys --> amp + cystrna + ppi	0	Infinity	0.00	6.1.1.16
CYTK1	[c] : atp + cmp <==> adp + cdp	-Infinity	Infinity	0.00	2.7.4.14
CYTK2	[c] : atp + dcmp <==> adp + dcdp	-Infinity	Infinity	0.00	2.7.4.14
DADK	[c] : atp + damp <==> adp + dadp	-Infinity	Infinity	0.00	2.7.4.11
DAGK	[c] : 12dgr + atp --> 12dag3p + adp + h	0	Infinity	0.00	2.7.1.107
DAHPS	[c] : e4p + h2o + pep --> 2dda7p + pi	0	Infinity	0.01	4.1.2.15
DAPAT	[c] : 26dap-LL + akg <==> glu-L + h + h2o + thdp	-Infinity	Infinity	-0.01	
DAPDC	[c] : 26dap-M + h --> co2 + lys-L	0	Infinity	0.01	4.1.1.20
DAPE	[c] : 26dap-LL <==> 26dap-M	-Infinity	Infinity	0.01	5.1.1.7
DB4PS	[c] : ru5p-D --> db4p + for + h	0	Infinity	0.00	
DCMPDA2ir	[c] : dcmp + h + h2o --> dump + nh4	0	Infinity	0.00	3.5.4.12
DCMPDA3ir	[c] : cmp + h + h2o --> nh4 + ump	0	Infinity	0.00	3.5.4.12
DDMAT5	[c] : 2tddACP + h + nadh --> ddcaACP + nad	0	Infinity	0.00	1.3.1.10
DEMAT4	[c] : 2tdeACP + h + nadh --> decACP + nad	0	Infinity	0.00	1.3.1.10
DGK1	[c] : atp + dgmp <==> adp + dgdp	-Infinity	Infinity	0.00	
DGTPH	[c] : dgtp + h2o --> dgsn + pppi	0	Infinity	0.00	3.1.5.1
DHAD1	[c] : 23dhmb --> 3mob + h2o	0	Infinity	0.01	4.2.1.9
DHAD2	[c] : 23dhmp --> 3mop + h2o	0	Infinity	0.00	
DHAD3	[c] : 23dhmp --> 2o3mpt + h2o	0	Infinity	0.00	4.2.1.9
DHDPRy	[c] : 23dhdp + h + nadph --> nadp + thdp	0	Infinity	0.01	1.3.1.26
DHDPS	[c] : aspsa + pyr --> 23dhdp + h + (2) h2o	0	Infinity	0.01	4.2.1.52
DHFOR2	[c] : dhf + nadp <==> fol + nadph	-Infinity	Infinity	0.00	1.5.1.3
DHFOR3	[c] : fol + h + (2) nadph <==> (2) nadp + thf	-Infinity	Infinity	0.00	1.5.1.3
DHFR	[c] : dhf + h + nadph <==> nadp + thf	-Infinity	Infinity	0.00	1.5.1.3
DHFS	[c] : atp + dhpt + glu-L --> adp + dhf + h + pi	0	Infinity	0.00	6.3.2.12
DHNAOT	[c] : dhna + nad + octdp --> 2dmmq8 + co2 + nadh + ppi	0	Infinity	0.00	
DHNPA	[c] : dhnpt --> 2ahhmp + gcald	0	Infinity	0.00	4.1.2.25
DHORDf	[c] : dhor-S + (2) fdox <==> (2) fdred + (2) h + orot	-Infinity	Infinity	0.01	1.3.99.11
DHORTS	[c] : dhor-S + h2o <==> cbasp + h	-Infinity	Infinity	-0.01	3.5.2.3
DHPPDA2	[c] : 25dhpp + h + h2o --> 5apru + nh4	0	Infinity	0.00	3.5.4.26

Abbreviation	Equation	LB	UB	Flux (mmol/gDCW/h)	EC Number
DHPS1	[c] : 2ahhmp + 4abz --> dhpt + h2o	0	Infinity	0.00	2.5.1.15
DHPS3	[c] : 2ahhmd + 4abz --> dhpt + ppi	0	Infinity	0.00	2.5.1.15
DHQD	[c] : 3dhq <==> 3dhsk + h2o	-Infinity	Infinity	0.01	4.2.1.10
DHQS	[c] : 2dda7p --> 3dhq + pi	0	Infinity	0.01	4.2.3.4
DMATT	[c] : dmpp + ipdp --> grdp + ppi	0	Infinity	0.00	2.5.1.1
DMPPS	[c] : h + h2mb4p + nadh --> dmpp + h2o + nad	0	Infinity	0.00	
DMQMT	[c] : 2omhmbl + amet --> ahcys + h + ubq8h2	0	Infinity	0.00	
DNMPPA	[c] : dhpmp + h2o --> dhnpt + pi	0	Infinity	0.00	
DNTPPA	[c] : ahdt + h2o --> dhpmp + h + ppi	0	Infinity	0.00	
DPCOAK	[c] : atp + dpcoa --> adp + coa + h	0	Infinity	0.00	2.7.1.24
DPR	[c] : 2dhp + h + nadph --> nadp + pant-R	0	Infinity	0.00	1.1.1.169
DTMPK	[c] : atp + dtmp <==> adp + dtdp	-Infinity	Infinity	0.00	2.7.4.9
DUTPDP	[c] : dutp + h2o --> dump + h + ppi	0	Infinity	0.00	3.6.1.23
DXPRI	[c] : dxyl5p + h + nadph <==> 2me4p + nadp	-Infinity	Infinity	0.00	
DXPS	[c] : g3p + h + pyr --> co2 + dxyl5p	0	Infinity	0.00	
E4PD	[c] : e4p + h2o + nad <==> 4per + (2) h + nadh	-Infinity	Infinity	0.00	
ENO	[c] : 2pg <==> h2o + pep	-Infinity	Infinity	-0.03	4.2.1.11
F1PK	[c] : atp + f1p --> adp + fdp + h	0	Infinity	0.00	2.7.1.56
FA120ACPH	[c] : ddcaACP + h2o <==> ACP + ddca + h	-Infinity	Infinity	0.00	3.1.2.14
FA140ACPH	[c] : h2o + myrsACP <==> ACP + h + ttdca	-Infinity	Infinity	0.00	3.1.2.14
FA160ACPH	[c] : h2o + palmACP <==> ACP + h + hdca	-Infinity	Infinity	0.00	3.1.2.14
FA180ACPH	[c] : h2o + ocdACP <==> ACP + h + ocdca	-Infinity	Infinity	0.00	3.1.2.14
FA200ACPH	[c] : h2o + icsACP <==> ACP + h + icsa	-Infinity	Infinity	0.00	3.1.2.14
FACOAL120	[c] : atp + coa + ddca <==> amp + ddcoa + ppi	-Infinity	Infinity	0.00	6.2.1.3
FACOAL140	[c] : atp + coa + ttdca <==> amp + ppi + tdcoa	-Infinity	Infinity	0.00	6.2.1.3
FACOAL160	[c] : atp + coa + hdca <==> amp + pmtcoa + ppi	-Infinity	Infinity	0.00	6.2.1.3
FACOAL170(ISO)	[c] : atp + coa + fa11 <==> amp + fa11coa + ppi	-Infinity	Infinity	0.00	6.2.1.3
FACOAL180	[c] : atp + coa + ocdca <==> amp + ppi + strcoa	-Infinity	Infinity	0.00	6.2.1.3
FACOAL181	[c] : atp + coa + ocdcea <==> amp + odecoa + ppi	-Infinity	Infinity	0.00	6.2.1.3
FACOAL200	[c] : atp + coa + icsa <==> amp + icscoa + ppi	-Infinity	Infinity	0.00	6.2.1.3
FBA	[c] : fdp <==> dhap + g3p	-Infinity	Infinity	-0.01	4.1.2.13
FBP	[c] : fdp + h2o --> f6p + pi	0	Infinity	0.01	3.1.3.11
FCLPA	[c] : fc1p <==> dhap + lald-L	-Infinity	Infinity	0.00	4.1.2.17
FDTS	[c] : dump + fadh2 + mlthf --> dtmp + fad + thf	0	Infinity	0.00	
FE2abc	atp[c] + fe2[e] + h2o[c] --> adp[c] + fe2[c] + h[c] + pi[c]	0	Infinity	0.00	
FMETDF	[c] : fmet + h2o --> for + met-L	0	Infinity	0.00	3.5.1.31
FMETTRS	[c] : 10fthf + mettrna --> fmettrna + thf	0	Infinity	0.00	2.1.2.9
FMNAT	[c] : atp + fmn + h --> fad + ppi	0	Infinity	0.00	2.7.7.2

Abbreviation	Equation	LB	UB	Flux (mmol/gDCW/h)	EC Number
FNOR2	[c] : (2) fdred + h + nadp <==> (2) fdox + nadph	-Infinity	Infinity	0.23	1.18.1.2
FRD5	[c] : mqn7 + succ <==> fum + mql7	-Infinity	Infinity	-0.02	1.3.5.1
FRHD	(2) fdox[c] + h2[e] <==> (2) fdred[c] + (2) h[e]	-Infinity	Infinity	10	1.12.7
FRTT	[c] : frdp + ipdp --> ggdp + ppi	0	Infinity	0.00	2.5.1.29
FTHFD	[c] : 10fthf + h2o --> for + h + thf	0	Infinity	0.00	3.5.1.10
FTHFLr	[c] : atp + for + thf <==> 10fthf + adp + pi	-Infinity	Infinity	0.00	6.3.4.3
FUM	[c] : fum + h2o <==> mal-L	-Infinity	Infinity	0.00	4.2.1.2
G1PACT	[c] : accoa + gam1p --> acgam1p + coa + h	0	Infinity	0.00	2.3.1.157
G1PTMT	[c] : dttp + g1p + h --> dtdpglc + ppi	0	Infinity	0.00	2.7.7.24
G3PD1	[c] : glyc3p + nad <==> dhap + h + nadh	-Infinity	Infinity	0.04	1.1.1.94
G3PD2	[c] : glyc3p + nadp <==> dhap + h + nadph	-Infinity	Infinity	-0.04	1.1.1.94
G5SADs	[c] : glu5sa <==> 1pyr5c + h + h2o	-Infinity	Infinity	0.00	
G5SD	[c] : glu5p + h + nadph --> glu5sa + nadp + pi	0	Infinity	0.00	1.2.1.41
GALU	[c] : g1p + h + utp <==> ppi + udpg	-Infinity	Infinity	0.00	2.7.7.9
GAPD	[c] : g3p + nad + pi <==> 13dpg + h + nadh	-Infinity	Infinity	-0.03	1.2.1.12
GARFT	[c] : 10fthf + gar <==> fgam + h + thf	-Infinity	Infinity	0.00	2.1.2.2
GCCc	[c] : dhlpro + nad --> h + lpro + nadh	0	Infinity	0.00	1.8.1.4
GF6PTA	[c] : f6p + gln-L --> gam6p + glu-L	0	Infinity	0.00	2.6.1.16
GGTT	[c] : ggdp + ipdp --> pendp + ppi	0	Infinity	0.00	
GHMT	[c] : ser-L + thf <==> gly + h2o + mlthf	-Infinity	Infinity	0.01	2.1.2.1
GK1	[c] : atp + gmp <==> adp + gdp	-Infinity	Infinity	0.00	2.7.4.8
GLCS1	[c] : adpglc --> adp + glycogen + h	0	Infinity	0.00	2.4.1.21
GLGC	[c] : atp + g1p + h --> adpglc + ppi	0	Infinity	0.00	2.7.7.27
GLNabc	atp[c] + gln-L[e] + h2o[c] --> adp[c] + gln-L[c] + h[c] + pi[c]	0	Infinity	0.00	
GLNS	[c] : atp + glu-L + nh4 --> adp + gln-L + h + pi	0	Infinity	0.12	6.3.1.2
GLNTRS	[c] : atp + gln-L + trnagln --> amp + glntrna + ppi	0	Infinity	0.00	6.1.1.18
GLU5K	[c] : atp + glu-L --> adp + glu5p	0	Infinity	0.00	2.7.2.11
GLUADT	[c] : atp + gln-L + glutrna + h2o --> adp + glntrna + glu-L + h + pi	0	Infinity	0.00	
GLUPRT	[c] : gln-L + h2o + prpp --> glu-L + ppi + pram	0	Infinity	0.00	2.4.2.14
GLUSx	[c] : akg + gln-L + h + nadh --> (2) glu-L + nad	0	Infinity	0.00	1.4.1.14
GLUSy	[c] : akg + gln-L + h + nadph --> (2) glu-L + nadp	0	Infinity	0.10	1.4.1.13
GLUTRS	[c] : atp + glu-L + trnaglu --> amp + glutrna + ppi	0	Infinity	0.00	6.1.1.17
GLYBt6	glyb[e] + h[e] <==> glyb[c] + h[c]	-Infinity	Infinity	0.00	
GLYOX	[c] : h2o + lgt-S --> gthrd + h + lac-D	0	Infinity	0.00	3.1.2.6
GLYTRS	[c] : atp + gly + trnagly --> amp + glytrna + ppi	0	Infinity	0.00	6.1.1.14
GMPR	[c] : gmp + (2) h + nadph --> imp + nadp + nh4	0	Infinity	0.00	1.7.1.7
GMPS2	[c] : atp + gln-L + h2o + xmp --> amp + glu-L + gmp + (2) h + ppi	0	Infinity	0.00	6.3.5.2

Abbreviation	Equation	LB	UB	Flux (mmol/gDCW/h)	EC Number
GRTT	[c] : grdp + ipdp --> frdp + ppi	0	Infinity	0.00	2.5.1.10
GTPCI	[c] : gtp + h2o --> ahdt + for + h	0	Infinity	0.00	3.5.4.16
GTPCII	[c] : gtp + (3) h2o --> 25dhpp + for + (2) h + ppi	0	Infinity	0.00	3.5.4.25
GTPDK	[c] : atp + gtp --> amp + gdptp + h	0	Infinity	0.00	2.7.6.5
GUAPRT	[c] : gua + prpp --> gmp + ppi	0	Infinity	0.00	
H2Ot5	h2o[e] <==> h2o[c]	-Infinity	Infinity	-0.51	
H2td	h2[c] <==> h2[e]	-Infinity	Infinity	0.00	
HBUHL1	[c] : 3hbACP --> 2beACP + h2o	0	Infinity	0.00	4.2.1.58
HBUR1	[c] : actACP + h + nadph --> 3hbACP + nadp	0	Infinity	0.00	1.1.1.100
HCITS	[c] : accoa + akg + h2o --> coa + h + hcit	0	Infinity	0.00	4.1.3.21
HCO3E	[c] : co2 + h2o <==> h + hco3	-Infinity	Infinity	0.07	4.2.1.1
HDDHL5	[c] : 3hddACP --> 2tddACP + h2o	0	Infinity	0.00	4.2.1.58
HDDR5	[c] : 3oxddACP + h + nadph --> 3hddACP + nadp	0	Infinity	0.00	1.1.1.100
HDEHL4	[c] : 3hdeACP --> 2tdeACP + h2o	0	Infinity	0.00	4.2.1.58
HDER4	[c] : 3oxdeACP + h + nadph --> 3hdeACP + nadp	0	Infinity	0.00	1.1.1.100
HDMAT7	[c] : nad + palmACP <==> h + hdeACP + nadh	-Infinity	Infinity	0.00	1.3.1.10
HEMAT2	[c] : 2theACP + h + nadh --> hexACP + nad	0	Infinity	0.00	1.3.1.10
HEPTT	[c] : hepdp + ipdp --> octdp + ppi	0	Infinity	0.00	
HEX1	[c] : atp + glc-D --> adp + g6p + h	0	Infinity	0.00	2.7.1.2
HEXTT	[c] : hexdp + ipdp --> hepdp + ppi	0	Infinity	0.00	2.5.1.30
HGR	[c] : hg2 + nadph <==> h + hg0 + nadp	-Infinity	Infinity	0.00	1.16.1.1
HHDHL7	[c] : 3hpaACP --> h2o + hdeACP	0	Infinity	0.00	4.2.1.58
HHDR7	[c] : 3oxhdACP + h + nadph --> 3hpaACP + nadp	0	Infinity	0.00	1.1.1.100
HHYHL2	[c] : 3hhACP --> 2theACP + h2o	0	Infinity	0.00	4.2.1.58
HHYR2	[c] : 3oxhACP + h + nadph --> 3hhACP + nadp	0	Infinity	0.00	1.1.1.100
HICHL9	[c] : 3hicsacp --> 2ticsacp + h2o	0	Infinity	0.00	4.2.1.58
HICR9	[c] : 3oxicsacp + h + nadph --> 3hicsacp + nadp	0	Infinity	0.00	1.1.1.100
HISTD	[c] : h2o + histd + (2) nad --> (3) h + his-L + (2) nadh	0	Infinity	0.00	1.1.1.23
HISTP	[c] : h2o + hisp --> histd + pi	0	Infinity	0.00	3.1.3.15
HISTRS	[c] : atp + his-L + trnahis --> amp + histrna + ppi	0	Infinity	0.00	6.1.1.21
HMPK1	[c] : 4ahmmp + atp --> 4ampm + adp + h	0	Infinity	0.00	2.7.1.49
HMPK2	[c] : 4ahmmp + ctp --> 4ampm + cdp + h	0	Infinity	0.00	2.7.1.49
HMPK3	[c] : 4ahmmp + utp --> 4ampm + h + udp	0	Infinity	0.00	2.7.1.49
HMPK4	[c] : 4ahmmp + gtp --> 4ampm + gdp + h	0	Infinity	0.00	2.7.1.49
HOCHL3	[c] : 3hocACP --> 2toceACP + h2o	0	Infinity	0.00	4.2.1.58
HOCR3	[c] : 3oxocACP + h + nadph --> 3hocACP + nadp	0	Infinity	0.00	1.1.1.100
HODHL8n	[c] : 3hocdacp --> 2tocdACP + h2o	0	Infinity	0.00	4.2.1.58
HODR8_#1	[c] : 3oxocdacp + h + nadph --> 3hocdacp + nadp	0	Infinity	0.00	1.1.1.100
HPPK	[c] : 2ahhmp + atp --> 2ahhmd + amp + h	0	Infinity	0.00	2.7.6.3

Abbreviation	Equation	LB	UB	Flux (mmol/gDCW/h)	EC Number
HSAT	[c] : accoa + hom-L <==> achms + coa	-Infinity	Infinity	0.00	2.3.1.31
HSDy	[c] : hom-L + nadp <==> aspsa + h + nadph	-Infinity	Infinity	-0.01	1.1.1.3
HSK	[c] : atp + hom-L --> adp + h + phom	0	Infinity	0.00	2.7.1.39
HSPMS	[c] : (2) ptrc --> hspmd + nh4	0	Infinity	0.00	2.5.1.44
HSTPT	[c] : glu-L + imacp --> akg + h + hisp	0	Infinity	0.00	2.6.1.9
HTDHL6	[c] : 3htdACP --> h2o + tdeACP	0	Infinity	0.00	4.2.1.58
HTDR6	[c] : 3oxtdACP + h + nadph --> 3htdACP + nadp	0	Infinity	0.00	1.1.1.100
HXPRT	[c] : hxan + prpp --> imp + ppi	0	Infinity	0.00	2.4.2.8
ICDHy	[c] : icit + nadp <==> akg + co2 + nadph	-Infinity	Infinity	0.00	1.1.1.42
ICSMAT9	[c] : 2ticsacp + h + nadh --> icsACP + nad	0	Infinity	0.00	1.3.1.10
IG3PS	[c] : gln-L + prlp --> aicar + eig3p + glu-L + (2) h	0	Infinity	0.00	
IGPDH	[c] : eig3p + h --> h2o + imacp	0	Infinity	0.00	4.2.1.19
IGPS	[c] : 2cpr5p + h --> 3ig3p + co2 + h2o	0	Infinity	0.00	4.1.1.48
ILEabc	atp[c] + h2o[c] + ile-L[e] --> adp[c] + h[c] + ile-L[c] + pi[c]	0	Infinity	0.00	
ILETA	[c] : akg + ile-L <==> 3mop + glu-L	-Infinity	Infinity	0.00	2.6.1.42
ILETRS	[c] : atp + ile-L + trnaile --> amp + iletrna + ppi	0	Infinity	0.00	6.1.1.5
IMPCir	[c] : fprica --> h2o + imp	0	Infinity	0.01	3.5.4.10
IMPD	[c] : h2o + imp + nad --> h + nadh + xmp	0	Infinity	0.00	1.1.1.205
IOR	[c] : coa + (2) fdox + indpyr <==> co2 + (2) fdred + h + indaccoa	-Infinity	Infinity	0.00	
IOR2	[c] : coa + (2) fdox + phpyr <==> co2 + (2) fdred + h + phaccoa	-Infinity	Infinity	0.00	
IOR3	[c] : 34hpp + coa + (2) fdox <==> 4hphaccoa + co2 + (2) fdred + h	-Infinity	Infinity	0.00	
IPDPS	[c] : h + h2mb4p + nadh --> h2o + ipdp + nad	0	Infinity	0.00	
IPMD	[c] : 3c2hmp + nad --> 3c4mop + h + nadh	0	Infinity	0.01	1.1.1.85
IPMDH	[c] : de3mlt + nad <==> 2obut + co2 + (2) h + nadh	-Infinity	Infinity	0.00	
IPMI1	[c] : citrmal <==> ccnt + h2o	-Infinity	Infinity	0.00	4.2.1.35
IPMI2	[c] : ccnt + h2o <==> de3mlt	-Infinity	Infinity	0.00	
IPPMIa	[c] : 3c2hmp <==> 2ippm + h2o	-Infinity	Infinity	-0.01	4.2.1.33
IPPMIb	[c] : 2ippm + h2o <==> 3c3hmp	-Infinity	Infinity	-0.01	
IPPS	[c] : 3mob + accoa + h2o --> 3c3hmp + coa + h	0	Infinity	0.01	4.1.3.12
ITPASE	[c] : ditp + h2o --> dimp + ppi	0	Infinity	0.00	
KARA1	[c] : 23dhmb + nadp <==> alac-S + h + nadph	-Infinity	Infinity	-0.01	1.1.1.86
KARA2	[c] : 2ahbut + h + nadph <==> 23dhmp + nadp	-Infinity	Infinity	0.00	1.1.1.86
KAS11	[c] : (17) h + ivcoa + (6) malcoa + (12) nadph --> (6) co2 + (7) coa + fa11 + (5) h2o + (12) nadp	0	Infinity	0.00	2.3.1.41
KAS17	[c] : accoa + (22) h + (8) malcoa + (15) nadph --> (8) co2 + (9) coa + (7) h2o + (15) nadp + ocdcea	0	Infinity	0.00	2.3.1.41

Abbreviation	Equation	LB	UB	Flux (mmol/gDCW/h)	EC Number
Kt6	h[e] + k[e] <==> h[c] + k[c]	-Infinity	Infinity	0.00	
LDH_L	[c] : lac-L + nad <==> h + nadh + pyr	-Infinity	Infinity	0.00	1.1.1.27
LEUabc	atp[c] + h2o[c] + leu-L[e] --> adp[c] + h[c] + leu-L[c] + pi[c]	0	Infinity	0.00	
LEUTA	[c] : akg + leu-L <==> 4mop + glu-L	-Infinity	Infinity	-0.01	2.6.1.42
LEUTRS	[c] : atp + leu-L + trnaleu --> amp + leutrna + ppi	0	Infinity	0.00	6.1.1.4
LPADSS	[c] : lipidX + u23ga --> h + lipidAds + udp	0	Infinity	0.00	2.4.1.182
LYSAM	[c] : lys-L <==> 36dahx	-Infinity	Infinity	0.00	5.4.3.2
LYSTRS	[c] : atp + lys-L + trnalys --> amp + lystrna + ppi	0	Infinity	0.00	6.1.1.6
MACPMT	[c] : ACP + malcoa --> coa + malACP	0	Infinity	0.01	2.3.1.39
MAN1PT1	[c] : gtp + h + man1p --> gdpman + ppi	0	Infinity	0.00	2.7.7.13
MAN1PT2	[c] : gdp + h + man1p --> gdpman + pi	0	Infinity	0.00	2.7.7.22
MAN6PI	[c] : man6p <==> f6p	-Infinity	Infinity	0.00	5.3.1.8
MCMAT2	[c] : butACP + h + malACP --> 3oxhACP + ACP + co2	0	Infinity	0.00	2.3.1.41
MCMAT3	[c] : h + hexACP + malACP --> 3oxocACP + ACP + co2	0	Infinity	0.00	2.3.1.41
MCMAT4	[c] : h + malACP + octACP --> 3oxdeACP + ACP + co2	0	Infinity	0.00	2.3.1.41
MCMAT5	[c] : decACP + h + malACP --> 3oxddACP + ACP + co2	0	Infinity	0.00	2.3.1.41
MCMAT6	[c] : ddcaACP + h + malACP --> 3oxtdACP + ACP + co2	0	Infinity	0.00	2.3.1.41
MCMAT7	[c] : h + malACP + myrsACP --> 3oxhdACP + ACP + co2	0	Infinity	0.00	2.3.1.41
MCMAT8	[c] : h + malACP + palmACP --> 3oxocdacp + ACP + co2	0	Infinity	0.00	2.3.1.41
MCMAT9	[c] : h + malACP + ocdACP --> 3oxicsacp + ACP + co2	0	Infinity	0.00	2.3.1.41
MDH	[c] : mal-L + nad <==> h + nadh + oaa	-Infinity	Infinity	0.00	1.1.1.37
MECDPDH	[c] : 2mecdp + nadh --> h2mb4p + h2o + nad	0	Infinity	0.00	
MECDPS	[c] : 2p4c2me --> 2mecdp + cmp	0	Infinity	0.00	
MEPCT	[c] : 2me4p + ctp + h --> 4c2me + ppi	0	Infinity	0.00	
METACH	[c] : achms + h2s <==> ac + hcys-L	-Infinity	Infinity	0.00	4.2.99.10
METAT	[c] : atp + h2o + met-L --> amet + pi + ppi	0	Infinity	0.00	2.5.1.6
METS	[c] : 5mthf + hcys-L --> h + met-L + thf	0	Infinity	0.00	2.1.1.13
METSR-S1	[c] : metox + trdrd --> h2o + met-L + trdox	0	Infinity	0.00	
METSR-S2	[c] : h2o2 + met-L --> h2o + metox	0	Infinity	0.00	
METTRS	[c] : atp + met-L + trnamet --> amp + mettrna + ppi	0	Infinity	0.00	6.1.1.10
MGSA	[c] : dhap --> mthgxl + pi	0	Infinity	0.00	4.2.3.3

Abbreviation	Equation	LB	UB	Flux (mmol/gDCW/h)	EC Number
MGt5	mg2[c] <==> mg2[e]	-Infinity	Infinity	0.00	
MICITH	[c] : 2mcacn + h2o --> micit	0	Infinity	0.00	4.2.1.99
MNabc	atp[c] + h2o[c] + mn2[e] --> adp[c] + h[c] + mn2[c] + pi[c]	0	Infinity	0.00	
MOBDabc	atp[c] + h2o[c] + mobd[e] --> adp[c] + h[c] + mobd[c] + pi[c]	0	Infinity	0.00	
MOHMT	[c] : 3mob + h2o + mlthf --> 2dhp + thf	0	Infinity	0.00	2.1.2.11
MPGSP	[c] : 3pg + gdpman + (2) h <==> 2m3pg + gdp	-Infinity	Infinity	0.00	2.4.1.217
MTAP	[c] : 5mta + pi --> 5mdr1p + ade	0	Infinity	0.00	2.4.2.28
MTHFC	[c] : h2o + methf <==> 10fthf + h	-Infinity	Infinity	0.01	3.5.4.9
MTHFD	[c] : mlthf + nadp <==> methf + nadph	-Infinity	Infinity	0.01	1.5.1.5
MTHFR1	[c] : (2) h + mlthf + nadph --> 5mthf + nadp	0	Infinity	0.00	1.7.99.5
MTRI	[c] : 5mdr1p <==> 5mdru1p	-Infinity	Infinity	0.00	5.3.1.23
N2t	n2[c] <==> n2[e]	-Infinity	Infinity	0.00	
NADH4	[c] : h + mqn7 + nadh --> mql7 + nad	0	Infinity	0.02	1.6.5.3
NADH6	(4.5) h[c] + nadh[c] + ubq8[c] --> (3.5) h[e] + nad[c] + ubq8h2[c]	0	Infinity	0.00	1.6.5.3
NADH7	(3) h[c] + mqn8[c] + nadh[c] --> (2) h[e] + mql8[c] + nad[c]	0	Infinity	0.00	1.6.5.3
NADH8	2dmmq8[c] + (3.8) h[c] + nadh[c] --> 2dmmql8[c] + (2.8) h[e] + nad[c]	0	Infinity	0.00	1.6.5.3
NADK	[c] : atp + nad --> adp + h + nadp	0	Infinity	0.00	2.7.1.23
NADPH12	[c] : h + nadph + ubq8 --> nadp + ubq8h2	0	Infinity	0.00	1.6.5.5
NADS1	[c] : atp + dnad + nh4 --> amp + h + nad + ppi	0	Infinity	0.00	6.3.1.5
NADS2	[c] : atp + dnad + gln-L + h2o --> amp + glu-L + h + nad + ppi	0	Infinity	0.00	6.3.5.1
NAMNPP	[c] : atp + h2o + nac + prpp --> adp + nicrnt + pi + ppi	0	Infinity	0.00	
NCTPPRT	[c] : nicrnt + ppi <==> h + nac + prpp	-Infinity	Infinity	0.00	2.4.2.11
NDPK1	[c] : atp + gdp <==> adp + gtp	-Infinity	Infinity	0.01	2.7.4.6
NDPK2	[c] : atp + udp <==> adp + utp	-Infinity	Infinity	0.01	2.7.4.6
NDPK3	[c] : atp + cdp <==> adp + ctp	-Infinity	Infinity	0.00	2.7.4.6
NDPK4	[c] : atp + dtdp <==> adp + dttp	-Infinity	Infinity	0.00	2.7.4.6
NDPK5	[c] : atp + dgdp <==> adp + dgtp	-Infinity	Infinity	0.00	2.7.4.6
NDPK6	[c] : atp + dudp <==> adp + dutp	-Infinity	Infinity	0.00	2.7.4.6
NDPK7	[c] : atp + dcdp <==> adp + dctp	-Infinity	Infinity	0.00	2.7.4.6
NDPK8	[c] : atp + dadp <==> adp + datp	-Infinity	Infinity	0.00	2.7.4.6
NH4t	nh4[e] <==> nh4[c]	-Infinity	Infinity	0.15	
NH4t3	k[c] + nh4[e] --> k[e] + nh4[c]	0	Infinity	0.00	

Abbreviation	Equation	LB	UB	Flux (mmol/gDCW/h)	EC Number
NIabc	atp[c] + h2o[c] + ni2[e] --> adp[c] + h[c] + ni2[c] + pi[c]	0	Infinity	0.00	
NITr	[c] : (16) atp + (8) fdred + (16) h2o + n2 <==> (16) adp + (8) fdox + (6) h + h2 + (2) nh4 + (16) pi	-Infinity	Infinity	0.00	1.18.6.1
NMNATr	[c] : atp + h + nmn <==> nad + ppi	-Infinity	Infinity	0.00	2.7.7.1
NNATr	[c] : atp + h + nicrnt <==> dnad + ppi	-Infinity	Infinity	0.00	2.7.7.18
NNDMBRT	[c] : dmbzid + nicrnt --> 5prdmbz + h + nac	0	Infinity	0.00	2.4.2.21
NNDMBRT3	[c] : ribflv --> dmbzid	0	Infinity	0.00	
NNDPR	[c] : (2) h + prpp + quln --> co2 + nicrnt + ppi	0	Infinity	0.00	2.4.2.19
NTPP1	[c] : dgtp + h2o --> dgmp + h + ppi	0	Infinity	0.00	
NTPP2	[c] : gtp + h2o --> gmp + h + ppi	0	Infinity	0.00	
NTPP3	[c] : dctp + h2o --> dcmp + h + ppi	0	Infinity	0.00	3.6.1.12
NTPP4	[c] : ctp + h2o --> cmp + h + ppi	0	Infinity	0.00	
NTPP5	[c] : datp + h2o --> damp + h + ppi	0	Infinity	0.00	
NTPP6	[c] : atp + h2o --> amp + h + ppi	0	Infinity	0.00	
NTPP7	[c] : dttp + h2o --> dtmp + h + ppi	0	Infinity	0.00	
NTPP8	[c] : h2o + utp --> h + ppi + ump	0	Infinity	0.00	
NTPTP2	[c] : gtp + h2o --> gsn + pppi	0	Infinity	0.00	3.1.5.1
NTRIRx	[c] : (4) h + (3) nadh + no2 --> h2o + (3) nad + nh4oh	0	Infinity	0.00	1.7.1.4
NTRIRy	[c] : (4) h + (3) nadph + no2 --> h2o + (3) nadp + nh4oh	0	Infinity	0.00	1.7.1.4
OBTFL	[c] : 2obut + coa --> for + ppcoa	0	Infinity	0.00	
OCBT	[c] : cbp + orn-L <==> citr-L + h + pi	-Infinity	Infinity	0.00	2.1.3.3
OCDMAT8	[c] : 2tocdACP + h + nadh --> nad + ocdACP	0	Infinity	0.00	1.3.1.10
OCMAT3	[c] : 2toceACP + h + nadh --> nad + octACP	0	Infinity	0.00	1.3.1.10
OCTT	[c] : ipdp + octdp --> nondp + ppi	0	Infinity	0.00	2.5.1.11
OHPHM	[c] : 2ohph + amet --> 2omph + ahcys + h	0	Infinity	0.00	
OIVD1	[c] : 4mop + coa + nad <==> co2 + ivcoa + nadh	-Infinity	Infinity	0.00	1.2.1.25
OIVD2	[c] : 3mob + coa + nad --> co2 + ibcoa + nadh	0	Infinity	0.00	1.2.1.25
OIVD3	[c] : 3mop + coa + nad --> 2mbcoa + co2 + nadh	0	Infinity	0.00	1.2.1.25
OMBZLM	[c] : 2ombzl + amet --> 2ommbl + ahcys + h	0	Infinity	0.00	
OMCDC	[c] : 3c4mop + h --> 4mop + co2	0	Infinity	0.01	
OMPDC	[c] : h + orot5p --> co2 + ump	0	Infinity	0.01	4.1.1.23
OOR2r	[c] : akg + coa + (2) fdox <==> co2 + (2) fdred + h + succoa	-Infinity	Infinity	-0.02	1.2.7.3
ORNCD	[c] : orn-L --> nh4 + pro-L	0	Infinity	0.00	4.3.1.12
ORNDC	[c] : h + orn-L --> co2 + ptrc	0	Infinity	0.00	4.1.1.17
ORNTA	[c] : akg + orn-L --> glu-L + glu5sa	0	Infinity	0.00	2.6.1.13

Abbreviation	Equation	LB	UB	Flux (mmol/gDCW/h)	EC Number
ORNTAC	[c] : acorn + glu-L <==> acglu + orn-L	-Infinity	Infinity	0.01	2.3.1.35
ORPT	[c] : orot5p + ppi <==> orot + prpp	-Infinity	Infinity	-0.01	2.4.2.10
OXAD2	[c] : h + oaa --> co2 + pyr	0	Infinity	0.00	4.1.1.3
P5CR	[c] : 1pyr5c + (2) h + nadph --> nadp + pro-L	0	Infinity	0.00	1.5.1.2
PACCOAL	[c] : atp + coa + pheac --> amp + phaccoa + ppi	0	Infinity	0.00	6.2.1.30
PANTS	[c] : ala-B + atp + pant-R --> amp + h + pnto-R + ppi	0	Infinity	0.00	6.3.2.1
PC	[c] : atp + hco3 + pyr --> adp + h + oaa + pi	0	Infinity	0.04	6.4.1.1
PDH	[c] : coa + nad + pyr --> accoa + co2 + nadh	0	Infinity	0.00	1.2.1.51
PDX5PO	[c] : o2 + pdx5p <==> h + h2o2 + pydx5p	-Infinity	Infinity	0.00	1.4.3.5
PDX5PS	[c] : dxyl5p + nad + phthr --> co2 + h + (2) h2o + nadh + pdx5p + pi	0	Infinity	0.00	
PFK	[c] : atp + f6p --> adp + fdp + h	0	Infinity	0.00	2.7.1.11
PFK_2	[c] : atp + tag6p-D --> adp + h + tagdp-D	0	Infinity	0.00	2.7.1.11
PFLi	[c] : coa + pyr --> accoa + for	0	Infinity	0.00	
PGAMT	[c] : gam1p <==> gam6p	-Infinity	Infinity	0.00	5.4.2.10
PGCD	[c] : 3pg + nad --> 3php + h + nadh	0	Infinity	0.00	1.1.1.95
PGCDr	[c] : 3pg + nad <==> 3php + h + nadh	-Infinity	Infinity	0.00	1.1.1.95
PGI	[c] : g6p <==> f6p	-Infinity	Infinity	0.00	5.3.1.9
PGK	[c] : 13dpg + adp <==> 3pg + atp	-Infinity	Infinity	-0.03	2.7.2.3
PGM	[c] : 3pg <==> 2pg	-Infinity	Infinity	-0.03	5.4.2.1
PGMT	[c] : g1p <==> g6p	-Infinity	Infinity	0.00	5.4.2.2
PGPPH	[c] : h2o + pglyp --> pgly + pi	0	Infinity	0.00	3.1.3.27
PHCYT	[c] : 12dag3p + ctp + h --> cdpdag + ppi	0	Infinity	0.00	2.7.7.41
PHETA1	[c] : akg + phe-L <==> glu-L + phpyr	-Infinity	Infinity	0.00	2.6.1.58
PHETRS	[c] : atp + phe-L + trnaphe --> amp + phetrna + ppi	0	Infinity	0.00	6.1.1.20
PIabc	atp[c] + h2o[c] + pi[e] --> adp[c] + h[c] + (2) pi[c]	0	Infinity	0.00	
PIt6	h[e] + pi[e] <==> h[c] + pi[c]	-Infinity	Infinity	0.03	
PMANM	[c] : man1p <==> man6p	-Infinity	Infinity	0.00	5.4.2.8
PMDPHT	[c] : 5aprbu + h2o --> 4r5au + pi	0	Infinity	0.00	
PMPK	[c] : 4ampm + atp --> 2mahmp + adp	0	Infinity	0.00	2.7.4.7
PNTEH	[c] : h2o + ptth --> cysam + pnto-R	0	Infinity	0.00	
PNTK	[c] : atp + pnto-R --> 4ppan + adp + h	0	Infinity	0.00	2.7.1.33
POR	[c] : coa + (2) fdox + pyr <==> accoa + co2 + (2) fdred + h	-Infinity	Infinity	-0.15	1.2.7.1
PPA	[c] : h2o + ppi --> h + (2) pi	0	Infinity	0.00	3.6.1.1
PPA_1	h2o[c] + ppi[c] --> h[e] + (2) pi[c]	0	Infinity	0.26	3.6.1.1
PPCDC	[c] : 4ppcys + h --> co2 + pan4p	0	Infinity	0.00	4.1.1.36
PPCOAC	[c] : atp + hco3 + ppcoa --> adp + h + mmcoa-S + pi	0	Infinity	0.00	6.4.1.3

Abbreviation	Equation	LB	UB	Flux (mmol/gDCW/h)	EC Number
PPDK	[c] : atp + pi + pyr --> amp + h + pep + ppi	0	Infinity	0.04	2.7.9.1
PPNCL	[c] : 4ppan + ctp + cys-L --> 4ppcys + cdp + h + pi	0	Infinity	0.00	6.3.2.5
PPND	[c] : nad + pphn --> 34hpp + co2 + nadh	0	Infinity	0.00	1.3.1.12
PPNDH	[c] : h + pphn --> co2 + h2o + phpyr	0	Infinity	0.00	4.2.1.51
PPS	[c] : atp + h2o + pyr --> amp + (2) h + pep + pi	0	Infinity	0.00	2.7.9.2
PPTT	[c] : ipdp + pendp --> hexdp + ppi	0	Infinity	0.00	2.5.1.33
PRAGS	[c] : atp + gly + pram --> adp + gar + h + pi	0	Infinity	0.00	6.3.4.13
PRAI	[c] : pran <==> 2cpr5p	-Infinity	Infinity	0.00	5.3.1.24
PRAIS	[c] : atp + fpram --> adp + air + h + pi	0	Infinity	0.00	6.3.3.1
PRAMPC	[c] : h + h2o + prbamp --> prfp	0	Infinity	0.00	3.5.4.19
PRASCS	[c] : 5aizc + asp-L + atp <==> 25aics + adp + h + pi	-Infinity	Infinity	0.00	6.3.2.6
PRATPP	[c] : h2o + prbatp --> h + ppi + prbamp	0	Infinity	0.00	3.6.1.31
PRFGS	[c] : atp + fgam + gln-L + h2o --> adp + fpram + glu-L + (2) h + pi	0	Infinity	0.00	6.3.5.3
PRMICI	[c] : prfp <==> prlp	-Infinity	Infinity	0.00	5.3.1.16
PROt6	h[e] + pro-L[e] <==> h[c] + pro-L[c]	-Infinity	Infinity	0.00	
PROTRS	[c] : atp + pro-L + trnapro --> amp + ppi + protrna	0	Infinity	0.00	6.1.1.15
PRPPS	[c] : atp + r5p <==> amp + h + prpp	-Infinity	Infinity	0.01	2.7.6.1
PSCVT	[c] : pep + skm5p <==> 3psme + pi	-Infinity	Infinity	0.01	2.5.1.19
PSERTr	[c] : 3php + glu-L <==> akg + pser-L	-Infinity	Infinity	0.00	2.6.1.52
PSP_L	[c] : h2o + pser-L --> pi + ser-L	0	Infinity	0.00	3.1.3.3
PSUDS	[c] : r5p + ura --> h2o + psd5p	0	Infinity	0.00	4.2.1.70
PTHPS	[c] : ahdt --> 6pthp + pppi	0	Infinity	0.00	4.2.3.12
PTPAT	[c] : atp + h + pan4p <==> dpcoa + ppi	-Infinity	Infinity	0.00	2.7.7.3
PYAM5PO	[c] : h2o + o2 + pyam5p --> h2o2 + nh4 + pydx5p	0	Infinity	0.00	1.4.3.5
PYK	[c] : adp + h + pep --> atp + pyr	0	Infinity	0.00	2.7.1.40
QULNS	[c] : dhap + iasp --> (2) h2o + pi + quln	0	Infinity	0.00	
RBFK	[c] : atp + ribflv --> adp + fmn + h	0	Infinity	0.00	2.7.1.26
RBFSa	[c] : 4r5au + db4p --> dmlz + (2) h2o + pi	0	Infinity	0.00	2.5.1.9
RBFSb	[c] : (2) dmlz --> 4r5au + ribflv	0	Infinity	0.00	2.5.1.9
RBK	[c] : atp + rib-D --> adp + h + r5p	0	Infinity	0.00	2.7.1.15
RBZP	[c] : 5prdmbz + h2o --> pi + rdmbzi	0	Infinity	0.00	
RDR1	(8) fdred[c] + (4) h[c] + hcb[e] --> (4) cl[e] + dcb[e] + (8) fdox[c]	0	Infinity	2.40	
RNDR1	[c] : adp + trdrd --> dadp + h2o + trdox	0	Infinity	0.00	1.17.4.1
RNDR2	[c] : gdp + trdrd --> dgdp + h2o + trdox	0	Infinity	0.00	1.17.4.1
RNDR3	[c] : cdp + trdrd --> dcdp + h2o + trdox	0	Infinity	0.00	1.17.4.1
RNDR4	[c] : trdrd + udp --> dudp + h2o + trdox	0	Infinity	0.00	1.17.4.1

Abbreviation	Equation	LB	UB	Flux (mmol/gDCW/h)	EC Number
RNTR1	[c] : atp + trdrd --> datp + h2o + trdox	0	Infinity	0.00	1.17.4.2
RNTR2	[c] : gtp + trdrd --> dgtp + h2o + trdox	0	Infinity	0.00	1.17.4.2
RNTR3	[c] : ctp + trdrd --> dctp + h2o + trdox	0	Infinity	0.00	1.17.4.2
RNTR4	[c] : trdrd + utp --> dutp + h2o + trdox	0	Infinity	0.00	1.17.4.2
RPE	[c] : ru5p-D <==> xu5p-D	-Infinity	Infinity	-0.01	5.1.3.1
RPI	[c] : r5p <==> ru5p-D	-Infinity	Infinity	-0.01	5.3.1.6
S7PI	[c] : s7p <==> gmh7p	-Infinity	Infinity	0.00	
SACCD2	[c] : Lsacchrp + h2o + nad <==> akg + h + lys-L + nadh	-Infinity	Infinity	0.00	1.5.1.7
SADT2	[c] : atp + gtp + h2o + so4 --> aps + gdp + pi + ppi	0	Infinity	0.00	2.7.7.4
SDPDS	[c] : h2o + sl26da --> 26dap-LL + succ	0	Infinity	0.00	3.5.1.18
SDPTA	[c] : akg + sl26da <==> glu-L + sl2a6o	-Infinity	Infinity	0.00	2.6.1.17
SERAT	[c] : accoa + ser-L <==> acser + coa	-Infinity	Infinity	0.00	2.3.1.30
SERD_Lr	[c] : ser-L <==> nh4 + pyr	-Infinity	Infinity	-0.02	4.3.1.17
SERTRS	[c] : atp + ser-L + trnaser --> amp + ppi + sertrna	0	Infinity	0.00	6.1.1.11
SHK3D	[c] : 3dhsk + h + nadph <==> nadp + skm	-Infinity	Infinity	0.01	1.1.1.25
SHKK	[c] : atp + skm --> adp + h + skm5p	0	Infinity	0.01	2.7.1.71
SOD	[c] : (2) h + (2) o2- --> h2o2 + o2	0	Infinity	0.00	1.15.1.1
SUCOAS	[c] : atp + coa + succ <==> adp + pi + succoa	-Infinity	Infinity	0.02	6.2.1.5
SULabc	atp[c] + h2o[c] + so4[e] --> adp[c] + h[c] + pi[c] + so4[c]	0	Infinity	0.00	
SULR	[c] : (3) h2o + h2s + (3) nadp <==> (4) h + (3) nadph + so3	-Infinity	Infinity	0.00	1.8.1.2
TAL	[c] : g3p + s7p <==> e4p + f6p	-Infinity	Infinity	0.00	2.2.1.2
TDMAT6	[c] : myrsACP + nad <==> h + nadh + tdeACP	-Infinity	Infinity	0.00	1.3.1.10
TGBPA	[c] : tagdp-D <==> dhap + g3p	-Infinity	Infinity	0.00	4.1.2.40
THDPS	[c] : h2o + succoa + thdp --> coa + sl2a6o	0	Infinity	0.00	2.3.1.117
THRA	[c] : thr-L <==> acald + gly	-Infinity	Infinity	0.00	4.1.2.5
THRD_L	[c] : thr-L --> 2obut + nh4	0	Infinity	0.00	
THRLAD	[c] : thr-LA --> acald + gly	0	Infinity	0.00	
THRPD	[c] : h + thrp --> 1ap2olp + co2	0	Infinity	0.00	
THRS	[c] : h2o + phom --> pi + thr-L	0	Infinity	0.00	4.2.3.1
THRTRS	[c] : atp + thr-L + trnathr --> amp + ppi + thrtrna	0	Infinity	0.00	6.1.1.3
THZPSN	[c] : atp + cys-L + dxyl5p + tyr-L --> 4hba + 4mpetz + ala-L + amp + co2 + h + h2o + ppi	0	Infinity	0.00	
TKT1	[c] : r5p + xu5p-D <==> g3p + s7p	-Infinity	Infinity	0.00	2.2.1.1
TKT2	[c] : e4p + xu5p-D <==> f6p + g3p	-Infinity	Infinity	-0.01	2.2.1.1
TMDK1	[c] : atp + thymd --> adp + dtmp + h	0	Infinity	0.00	2.7.1.21
TMDS	[c] : dump + mlthf --> dhf + dtmp	0	Infinity	0.00	2.1.1.45
TMPKr	[c] : atp + thmmp <==> adp + thmpp	-Infinity	Infinity	0.00	2.7.4.16

Abbreviation	Equation	LB	UB	Flux (mmol/gDCW/h)	EC Number
TMPPP	[c] : 2mahmp + 4mpetz + h --> ppi + thmmp	0	Infinity	0.00	2.5.1.3
TPI	[c] : dhap <==> g3p	-Infinity	Infinity	-0.01	5.3.1.1
TRDR	[c] : h + nadph + trdox --> nadp + trdrd	0	Infinity	0.01	1.8.1.9
TRPS1	[c] : 3ig3p + ser-L --> g3p + h2o + trp-L	0	Infinity	0.00	4.2.1.20
TRPTRS	[c] : atp + trnatrp + trp-L --> amp + ppi + trptrna	0	Infinity	0.00	6.1.1.2
TSULabc	atp[c] + h2o[c] + tsul[e] --> adp[c] + h[c] + pi[c] + tsul[c]	0	Infinity	0.00	
TYRTA	[c] : akg + tyr-L <==> 34hpp + glu-L	-Infinity	Infinity	0.00	2.6.1.5
TYRTRS	[c] : atp + trnatyr + tyr-L --> amp + ppi + tyrtrna	0	Infinity	0.00	6.1.1.1
UAGCVT	[c] : pep + uacgam --> pi + uaccg	0	Infinity	0.00	2.5.1.7
UAGDP	[c] : acgam1p + h + utp --> ppi + uacgam	0	Infinity	0.00	2.7.7.23
UAPGR	[c] : h + nadph + uaccg --> nadp + uamr	0	Infinity	0.00	1.1.1.158
UDPDPS	[c] : decdp + ipdp --> ppi + udcpdp	0	Infinity	0.00	2.5.1.31
UDPG4E	[c] : udpg <==> udpgal	-Infinity	Infinity	0.00	5.1.3.2
UMPK	[c] : atp + ump <==> adp + udp	-Infinity	Infinity	0.01	
UPP3MT	[c] : (2) amet + uppg3 --> (2) ahcys + (2) h + shcl	0	Infinity	0.00	2.1.1.107
UPP3S	[c] : hmbil --> h2o + uppg3	0	Infinity	0.00	4.2.1.75
UPPRT	[c] : prpp + ura --> ppi + ump	0	Infinity	0.00	2.4.2.9
URIDK1	[c] : atp + ump --> adp + udp	0	Infinity	0.00	
URIDK2	[c] : atp + dump --> adp + dudp	0	Infinity	0.00	
VALabc	atp[c] + h2o[c] + val-L[e] --> adp[c] + h[c] + pi[c] + val-L[c]	0	Infinity	0.00	
VALTA	[c] : akg + val-L <==> 3mob + glu-L	-Infinity	Infinity	-0.01	2.6.1.42
VALTRS	[c] : atp + trnaval + val-L --> amp + ppi + valtrna	0	Infinity	0.00	6.1.1.9
X00005	[c] : coa + gcald --> accoa + h2o	0	Infinity	0.00	
XTPASE	[c] : h2o + xtp --> h + ppi + xmp	0	Infinity	0.00	
XYLI2i	[c] : fru --> glc-D	0	Infinity	0.00	5.3.1.5
ZN2abc2	atp[c] + h2o[c] + zn2[e] --> adp[c] + h[c] + pi[c] + zn2[c]	0	Infinity	0.00	
EX_ac(e)	[e] : ac <==>	-5	0	-0.18	
EX_antim(e)	[e] : antim <==>	0	0	0.00	
EX_arsna(e)	[e] : arsna <==>	0	0	0.00	
EX_arsni2(e)	[e] : arsni2 <==>	0	0	0.00	
EX_cbl1(e)	[e] : cbl1 <==>	-Infinity	Infinity	0.00	
EX_cit(e)	[e] : cit <==>	0	0	0.00	
EX_cl(e)	[e] : cl <==>	0	Infinity	9.61	
EX_co2(e)	[e] : co2 <==>	-Infinity	Infinity	-0.17	
EX_cobalt2(e)	[e] : cobalt2 <==>	0	0	0.00	
EX_cu2(e)	[e] : cu2 <==>	0	0	0.00	
EX_dcb(e)	[e] : dcb <==>	0	Infinity	2.40	

Abbreviation	Equation	LB	UB	Flux (mmol/gDCW/h)	EC Number
EX_etl(e)	[e] : etl <==>	0	Infinity	0.00	
EX_fe2(e)	[e] : fe2 <==>	0	0	0.00	
EX_gln-L(e)	[e] : gln-L <==>	0	0	0.00	
EX_glyb(e)	[e] : glyb <==>	0	0	0.00	
EX_h(e)	[e] : h <==>	-Infinity	Infinity	9.55	
EX_h2(e)	[e] : h2 <==>	-10	0	-10.00	
EX_h2o(e)	[e] : h2o <==>	-Infinity	Infinity	0.51	
EX_hcb(e)	[e] : hcb <==>	-Infinity	0	-2.40	
EX_ile-L(e)	[e] : ile-L <==>	0	0	0.00	
EX_k(e)	[e] : k <==>	0	0	0.00	
EX_leu-L(e)	[e] : leu-L <==>	0	0	0.00	
EX_mg2(e)	[e] : mg2 <==>	0	0	0.00	
EX_mn2(e)	[e] : mn2 <==>	0	0	0.00	
EX_mobd(e)	[e] : mobd <==>	0	0	0.00	
EX_n2(e)	[e] : n2 <==>	0	0	0.00	
EX_nh4(e)	[e] : nh4 <==>	-Infinity	Infinity	-0.15	
EX_ni2(e)	[e] : ni2 <==>	0	0	0.00	
EX_pce(e)	[e] : pce <==>	-Infinity	0	0.00	
EX_pi(e)	[e] : pi <==>	-Infinity	Infinity	-0.03	
EX_pro-L(e)	[e] : pro-L <==>	0	0	0.00	
EX_so4(e)	[e] : so4 <==>	-Infinity	Infinity	0.00	
EX_tsul(e)	[e] : tsul <==>	0	0	0.00	
EX_val-L(e)	[e] : val-L <==>	0	0	0.00	
EX_zn2(e)	[e] : zn2 <==>	0	0	0.00	
