# Supplementary material for: Characterizing the Metabolism of Dehalococcoides with a Constraint-Based Model
Source: PLoS Comput Biol. 2010 Aug 19;6(8):e1000887. doi: 10.1371/journal.pcbi.1000887 (PMC2930330; doi:10.1371/journal.pcbi.1000887)
Supplement: Text S2 — This file contains the tables of detailed macromolecular composition of a gram of Dehalococcoides cell, experimental values of various pan-model parameters and the detailed procedure to calculate those parameters, as well as supplemental text regarding energy conservation process of Dehalococcoides. In addition, all the supplemental figures are included at the end of this document. (0.31 MB PDF) [file pcbi.1000887.s003.pdf]

Supplementary Information - 2

Characterizing the Metabolism of *Dehalococcoides* with a Constraint-based Model

M. Ahsanul Islam, Elizabeth A. Edwards, Radhakrishnan Mahadevan*

Department of Chemical Engineering and Applied Chemistry, University of Toronto

Toronto, ON M5S 3E5, Canada

***Corresponding author**

Email addresses:
ahsan.islam@utoronto.ca
elizabeth.edwards@utoronto.ca
krishna.mahadevan@utoronto.ca

Supplementary information - 2: This file contains the tables of detailed macromolecular composition of a gram of *Dehalococcoides* cell, experimental values of various pan-model parameters and the detailed procedure to calculate those parameters, as well as supplemental text regarding energy conservation process of *Dehalococcoides*. In addition, all the supplemental figures are included at the end of this document.

Table of Contents

Table 18. Overall Macromolecular Composition of a *Dehalococcoides* Cell ······ 2
Table 19. Protein Composition of 1 Gram of *Dehalococcoides* Cell ······ 2
Table 20. DNA Composition of 1 Gram of *Dehalococcoides* Cell ······ 3
Table 21. RNA Composition of 1 Gram of *Dehalococcoides* Cell ······ 3
Table 22. Lipid Composition of 1 Gram of *Dehalococcoides* Cell ······ 3
Table 23. Composition of Cofactors and Other Soluble Pools of 1 Gram of *Dehalococcoides* Cell ······ 4
Table 24. Experimental Growth Yields of Various *Dehalococcoides* Cultures ······ 4
Table 25. Experimental Growth Rates of Various *Dehalococcoides* Cultures ······ 7
Table 26. Experimental Decay Rates of Different Anaerobes ······ 7
Table 27. Energy Cost for Processing and Polymerization of Macromolecules (GAM) of a Typical Bacterial Cell ······ 8
Table 28. Standard Gibbs Free Energies for Different Dechlorination Reactions ······ 8
Table 29. Theoretical ATP/e^- and H^+/e^- Ratios of Reductive Dechlorination by *Dehalococcoides* 9
Table 30. Experimental Values of Corrinoid Content of Various Anaerobes ······ 10
Table 31. Growth Rate Simulations with and without the Citrate Synthase (CS) Reaction in the TCA-cycle ······ 10
Supplementary Text ······ 11
- *Dehalococcoides* Biomass Synthesis Reaction ······ 11
- Calculation of *Dehalococcoides* Cell Composition ······ 11
- Calculation of NGAM and GAM Parameters of *i*AI549 ······ 13
- Energy Conservation Process of *Dehalococcoides* ······ 13
- Calculation of Theoretical Maximum Energy Transfer Efficiency (ATP/e^-) and Proton Translocation Stoichiometry (H^+/e^- ratio) of *Dehalococcoides* Electron Transport Chain (ETC) ······ 14

Supplementary Figures ······ 16
- Figure 1. Steps involved to identify *Dehalococcoides* pan-genome ······ 16
- Figure 2. Steps involved to identify *Dehalococcoides* core-genome ······ 17
- Figure 3. Steps involved to identify *Dehalococcoides* unique-genome ······ 18
- Figure 4. Steps involved to identify *Dehalococcoides* dispensable-genome ······ 19
- Figure 5. Reconstructed Wood-Ljungdahl pathway for *Dehalococcoides*. ······ 20
- Figure 6. Tentative scheme for the electron transport chain of *Dehalococcoides* ······ 21
- Figure 7. Distribution of metabolic genes in different subsystems of *i*AI549 ······ 22
- Figure 8. Distribution of gene-associated model reactions in different subsystems of *i*AI549 23

References ······ 24

Table 18. Overall Macromolecular Composition of a *Dehalococcoides* Cell[a]

Protein	63%
RNA	16%
DNA	12%
Lipid	5%
Carbohydrate	1%
Soluble pools and ions	3%
Total	100%

[a]Assumption based on *i*AF692 (*Methanosarcina barkeri* model) [1]. The DNA content is higher than *M. barkeri* because *Dehalococcoides* are disc shaped and smaller in size than *M. barkeri* [2].

Table 19. Protein Composition of 1 Gram of *Dehalococcoides* Cell[a]

Amino acids	Content (mol%)	Content (mmol/g DCW)
L – Alanine	9.58	0.5588
L – Arginine	5.52	0.3220
L – Asparagine	4.5	0.2625
L – Aspartate	4.5	0.2625
L – Cysteine	1.72	0.1003
L – Glutamine	4.91	0.2864
L – Glutamate	4.91	0.2864
Glycine	11.45	0.6679
L – Histidine	1.78	0.1038
L – Isoleucine	5.45	0.3179
L – Leucine	8.45	0.4929
L – Lysine	6.4	0.3733
L – Methionine	2.88	0.1680
L – Phenylalanine	3.47	0.2024
L – Proline	4.15	0.2421
L – Serine	4.05	0.2363
L – Threonine	4.73	0.2759
L – Tryptophan	1.07	0.0624
L – Tyrosine	2.59	0.1511
L – Valine	7.89	0.4603

[a] [21], [24]

Table 20. DNA Composition of 1 Gram of *Dehalococcoides* Cell[a]

dNTPs	Content (mol%)	Content (mmol/g DCW)
dATP	95.5	0.0955
dGTP	84.7	0.0847
dCTP	84.7	0.0847
dTTP	95.5	0.0955

[a]http://img.jgi.doe.gov/cgi-bin/pub/main.cgi

Table 21. RNA Composition of 1 Gram of *Dehalococcoides* Cell[a]

rNTPs	Content (mol%)	Content (mmol/g DCW)
ATP	26.19	0.1289
GTP	32.22	0.1586
CTP	20.00	0.1063
UTP	21.59	0.0985

[a]Elizabeth A. Edwards (personal communication)

Table 22. Lipid Composition of 1 Gram of *Dehalococcoides* Cell[a]

Lipids	Content (mol%)	Content (mmol/g DCW)
Dodecanoic acid (C12:0)	0.91	0.0019
Tetradecanoic acid (C14:0)	7.44	0.0154
Hexadecanoic acid (C16:0)	41.85	0.0865
Octadecanoic acid (C18:0)	18.19	0.0376
Eicosanoic acid (C20:0)	0.49	0.0010
Oleic acid (18:1w9c)	0.35	0.0007
10-R-Methylhexadecanoic acid (10Me16:0)	22.8	0.0471
Dodecanoic acid (C12:0)	0.91	0.0019

[a][25]

Table 23. Composition of Cofactors and Other Soluble Pools of 1 Gram of *Dehalococcoides* Cell[a]

Components	Content (mmol/g DCW)
Putrescine	0.0262
Homospermidine	0.0047
Acetyl-CoA	0.0001
CoA	0.000006
NAD	0.0022
NADH	0.0001
NADP	0.0001
NADPH	0.0004
Succinyl-CoA	0.000003
AMP	0.0010
ADP	0.002
ATP	0.004
5,6,7,8-tetrahydrofolate	0.0001
Adenosylcobalamin	0.0047
Glycogen	0.0154

[a][1]

Table 24. Experimental Growth Yields of Various *Dehalococcoides* Cultures

Dehalococcoides culture	Electron acceptor[a]	Yield (g protein/mol Cl)[b]	Yield (copy/µmol ethene)	Yield (copy/µmol Cl)	Yield (gDCW/mol Cl)[c]	Yield (gDCW/eeq)	Reference
**Pure cultures**							
Strain CBDB1	HCB	**2.1**	-	9.13×10^7	1.11	0.55	[3]
Strain CBDB1	PeCB	**2.9**	-	1.26×10^8	1.53	0.77	[3]
Strain CBDB1	2,3-DCP	**1.73**	-	7.52×10^7	0.91	0.46	[4]
Strain 195	PCE	**4.8**	-	2.9×10^8	2.53	1.27	[5]

***Dehalococcoides* culture**	**Electron acceptor[a]**	**Yield (g protein/mol Cl)[b]**	**Yield (copy/µmol ethene)**	**Yield (copy/µmol Cl)**	**Yield (gDCW/mol Cl)[c]**	**Yield (gDCW/eeq)**	**Reference**
Strain 195	2,3-DCP	-	-	**8.30 x 10^7**	1.01	0.50	[4]
Strain BAV1	VC	-	-	**6.30 x 10^7**	0.76	0.38	[6]
Strain FL2	TCE	-	-	**7.80 x 10^7**	0.95	0.47	[7]
Strain FL2	cis-DCE	-	-	**8.40 x 10^7**	1.02	0.51	[7]
Strain FL2	trans-DCE	-	-	**8.10 x 10^7**	0.98	0.49	[7]
Strain GT	VC	-	-	**2.50 x 10^8**	3.03	1.52	[8]
Strain GT	TCE	-	**9.30 x 10^8**	3.10 x 10^8	3.76	1.88	[8]
Average (± standard deviation)					1.38 (± 1.03)	0.69 (± 0.51)	
**Mixed cultures**							
VS enrichment	VC	-	-	**5.20 x 10^8**	6.31	3.16	[9]
KB1/VC enrichment	VC	-	-	**5.60 x 10^8**	6.80	3.40	[10]
KB1/VC enrichment	TCE	-	-	**3.60 x 10^8**	4.37	2.19	[10]
ANAS enrichment	VC	-	-	**1.30 x 10^7**	0.16	0.08	[11]
ANAS enrichment	cis-DCE	-	-	**1.2 x 10^7**	0.15	0.07	[11]

***Dehalococcoides* culture**	**Electron acceptor[a]**	**Yield (g protein/mol Cl)[b]**	**Yield (copy/µmol ethene)**	**Yield (copy/µmol Cl)**	**Yield (gDCW/mol Cl)[c]**	**Yield (gDCW/eeq)**	**Reference**
ANAS enrichment	TCE	-	-	**1.4×10^{7}**	0.17	0.08	[11]
JN culture	PCB	-	-	**9.25×10^{8}**	11.23	5.61	[12]
KB1/TCE enrichment	1,2-DCA	-	**3.20×10^{8}**	1.60×10^{8}	1.94	1.94	[2]
KB1/TCE enrichment	VC	-	**2.90×10^{8}**	2.90×10^{8}	3.52	1.76	[2]
KB1/TCE enrichment	cis-DCE	-	**3.50×10^{8}**	1.75×10^{8}	2.12	1.06	[2]
Average (± standard deviation)[e]					4.18 (± 2.05)	2.25 (± 0.88)	

[a]Short forms for electron acceptors are: HCB, Hexachlorobenzene; PeCB, Pentachlorobenzene; PCB, Polychlorinated biphenyls; 2,3-DCP, 2,3-Dichlorophenol; PCE, Tetrachloroethene; VC, Vinyl chloride; TCE, Trichloroethene; cis-DCE, cis-Dichloroethene; trans-DCE, trans-Dichloroethene; 1,2-DCA, 1,2-Dichloroethane.

[b]A conversion factor of 2.3×10^{-14} g protein $cell^{-1}$ is used to convert the numbers in g protein to copy [4].

[c]Copy numbers are converted to gram dry cell weight (gDCW) by assuming cylindrical shape, 0.5 µm diameter, 0.2 µm thickness and 70% water content of a *Dehalococcoides* cell as well as 1 copy of the 16S rRNA gene per genome or per cell.

[d]Bold numbers are yield values cited in the literature.

[e]Average and standard deviation of mixed cultures are calculated without including ANAS and JN cultures' yield since those are outliers. ANAS yields are based on long term experiments where dechlorination and growth may be uncoupled [11]. JN yield is likely to be inaccurate due to difficulties in measuring PCB concentration.

Table 25. Experimental Growth Rates of Various *Dehalococcoides* Cultures

***Dehalococcoides* culture**	**Electron acceptor[a]**	**Growth rate (d^{-1})**	**Growth rate (h^{-1})**	**Reference**
**Pure cultures**				
Strain CBDB1	2,3-DCP	0.41	0.017	[4]
Strain 195	PCE	1.26	0.053[b]	[13]
Strain BAV1	VC	0.32	0.013	[6]
Strain GT	VC	0.35	0.014	[8]
Strain FL2	VC	0.29	0.012	[7]
Average (± standard deviation)			0.014 (± 0.002)	
**Mixed cultures**				
VS enrichment	TCE	0.35	0.015	[14]
VS enrichment	cis-DCE	0.46	0.019	[14]
VS enrichment	VC	0.49	0.020	[14]
KB1/VC enrichment	TCE	0.33	0.014	[14]
KB1/VC enrichment	cis-DCE	0.44	0.018	[14]
KB1/VC enrichment	VC	0.42	0.018	[14]
Average (± standard deviation)			0.017 (± 0.003)	

[a]Short forms for electron acceptors are: 2,3-DCP, 2,3-Dichlorophenol; VC, Vinyl chloride; PCE, Tetrachloroethene; TCE, Trichloroethene; cis-DCE, cis-Dichloroethene.

[b]Growth rate calculation was not substantiated; hence, not used in calculating average.

Table 26. Experimental Decay Rates of Different Anaerobes

**Organism**	**Decay rate (d^{-1})**	**Reference**
Dehalococcoides sp. strain VS (during growth)	0.05	[9]
Dehalococcoides sp. strain VS (no growth)	0.09	[9]

Organism	Decay rate (d^{-1})	Reference
Methanobacterium bryantii	0.088	[13]
Typical for anaerobes	0.02	[15]

Table 27. Energy Cost for Processing and Polymerization of Macromolecules (GAM) of a Typical Bacterial Cell[a]

Process	mmol/g DCW	µmol ATP/µmol	mmol ATP/g DCW
**Protein**			
Activation	5.8333	4.0000	23.3332
mRNA synthesis		0.2000	1.1667
Proofreading		0.1000	0.5833
Assembly/modification		0.0060	0.1399
**RNA**			
Discarding segments	0.4923	0.3800	0.1871
Modification		0.0200	0.0098
**DNA**			
Unwinding helix	0.3604	1.0000	0.3604
Proofreading		0.3600	0.1297
Discontinuous synthesis		0.0060	0.0022
Negative supercoiling		0.0050	0.0018
Methylation		0.0010	0.0004
**Total cost**			**25.9145**

[a][21]

Table 28. Standard Gibbs Free Energies for Different Dechlorination Reactions

Electron donor	Electron acceptor	Product	Reaction $\Delta G_0'$ (kJ/mol)	Reference
Hydrogen	Tetrachloroethene	Trichloroethene	-175.31	[15,16]

Electron donor	Electron acceptor	Product	Reaction $\Delta G_0'$ (kJ/mol)	Reference
Hydrogen	Trichloroethene	Dichloroethene	-166.31	[15,16]
Hydrogen	Dichloroethene	Chloroethene	-145.71	[15,16]
Hydrogen	Chloroethene	Ethene	-151.35	[15,16]
Hydrogen	Hexachlorobenzene	Pentachlorobenzene	-171.40	[17]
Hydrogen	Pentachlorobenzene	Tetrachlorobenzene	-164.07	[17]
Hydrogen	Tetrachlorobenzene	Trichlorobenzene	-164.3	[17]
Hydrogen	Trichlorobenzene	Dichlorobenzene	-152.77	[17]
**Average $\Delta G_0'$**			**-161.40**	

Table 29. Theoretical ATP/e^- and H^+/e^- Ratios of Reductive Dechlorination by *Dehalococcoides*

Average $\Delta G_0'$ (kJ/mol)	ATP/e^- ratio (maximum)	Proton translocation/mole ATP	H^+/e^- ratio		(Assumed/Maximum) X 100 (Energy transfer efficiency)	ATP/e^- (Assumed)
			Maximum	Assumed		
161.40	1.64	3	4.92	5	100	1.64
				4	80	1.30
				3	60	1.00
				2	40	0.66
				1	20	0.33

Table 30. Experimental Values of Corrinoid Content of Various Anaerobes

Organism	Corrinoid content (literature values)	Corrinoid content (mmol/gDCW)	*Dehalococcoides* yield prediction by *i*AI549 during corrinoid salvage from the medium (gDCW/eeq)	*Dehalococcoides* yield prediction by *i*AI549 during *de novo* corrinoid synthesis (gDCW/eeq)	Reference
Clostridium cochlearium	30 nmol/g wet mass	0.00015	0.713	0.713	[18]
Acetobacterium woodii	650 nmol/g dry mass	0.00065	0.713	0.713	[19]
Clostridium formicoaceticum	950 nmol/g dry mass	0.00095	0.713	0.713	[19]
Sporomusa ovata	3100 nmol/g dry mass	0.0031	0.713	0.710	[19]
Methanosarcina barkeri (used in *i*AI549)	-	0.0047	0.713	0.709	[20]
10X *Methanosarcina barkeri*	-	0.047	0.707	0.676	Assumption

Table 31. Growth Rate Simulations with and without the Citrate Synthase (CS) Reaction in the TCA-cycle

Exchange reactions	Flux values without the CS reaction (mmol/gDCW.h)	Flux values with the CS reaction (mmol/gDCW.h)
Acetate exchange, EX_ac(e)	0.1820	0.1943
Cobalamin exchange, EX_cbl1(e)	0.0001	0.0001
Carbon dioxide exchange, EX_co2(e)	0.1741	0.1383
Hydrogen exchange, EX_h2(e)	10.0000	10.0000
Chloride exchange,	9.6067	9.6793

Exchange reactions	Flux values without the CS reaction (mmol/gDCW.h)	Flux values with the CS reaction (mmol/gDCW.h)
EX_Cl(e)		
**Parameters**	**Without the CS reaction**	**With the CS reaction**
Growth rate	0.014 h^{-1}	0.0137 h^{-1}
Growth yield	0.72 gDCW/eeq	0.71 gDCW/eeq

Supplementary Text

Dehalococcoides Biomass Synthesis Reaction

The detail macromolecular composition of one (1) gram of *Dehalococcoides* cell, presented in Tables S18-S23, as well as the GAM (61 mmol ATP. $gDCW^{-1}$) has been included in *i*AI549 as a biomass synthesis reaction: BIO_DHC_DM_61.

Considering a basis of 1 gram dry cell weight, the biomass synthesis equation is defined as:

0.0001 mmol Acetyl-CoA + 0.0047 mmol Adenosylcobalamin + 0.5588 mmol L-alanine + 0.001 mmol AMP + 0.3320 mmol L-arginine + 0.2625 mmol L-asparagine + 0.2625 mmol L-aspartate + 61 mmol ATP + 0.000006 mmol CoenzymeA + 0.1063 mmol CTP + 0.1003 mmol L-cysteine + 0.0955 mmol dATP + 0.0847 mmol dCTP + 0.0019 mmol Dodecanoic acid + 0.0847 mmol dGTP + 0.0955 mmol dTTP + 0.0471 mmol 10-R-Methylhexadecanoic acid + 0.2684 mmol L-glutamine + 0.2684 mmol L-glutamate + 0.6679 mmol Glycine + 0.0154 mmol Glycogen + 0.1586 mmol GTP + 61 mmol H_2O + 0.0865 mmol Hexadecanoic acid + 0.1038 mmol L-histidine + 0.0047 mmol Homospermidine + 0.001 mmol Eicosanoic acid + 0.3179 mmol L-isoleucine + 0.4929 mmol L-leucine + 0.3733 mmol L-lysine + 0.1680 mmol L-methionine + 0.0022 mmol NAD + 0.0001 mmol NADH + 0.0001 mmol NADP + 0.0004 mmol NADPH + 0.0376 mmol Octadecanoic acid + 0.0007 mmol Oleic acid + 0.0347 mmol L-phenylalanine + 0.0415 mmol L-proline + 0.0262 mmol Putrescine + 0.0405 mmol L-serine + 0.000003 mmol Succinyl-CoA + 0.0001 mmol 5,6,7,8-tetrahydrofolate + 0.2759 mmol L-threonine + 0.0624 mmol L-tryptophan + 0.0154 mmol Tetradecanoic acid + 0.1511 mmol L-tyrosine + 0.0985 mmol UTP + 0.4603 mmol L-valine ----> 61 mmol ADP + 61 mmol H^+ + 61 mmol Inorganic Phosphate

Calculation of *Dehalococcoides* Cell Composition

The shape of *Dehalococcoides* cell is reported to be cylindrical [2].
Diameter of one *Dehalococcoides* cell = 0.5 μm [2]
Thickness of one *Dehalococcoides* cell = 0.2 μm

Hence, the volume of one *Dehalococcoides* cell = $\pi r^2 h = \pi\left(\frac{D}{2}\right)^2 h = \pi\left(\frac{0.5}{2}\right)^2 0.2$

$= 0.0393\ \mu m^3$

Assume, cell density is equal to the density of water = 1.03 g/ml.
Therefore, mass of one *Dehalococcoides* cell = 1.03 g/ml x 3.93 x 10^{-2} μm^3 x 10^{-12} ml/μm^3
= 4.05 x 10^{-14} g

Typically, a bacterial cell has 70% water [21]
Hence, dry mass of one *Dehalococcoides* cell = 4.05 x 10^{-14} x 0.3 g
= 1.21 x 10^{-14} g

Length of *Dehalococcoides* DNA (roughly) = 1.4 x 10^6 base pairs (bp) [22]

Assume, the average molecular mass of a nucleotide or 1 bp = 666 g/mol
Hence, the molar mass of a *Dehalococcoides* genome = 1.4 x10^6 x 666 g/mol
Since, 1 mole of nucleotide = 6.023 x 10^{23} molecules of nucleotide

Therefore,
The mass of 1 molecule of *Dehalococcoides* nucleotide (or genome)
= (1.4 x10^6 x 666)/(6.023 x 10^{23}) g
= 1.55 x 10^{-15} g

So, the percentage of DNA in 1 gram dry cell mass $= \left(\frac{1.55 \times 10^{-15}}{1.21 \times 10^{-14}} \right) \times 100\% = 12.75\%$

We know, the amount of RNA in a 50 ml culture = 50 μg (Elizabeth A. Edwards, personal communication)
So, 1 ml of culture contains 1 μg of RNA.

Also, 1 ml of similar culture contains 1 x 10^7 ~ 5 x 10^8 copies of *Dehalococcoides* cells (Elizabeth A. Edwards, personal communication)

Assuming that 1 ml of culture has 5 x 10^8 copies of *Dehalococcoides* cells.
Hence, the dry mass of 5 x 10^8 cells = 5 x 10^8 x 1.21 x 10^{-14} g = 6.05 x 10^{-6} g
So, 6.05 x 10^{-6} g of cells has 1 x 10^{-6} g of RNA

Therefore,
The percentage of RNA in 1 gram dry cell mass $= \left(\frac{1 \times 10^{-6}}{6.05 \times 10^{-6}} \right) \times 100 = 16.53\,\%$

Since, the experimental data for estimating the percentage contents of other components of a *Dehalococcoides* cell were not available, the corresponding estimates from the published *Methanosarcina barkeri* model [1] that included protein, lipid, carbohydrate, and soluble pools and ions were used in this model.

In order to determine the amount of individual component of the macromolecules of a *Dehalococcoides* cell, physiological data from various published models of different

microorganisms [1,21,23,24] have been used. The contents of different fatty acids were calculated from White et al. [25].

Calculation of NGAM and GAM Parameters of *i*AI549

Non-growth associated (NGAM) and growth associated maintenance (GAM) parameters for *Dehalococcoides* were estimated using the published data from [3,4,6,7,8,9,14,21,26] and the equation from [27,28,29], as well as simulations in SimPheny™.

The non-growth associated ATP maintenance is given by

$$m = \frac{b}{Y_G}$$

where, b = specific maintenance rate or decay rate (d^{-1})

Y_G = True growth yield or yield without maintenance (g DCW/eeq)

Assuming Y_G = Y= Observed growth yield for *Dehalococcoides* bacteria,

$$m = \frac{b}{Y}$$

Using pure culture growth yield, Y = 0.69 gDCW/eeq (Table 24) and b = 0.09 d^{-1} (Table 26), the calculated NGAM for *Dehalococcoides* bacteria is

$$m = \frac{0.09 \times 1 \times 1000}{0.69 \times 24 \times 3} = 1.8 \text{ mmol ATP/g DCW.h}$$

Energy Conservation Process of *Dehalococcoides*

Dehalococcoides strains respire through a membrane-bound electron transport chain (ETC) [30,31,32], which is incompletely defined. In addition to RDase and hydrogenase (H_2ase) enzymes, the ETC of *Dehalococcoides* requires an *in vivo* electron carrier to mediate electron transport between H_2ase and RDase. The reductive dechlorination reaction requires an *in vivo* electron donor of redox potential (E_0') ≤-360 mV [30,32] similar to other dechlorinating bacteria [33,34,35]. The cob(II)alamin of corrinoid cofactor in the RDase enzyme is reduced to cob(I)alamin during the reductive dechlorination reaction; hence, necessitating a low-potential donor because the redox potential (E_0') of Co(II)/Co(I) couple is between -500 and -600 mV [33,34,36]. While quinones, such as menaquinone or ubiquinone could act as electron carriers in anaerobes [37,38,39], experimental evidence suggests this is not the case in *Dehalococcoides* [3,32]. Moreover, the half reaction potentials for quinones (Menaquinone ox/red E_0' = -70 mV, Ubiquinone ox/red E_0' = +113 mV; [40]) are not compatible with RDases that require a donor of E_0' ≤-360 mV.

Therefore, we hypothesize that ferredoxin could be a low-potential electron donor for the RDase of *Dehalococcoides* because it is the most electronegative electron carrier yet found in the bacterial ETCs [41,42]. Various redox potentials had been reported for bacterial ferredoxins,

which included -417 mV at pH 7.55 for *Clostridium pasteurianum* [43], -398 and -367 mV in the range of pH 6.13 to 7.41 for *C. pasteurianum* [40,44], -445 mV at pH 7 for *Dehalospirillum multivorans* [35], -453 mV at pH 8 for *Thermotoga maritima* [45]. While these experimental data illustrate the differences in ferredoxin potential across microbes, it also supports their putative role as a low-potential electron carrier in the *Dehalococcoides* ETC. Furthermore, there was strong genomic evidence that the sequences of *rdh* genes contained two iron-sulfur cluster binding motifs, which are the characteristic motifs for bacterial ferredoxins [46]. So far, the genomes of *Dehalococcoides* have 6 putative ferredoxin-encoding genes (Tables 3-7 in Text S1), but no gene was identified for a b-type cytochrome. Miller and colleagues [35] described a mechanism for the ETC of *D. multivorans* involving both H_2ase and RDase enzymes where they propose the "reverse electron transport", and the requirement of both a low-potential and a high-potential electron carrier for the ETC. Recently, Thauer et al. [47] suggested that the energy conservation process of methanogens without cytochromes (a system similar to *Dehalococcoides*) used a flavin-based "electron bifurcation" system where an endergonic reaction was driven by the energy from an exergonic reaction that took place simultaneously. A similar bifurcation mechanism was also proposed for the trimeric [Fe]-only H_2ase of *T. maritima* [48]. Based on the literature and considering the lack of information on the *Dehalococcoides* ETC, we propose the following simplified mechanism of energy conservation for its ETC (Figure 6).

We assumed that the H_2ase of *Dehalococcoides* reduced ferredoxin in a similar process as described for *M. barkeri* [47,49,50,51]. Subsequently, the reduced ferredoxin was assumed to transfer electrons to terminal electron acceptors, such as chloroethenes or chlorobenzenes (RX) via cob(II)alamin where cob(II)alamin was reduced to cob(I)alamin [33,34], and RX was reduced to lower chlorinated compounds or ethenes (RH). Alternatively, the endergonic reduction of ferredoxin with H_2 could be coupled to the exergonic reduction of RX with reduced ferredoxin where the later reaction was catalyzed by RDase in a similar manner as the electron bifurcation scheme. This might be possible because a corrinoid protein, like a flavo-protein, could also be a site for electron bifurcation (R. K. Thauer, personal communication). In either case, we assume that the uptake of two protons ($2H^+$) from the cytoplasm occurs during the transfer of $2e^-$ from the donor H_2 to the acceptor RX; thus, resulting in a net proton translocation stoichiometry of 1 H^+ per e^- (Figure 6) .

Calculation of Theoretical Maximum Energy Transfer Efficiency (ATP/e$^-$) and Proton Translocation Stoichiometry (H$^+$/e$^-$ ratio) of *Dehalococcoides* Electron Transport Chain (ETC)

The theoretical maximum ATP/e$^-$ ratio, $(\eta_{ATP}/\eta_e)_{max}$ can be determined from the following equation [37]:

$$\left(\eta_{ATP} / \eta_e \right)_{\max} = \Delta E_0{}' F / \Delta G_P{}' \qquad (1)$$

where, F is the Faraday constant (96,500 J/mol .V), $\Delta E_0{}'$ is the difference in standard redox potential between the electron donor and acceptor, and $\Delta G_P{}'$ is the free energy of phosphorylation reaction at pH 7 and physiological condition.

Since, $\Delta G_0{}' = -nF\Delta E_0{}'$

Therefore, $\left(\eta_{ATP} / \eta_e \right)_{\max} = \Delta G_0{}' / n\Delta G_P{}'$ (2)

Where n is the number of electrons transferred in the reaction

$\Delta G_P{}'$ at physiological conditions can be calculated from the free energy of the phosphorylation reaction at standard conditions and pH 7 ($\Delta G_{0,p}{}'$) using the following equation:

$$\Delta G_p{}' = \Delta G_{0,p}{}' + RT \ln\left(\frac{[ATP]}{[ADP][P_i]} \right) \quad (3)$$

where, $\Delta G_{0,p}{}'$ = 32 kJ/mol [40], R is the universal gas constant having a value of 8.314 J/mol.K and T is the absolute temperature, 298.15 K at 25 ºC.

Assuming that the concentrations of ATP and ADP are equal and that the concentration of P_i is 1 mM, then the calculated value of $\Delta G_P{}'$ using equation (3) is 49.12 kJ/mol.

The average standard free energy for dechlorination was found to be $\Delta G_0{}'$ of -161.40 kJ/mol (Table 28).

Therefore, theoretical maximum ATP/e^- using equation (2) is:

$$\left(\eta_{ATP} / \eta_e \right)_{\max} = \frac{161.40}{2 \times 49.12} = 1.64$$

Assuming the number of H^+ translocated across the cell membrane during the phosphorylation of ADP is 3 [52], we obtain the theoretical maximum H^+/e^- of dechlorination process is 4.92 (Table 29) which means, the H^+/e^- should be either 5 or 4.

Since the ATP/e^- value (0.33) corresponding to 1 H^+/e^- (Table 29) was found to be in agreement with the experimental ATP/e^- value of 0.6 mol ATP/mol Cl^- [35,53,54], the proton translocation stoichiometry of *Dehalococcoides* ETC was chosen as 1 H^+/e^-.

Supplementary Figures

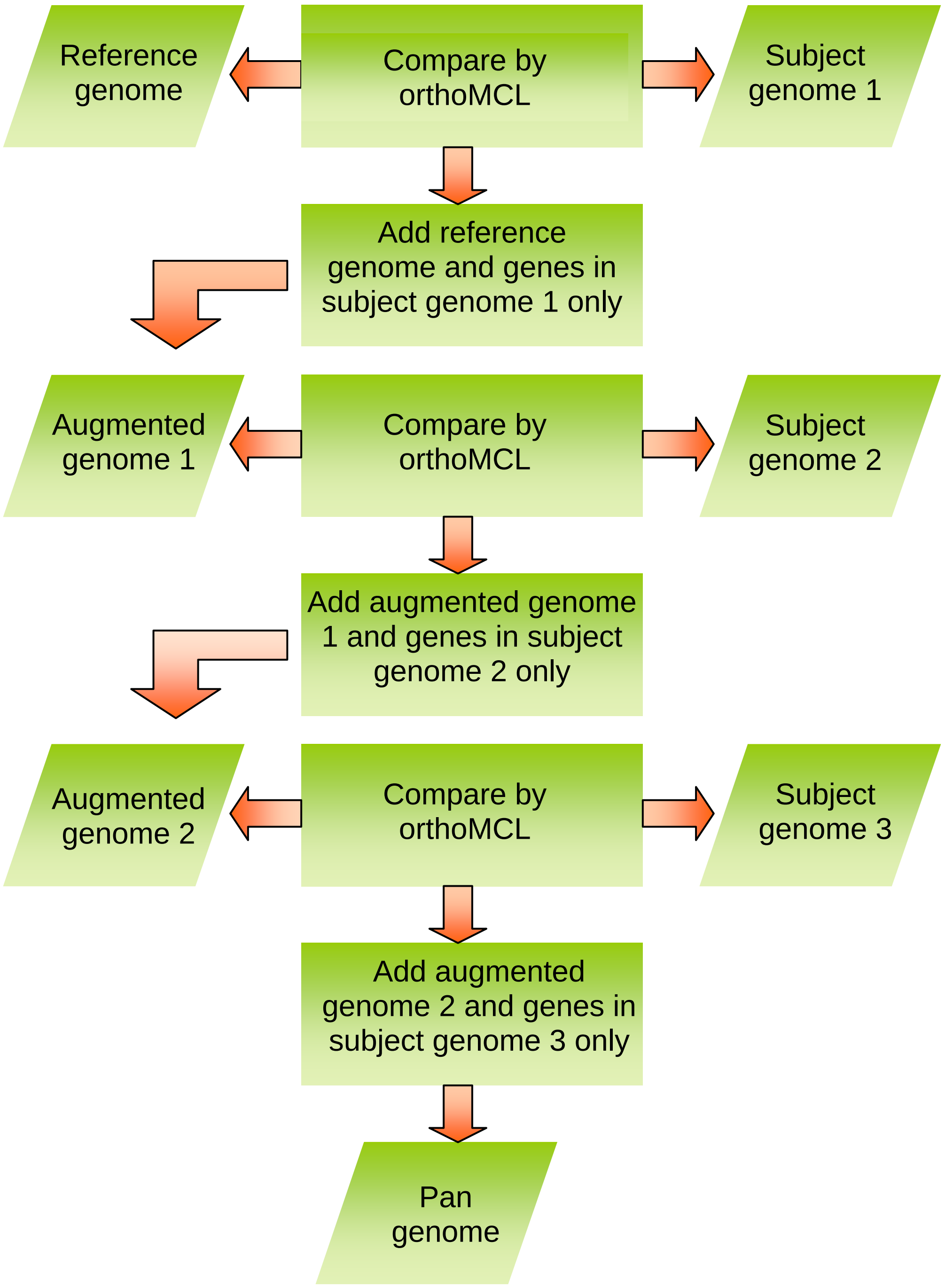

Figure 1. Steps involved to identify *Dehalococcoides* pan-genome

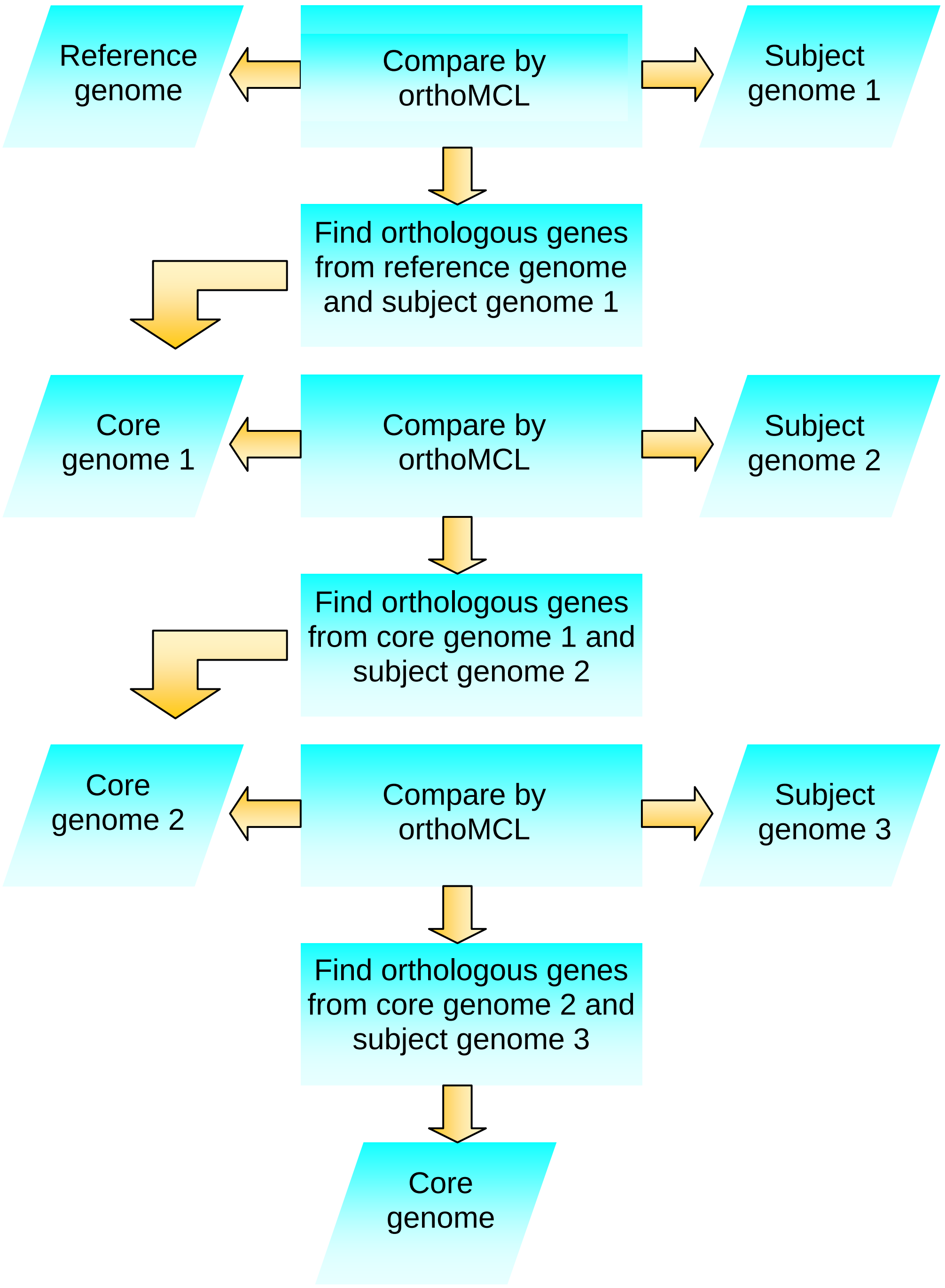

Figure 2. Steps involved to identify *Dehalococcoides* core-genome

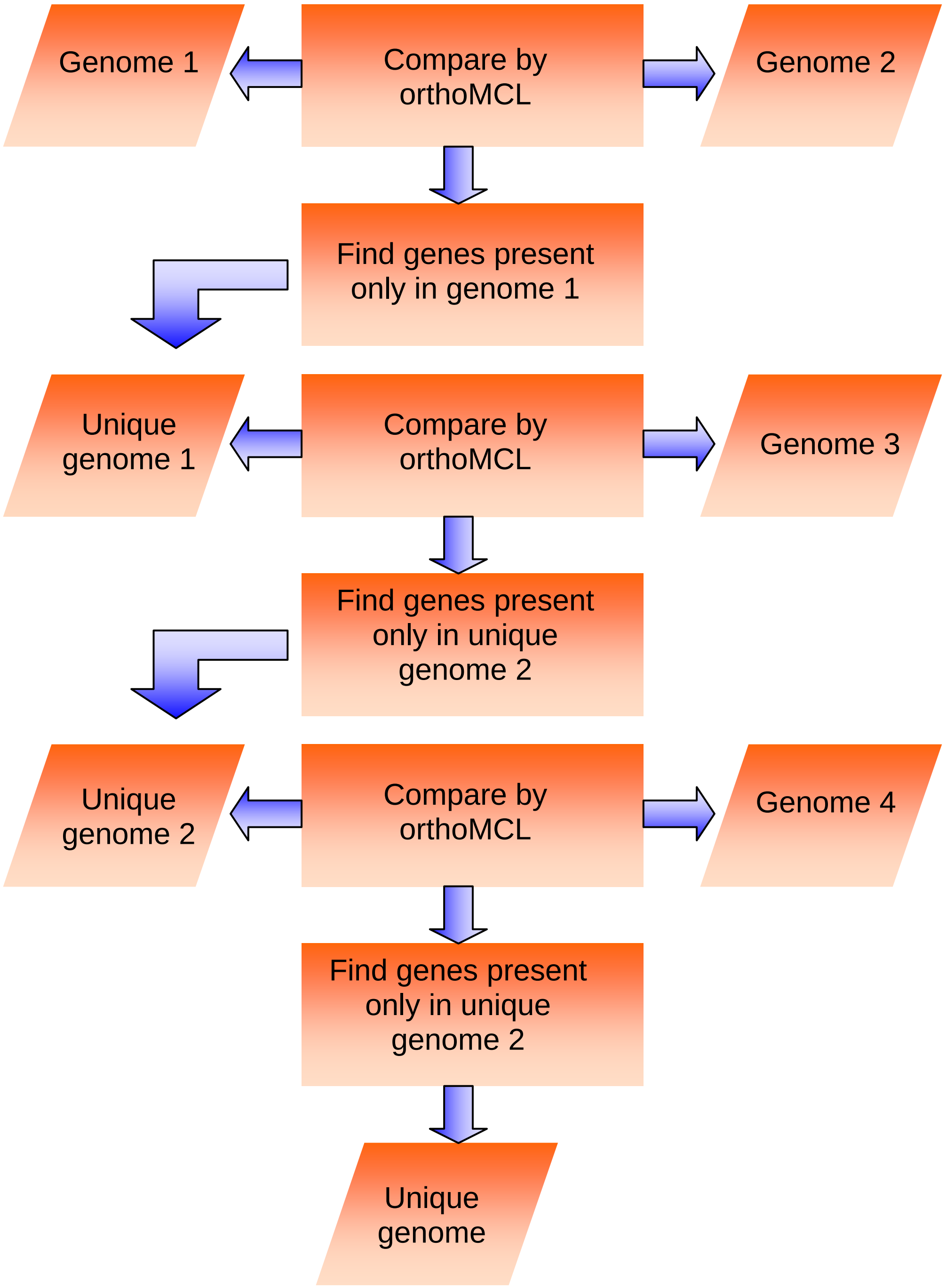

Figure 3. Steps involved to identify *Dehalococcoides* unique-genome

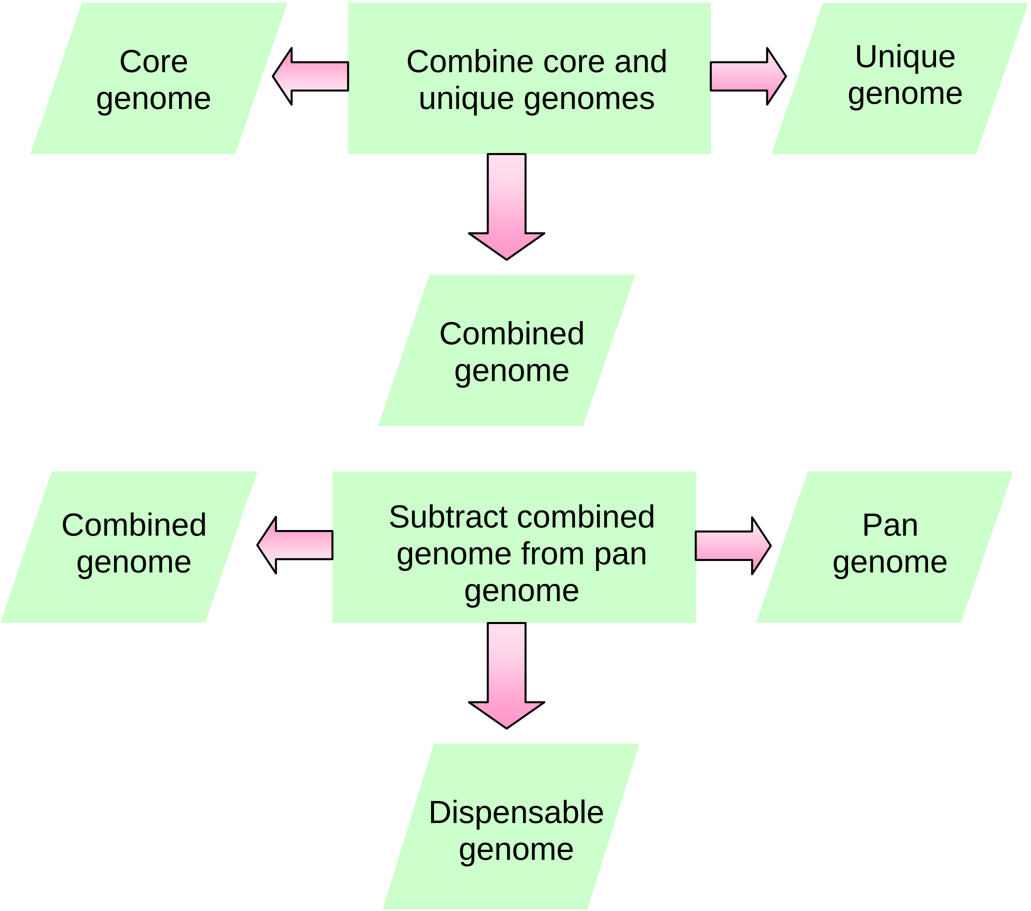

Figure 4. Steps involved to identify *Dehalococcoides* dispensable-genome

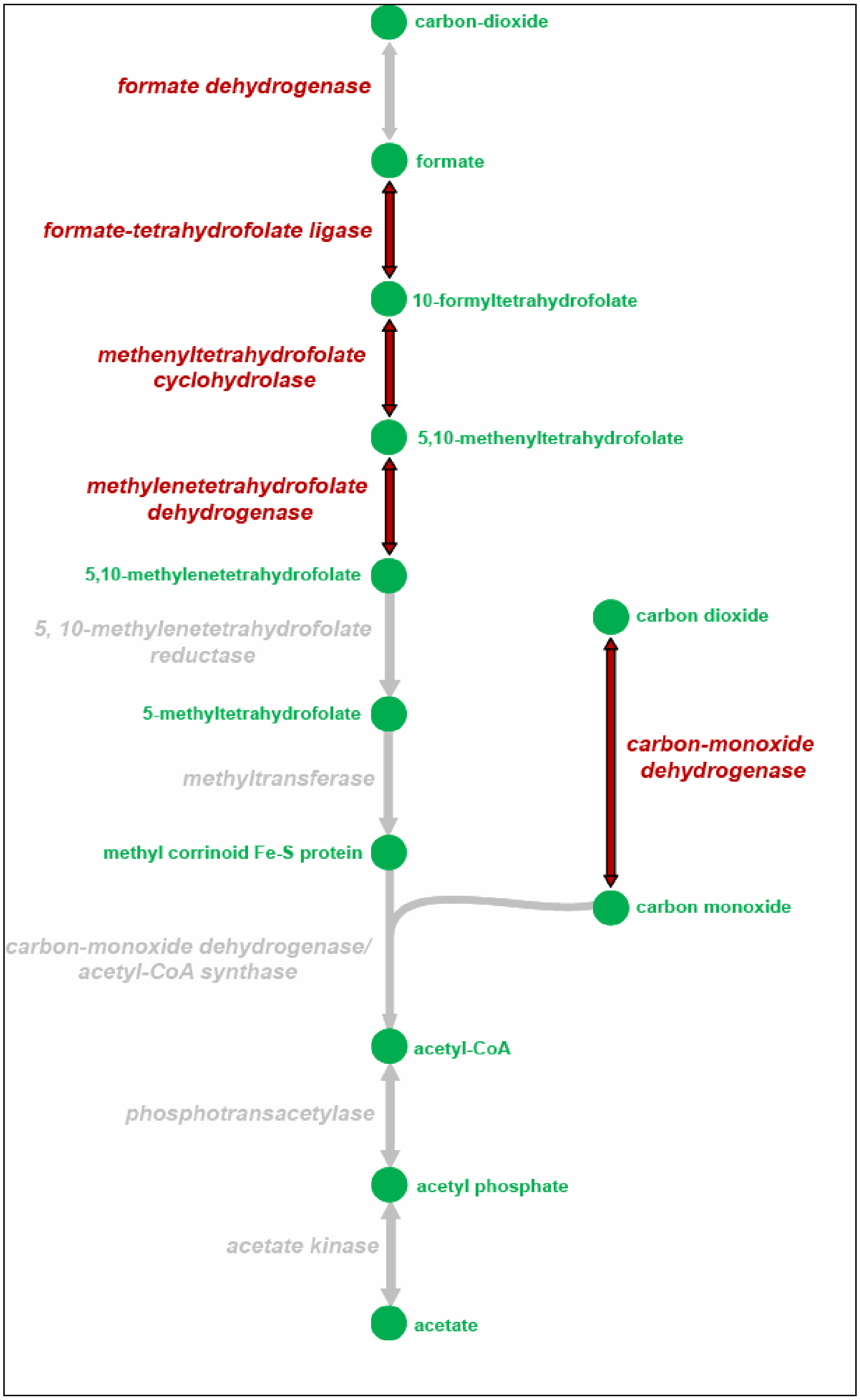

Figure 5. Reconstructed Wood-Ljungdahl pathway for *Dehalococcoides*. Grey lines indicate missing pathways and red lines indicate existing pathways, the genes of which are identified in the genomes of *Dehalococcoides* during the reconstruction of *i*AI549. The arrows are denoting the directionality of the reactions. Due to the missing enzymes, *Dehalococcoides* seem not to be able to fix carbon or CO_2 by the Wood-Ljungdahl pathway which is also supported by the result from Tang *et al* [55].

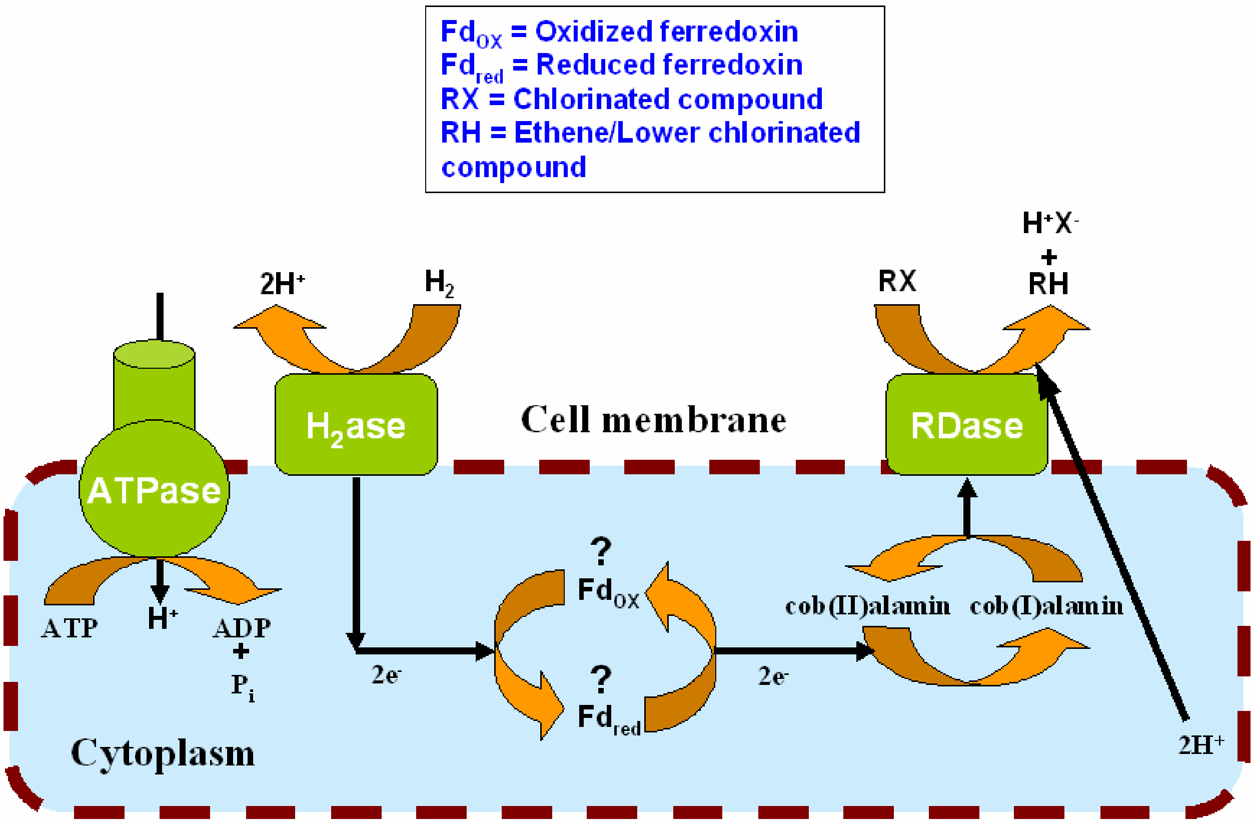

Figure 6. Tentative scheme for the electron transport chain of *Dehalococcoides*

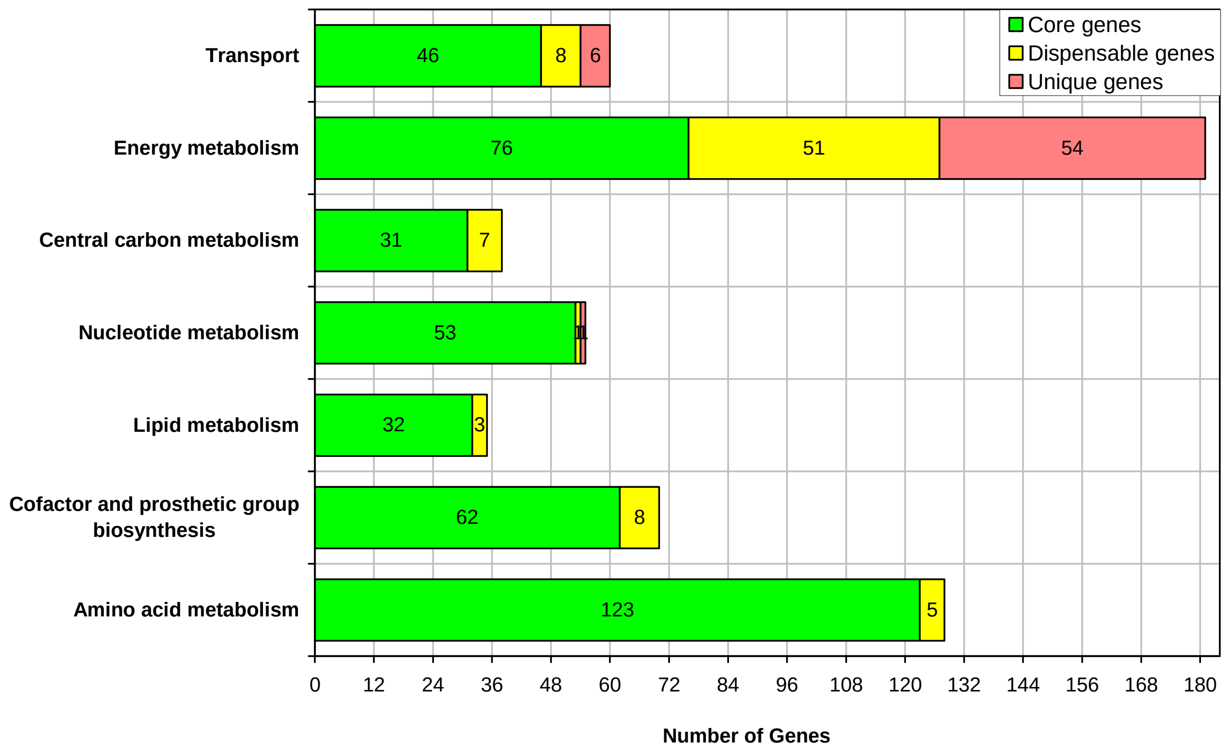

Figure 7. Distribution of metabolic genes in different subsystems of *i*AI549

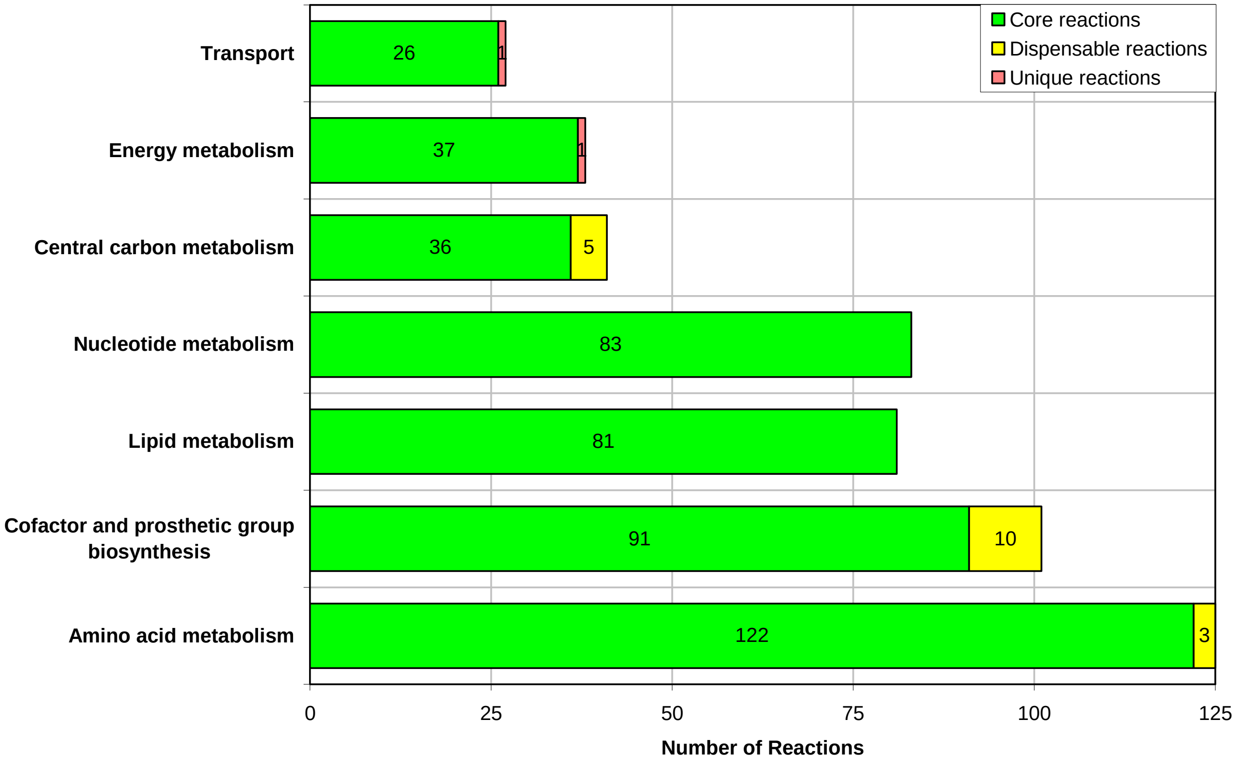

Figure 8. Distribution of gene-associated model reactions in different subsystems of *i*AI549

References

1. Feist AM, Scholten JCM, Palsson BO, Brockman FJ, Ideker T (2006) Modelling methanogenesis with a genome-scale metabolic reconstruction of *Methanosarcina barkeri*. Molecular Systems Biology 2: 1-14.
2. Duhamel M, Edwards EA (2007) Growth and yields of dechlorinators, acetogens, and methanogens during reductive dechlorination of chlorinated ethenes and dihaloelimination of 1,2-Dichloroethane. Environmental Science & Technology 41: 2303-2310.
3. Jayachandran G, Gorisch H, Adrian L (2003) Dehalorespiration with hexachlorobenzene and pentachlorobenzene by *Dehalococcoides* sp. strain CBDB1. Arch Microbiol 180: 411-416.
4. Adrian L, Hansen SK, Fung JM, Gorisch H, Zinder SH (2007) Growth of *Dehalococcoides* strains with chlorophenols as electron acceptors. Environmental Science &Technology 41: 2318-2323.
5. Maymo-Gatell X, Chien Y-t, Gossett JM, Zinder SH (1997) Isolation of a bacterium that reductively dechlorinates tetrachloroethene to ethene. Science 276: 1568-1571.
6. He J, Ritalahti KM, Yang K-L, Koenigsberg SS, Loffler FE (2003) Detoxification of vinyl chloride to ethene coupled to growth of an anaerobic bacterium. Nature 424: 62-65.
7. He J, Sung Y, Brown RK, Ritalahti KM, Loffler FE (2005) Isolation and characterization of *Dehalococcoides* sp. strain FL2, a trichloroethene (TCE)- and 1,2- dechloroethene-respiring anaerobe. Environmental Microbiology 7: 1442-1450.
8. Sung Y, Ritalahti KM, Apkarian RP, Loffler FE (2006) Quantitive PCR confirms purity of strain GT, a novel trichloroethene-to-ethene-respiring *Dehalococcoides* isolate. Applied and Environmental Microbiology 72: 1980-1987.
9. Cupples AM, Spormann AM, McCarty PL (2003) Growth of a *Dehalococcoides* - like microorganism on vinyl chloride and cis - dichloroethene as electron acceptors as determined by competitive PCR. Applied and Environmental Microbiology 69: 953-959.
10. Duhamel M, Mo K, Edwards EA (2004) Characterization of a highly enriched *Dehalococcoides*-containing culture that grows on vinyl chloride and trichloroethene. Applied and Environmental Microbiology 70: 5538-5545.
11. Holmes VF, He J, Lee PKH, Alvarez-Cohen L (2006) Discrimination of multiple *Dehalococcoides* strains in a trichloroethene enrichment by quantification of their reductive dehalogenase genes. Applied and Environment Microbiology 72: 5877-5883.
12. Bedard DL, Ritalahti KM, Loffler FE (2007) The *Dehalococcoides* population in sediment-free mixed cultures metabolically dechlorinates the commercial polychlorinated biphenyl mixture Aroclor 1260. Applied and Environment Microbiology 73: 2513-2521.
13. Karadagli F, Rittmann BE (2005) Kinetic characterization of *Methanobacterium bryantii* M.o.H. Environmental Science & Technology 39: 4900-4905.
14. Cupples AM, Spormann AM, McCarty PL (2004) Vinyl chloride and cis-Dichloroethene dechlorination kinetics and microorganism growth under substrate limiting conditions. Environmental Science & Technology 38: 1102-1107.
15. Rittmann BE, McCarty PL (2001) Environmental biotechnology: principles and applications. New York: McGraw-Hill. 754 p.
16. Dolfing J, Janssen DB (1994) Estimates of Gibbs free energies of formation of chlorinated aliphatic compounds. Biodegradation 5: 21-28.

17. Dolfing J, Harrison BK (1992) Gibbs free energy of formation of halogenated aromatic compounds and their potential role as electron acceptors in anaerobic environments. Environmental Science & Technology 26: 2213-2218.
18. Hoffmann B, Oberhuber M, Stupperich E, Bothe H, Buckel W, et al. (2000) Native corrinoids from *Clostridium cochlearium* are adeninylcobamides: spectroscopic analysis and identification of pseudovitamin B(12) and factor A. Journal of Bacteriology 182: 4773-4782.
19. Stupperich E, Eisinger HJ, Kräutler B (1988) Diversity of corrinoids in acetogenic bacteria. P-cresolylcobamide from *Sporomusa ovata,* 5-methoxy-6-methylbenzimidazolylcobamide from *Clostridium formicoaceticum* and vitamin B12 from *Acetobacterium woodii*. European Journal of Biochemistry 172: 459-464.
20. Gorris L, van der Drift C (1994) Cofactor contents of methanogenic bacteria reviewed. Biofactors 4: 139-145.
21. Neidhardt FC, Ingraham JL, Schaechter M (1990) Physiology of the bacterial cell: a molecular approach. Sunderland, Massachusetts: Sinauer Associates, Inc.
22. Kube M, Beck A, Zinder SH, Kuhl H, Reinhardt R, et al. (2005) Genome sequence of the chlorinated compound respiring bacterium *Dehalococcoides* species strain CBDB1. Nature Biotechnology 23: 1269-1273.
23. Mahadevan R, Bond DR, Butler JE, Esteve-Nuñez A, Coppi MV, et al. (2006) Characterization of metabolism in the Fe (III)-reducing organism *Geobacter sulfurreducens* by constraint-based modeling. Applied and Environmental Microbiology 72: 1558-1568.
24. Pramanik J, Keasling JD (1997) Stoichiometric model of *Escherichia coli* metabolism: incorporation of growth-rate dependent biomass composition and mechanistic energy requirements. Biotechnology and Bioengineering 56: 398-421.
25. White DC, Geyer R, Peacock AD, Hedrick DB, Koenigsberg SS, et al. (2005) Phospholipid furan fatty acids and Ubiquinone-8: lipid biomakers that may protect *Dehalococcoides* strains from free radicals. Applied and Environmental Microbiology 71: 8426-8433.
26. Simmonds A (2007) Dechlorination rates in KB-1, a commercial trichloroethylene degrading bacterial culture. MASc Thesis [MASc Thesis]. Toronto ON Canada: University of Toronto.
27. Pirt SJ (1965) The maintenance of bacteria in growing cultures. Proceedings of the Royal Society of London, Series B: Biological Sciences 163: 224-231.
28. Pirt SJ (1982) Maintenance energy: a general model for energy-limited and energy-sufficient growth. Arch Microbiol 133: 300-302.
29. Russell JB, Cook GM (1995) Energetics of bacterial growth: balance of anabolic and catabolic reactions. Microbiological Reviews 59: 48-62.
30. Holscher T, Gorisch H, Adrian L (2003) Reductive dehalogenation of chlorobenzene congeners in cell extracts of *Dehalococcoides* sp. strain CBDB1. Applied and Environmental Microbiology 69: 2999-3001.
31. Jayachandran G, Gorisch H, Adrian L (2004) Studies on hydrogenase activity and chlorobenzene respiration in *Dehalococcoides* sp. strain CBDB1. Arch Microbiol 182: 498-504.
32. Nijenhuis I, Zinder SH (2005) Characterization of hydrogenase and reductive dehalogenase activities of *Dehalococcoides ethenogenes* strain 195. Applied and Environmental Microbiology 71: 1664-1667.

33. Holliger C, Wohlfarth G, Diekert G (1999) Reductive dechlorination in the energy metabolism of anaerobic bacteria. FEMS Microbiology Reviews 22: 383-398.
34. Krasotkina J, Walters T, Maruya KA, Ragsdale SW (2001) Characterization of the B12 and iron-sulfur-containing reductive dehalogenase from *Desulfitobacterium chlororespirans*. The Journal of Biological Chemistry 276: 40991-40997.
35. Miller E, Wohlfarth G, Diekert G (1997) Studies on tetrachloroethene respiration in *Dehalospirillum multivorans*. Arch Microbiol 166: 379-387.
36. Banerjee R, Ragsdale SW (2003) The many faces of vitamin B12: catalysis by cobalamin-dependent enzymes. Annual Review of Biochemistry 72: 209-247.
37. Kroger A, Biel S, Simon J, Gross R, Unden G, et al. (2002) Fumarate respiration of *Wolinella succinogens*: enzymology, energetics and coupling mechanism. Biochimica et Biophysica Acta 1553: 23-38.
38. Louie TM, Mohn WW (1999) Evidence for a chemiosmotic model of dehalorespiration in *Desulfomonile tiedjei* DCB-1. Journal of Bacteriology 181: 40-46.
39. Schumacher W, Holliger C (1996) The proton/electron ration of the menaquinone-dependent electron transport from dihydrogen to tetrachloroethene in "*Dehalobacter restrictus*". Journal of Bacteriology 178: 2328-2333.
40. Thauer RK, Jungermann K, Decker K (1977) Energy conservation in chemotrophic anaerobic bacteria. Bacteriological reviews 41: 100-180.
41. Bruschi M, Guerlesquin F (1988) Structure, function and evolution of bacterial ferredoxins. FEMS Microbiology Reviews 54: 155-176.
42. Valentine RC (1964) Bacterial ferredoxin. Bacteriological reviews 28: 497-517.
43. Valentine RC, Wolfe RS (1963) Role of ferredoxin in the metabolism of molecular hydrogen. Journal of Bacteriology 85: 1114-1120.
44. Eisenstein KK, Wang JH (1969) Conversion of light to chemical free energy. The Journal of Biological Chemistry 244: 1720-1728.
45. Sterner R (2001) Ferredoxin from *Thermotoga maritima*. Methods in Enzymology 334: 23-30.
46. Holscher T, Brown RK, Ritalahti KM, Wintzingerode Fv, Gorisch H, et al. (2004) Multiple nonidentical reductive-dehalogenase-homologous genes are common in *Dehalococcoides*. Applied and Environmental Microbiology 70: 5290-5297.
47. Thauer RK, Kaster A-K, Seedorf H, Buckel W, Hedderich R (2008) Methanogenic archaea: ecologically relevant differences in energy conservation. Nature Reviews: Microbiology 6: 579-591.
48. Schut GJ, Adams MWW (2009) The iron-hydrogenase of *Thermotoga maritima* utilizes ferredoxin and NADH synergistically: a new perspective on anaerobic hydrogen production. Journal of Bacteriology 191: 4451-4457.
49. Deppenmeier U (2002) Redox-driven proton translocation in methanogenic Archaea. Cellular and Molecular Life Sciences 59: 1513-1533.
50. Deppenmeier U (2004) The membrane-bound electron transport system of Methanosarcina species. Journal of Bioenergetics and Biomembranes 36: 55-64.
51. Hedderich R (2004) Energy-converting [NiFe] hydrogenases from archaea and extremophiles: ancestors of complex I. Journal of Bioenergetics and Biomembranes 36: 65-75.

52. Harold FM, Maloney PC (1996) Energy transduction by ion currents. In: Neidhardt FC, Curtiss R, Ingraham JL, Lin ECC, Low KB et al., editors. *Escherichia coli* and *Salmonella* Cellular and molecular biology. Washington, D. C.: ASM Press. pp. 283-306.
53. Jayachandran G (2004) Physiological and enzymatic studies of respiration in *Dehalococcoides* species strain CBDB1. PhD Thesis. [PhD]: Technischen Universität Berlin, Germany.
54. Loubiere P, Lindely ND (1991) The use of acetate as additional co- substrate improves methylotropic growth of the acetogeneic anaerobe *Eubacterium limosum* when CO_2 fixation is rate limiting. Journal of General Microbiology 137: 2247-2251.
55. Tang YJ, Yi S, Zhuang W-Q, Zinder SH, Keasling JD, et al. (2009) Investigation of carbon metabolism in "*Dehalococcoides ethenogenes*" strain 195 using isotopomer and transcriptomic analyses. Journal of Bacteriology 191: 5224-5231.
